# Visible-Light-Induced Alkoxyl Radicals Enable α-C(sp^3^)-H Bond Allylation

**DOI:** 10.1016/j.isci.2019.100755

**Published:** 2019-12-04

**Authors:** Jing Zhang, Dan Liu, Song Liu, Yuanyuan Ge, Yu Lan, Yiyun Chen

**Affiliations:** 1 State Key Laboratory of Bioorganic and Natural Products Chemistry, Center for Excellence in Molecular Synthesis, Shanghai Institute of Organic Chemistry, University of Chinese Academy of Sciences, Chinese Academy of Sciences, 345 Lingling Road, Shanghai 200032, China; 2 School of Chemistry and Chemical Engineering, Chongqing University, Chongqing 400030, China; 3 Green Catalysis Center, College of Chemistry, Zhengzhou University, Zhengzhou, Henan 450001, China

**Keywords:** Organic Chemistry, Organic Reaction, Physical Organic Chemistry

## Abstract

The alkoxyl radical is an essential reactive intermediate in mechanistic studies and organic synthesis with hydrogen atom transfer (HAT) reactivity. However, compared with intramolecular 1,5-HAT or intermolecular HAT of alkoxyl radicals, the intramolecular 1,2-HAT reactivity has been limited to theoretical studies and rarely synthetically utilized. Here we report the first selective 1,2-HAT of alkoxyl radicals for α-C(sp^3^)-H bond allylation of α-carbonyl, α-cyano, α-trifluoromethyl, and benzylic N-alkoxylphthalimides. The mechanistic probing experiments, electron paramagnetic resonance (EPR) studies, and density functional theory (DFT) calculations confirmed the 1,2-HAT reactivity of alkoxyl radicals, and the use of protic solvents lowered the activation energy by up to 10.4 kcal/mol to facilitate the α-C(sp^3^)-H allylation reaction.

## Highlights

• 1,2-Hydrogen atom transfer (HAT) of alkoxyl radical enables α-C(sp^3^)-H allylation
• α-Carbonyl, α-cyano, α-trifluoromethyl, and benzylic C(sp^3^)-H bonds are applicable
• Mechanistic and electron paramagnetic resonance (EPR) studies confirmed 1,2-HAT
• DFT calculations explained the methanol acceleration of alkoxyl radical 1,2-HAT

## Graphical Abstract

**Figure:**
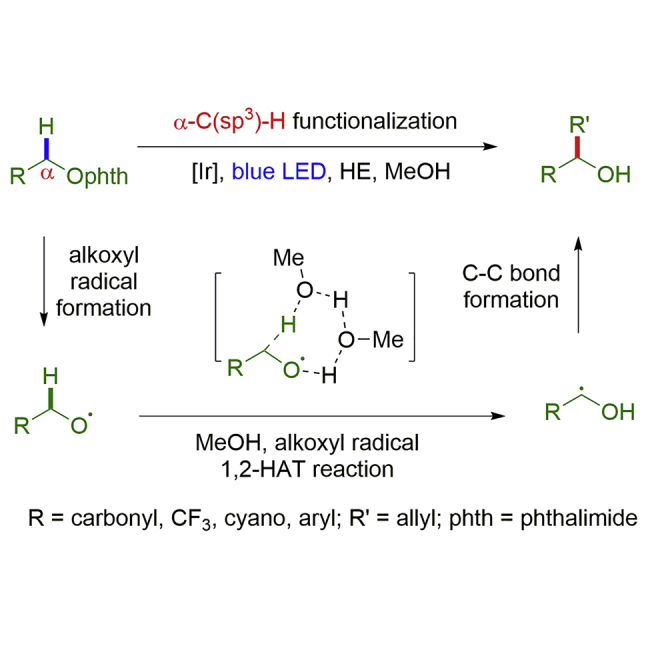

## Introduction

**Scheme 1:**
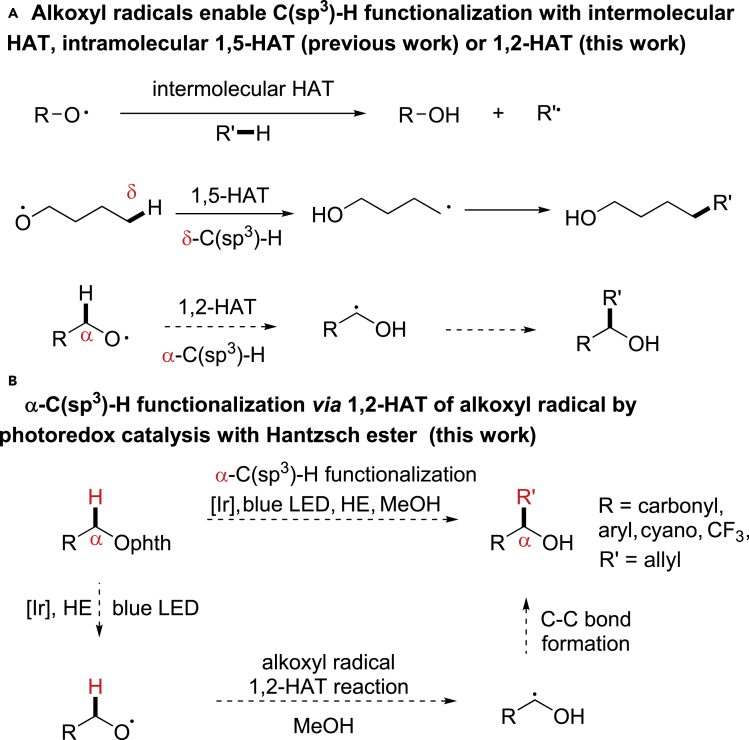
Selective C(sp^3^)-H Functionalization via Hydrogen Atom Transfer of Alkoxyl Radicals phth, Phthalimide. (A) Alkoxyl radicals enable C(sp^^3^^)-H functionalization with intermolecular HAT, intramolecular 1,5-HAT or 1,2-HAT. (B) α-C(sp^^3^^)-H allylation via 1,2-HAT of alkoxyl radical by photoredox catalysis with Hantzsch ester.

The selective inert C(sp^3^)-H bond activation for new C-C bond formation is very desirable in organic synthesis (Chen et al., 2009, Lyons and Sanford, 2010, Prier et al., 2013, Gensch et al., 2016, Yi et al., 2017). The hydroxyl groups are ubiquitous in organic molecules, and the use of hydroxyl derivatives provides an effective tool to differentiate chemically indistinguishable C-H bonds (Holmes et al., 2018, Engle et al., 2012, Ren et al., 2012, Wappes et al., 2017, Espino et al., 2001, Simmons and Hartwig, 2012a, Chen et al., 2008, Chen et al., 2015, Karmel et al., 2018). The alkoxyl radical is an essential reactive intermediate in mechanistic studies and organic synthesis, and its highly reactive character enables unactivated C-H bond functionalization with the hydrogen atom transfer (HAT) reactivity (Čeković, 2003, Čeković, 2005, Hartung, 2001, Chiba and Chen, 2014, Lundgren et al., 2006, Salamone et al., 2011, Salamone et al., 2012, Salamone et al., 2013, Salamone et al., 2014a, Salamone et al., 2014b, Salamone et al., 2016a, Salamone et al., 2016b, Bietti and Salamone, 2014, Salamone and Bietti, 2015). When intramolecular δ-C-H bonds are present within the molecule, the 1,5-HAT reaction of alkoxyl radicals preferentially occurs to abstract the δ-C-H; otherwise, the intermolecular HAT reaction dominates (Scheme 1A) (Dorigo and Houk, 1988, Robertson et al., 2001, Weavers, 2001, Burke et al., 1988, Petrovic et al., 2004, Zhu et al., 2009, Zhu et al., 2015, Rueda-Becerril et al., 2011, Hu et al., 2018, Wu et al., 2018a, Wu et al., 2018b, Guan et al., 2018). In contrast, the intramolecular C-H abstraction at positions other than δ-position by alkoxyl radicals has been less reported owing to the unfavorable transition states and high activation energies (Čeković, 2003, Čeković, 2005, Hartung, 2001, Chiba and Chen, 2014). Currently, there are only a few reports on the 1,2-HAT reactivity of alkoxyl radicals in theoretical or biological studies, and the synthetic utilization of 1,2-HAT for new C-C bond formation remains elusive (Buszek et al., 2011, Elford and Roberts, 1996, Fernández-Ramos and Zgierski, 2002, Konya et al., 2000, Gilbert et al., 1976, Che et al., 2016, Che et al., 2018). Here we report the first visible-light-induced α-C(sp^3^)-H allylation reaction enabled by the selective 1,2-HAT of alkoxyl radicals, which is facilitated by protic solvents and applicable to various α-carbonyl, α-cyano, α-trifluoromethyl, and benzylic C(sp^3^)-H bonds (Scheme 1B).

## Results and Discussion

### Optimization of the Reaction Conditions

**Scheme 2:**
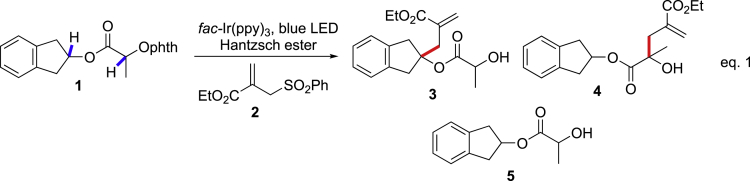
1,2-HAT Reaction of N-alkoxylphthalimide 1

Our investigation was initiated by the serendipitous discovery with N-alkoxylphthalimide **1** as the alkoxyl radical precursor, which can be readily prepared from alcohols and are bench-stable (Scheme 2) (Zhu et al., 2009, Kim et al., 1998, Zhang et al., 2016, Zhang et al., 2017, Wang et al., 2016, Ito et al., 2018, Han et al., 2019, Deng et al., 2019, Shi et al., 2019). Under the reaction conditions of *fac*-Ir(ppy)_3_ and Hantzsch ester known to generate alkoxyl radicals (Zhang et al., 2016, Zhang et al., 2017, Wang et al., 2016, Ito et al., 2018, Han et al., 2019, Deng et al., 2019, Shi et al., 2019), the ester-derived N-alkoxylphthalimide **1** gave no δ-C(sp^3^)-H allylation adduct **3** with allyl sulfone **2** under blue LED irradiation. Instead, the α-C(sp^3^)-H allylation adduct **4** was observed in 41% yield, together with the hydrogenation adduct alcohol **5** in 52% yield (entry 1 in Table 1) (Zhang et al., 2016). These results were in sharp contrast with our previous observation on the reactivity of alkoxyl radicals under photocatalysis conditions (Zhang et al., 2016, Zhang et al., 2017). We then tested the addition of acids or bases to the reaction and found the outcomes of the reaction were not significantly affected (entries 2–3). The further screen of different Hantzsch ester derivatives has little effect on the reaction (entries 4–6) (Chen et al., 2016). Significantly, the use of ethanol or methanol as solvents dramatically improved the α-C(sp^3^)-H allylation adduct to 93%–97% yields (93% isolated yield, entries 7–8) and minimalized the hydrogenation adduct alcohol **5** formations. The mixed protic solvents were also beneficial that the addition of methanol or water improved the reaction of dioxane from 41% to 52%–66% yields (entries 9–10) (see Tables S2 and S3).

**Table 1:** Discovery and Optimization of the 1,2-HAT of Alkoxyl Radicals for α-C(sp^3^)-H Allylation

Entry	Conditions^a^	4 Yield (%)^b^	5 Yield (%)^b^
1	Dioxane	41	52
2	Entry 1, 2.0 equiv. Na_2_CO_3_	56	42
3	Entry 1, 2.0 equiv. HCO_2_H	40	56
4	Entry 1, COOMe-HE	43	56
5	Entry 1, COO^i^Pr-HE	47	52
6	Entry 1, COO^t^Bu-HE	45	54
7	EtOH	93	7
8	MeOH	97 (93)	<5
9	MeOH/dioxane = 1:9	52	45
10	H_2_O/dioxane = 1:9	66	33

a Reaction conditions: **1** (0.10 mmol, 1.0 equiv.), **2** (0.30 mmol, 3.0 equiv.), *fac*-Ir(ppy)_3_ (0.001 mmol, 1%), and Hantzsch ester (0.15 mmol, 1.5 equiv.) in 1.0 mL solvent under nitrogen with 4 W blue LED irradiation at ambient temperature for 3 h, conversion was >95%, unless otherwise noted.

b Conversion and yields were determined by ^1^H NMR analysis, and isolated yields are in parentheses.

### Scope

We next explored the scope of this 1,2-HAT reaction for other substrates (Scheme 3). The glycol-derived **6** without ring strain at the δ-C-H bonds afforded **7** in 89% yield, without the observation of the δ-C-H allylation adducts. The benzyl ester **8** with the activated benzylic δ-C-H bonds gave the α-C(sp^3^)-H allylation adduct **9** in 92% yield. The N-alkoxylphthalimides **10** and **12** provided 88% and 95% yields of α-C-H allylation adducts, successfully, with the ketone or the free hydroxyl group unaffected. The N-alkoxylphthalimide **14** with the amide linkage provided the 1,2-HAT adduct **15** smoothly in 52% yield, together with 17% yield of hydrogenation adduct as the side product (See Figures S1–S26).

**Scheme 3:**
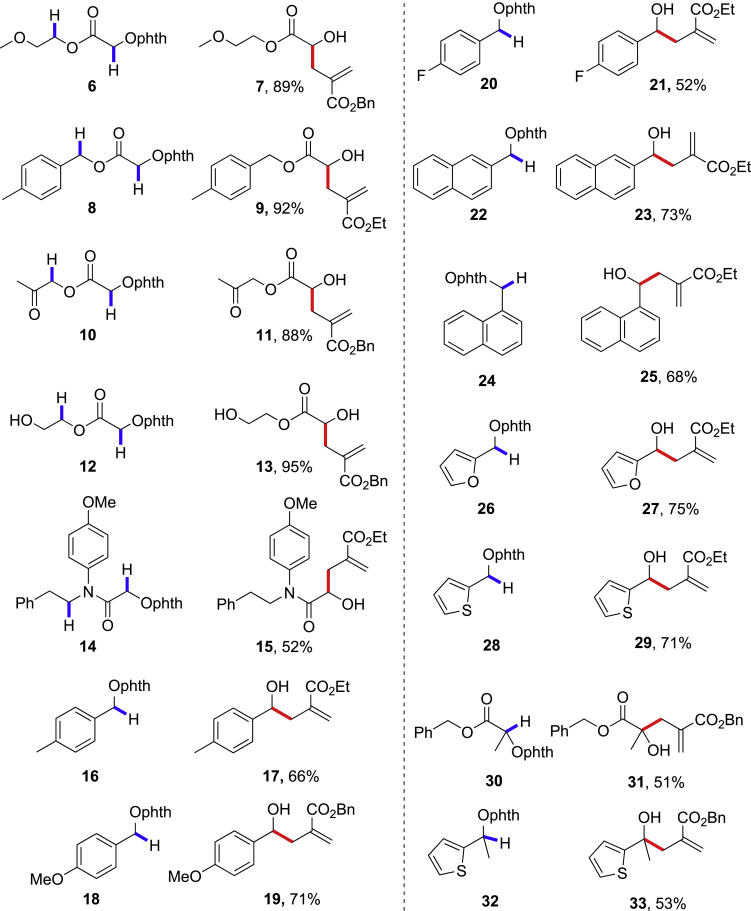
Substrate Scope of the 1,2-HAT Reactions Reaction condition is in entry 8 in Table 1, and isolated yields are reported.

The 1,2-HAT reaction is also applicable to N-alkoxylphthalimides without the ester or amide linkages. The N-alkoxylphthalimide **16** with benzyl C(sp^3^)-H bonds gave the α-C(sp^3^)-H allylation adduct **17** in 66% yield (Scheme 3). The incorporation of electron-rich methoxyl group on the phenyl ring slightly improved the reaction to give **19** in 71% yield, whereas the electron-deficient fluorides decreased the reaction to give **21** in 52% yield. The α- and β-substituted naphthalenes reacted nicely to give **23** and **25** in 68%–73% yields. The heterocyclic furans and thiophenes reacted to provide **27** and **29** in 71%–75% yields. The secondary-alcohol-derived N-alkoxylphthalimides **30** and **32** gave the corresponding tertiary homoallylic alcohols **31** and **33** in 51% and 53% yields, respectively (See Figures S27–S64).

**Scheme 4:**
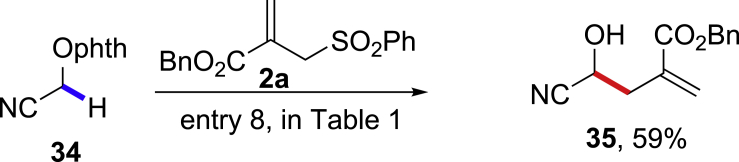
1,2-HAT Reaction of N-alkoxylphthalimide 34

**Scheme 5:**
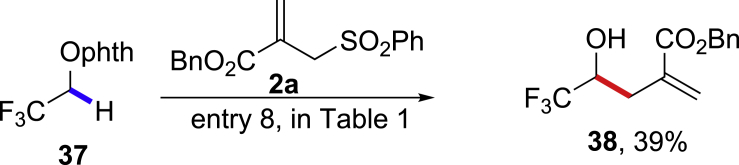
1,2-HAT Reaction of N-alkoxylphthalimide 38

**Scheme 6:**
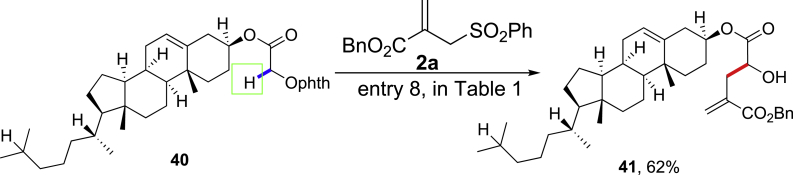
1,2-HAT Reaction of N-alkoxylphthalimide 40

This reaction is particularly valuable for the homoallylic alcohol synthesis when the corresponding aldehydes are inaccessible by the nucleophilic addition methods (Yamamoto and Asao, 1993, Yus et al., 2011). The cyano-substituted homoallylic alcohol **35** can be obtained from the stable N-alkoxylphthalimide **34** by 1,2-HAT reaction smoothly in 59% yield, and the corresponding formyl cyanide **36** is unstable and cannot be synthetically utilized (Scheme 4) (Lewis-Bevan et al., 1992). Similarly, the trifluoromethyl-substituted homoallylic alcohol **37** can be prepared from the stable N-alkoxylphthalimide **38** in 39% yield, whereas the trifluoromethyl aldehyde **39** is very unstable and volatile (Scheme 5) (Ishikawa et al., 1984, Loh and Li, 1999). We also tested a structurally complexed steroid derivative **40** with multiple tertiary and allylic C-H bonds, which are challenging substrates to differentiate the targeted α-C-H bonds by intermolecular HAT reactions (Scheme 6) (Roberts, 1999, Chu and Rovis, 2018). Gratifyingly, the α-C(sp^3^)-H allylation adduct **41** was selectively obtained in 62% yield, leaving other six tertiary C-H and four allylic C-H bonds untouched (see Figures S65–S78).

### Mechanistic Investigations

**Scheme 7:**
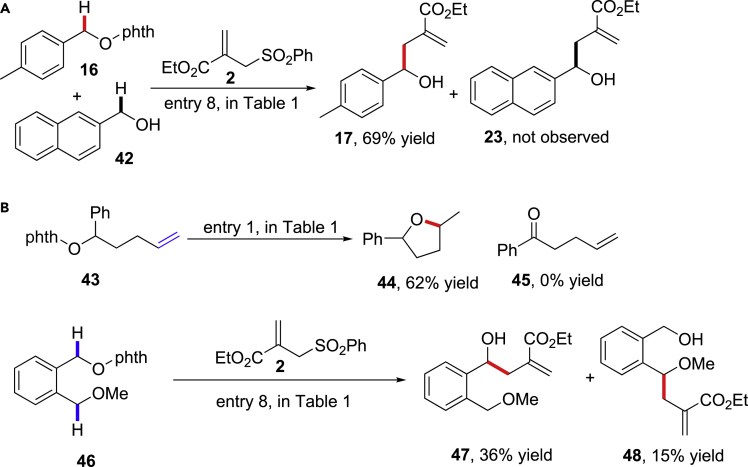
Mechanistic Investigations of the 1,2-HAT of Alkoxyl Radicals (A) The crossover experiment with N-alkoxylphthalimide ****16**** and alcohol ****42****. (B) The investigation of potential reaction pathways of alkoxyl radicals.

We next carried out mechanistic investigations (Scheme 7). The possible intermolecular hydrogen atom transfer pathway instead of the 1,2-HAT was first evaluated by crossover experiments (Schemes 7A and S1). With N-alkoxylphthalimide **16** and a structurally similar alcohol **42**, the reaction with allylsulfone **2** gave the exclusive homoallylic alcohol **17** from **16** in 69% yield, whereas the formation of **23** from **42** was not observed. This result together with the chemoselective α-C(sp^3^)-H allylation demonstrated in Scheme 6 excluded the intermolecular HAT reaction pathway. We then compared the 1,2-HAT with other potential reaction pathways of alkoxyl radicals in different N-alkoxylphthalimides (Scheme 7B). With benzyl alcohol-derived N-alkoxylphthalimide **43** bearing a pendant alkene at the δ-position, the tetrahydrofuran **44** was obtained via the preferential 5-exo cyclization of alkoxyl radicals, whereas neither the α-C(sp^3^)-H allylation adduct nor the oxidized ketone adduct **45** was observed (Zlotorzynska and Sammis, 2011). With benzyl alcohol-derived N-alkoxylphthalimide **46** bearing activated δ-C-H bonds, the α-C(sp^3^)-H allylation adduct **47** was observed in 36% yield, together with the δ-C(sp^3^)-H allylation adduct **48** in 15% yield (see Scheme S3 for details). These results confirmed the presence of alkoxyl radicals and suggested other alkoxyl radical reaction pathways may be favored over 1,2-HAT pathway in certain substrates (the KIE (k_H_/k_D_) with deuterated N-alkoxylphthalimide was measured to be 0.87, suggesting the cleavage of the C-H bond was not the rate-determining step, see Table S1, Scheme S2 and Simmons and Hartwig, 2012b) (see Figures S79–S96).

**Scheme 8:**
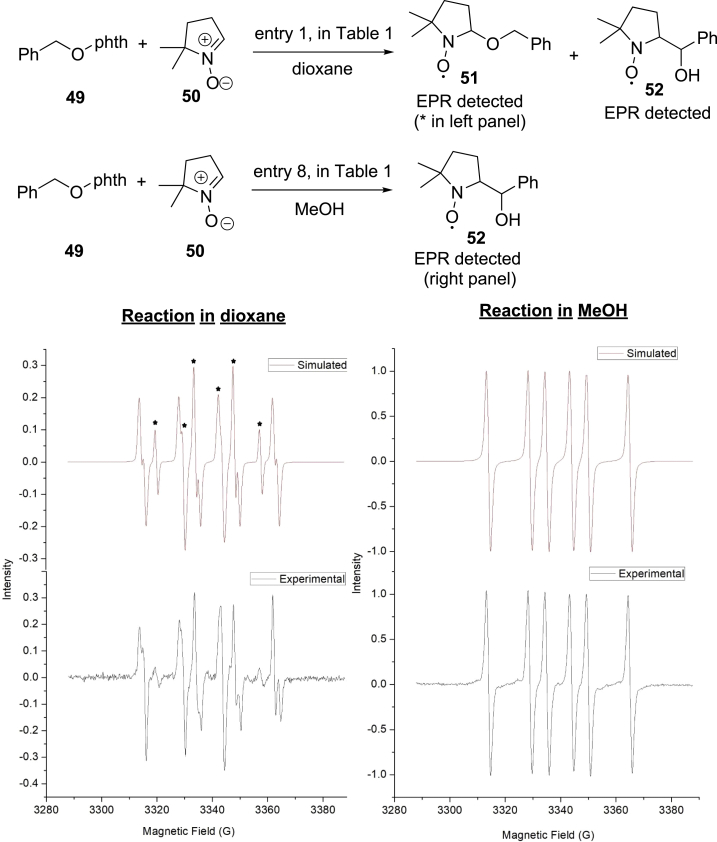
EPR Studies of the 1,2-HAT of Alkoxyl Radicals

We further investigated the radical intermediates in the reaction by the electron paramagnetic resonance measurements (EPR) using 5,5-dimethyl-pyrroline N-oxide (DMPO) **50** as the radical spin trap. Scheme 8 illustrates the EPR spectrum from the addition of DMPO to the reaction of N-alkoxylphthalimide **49** (see Schemes S11–S13). The spectrum can be fit as the admixture of a triplet of doublets (a_N_ = 14.1 G, a_H_ = 9.6 G) and a triplet of doublets (a_H_ = 14.2 G, a_H_ = 19.7 G) in dioxane. The first triplet of doublets is attributed to DMPO-trapped alkoxyl radical **51** (asterisk ∗ signals in the left panel), and the second triplet of doublets is attributed to DMPO-trapped ketyl radical **52**. However, only the ketyl radical trapping adduct **51** (right panel) could be observed in methanol. These results were consistent with the increased hydrogenation adduct from alkoxyl radicals in dioxane compared with in methanol, which indicated that the 1,2-HAT process of alkoxyl radicals to yield ketyl radicals was accelerated in methanol (the Stern-Volmer plots suggest the Hantzsch ester quenched the photoexcited fac-Ir(ppy)_3_ more effectively than N-alkoxylphthalimides and allyl sulfones; see Schemes S4–S8 for details).

**Scheme 9:**
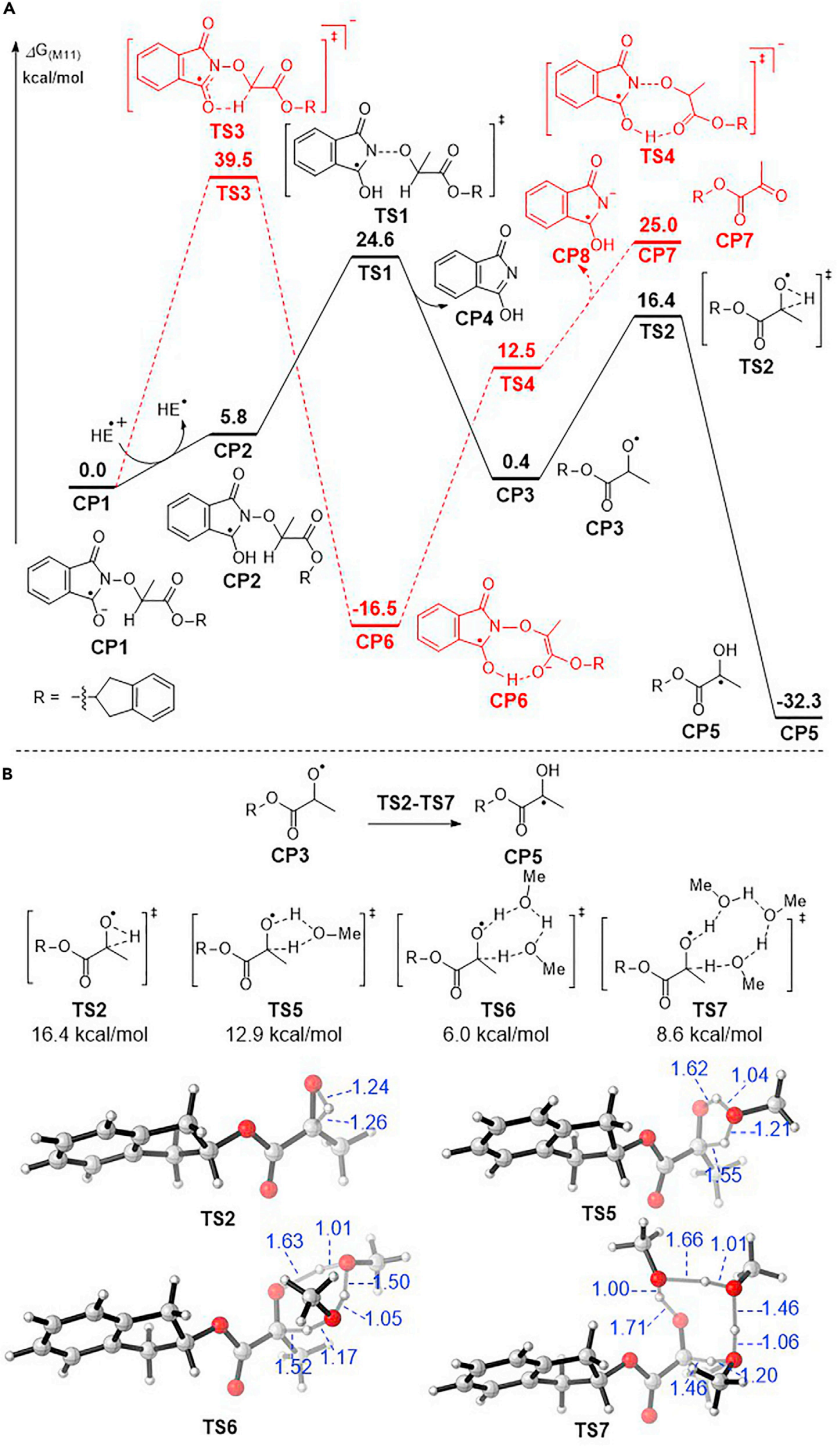
DFT Calculation of the 1,2-HAT of Alkoxyl Radicals (A) The free energy profile of two reaction pathways of N-alkoxylphthalimides. (B) The free energy profile of methanol-assisted 1,2-HAT of alkoxyl radicals.

We then performed density functional theory (DFT) calculations to investigate the free energy profiles of the alkoxyl radical generation (Scheme 9A). From computational studies, the N-alkoxylphthalimide **1** first undergoes single electron reduction to generate the radical anion **CP1**. After protonation by the Hantzsch ester radical cation, the alkyl radical intermediate **CP2** was formed with 5.8 kcal/mol endothermically (black line) (Azizi et al., 2015, McSkimming and Colbran, 2013, Zheng and You, 2012, Turovska et al., 2008, Zhu et al., 1999). The N-O bond was then homolytically cleaved to form the alkoxyl radical **CP3** via the transition state **TS1** with an activation energy of 18.8 kcal/mol. Alternatively, the radical anion **CP1** may form the intermediate **CP6** via the transition state **TS3** with 39.5 kcal/mol of activation energy, and the following redox fragmentation generates the ketoester **CP7** (red line) (Qi and Chen, 2016). However, the prohibitively high 39.5 kcal/mol of activation energy of **TS3** excludes it as the major reaction pathway, which is consistent with the experimental observation of the preferential formation of alkoxyl radicals in different substrates.

The methanol-assisted 1,2-HAT of alkoxyl radicals was next investigated by computational studies (Scheme 9B). The direct hydrogen atom transfer to form the ketyl radical **CP5** was calculated to have 16.4 kcal/mol of activation energy in **TS2**. In contrast, the involvement of methanol dramatically affects the energy diagram with hydrogen bonds. With one molecule of methanol participation, a 3.5-kcal/mol decrease of activation energy can be obtained in **TS5**. Significantly, two methanol molecules reduce the activation energy by 10.4 kcal/mol to merely 6.0 kcal/mol in **TS6** with multiple hydrogen bond formation, and three methanol molecules can lower the activation energy by 7.8 kcal/mol in **TS7**. From the computational studies mentioned above, the methanol facilitates the alkoxyl radical **CP3** rearrangement to ketyl radical **CP5** with hydrogen bonds, and an up to 10.4 kcal/mol decrease of activation energy can be obtained with the methanol assistance (the involvement of one methanol and one water molecule decreased the activation barrier to 6.9 kcal/mol; see Scheme S14. The α-C-H functionalization product distribution in different solvents is not only determined by the 1,2-HAT reactivity, but also affected by the alkoxyl radical generation) (see Tables S4, S5, S6, and S7).

**Scheme 10:**
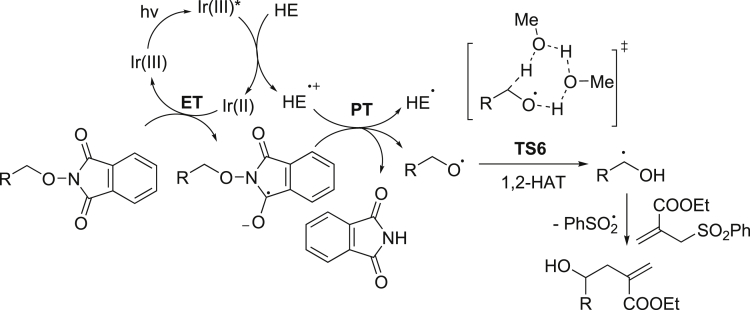
Mechanistic Proposals

With mechanistic experiments and DFT calculations mentioned above, we propose the reaction is initiated from the reductive quenching of the photoexcited Ir(III)∗ to Ir(II) by Hantzsch ester, and Ir(II) subsequently reduces the N-alkoxylphthalimides to the radical anion (Scheme 10). The radical anion undergoes proton transfer with Hantzsch ester radical cation and subsequent N-O bond cleavage to form the alkoxyl radical (Fukuzumi et al., 1983, Lackner et al., 2015, Pratsch et al., 2015, Taylor et al., 2018, and see Schemes S4–S10 for details). Two methanol molecules then assist the 1,2-HAT reaction with hydrogen bonds at the α-carbonyl, α-cyano, α-trifluoromethyl, or benzylic C(sp^3^)-H bonds to form ketyl radicals for new C-C bond formations (Poutsma, 2007, Nechab et al., 2014).

### Conclusions

In conclusion, we have developed the first regioselective α-C(sp^3^)-H functionalization enabled by 1,2-HAT of alkoxyl radicals using photoredox catalysis. The 1,2-HAT of alkoxyl radicals was confirmed by various mechanistic investigations including EPR studies and was useful for the new C-C bond formation of α-carbonyl, α-cyano, α-trifluoromethyl, and benzylic N-alkoxylphthalimides. The computational studies indicate the assistance of protic solvents significantly facilities the 1,2-HAT reaction of alkoxyl radicals for new C-C bond formations. Further investigations are ongoing to explore this new 1,2-HAT reactivity of alkoxyl radicals.

### Limitations of the Study

The 1,2-HAT pathway is not always favored for alkoxyl radicals; other alkoxyl radical reaction pathways may complete over 1,2-HAT pathway in different substrates. The existence of the carbonyl intermediate cannot be completely excluded; however, it is not the main reaction pathway from the performed computational and experimental studies.

## Methods

All methods can be found in the accompanying Transparent Methods supplemental file.

## Supplemental Information

Document S1. Transparent Methods, Figures S1–S96, Schemes S1–S27, and Tables S1–S4, and S6

Table S5. B3LYP and M11 Absolute Calculation Energies, Enthalpies, and Free Energies, Related to Scheme 5

Table S7. B3LYP Geometries for All the Optimized Compounds and Transition States, Related to Scheme 5
