# Supplementary material for: Visible-Light-Induced Alkoxyl Radicals Enable α-C(sp3)-H Bond Allylation
Source: iScience. 2019 Dec 4;23(1):100755. doi: 10.1016/j.isci.2019.100755 (PMC6941871; doi:10.1016/j.isci.2019.100755)
Supplement: Document S1. Transparent Methods, Figures S1–S96, Schemes S1–S27, and Tables S1–S4, and S6 [file mmc1.pdf]

ISCI, Volume *23*

Supplemental Information

Visible-Light-Induced Alkoxyl Radicals Enable α-C(sp^3)-H Bond Allylation

Jing Zhang, Dan Liu, Song Liu, Yuanyuan Ge, Yu Lan, and Yiyun Chen

I. Spectra of New Compounds

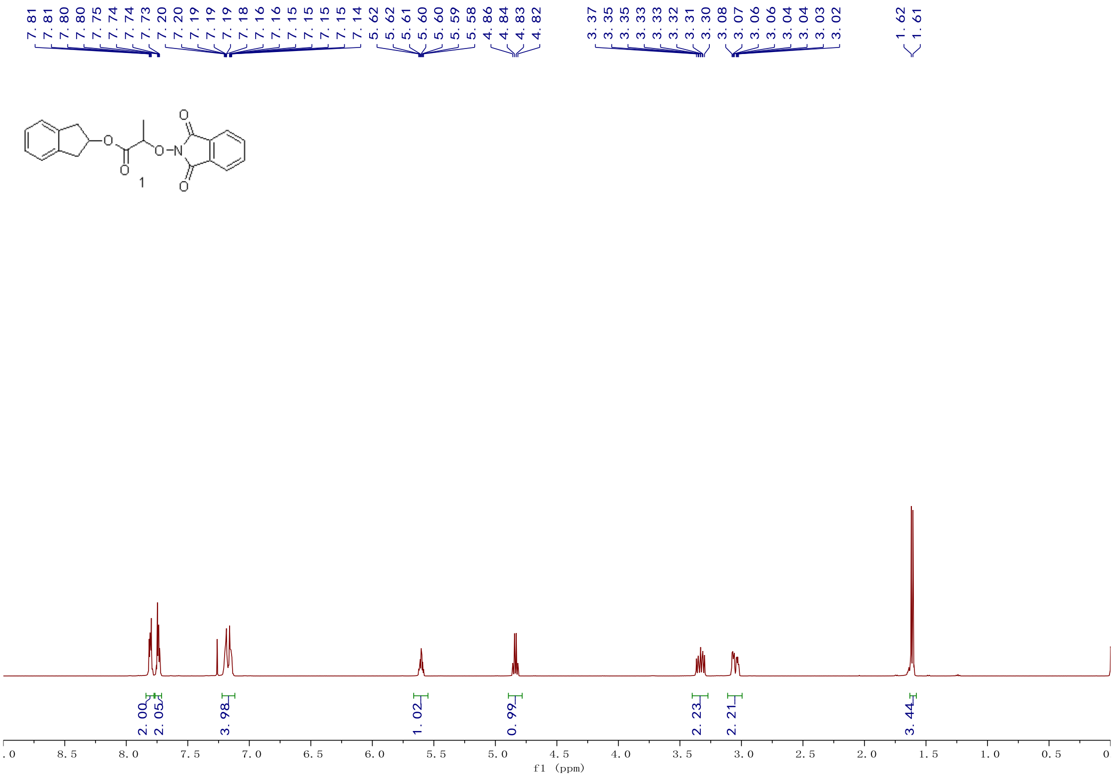

Figure S1. ^{1}H NMR (500 MHz, CDCl3) spectrum of compound 1, related to Scheme 2.

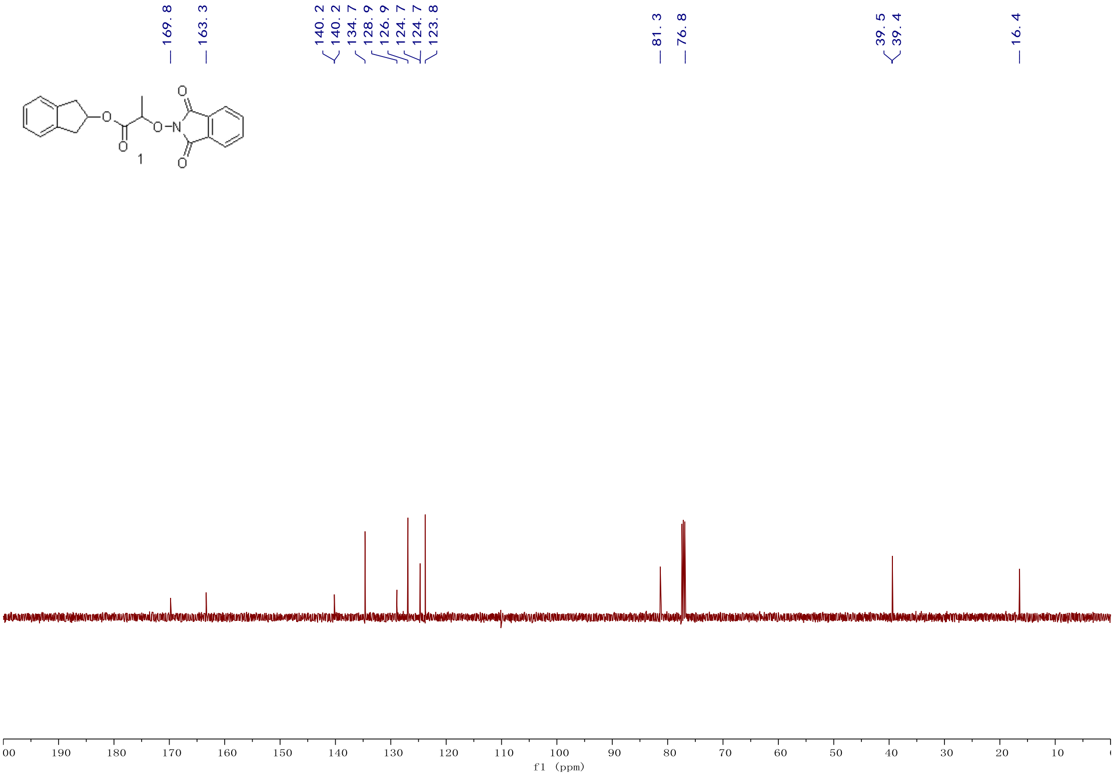

Figure S2. ^{13}C NMR (126 MHz, CDCl3) spectrum of compound 1, related to Scheme 2.

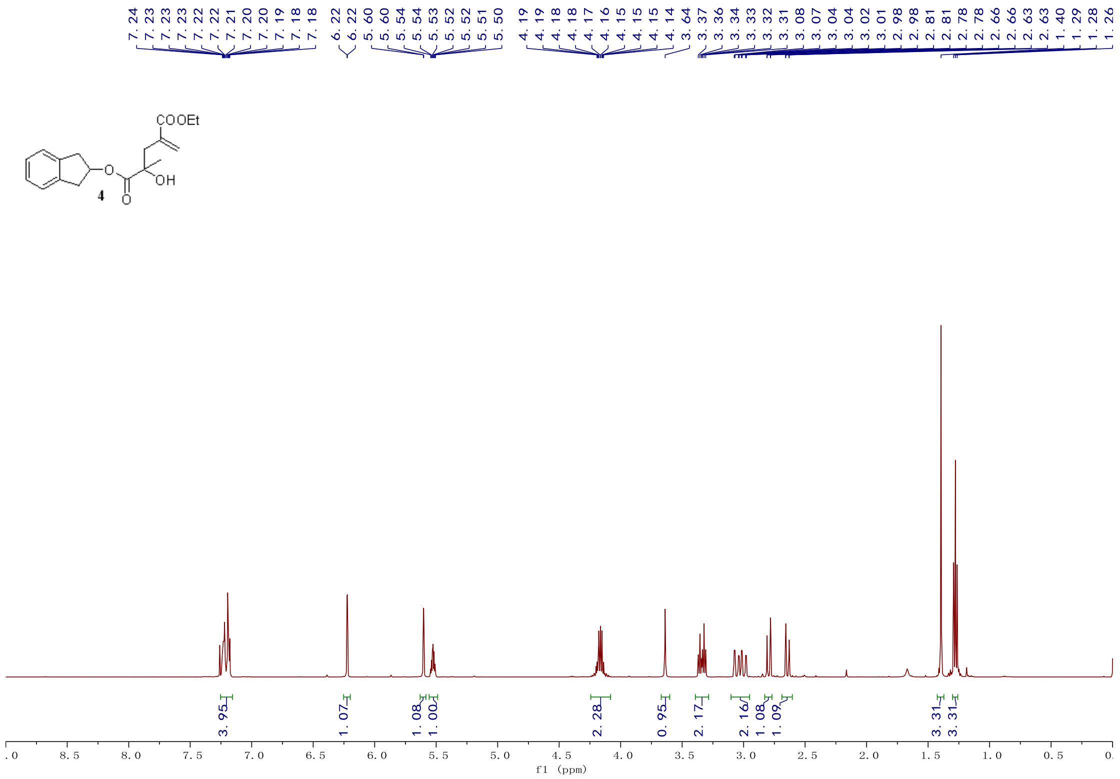

Figure S3. ^{1}H NMR (500 MHz, CDCl3) spectrum of compound 4, related to Scheme 2.

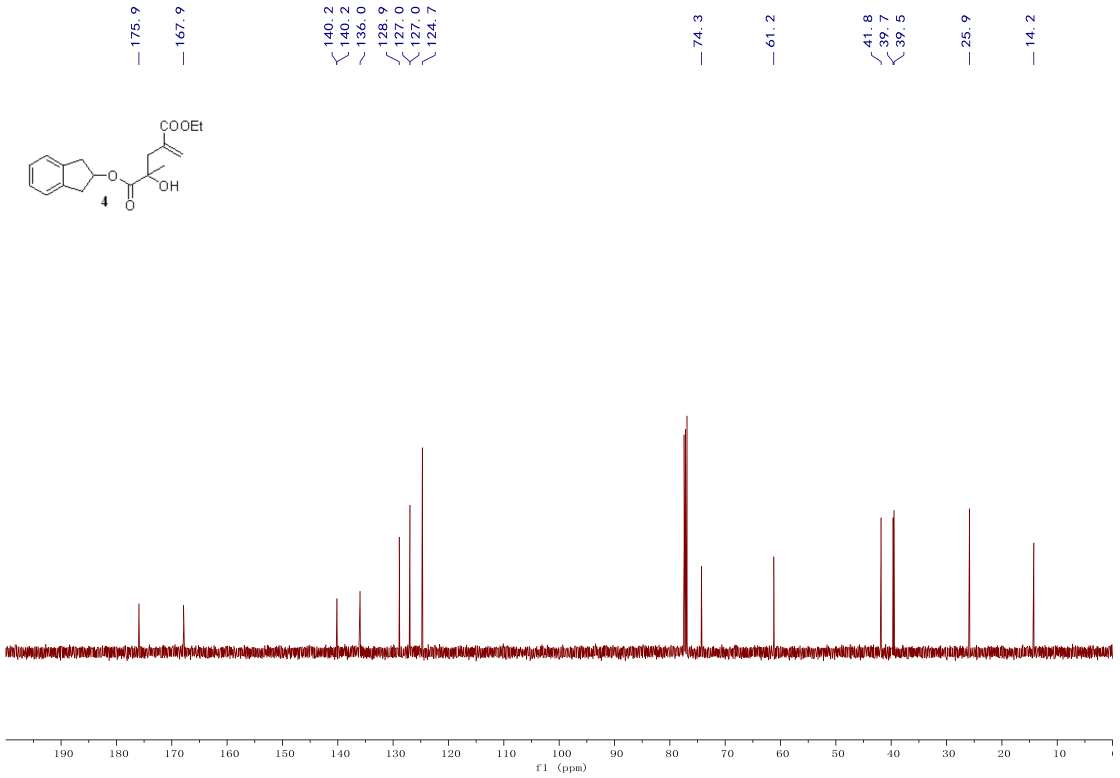

Figure S4. ^{13}C NMR (126 MHz, CDCl3) spectrum of compound 4, related to Scheme 2.

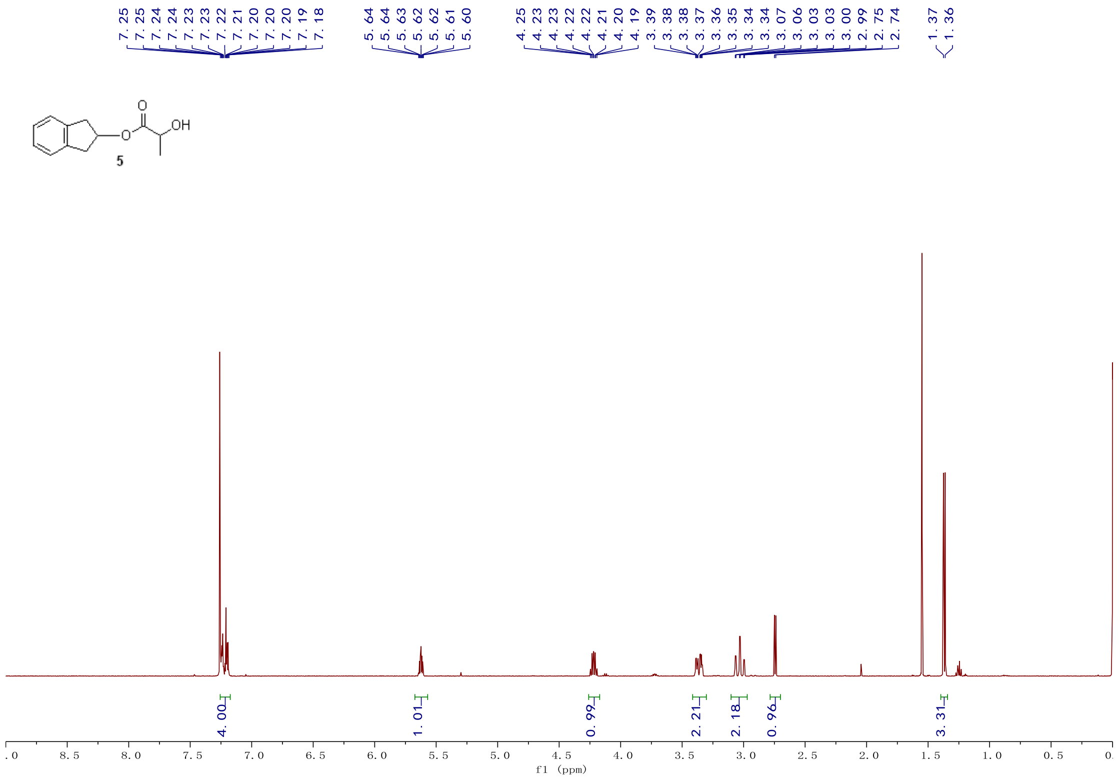

Figure S5. ^{1}H NMR (500 MHz, CDCl3) spectrum of compound 5, related to Scheme 2.

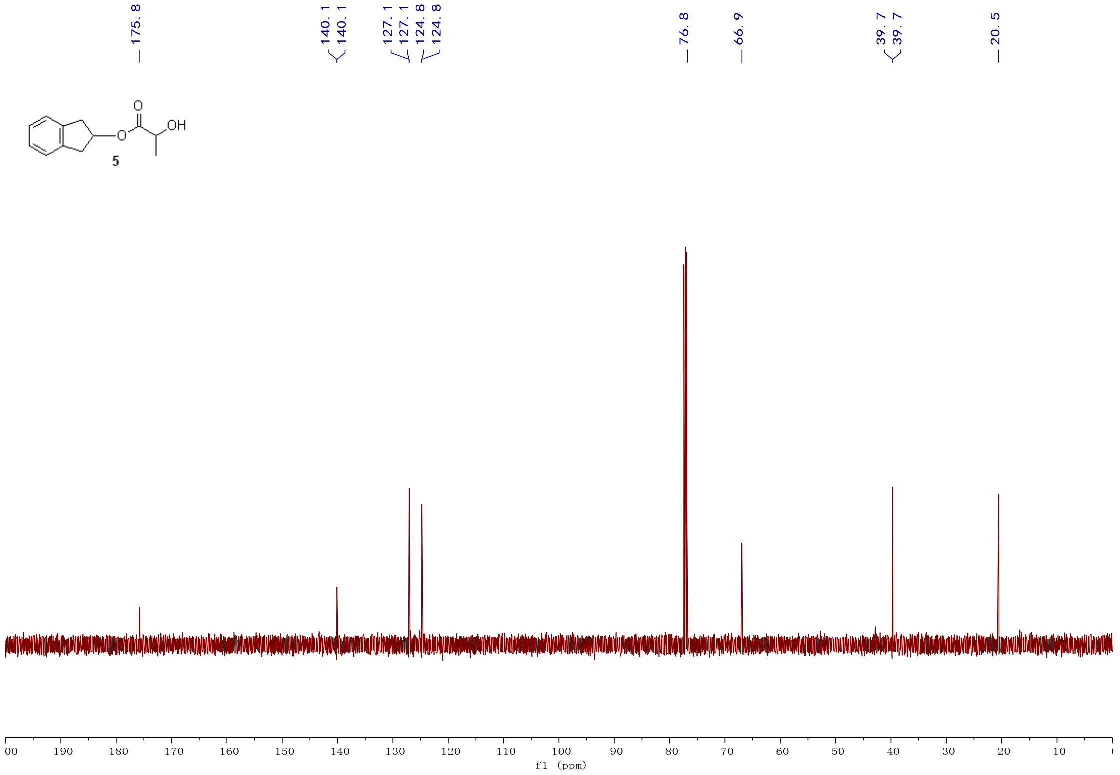

Figure S6. ^{13}C NMR (126 MHz, CDCl3) spectrum of compound 5, related to Scheme 2.

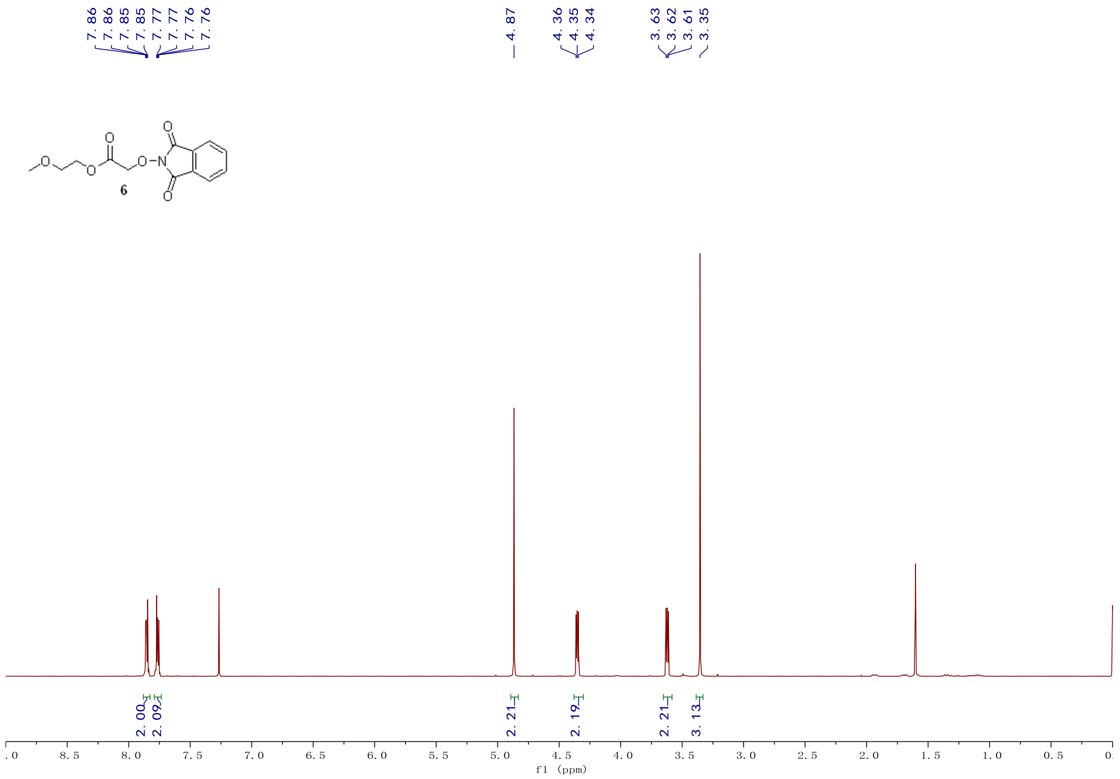

Figure S7. [1]**H NMR (500 MHz, CDCl3) spectrum of compound 6, related to Scheme 2.**

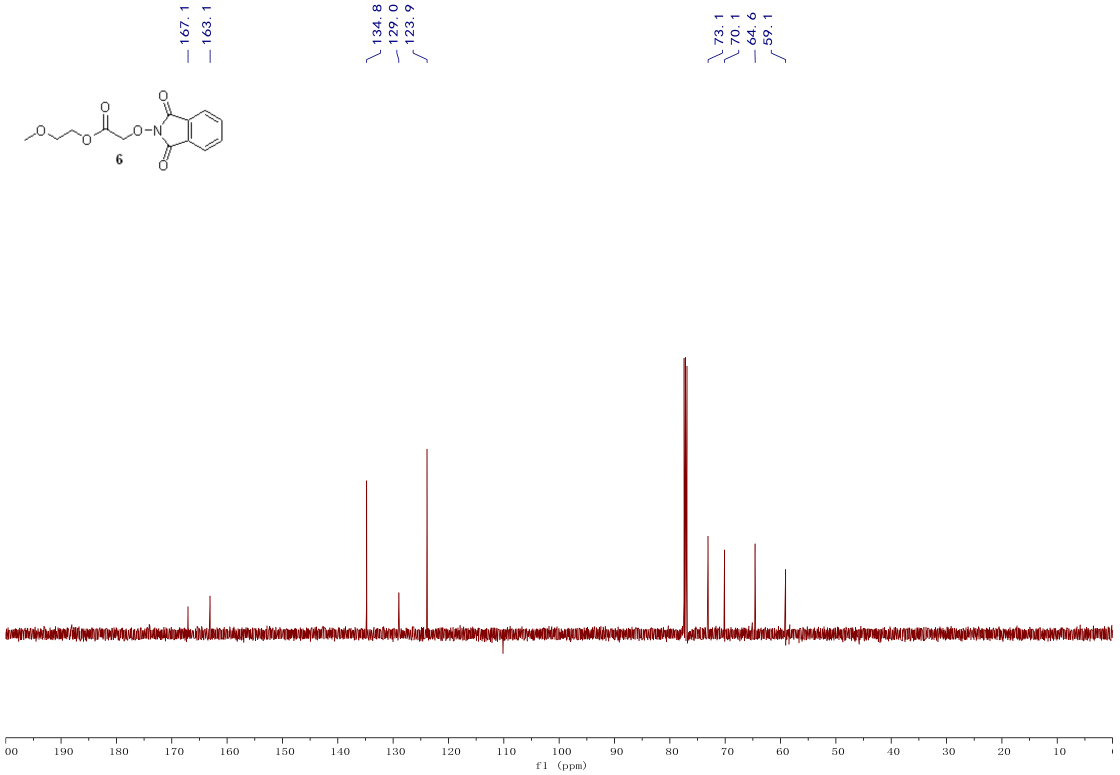

Figure S8. [13]**C NMR (126 MHz, CDCl3) spectrum of compound 6, related to Scheme 2.**

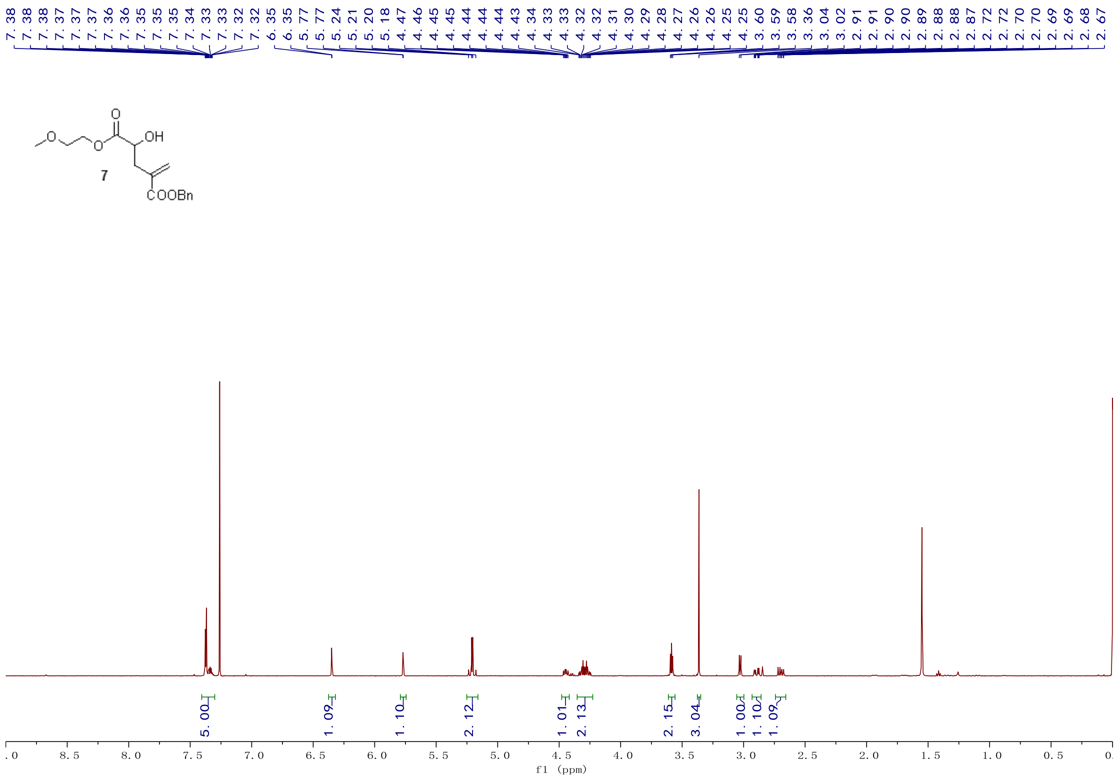

Figure S9. ^{1}H NMR (500 MHz, CDCl3) spectrum of compound 7, related to Scheme 2.

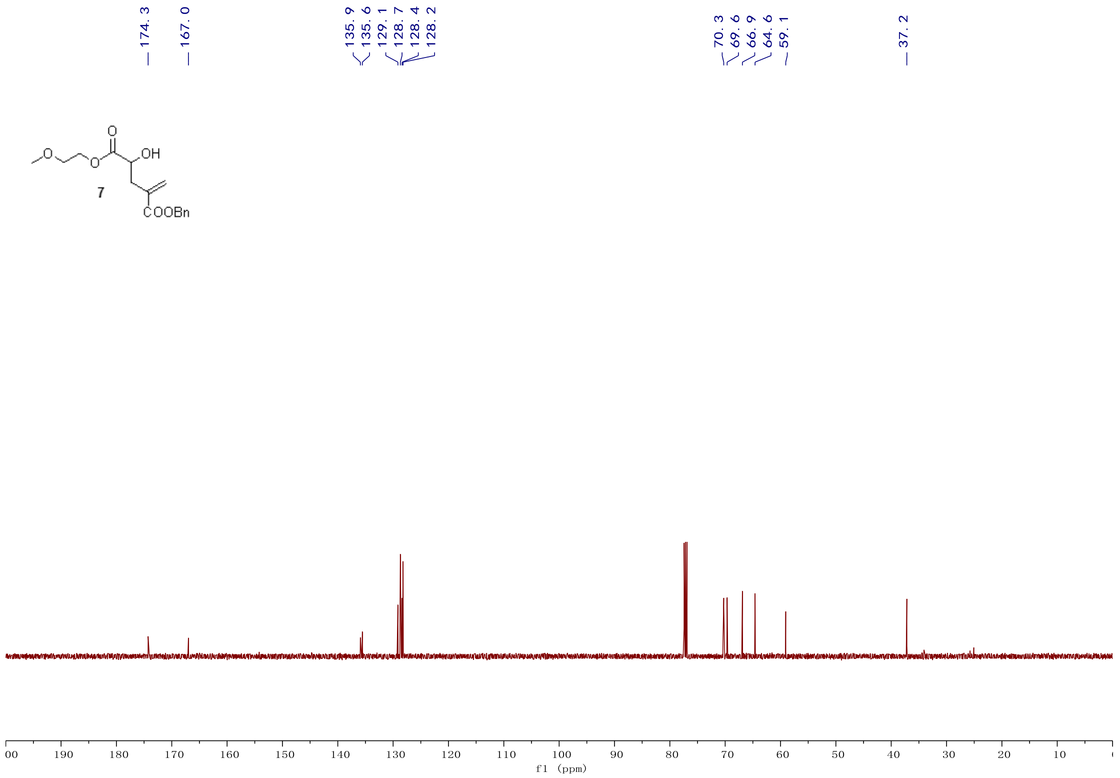

Figure S10. ^{13}C NMR (126 MHz, CDCl3) spectrum of compound 7, related to Scheme 2.

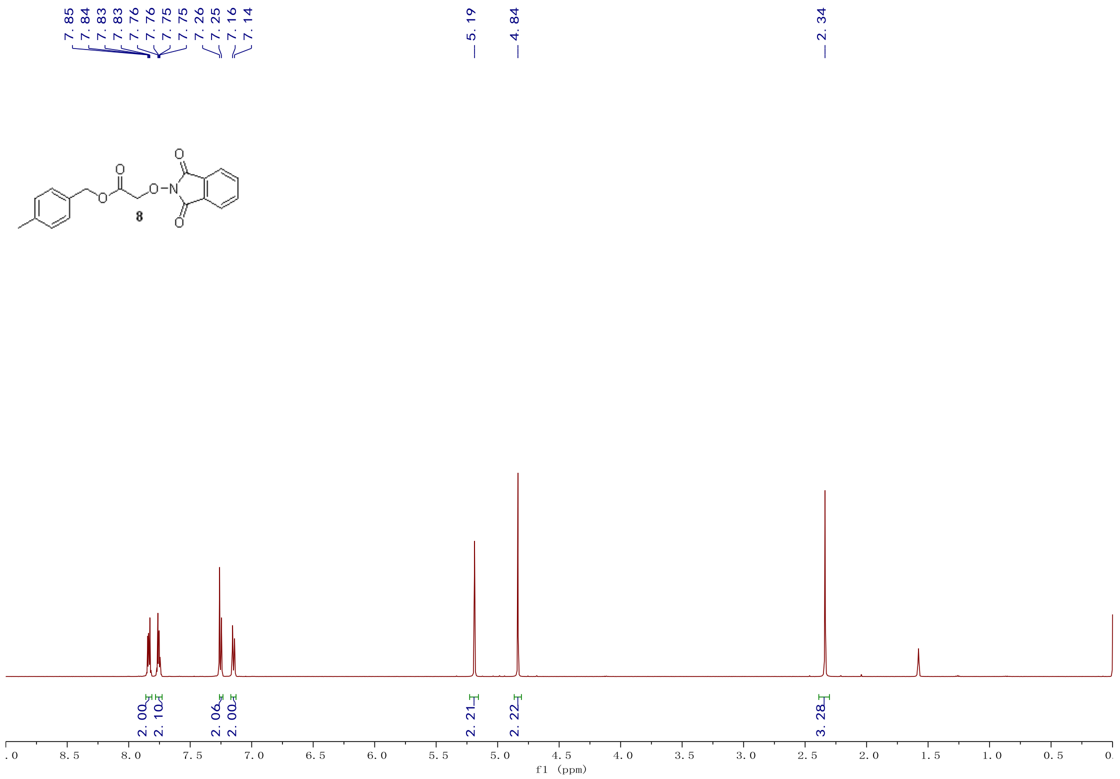

Figure S11. [1]**H NMR (500 MHz, CDCl3) spectrum of compound 8, related to Scheme 2.**

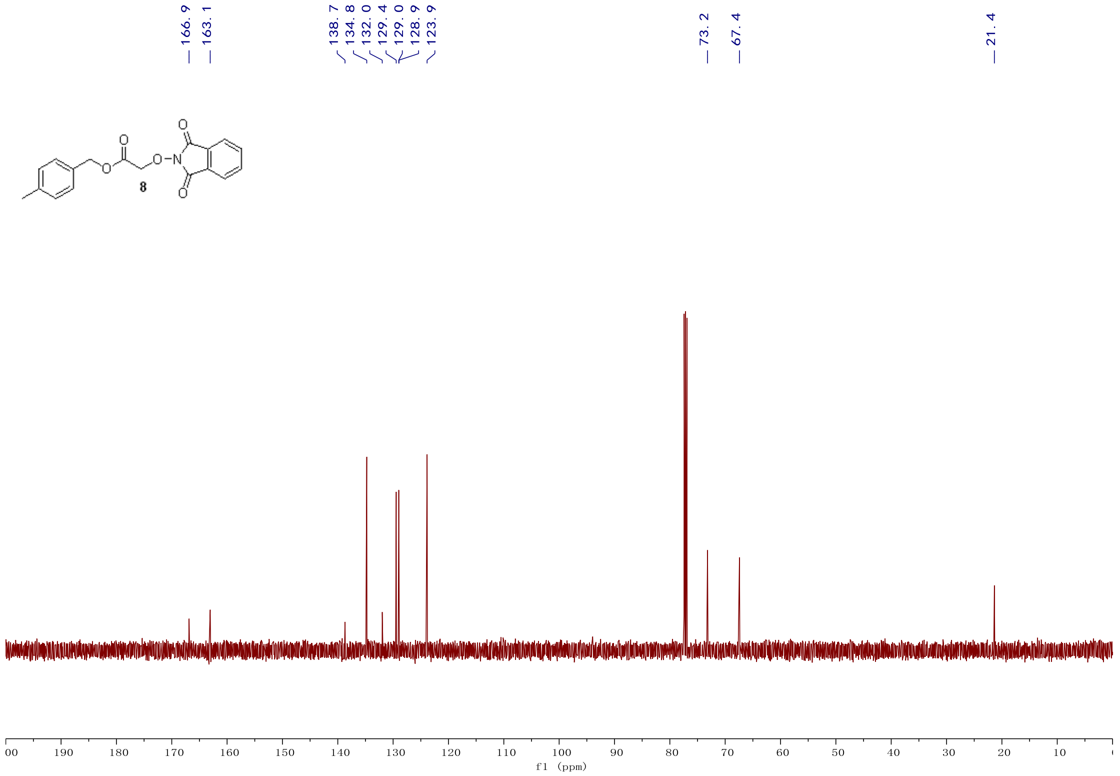

Figure S12. [13]**C NMR (126 MHz, CDCl3) spectrum of compound 8, related to Scheme 2.**

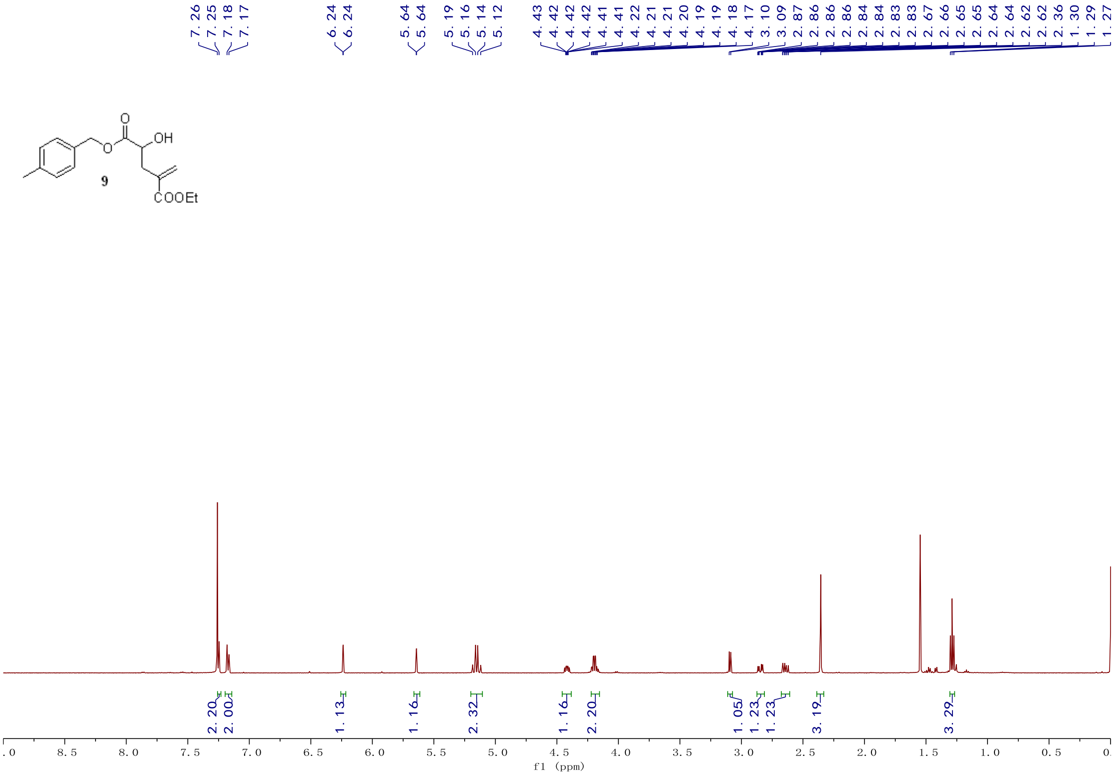

Figure S13. ^{1}H NMR (500 MHz, CDCl3) spectrum of compound 9, related to Scheme 2.

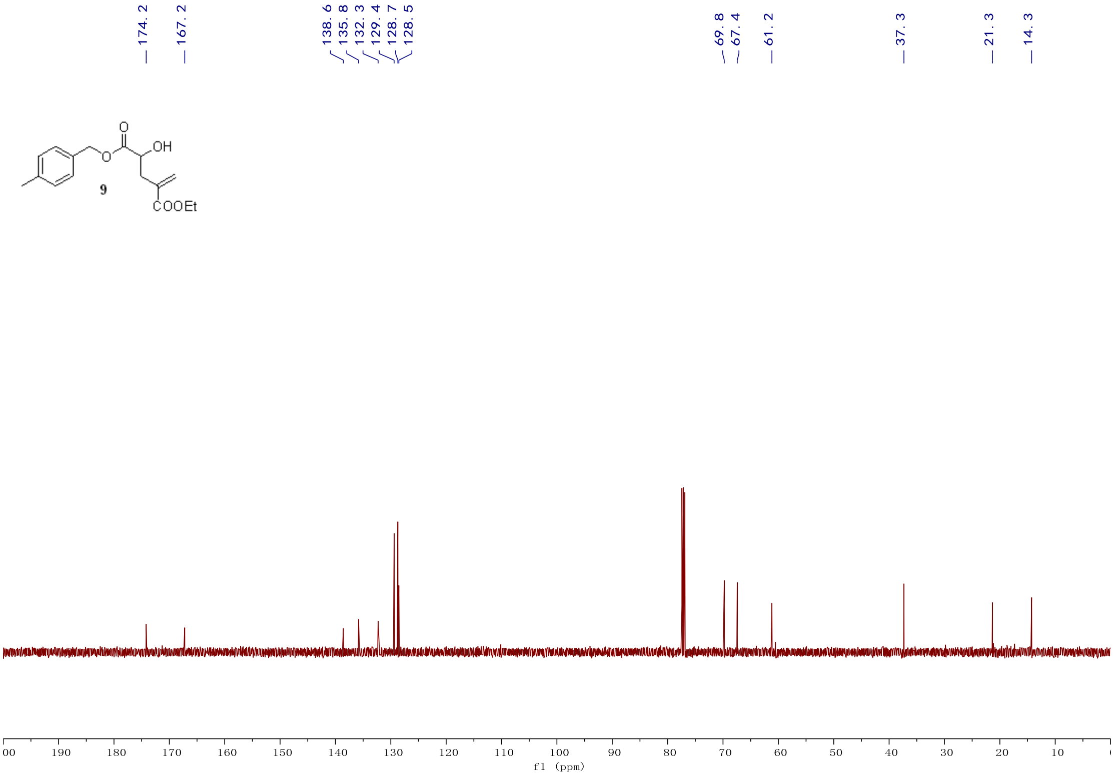

Figure S14. ^{13}C NMR (126 MHz, CDCl3) spectrum of compound 9, related to Scheme 2.

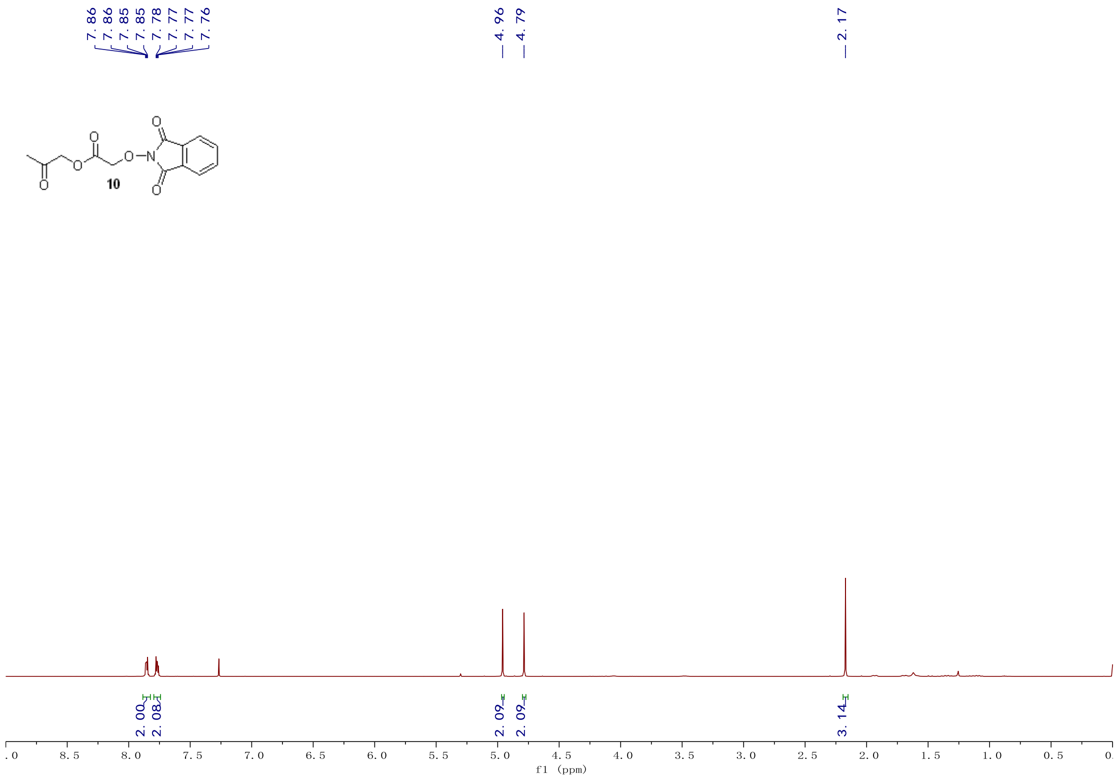

Figure S15. ^{1}H **NMR (500 MHz, CDCl3) spectrum of compound 10, related to Scheme 2.**

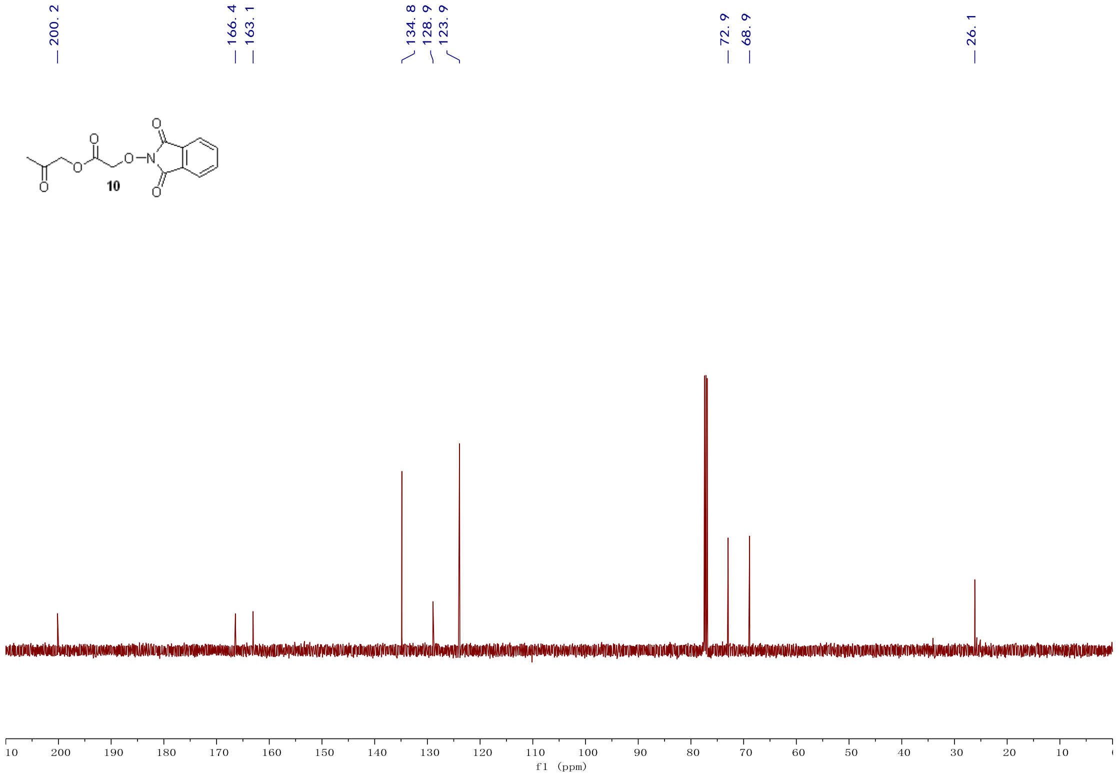

Figure S16. ^{13}C **NMR (126 MHz, CDCl3) spectrum of compound 10, related to Scheme 2.**

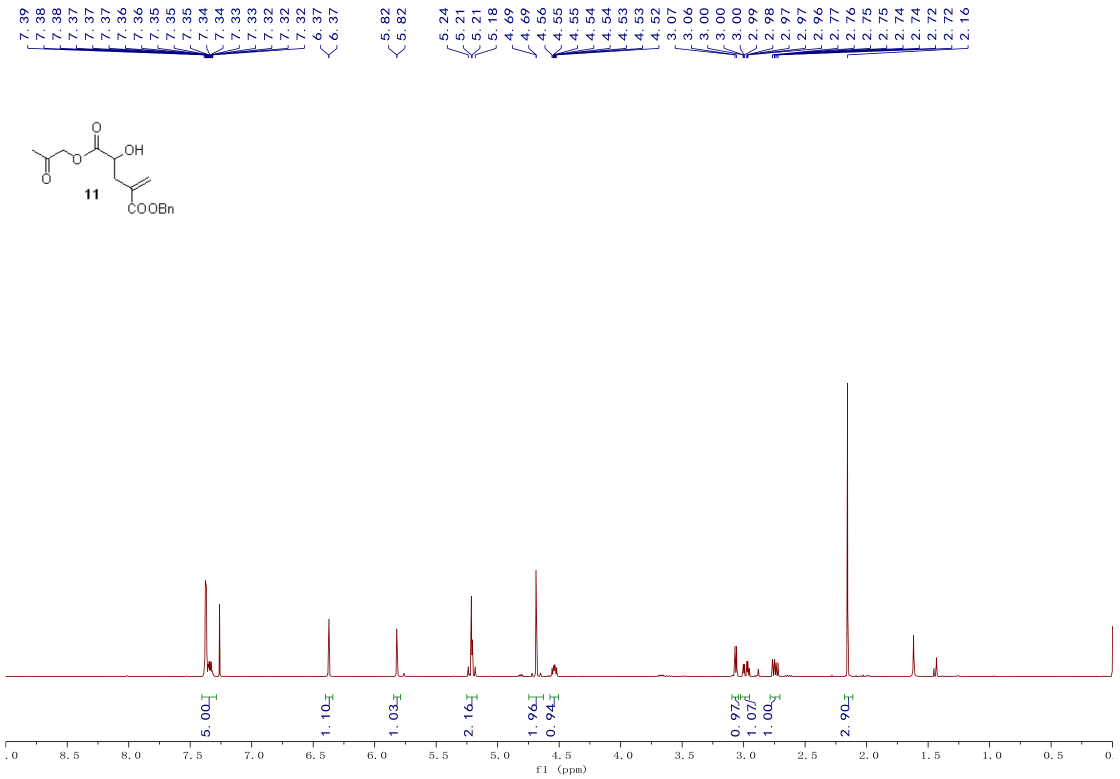

Figure S17. ^{1}H NMR (500 MHz, CDCl3) spectrum of compound 11, related to Scheme 2.

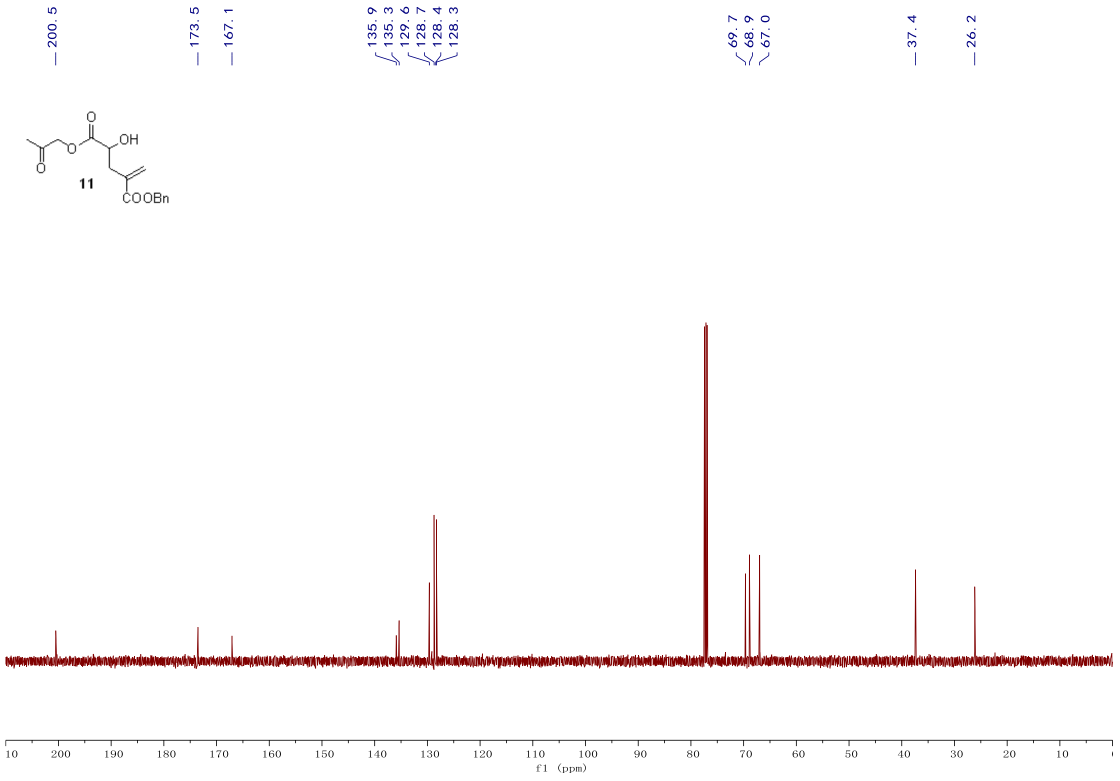

Figure S18. ^{13}C NMR (126 MHz, CDCl3) spectrum of compound 11, related to Scheme 2.

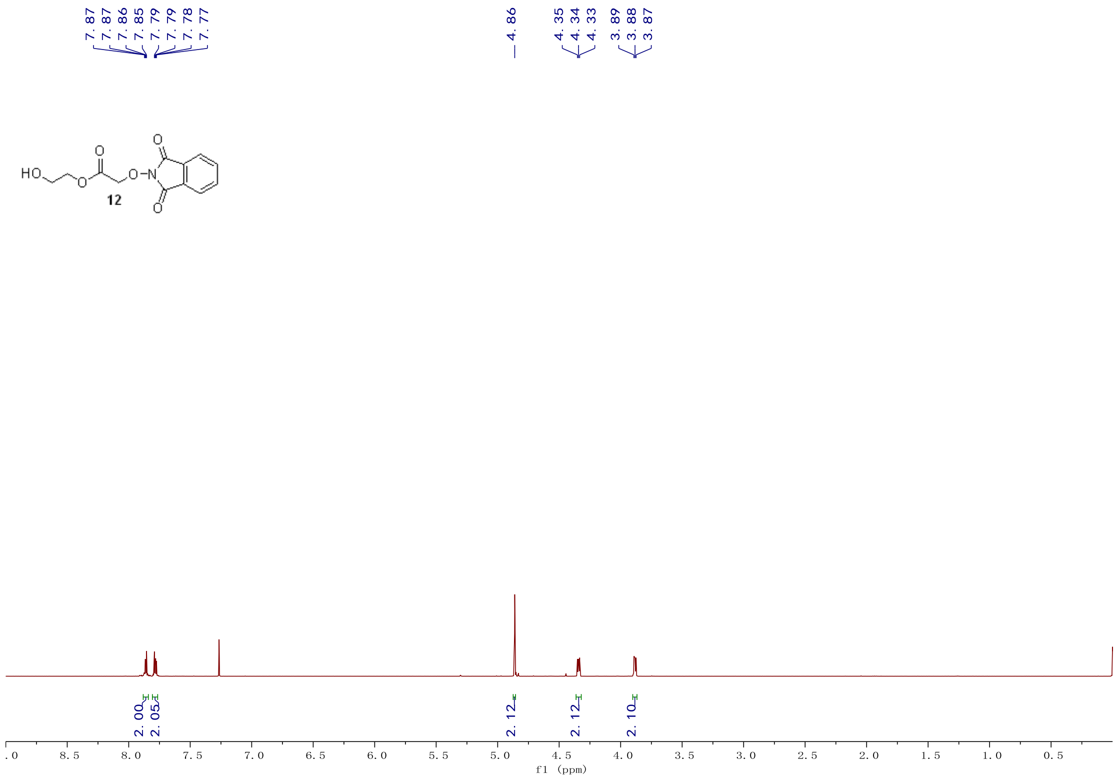

Figure S19. ¹H NMR (500 MHz, CDCl3) spectrum of compound 12, related to Scheme 2.

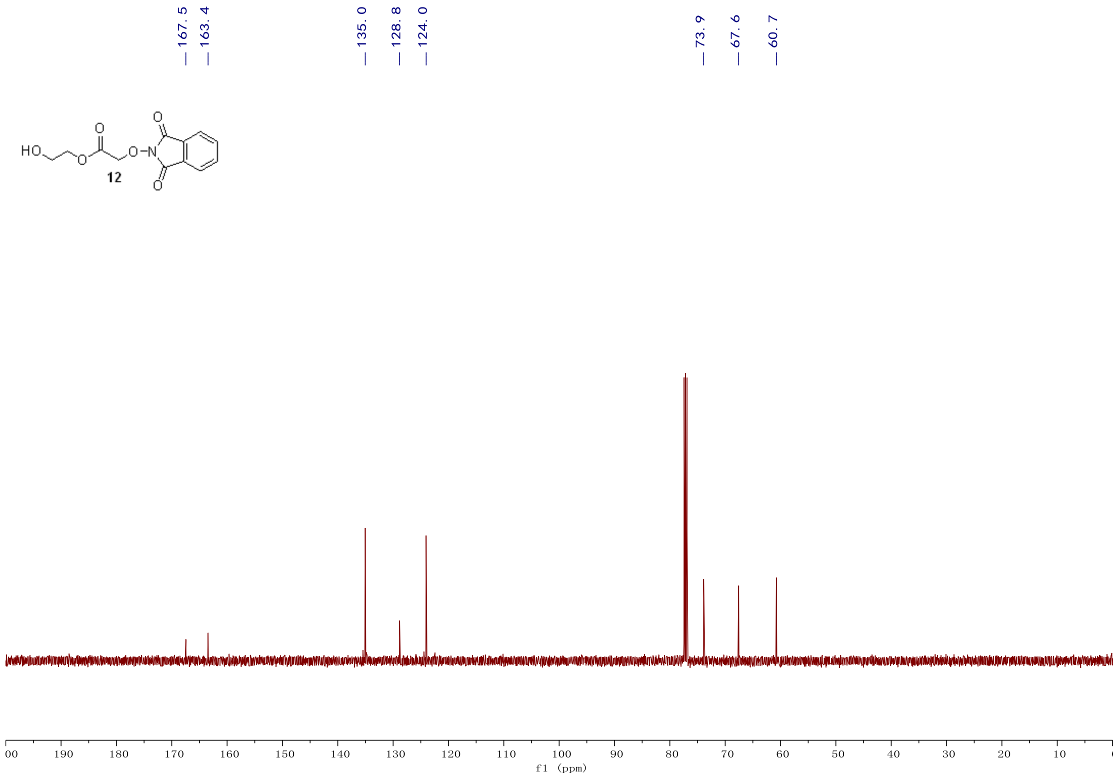

Figure S20. ¹³C NMR (126 MHz, CDCl3) spectrum of compound 12, related to Scheme 2.

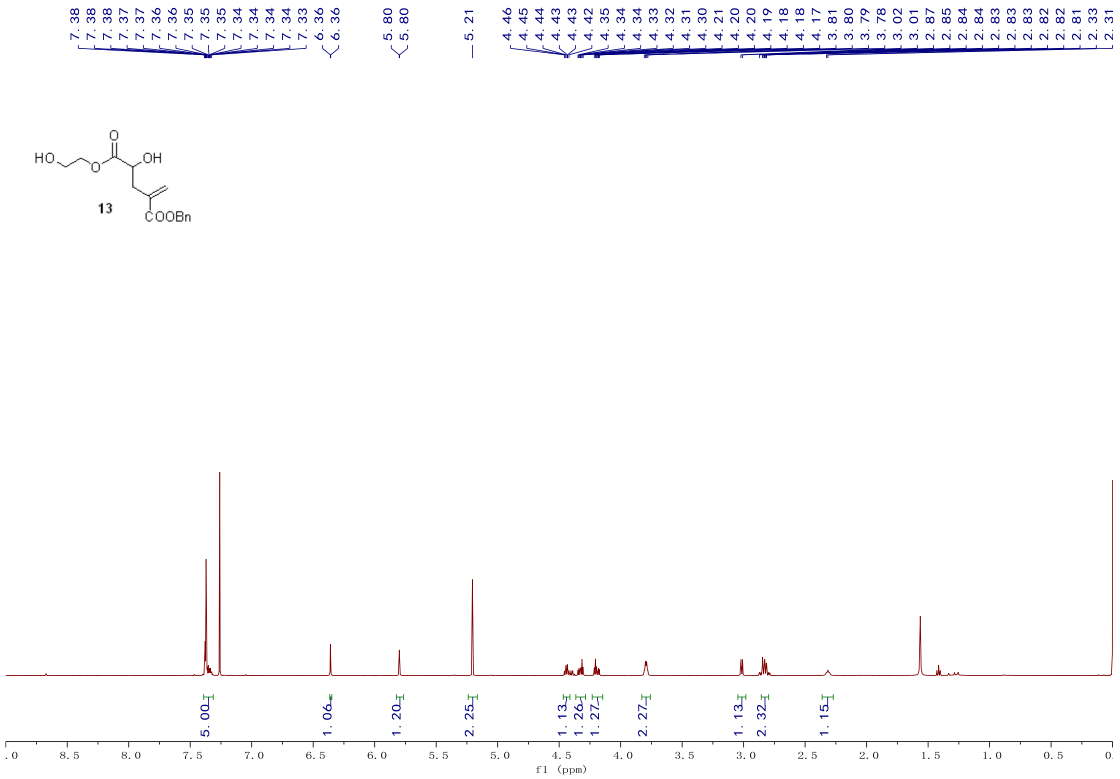

Figure S21. ^{1}H **NMR (500 MHz, CDCl3) spectrum of compound 13, related to Scheme 2.**

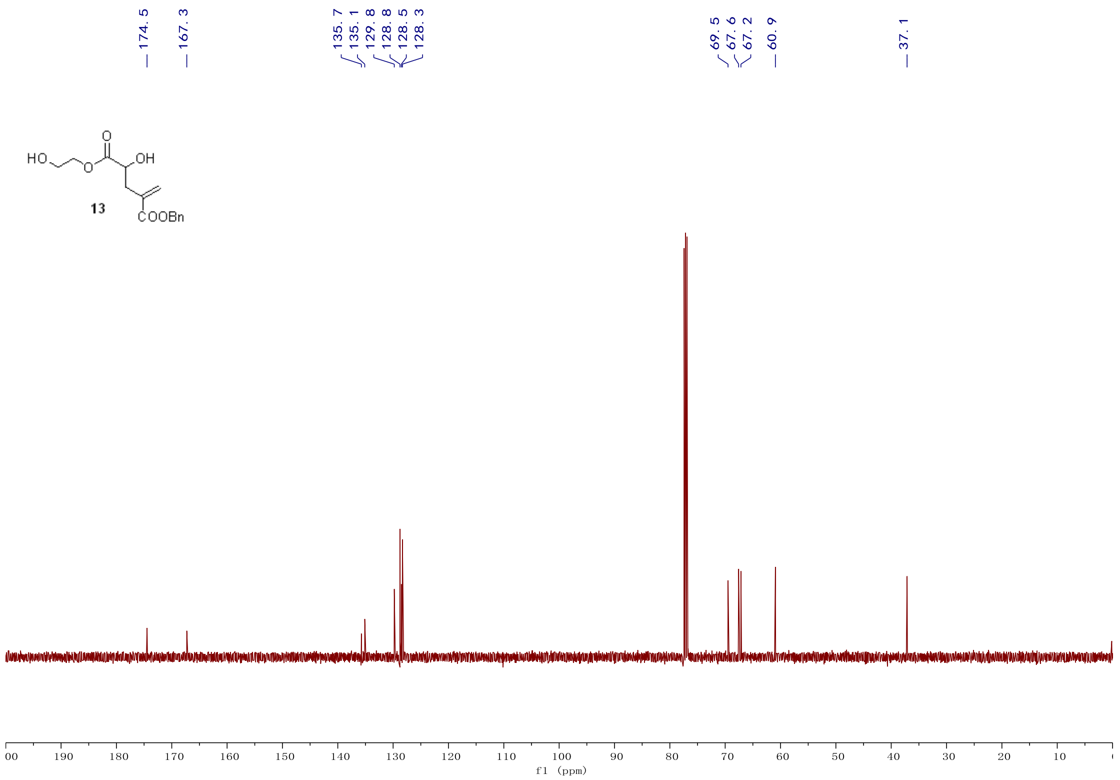

Figure S22. ^{13}C **NMR (126 MHz, CDCl3) spectrum of compound 13, related to Scheme 2.**

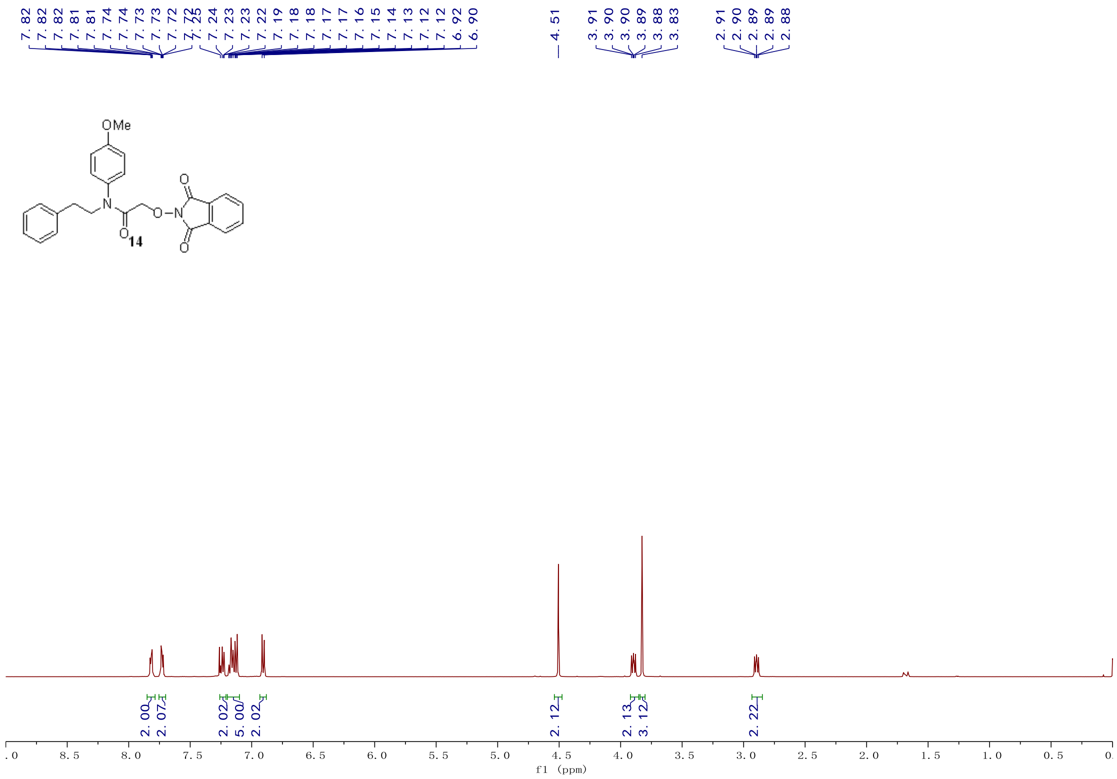

Figure S23. [1]**H NMR (500 MHz, CDCl3) spectrum of compound 14, related to Scheme 2.**

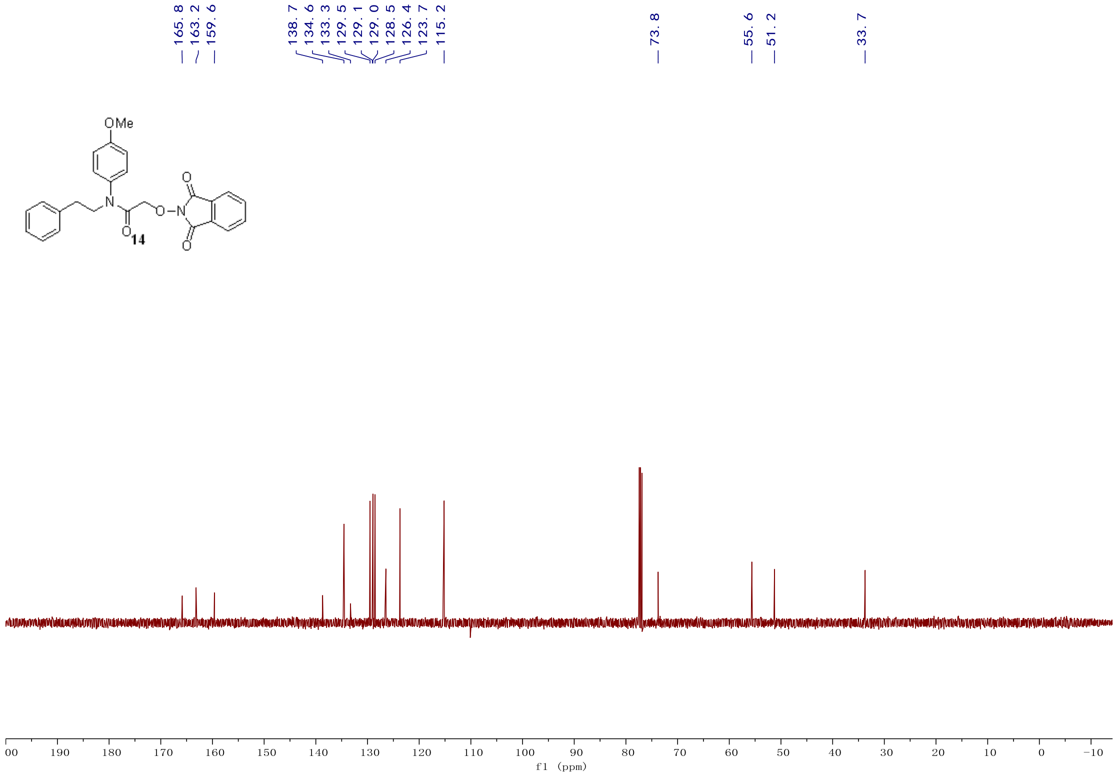

Figure S24. [13]**C NMR (126 MHz, CDCl3) spectrum of compound 14, related to Scheme 2.**

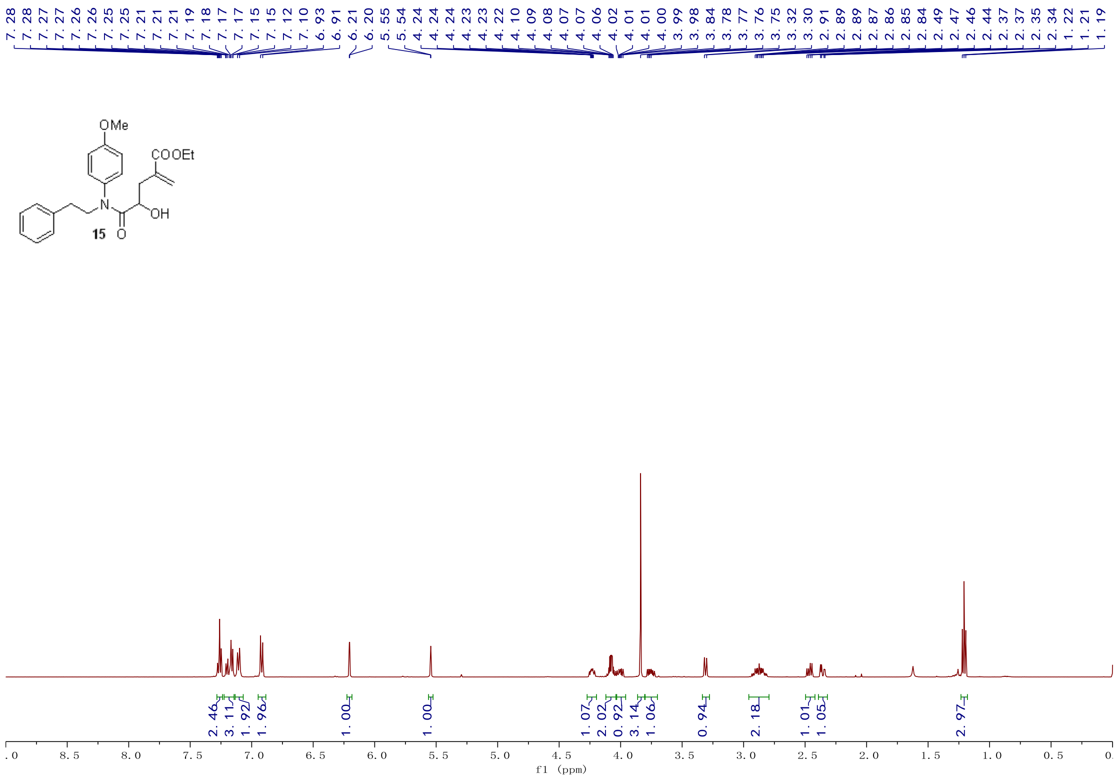

Figure S25. ^{1}H NMR (500 MHz, CDCl3) spectrum of compound 15, related to Scheme 2.

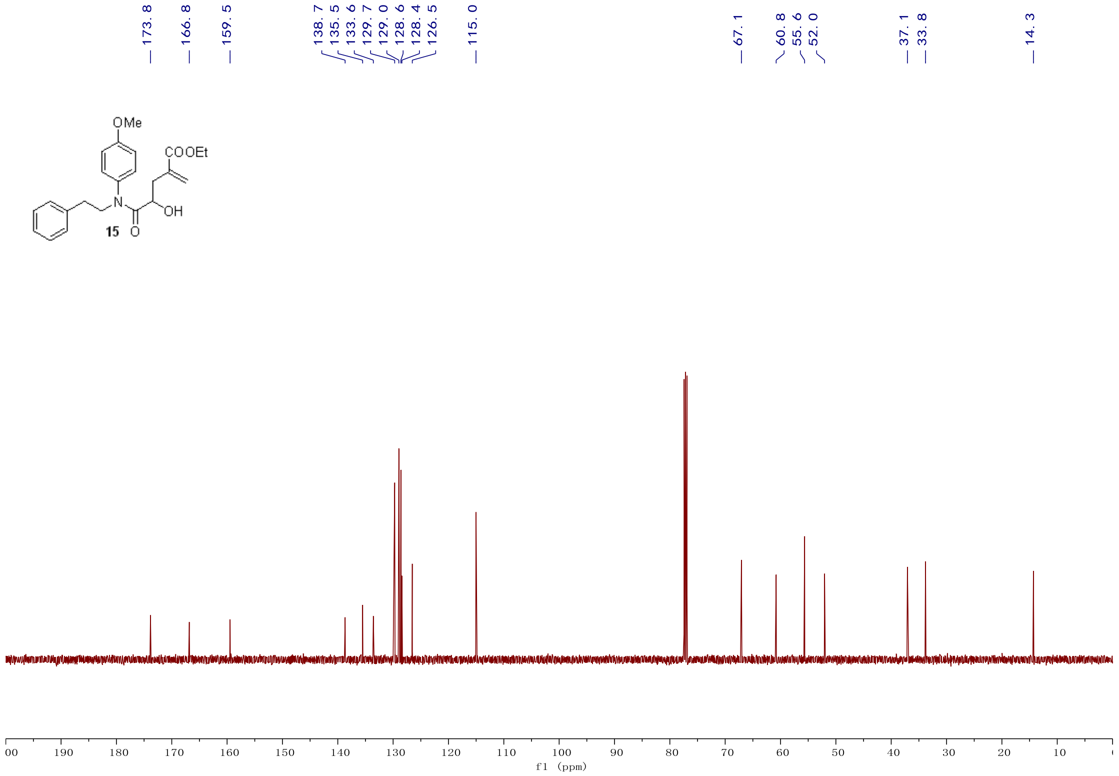

Figure S26. ^{13}C NMR (126 MHz, CDCl3) spectrum of compound 15, related to Scheme 2.

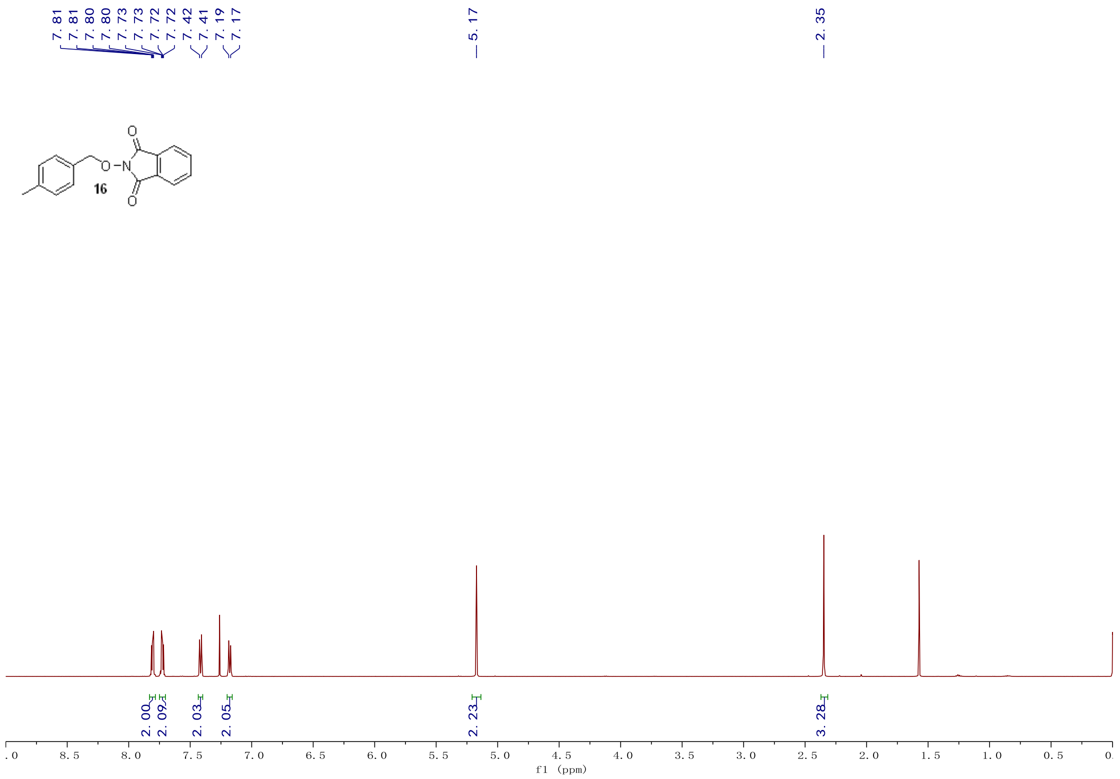

Figure S27. ^{1}**H NMR (500 MHz, CDCl3) spectrum of compound 16, related to Scheme 2.**

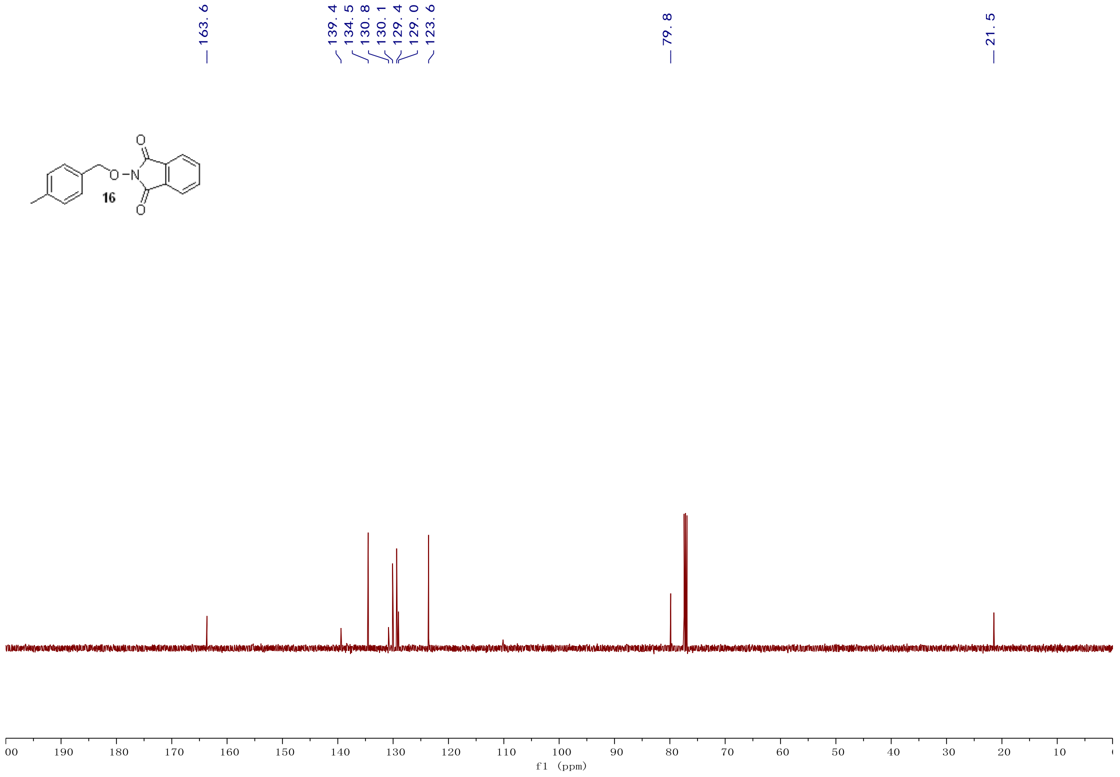

Figure S28. ^{13}**C NMR (126 MHz, CDCl3) spectrum of compound 16, related to Scheme 2.**

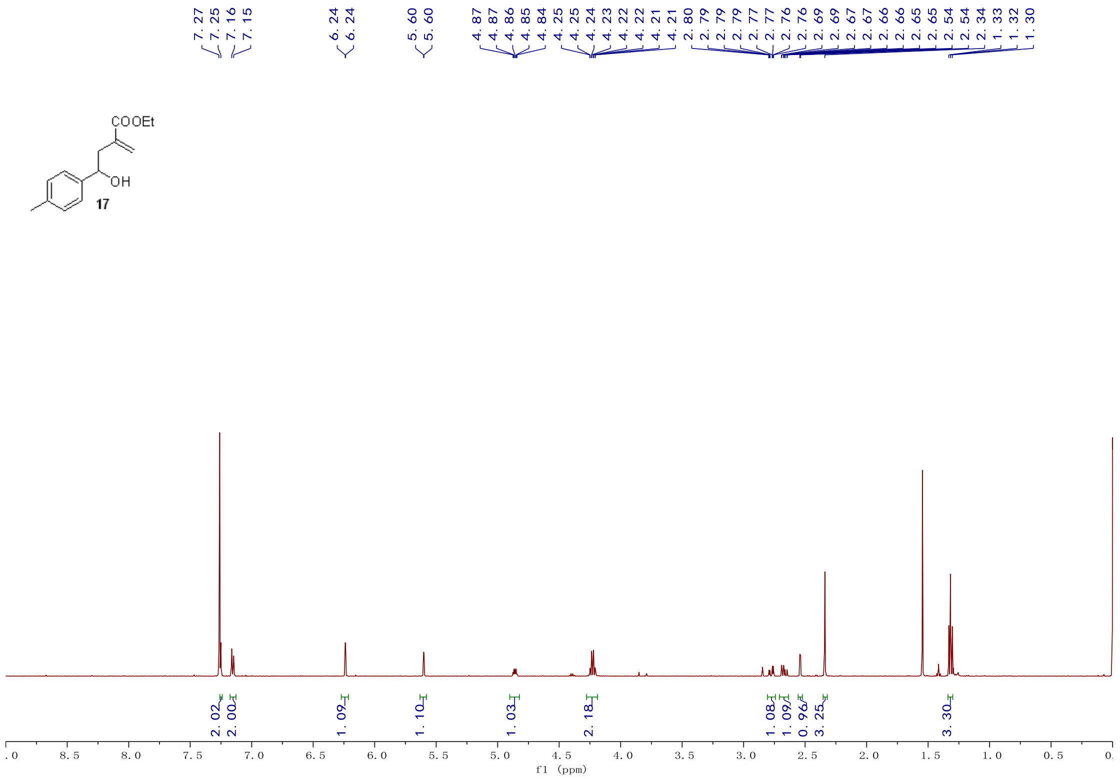

Figure S29. ^{1}H NMR (500 MHz, CDCl3) spectrum of compound 17, related to Scheme 2.

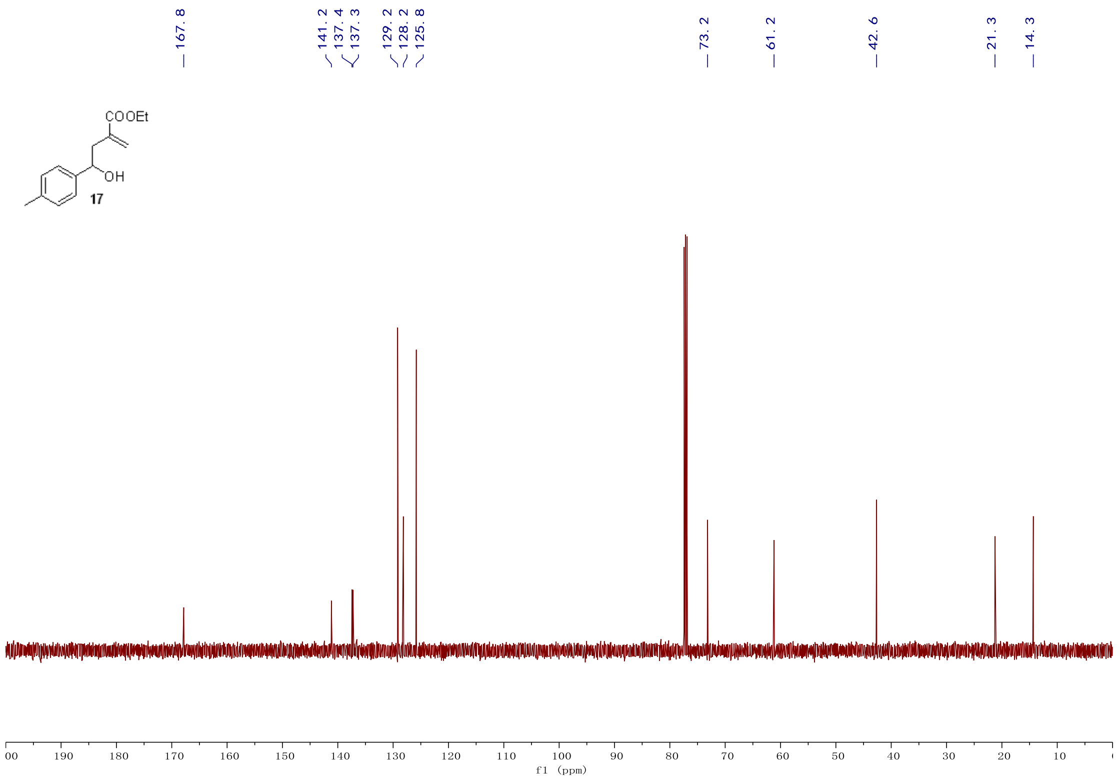

Figure S30. ^{13}C NMR (126 MHz, CDCl3) spectrum of compound 17, related to Scheme 2.

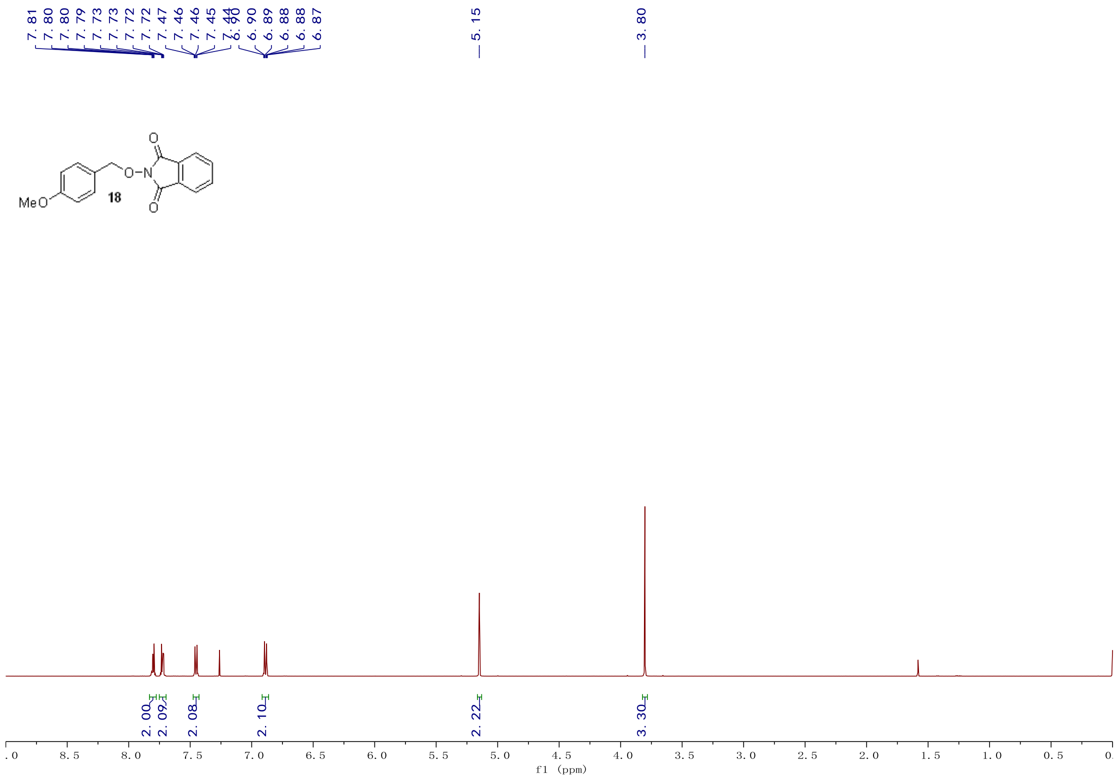

Figure S31. ^{1}H NMR (500 MHz, CDCl3) spectrum of compound 18, related to Scheme 2.

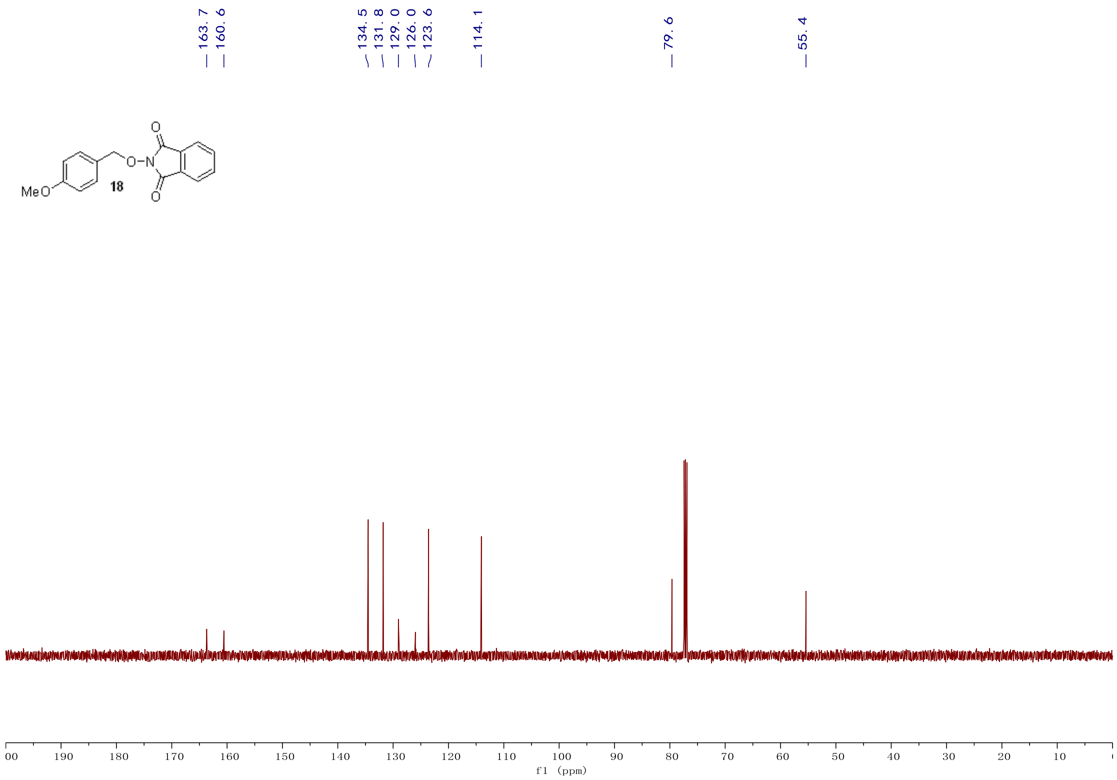

Figure S32. ^{13}C NMR (126 MHz, CDCl3) spectrum of compound 18, related to Scheme 2.

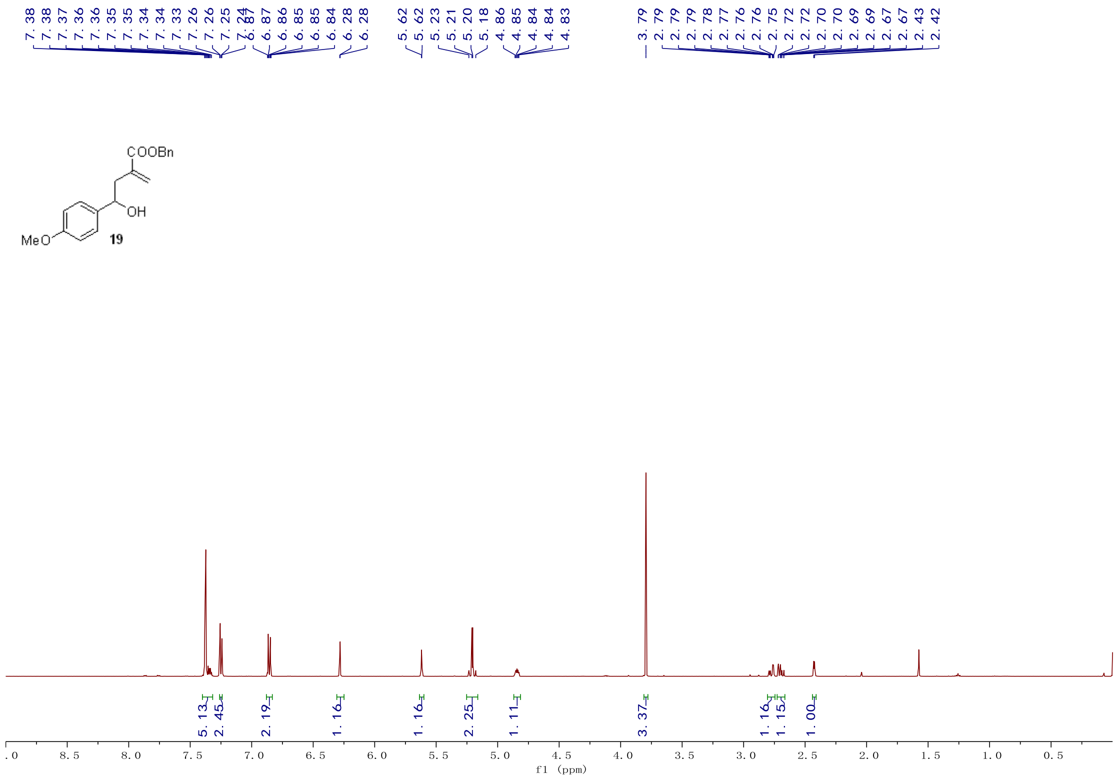

Figure S33. ^{1}**H NMR (500 MHz, CDCl3) spectrum of compound 19, related to Scheme 2.**

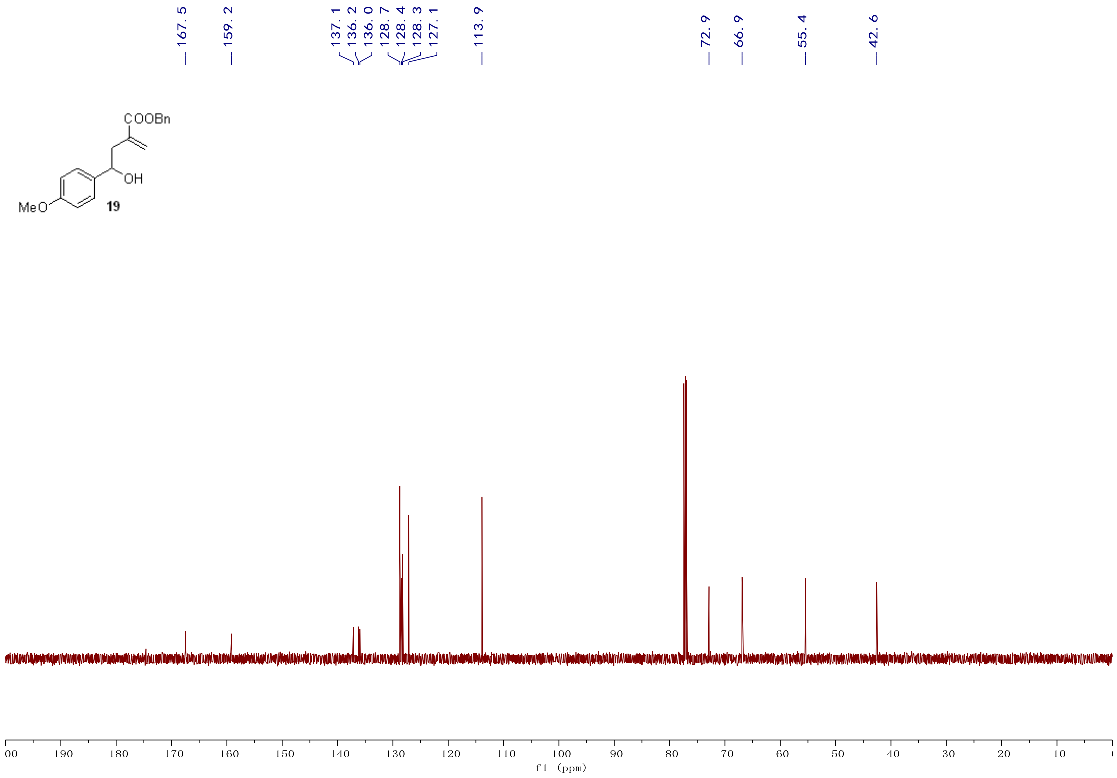

Figure S34. ^{13}**C NMR (126 MHz, CDCl3) spectrum of compound 19, related to Scheme 2.**

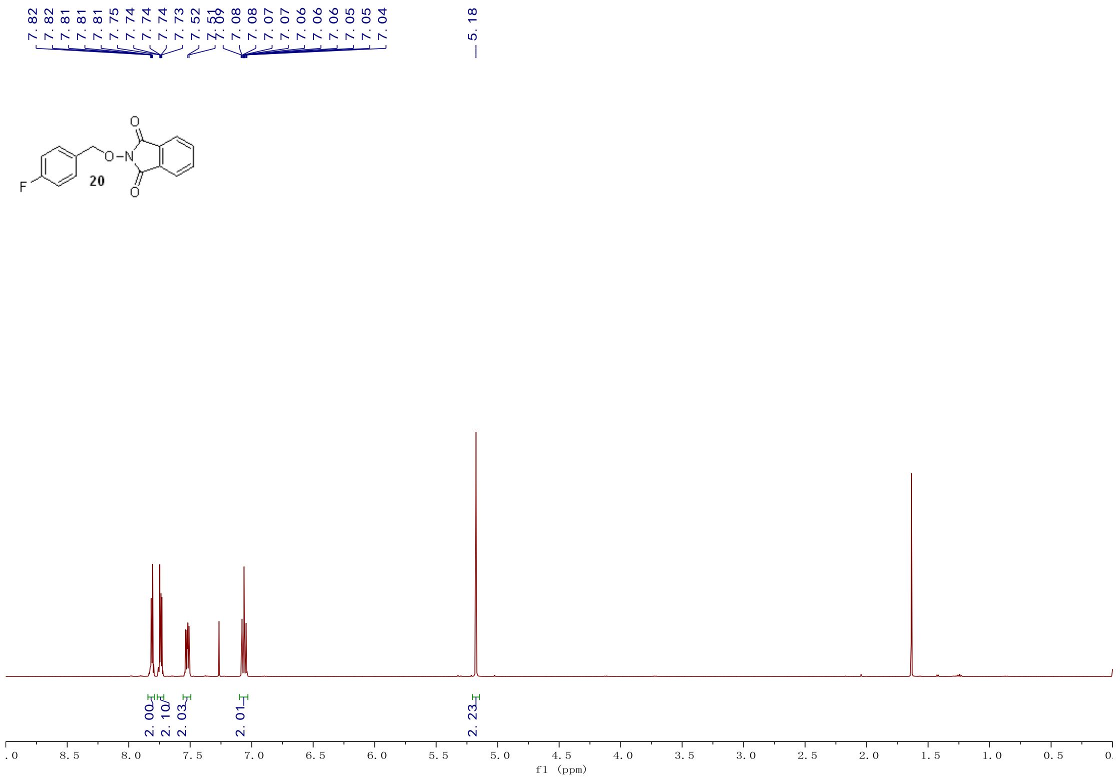

Figure S35. ^{1}H **NMR (500 MHz, CDCl3) spectrum of compound 20, related to Scheme 2.**

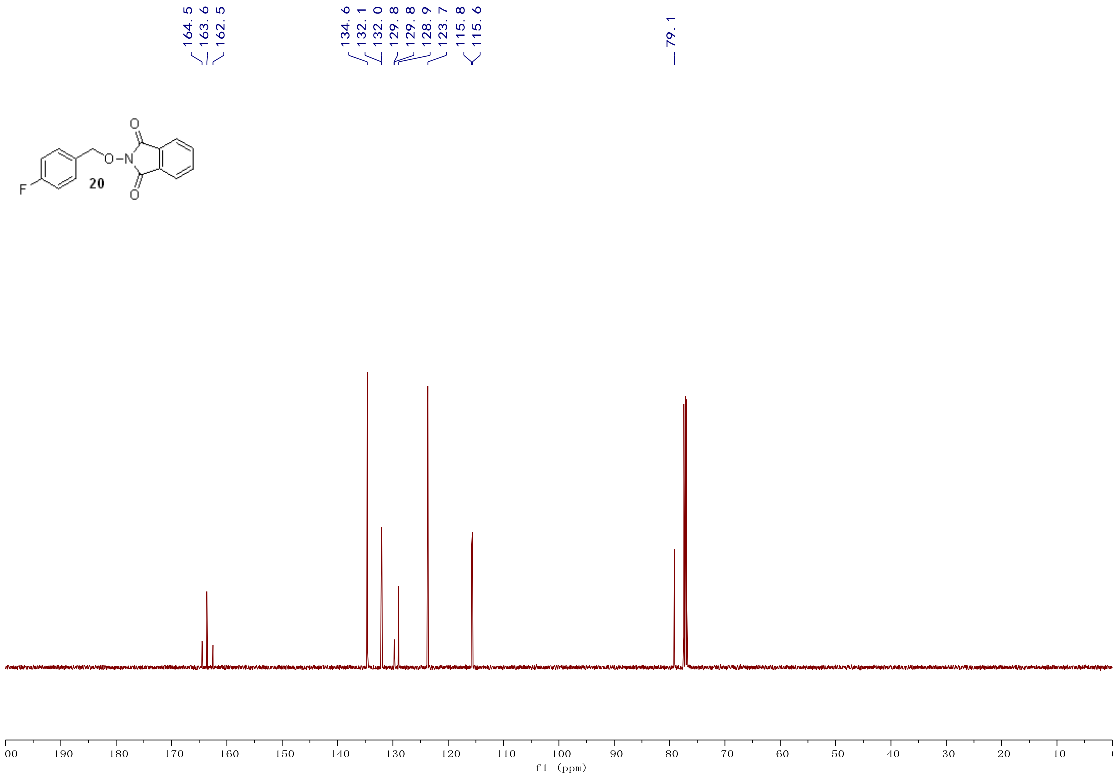

Figure S36. ^{13}C **NMR (126 MHz, CDCl3) spectrum of compound 20, related to Scheme 2.**

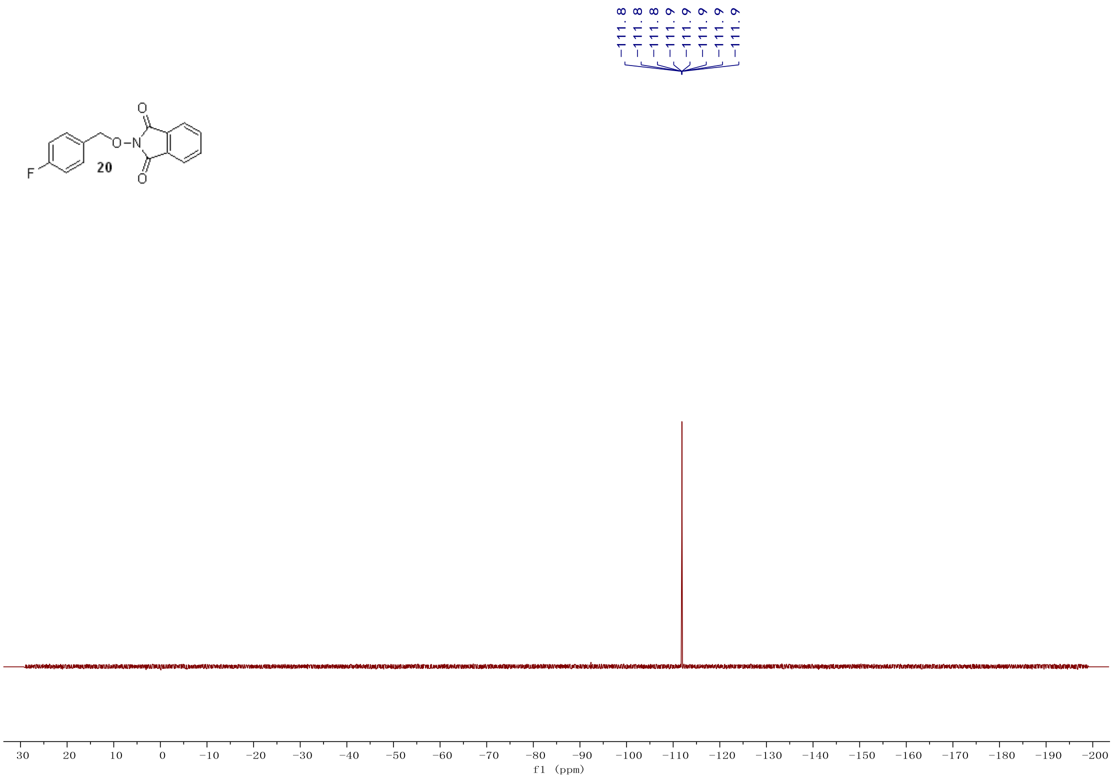

Figure S37. ^{19}F NMR (376 MHz, CDCl3) spectrum of compound 20, related to Scheme 2.

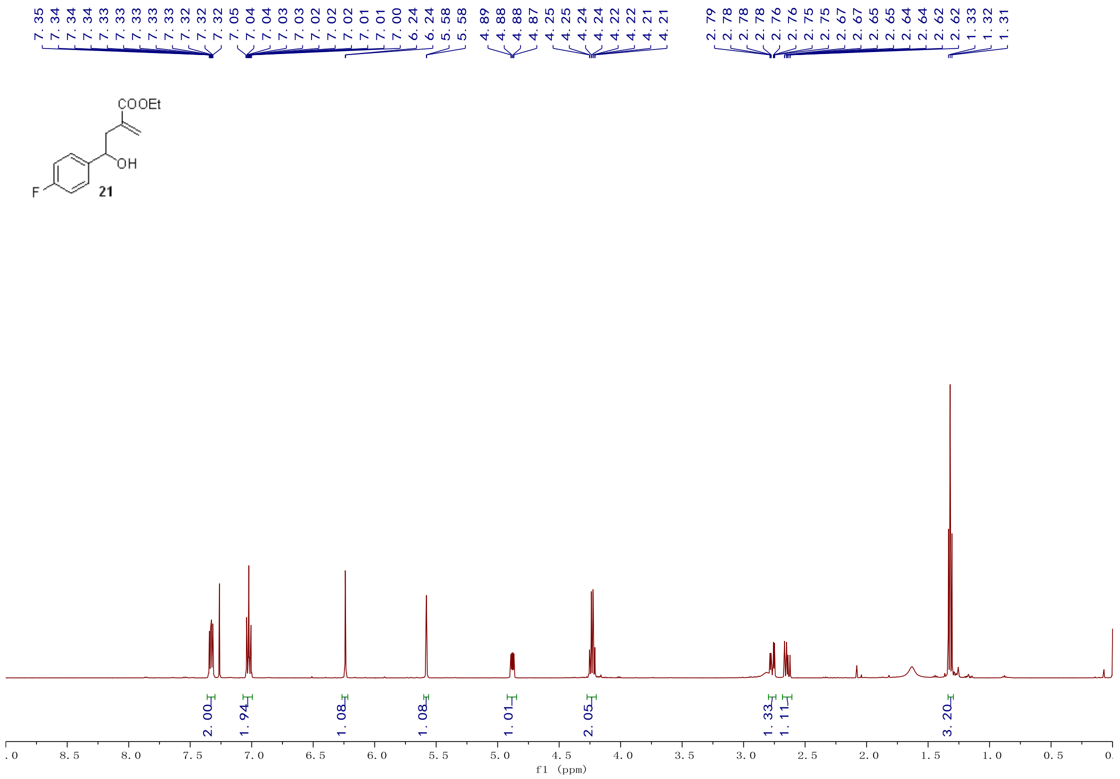

Figure S38. **^{1}H NMR (500 MHz, CDCl3) spectrum of compound 21, related to Scheme 2.**

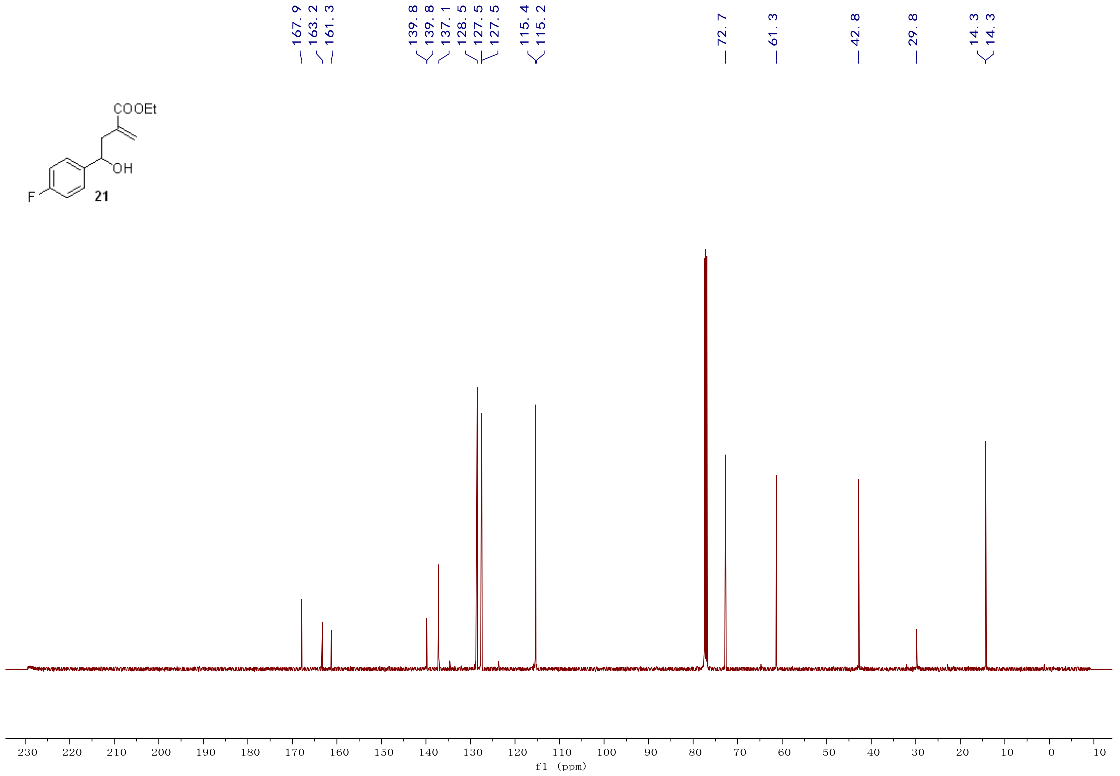

Figure S39. **^{13}C NMR (126 MHz, CDCl3) spectrum of compound 21, related to Scheme 2.**

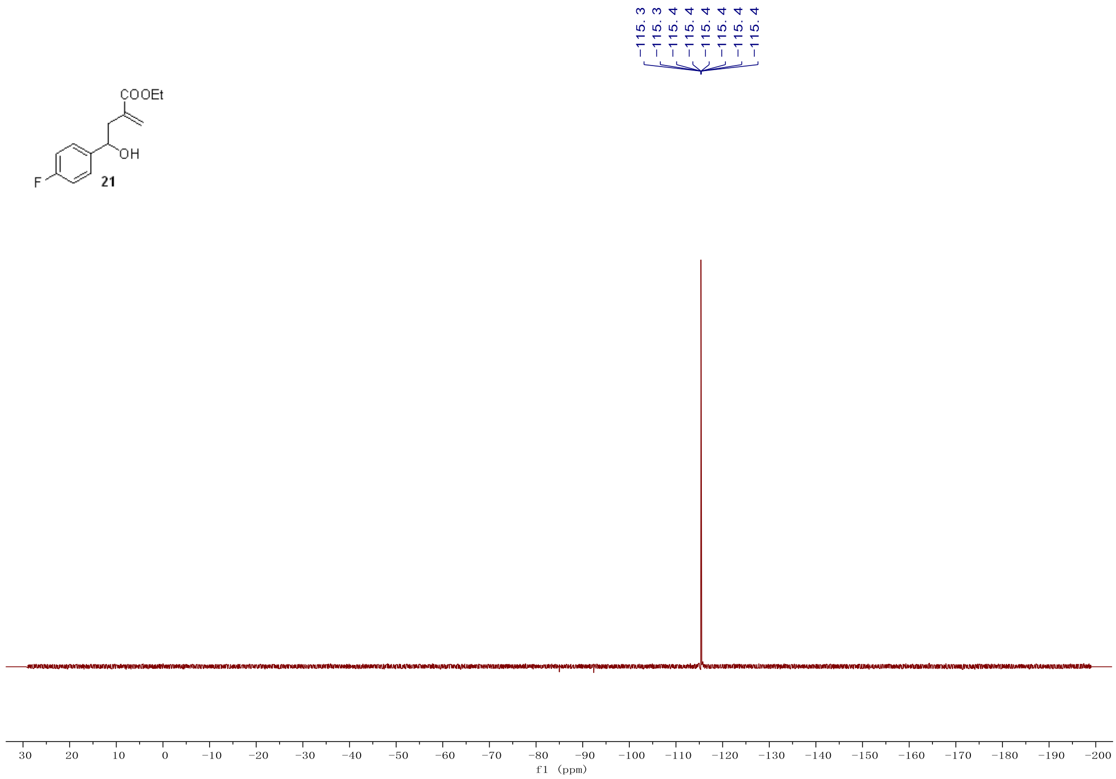

Figure S40. ^{19}F NMR (376 MHz, CDCl3) spectrum of compound 21, related to Scheme 2

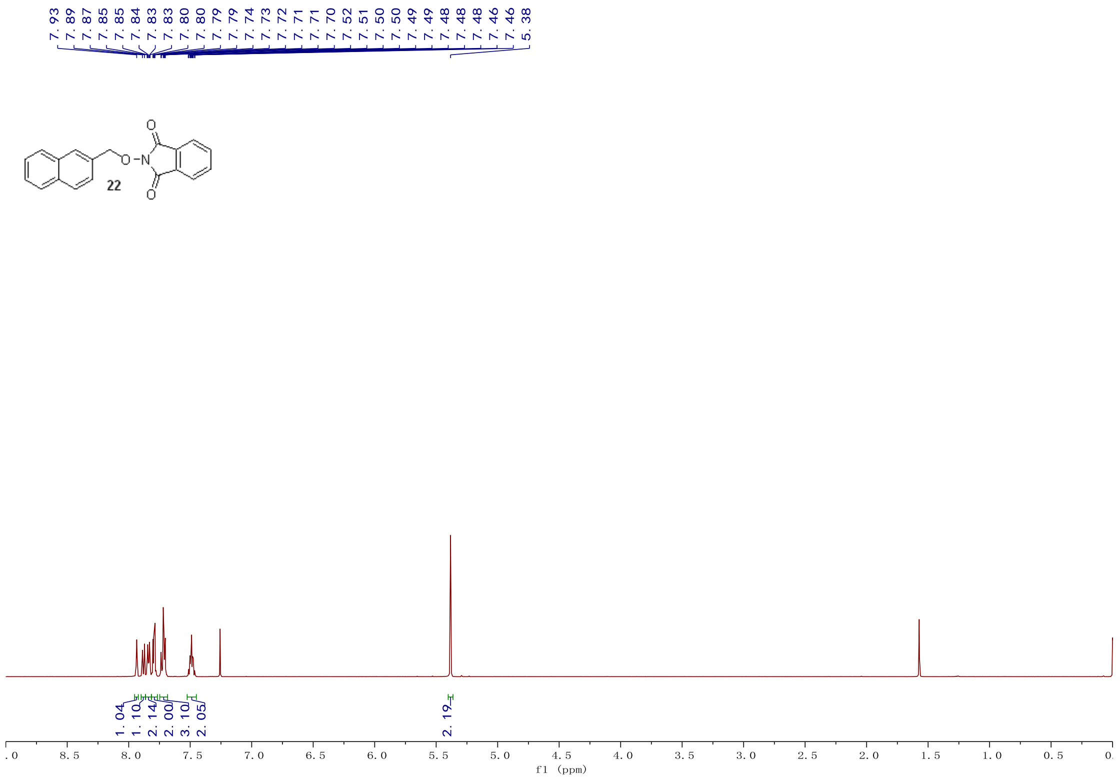

Figure S41. ^{1}H NMR (500 MHz, CDCl3) spectrum of compound 22, related to Scheme 2.

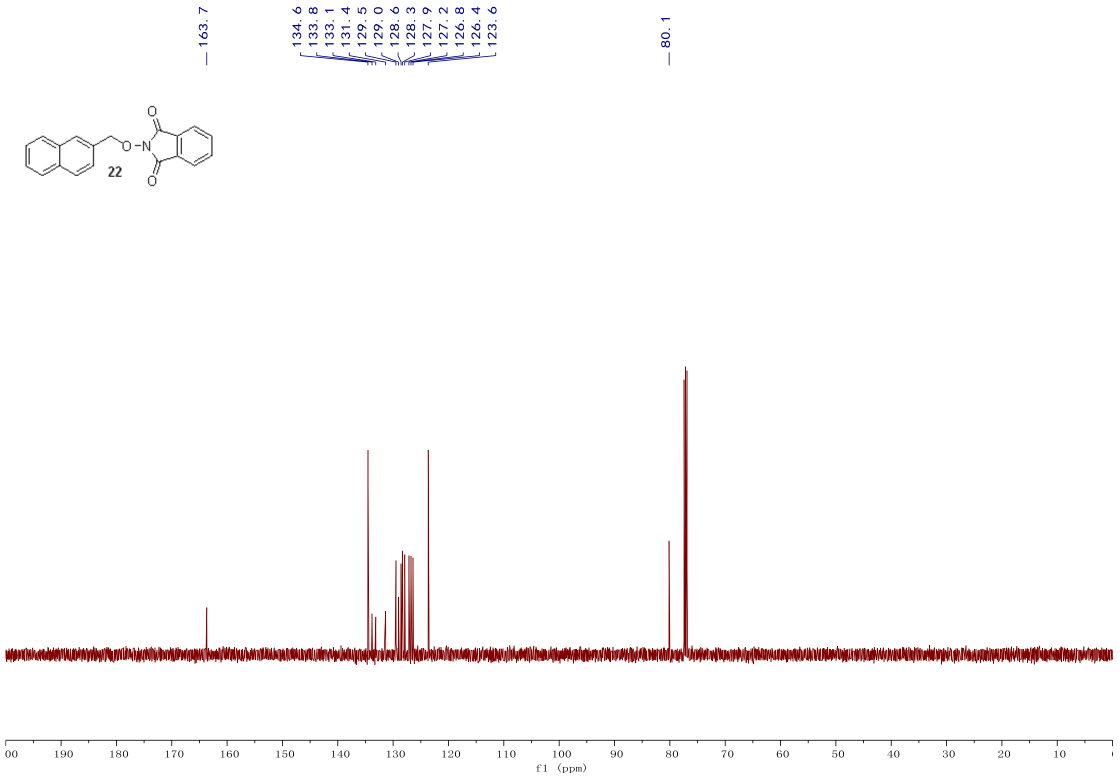

Figure S42. ^{13}C NMR (126 MHz, CDCl3) spectrum of compound 22, related to Scheme 2.

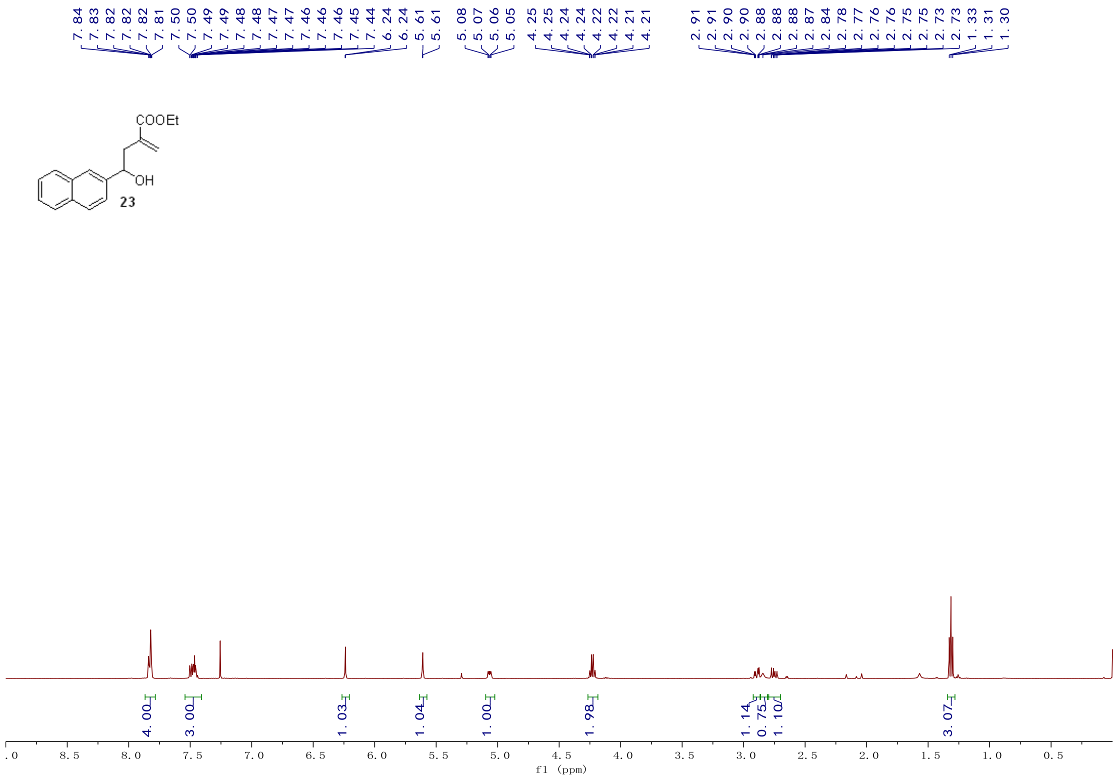

Figure S43. ^{1}H NMR (500 MHz, CDCl3) spectrum of compound 23, related to Scheme 2.

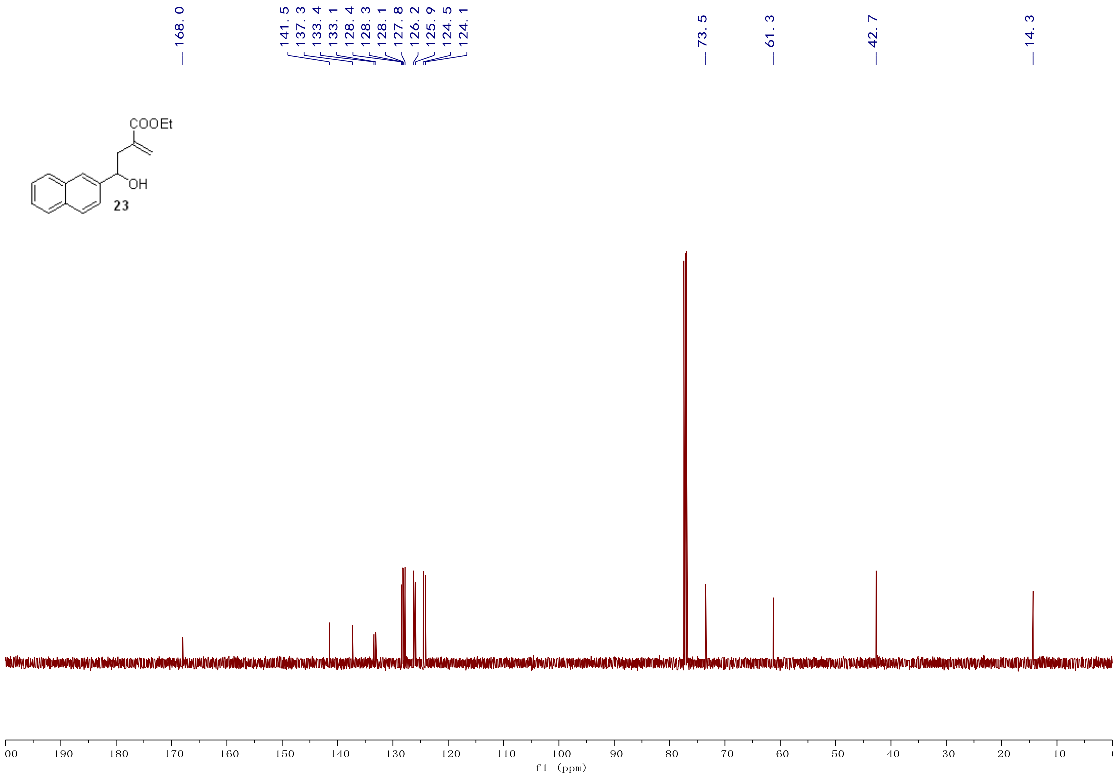

Figure S44. ^{13}C NMR (126 MHz, CDCl3) spectrum of compound 23, related to Scheme 2.

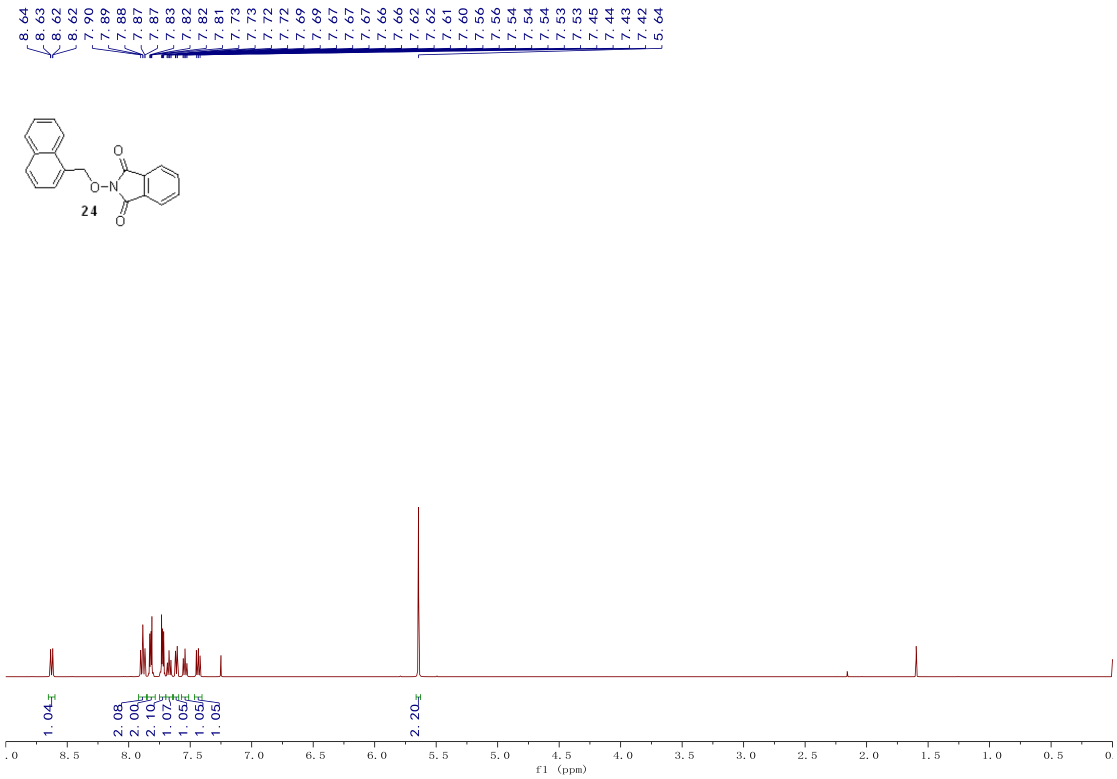

Figure S45. ^{1}H NMR (500 MHz, CDCl3) spectrum of compound 24, related to Scheme 2.

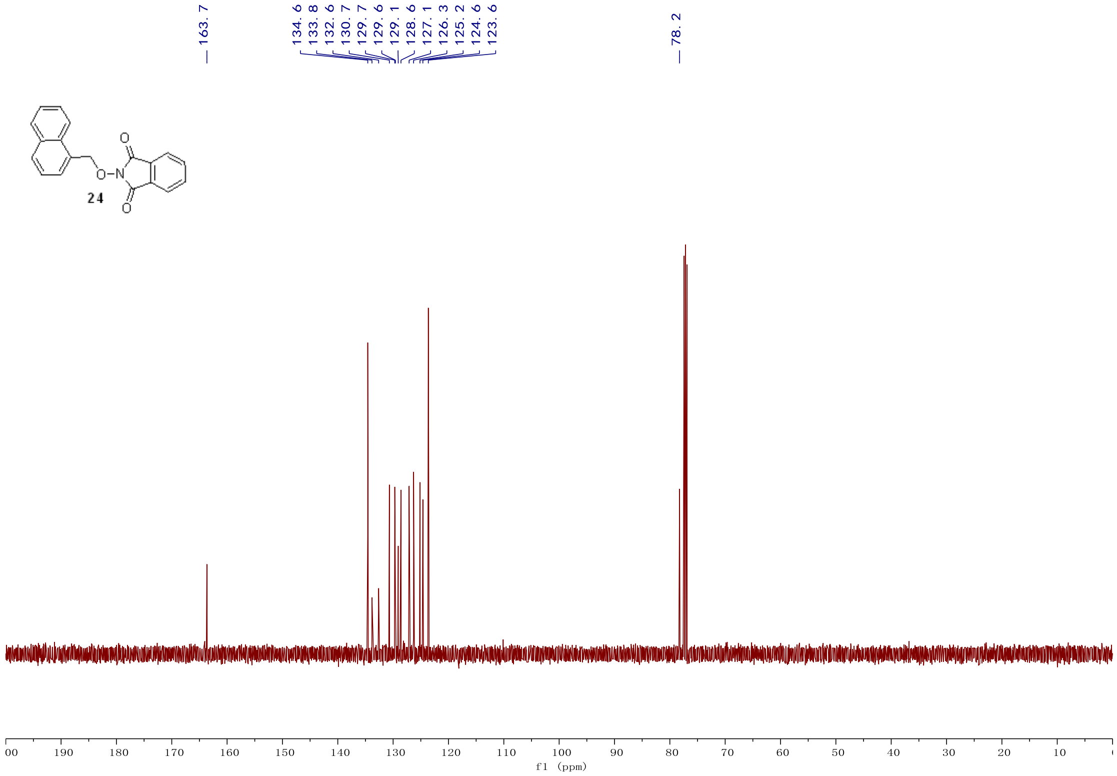

Figure S46. ^{13}C NMR (126 MHz, CDCl3) spectrum of compound 24, related to Scheme 2.

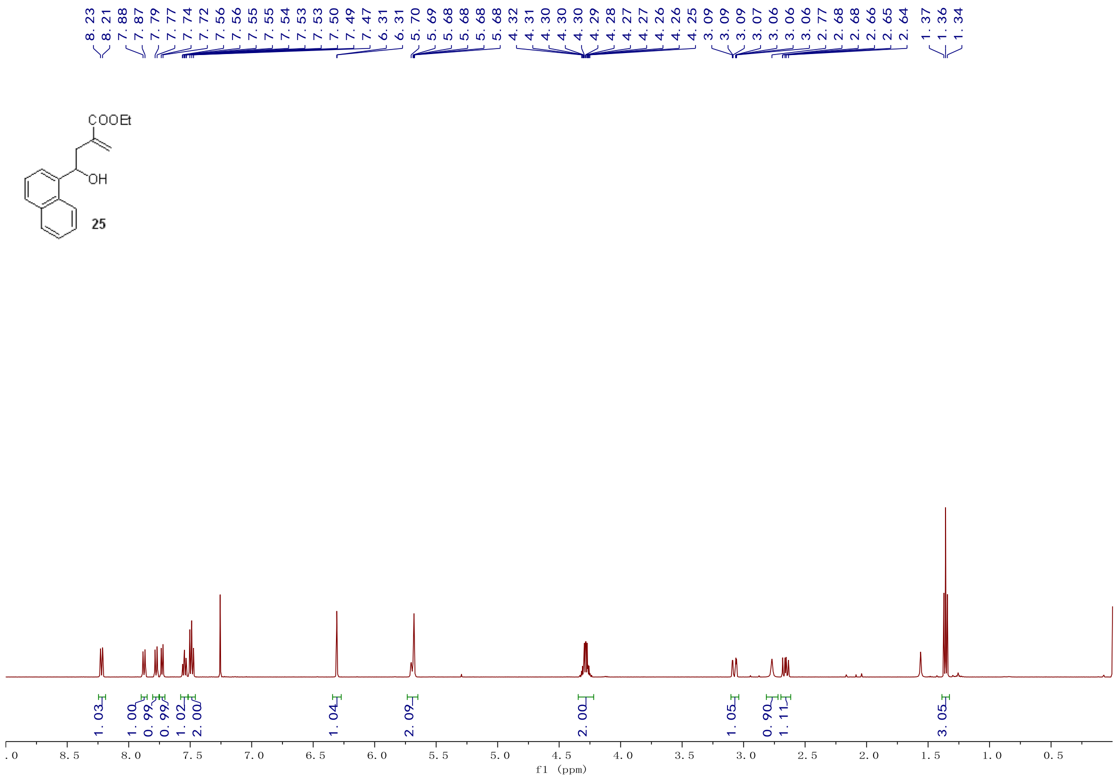

Figure S47. ^{1}H NMR (500 MHz, CDCl3) spectrum of compound 25, related to Scheme 2.

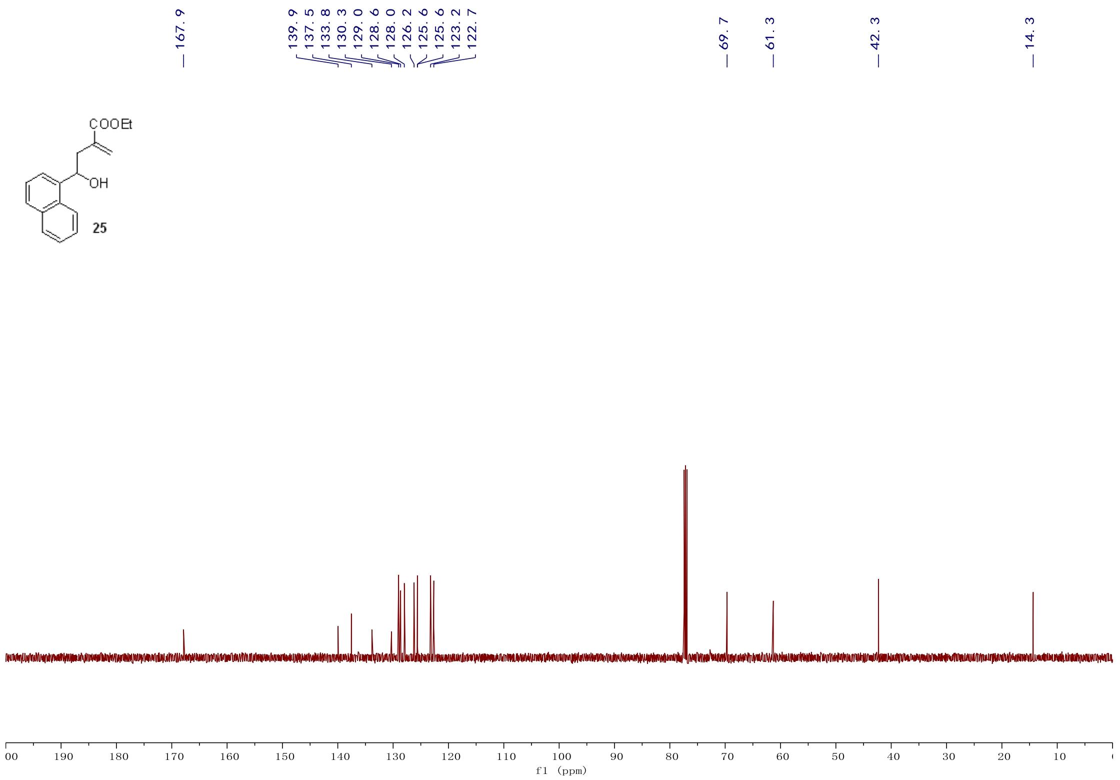

Figure S48. ^{13}C NMR (126 MHz, CDCl3) spectrum of compound 25, related to Scheme 2.

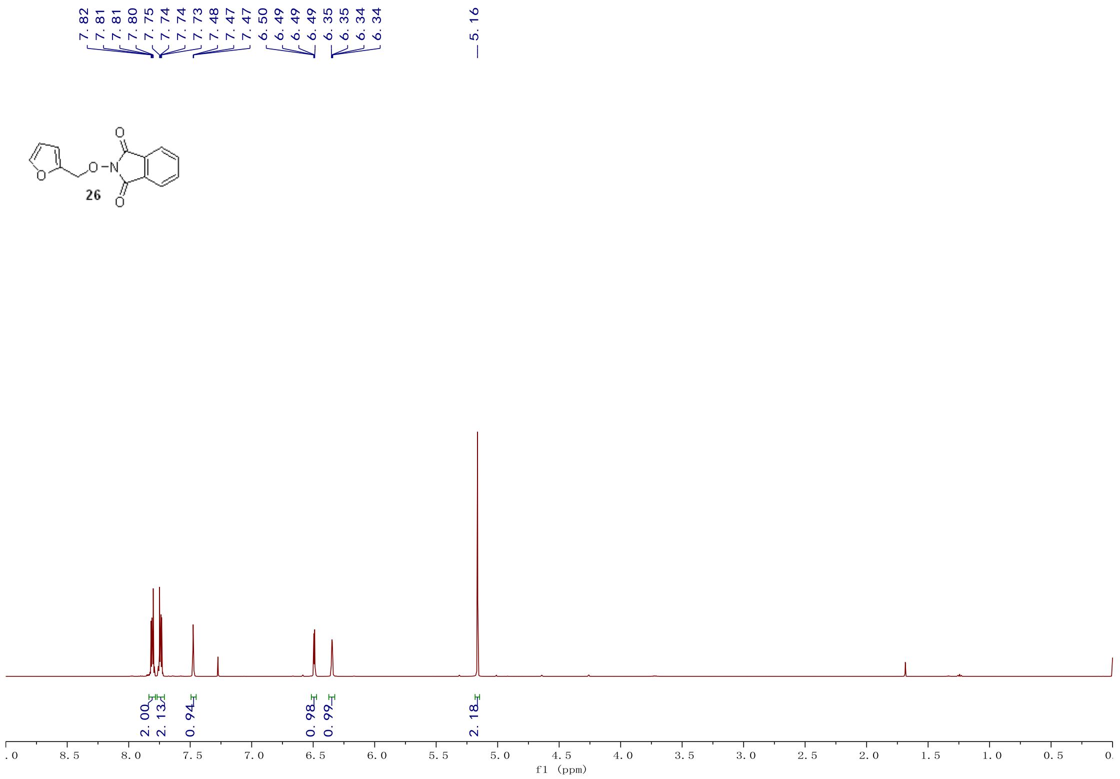

Figure S49. ^{1}H NMR (500 MHz, CDCl3) spectrum of compound 26, related to Scheme 2.

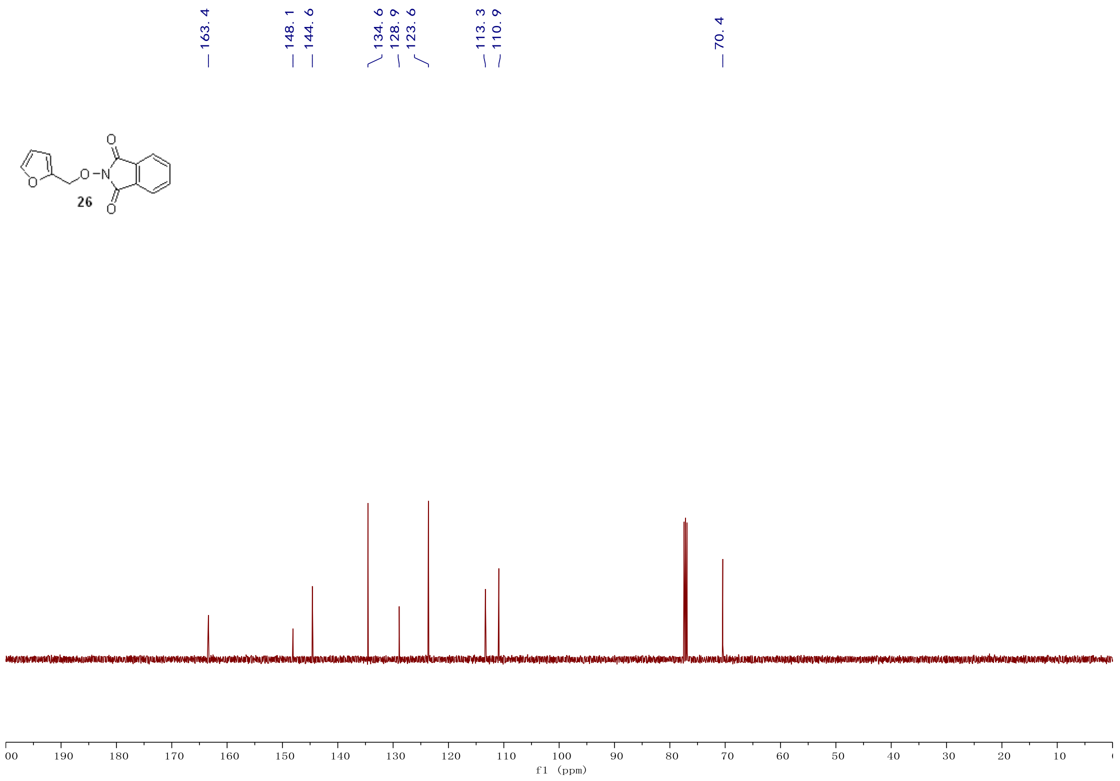

Figure S50. ^{13}C NMR (126 MHz, CDCl3) spectrum of compound 26, related to Scheme 2.

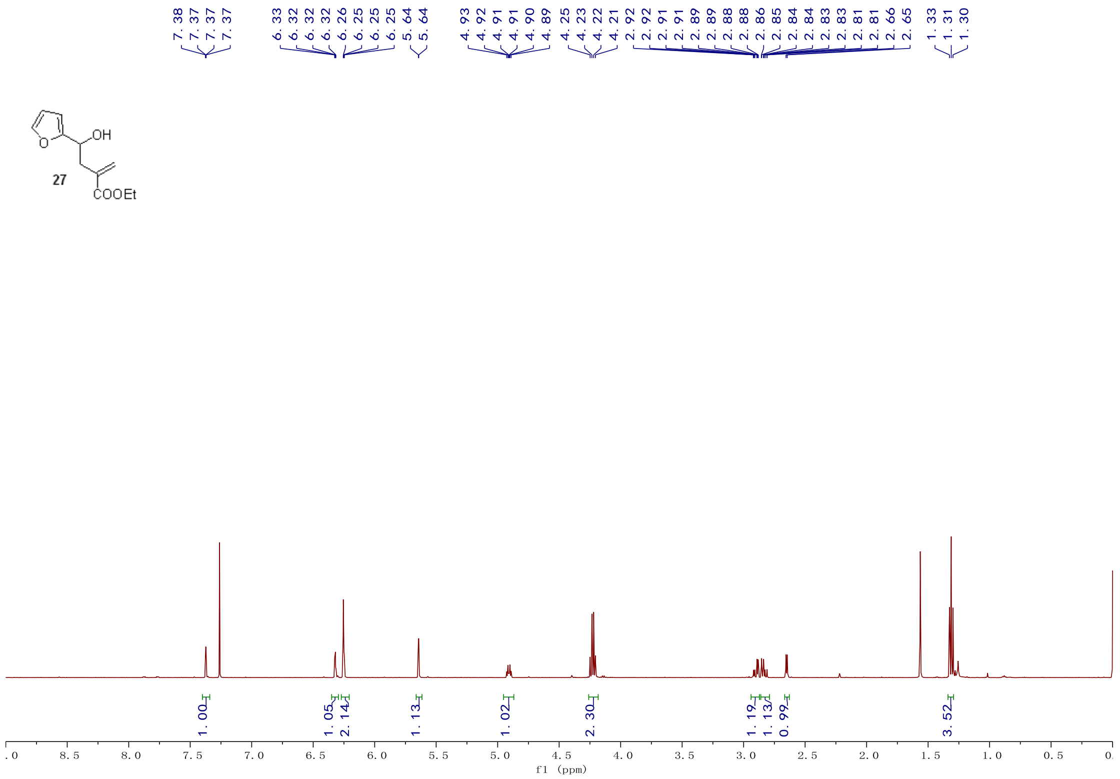

Figure S51. ^{1}H NMR (500 MHz, CDCl3) spectrum of compound 27, related to Scheme 2.

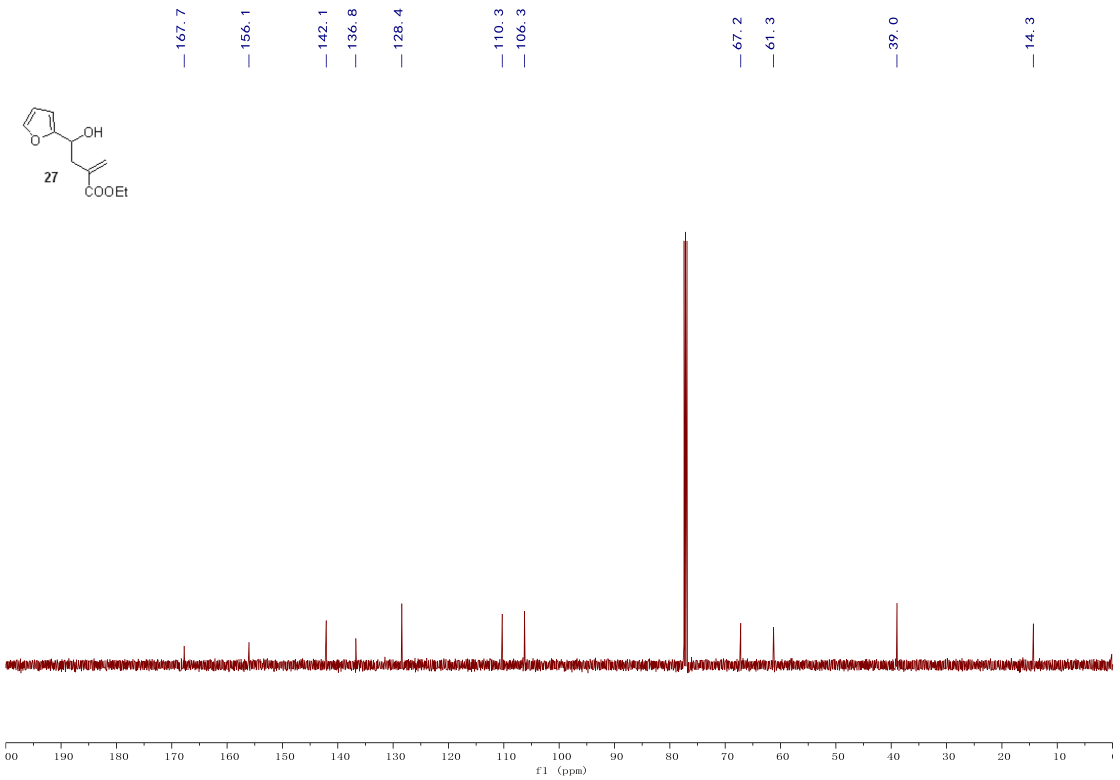

Figure S52. ^{13}C NMR (126 MHz, CDCl3) spectrum of compound 27, related to Scheme 2.

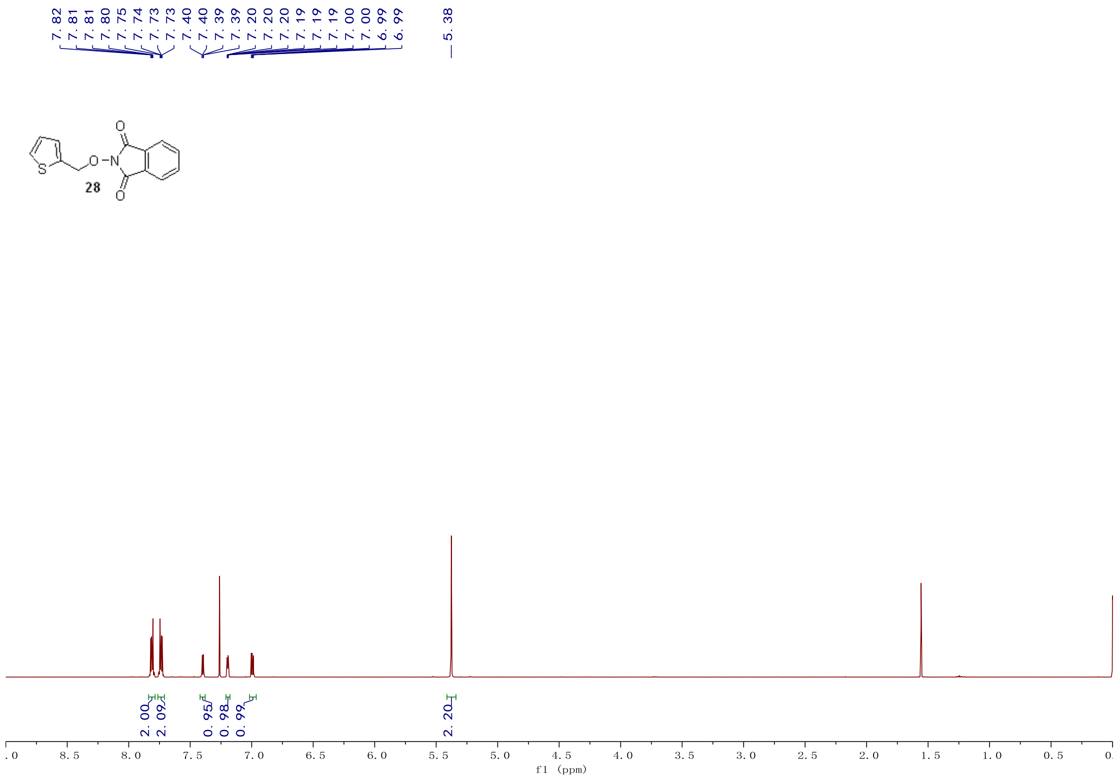

Figure S53. ^{1}**H NMR (500 MHz, CDCl3) spectrum of compound 28, related to Scheme 2.**

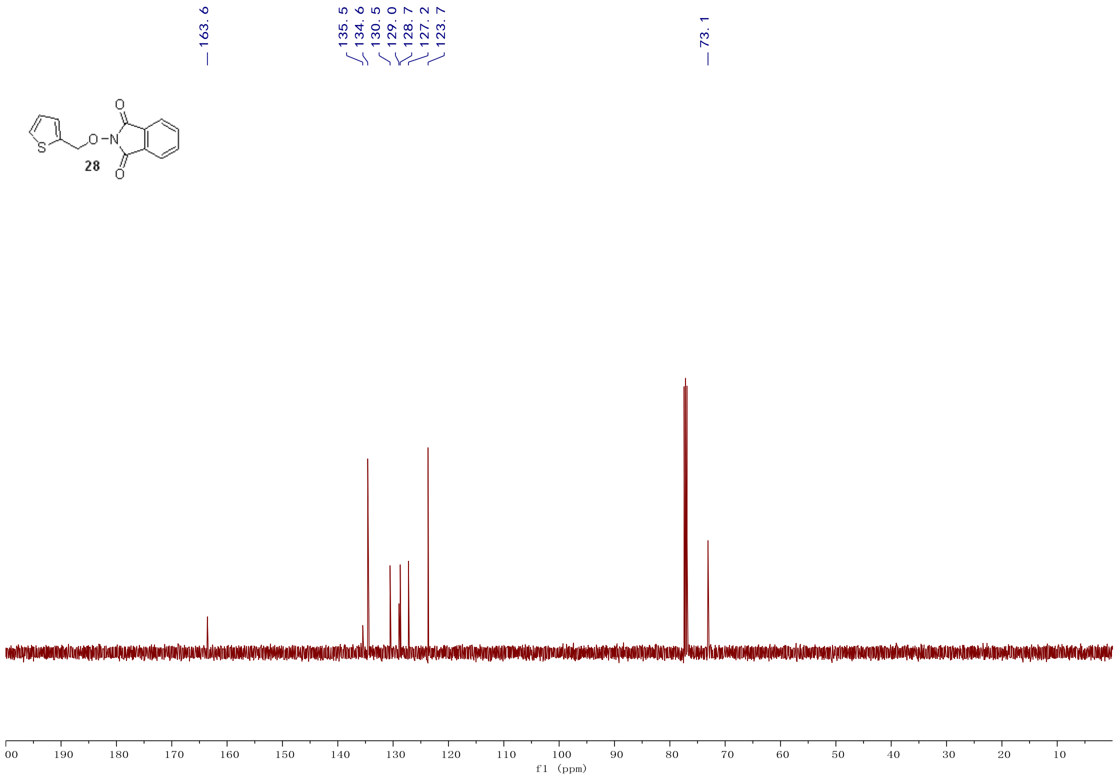

Figure S54. ^{13}**C NMR (126 MHz, CDCl3) spectrum of compound 28, related to Scheme 2.**

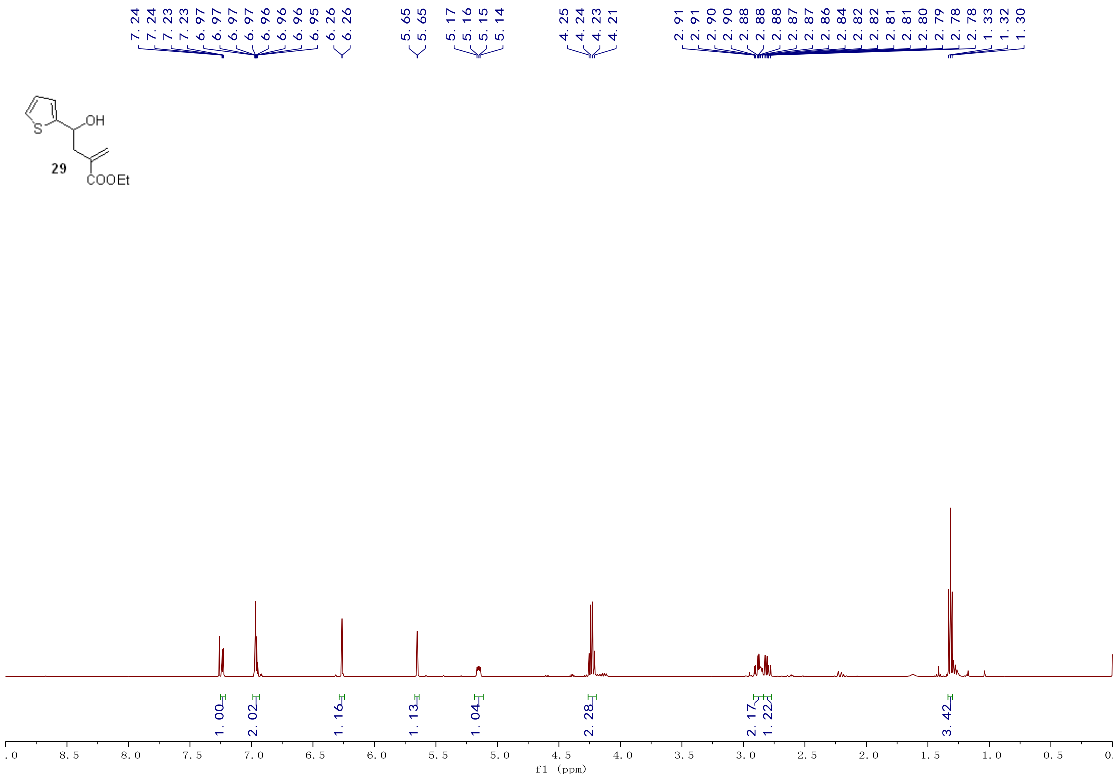

Figure S55. ^{1}H **NMR (500 MHz, CDCl3) spectrum of compound 29, related to Scheme 2.**

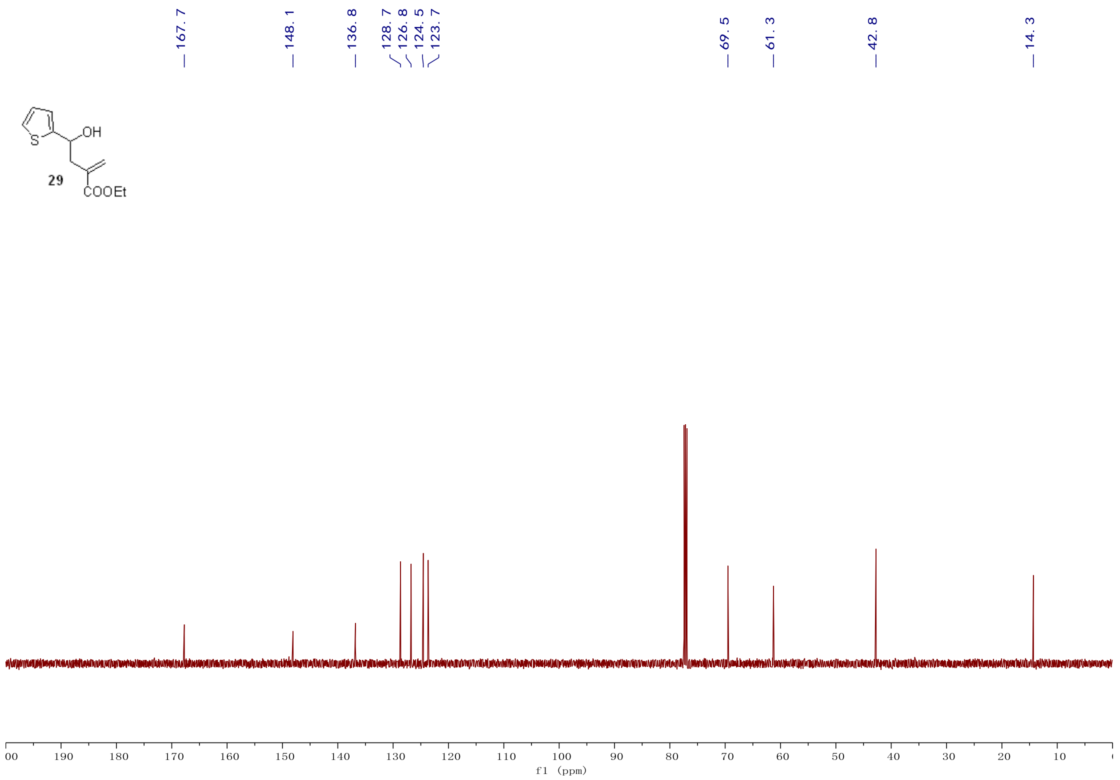

Figure S56. ^{13}C **NMR (126 MHz, CDCl3) spectrum of compound 29, related to Scheme 2.**

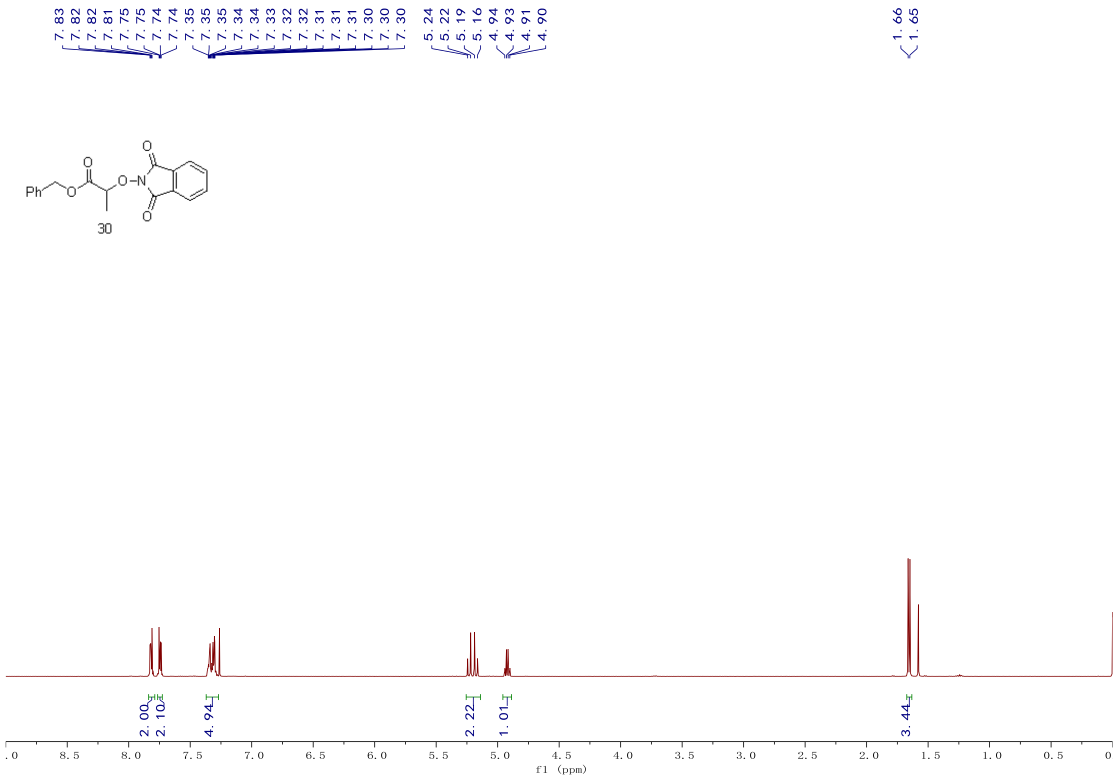

Figure S57. ^{1}**H NMR (500 MHz, CDCl3) spectrum of compound 30, related to Scheme 2.**

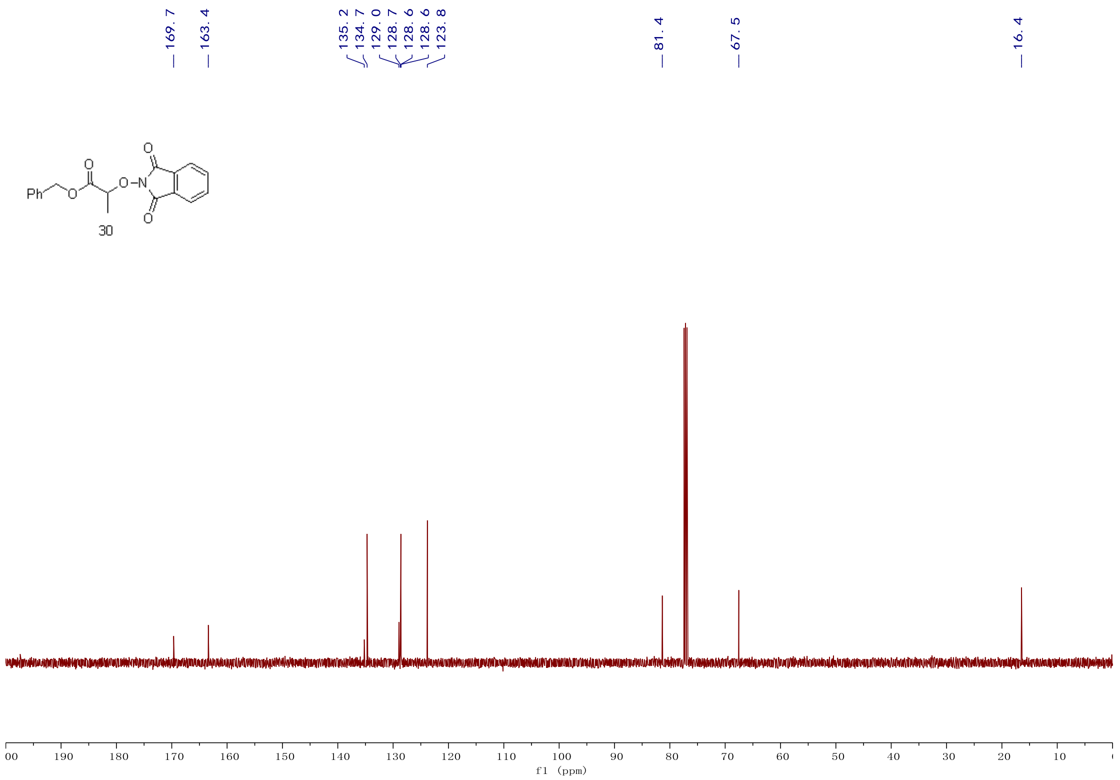

Figure S58. ^{13}**C NMR (126 MHz, CDCl3) spectrum of compound 30, related to Scheme 2.**

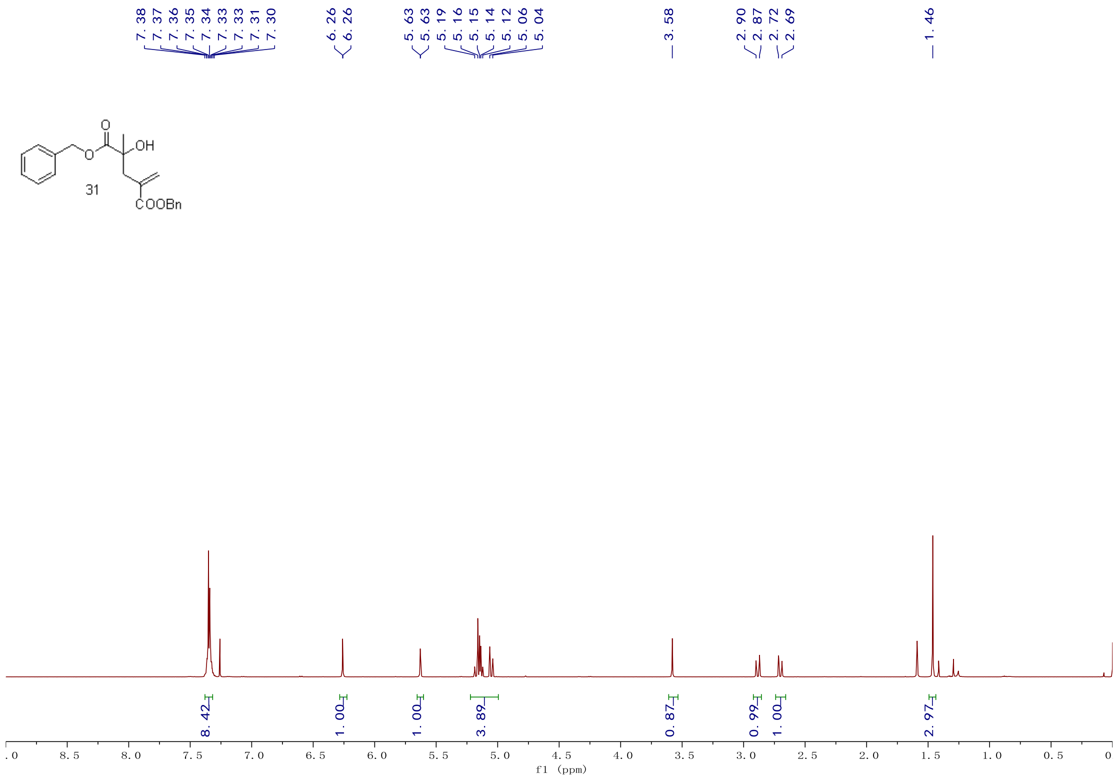

Figure S59. ^{1}H NMR (500 MHz, CDCl3) spectrum of compound 31, related to Scheme 2.

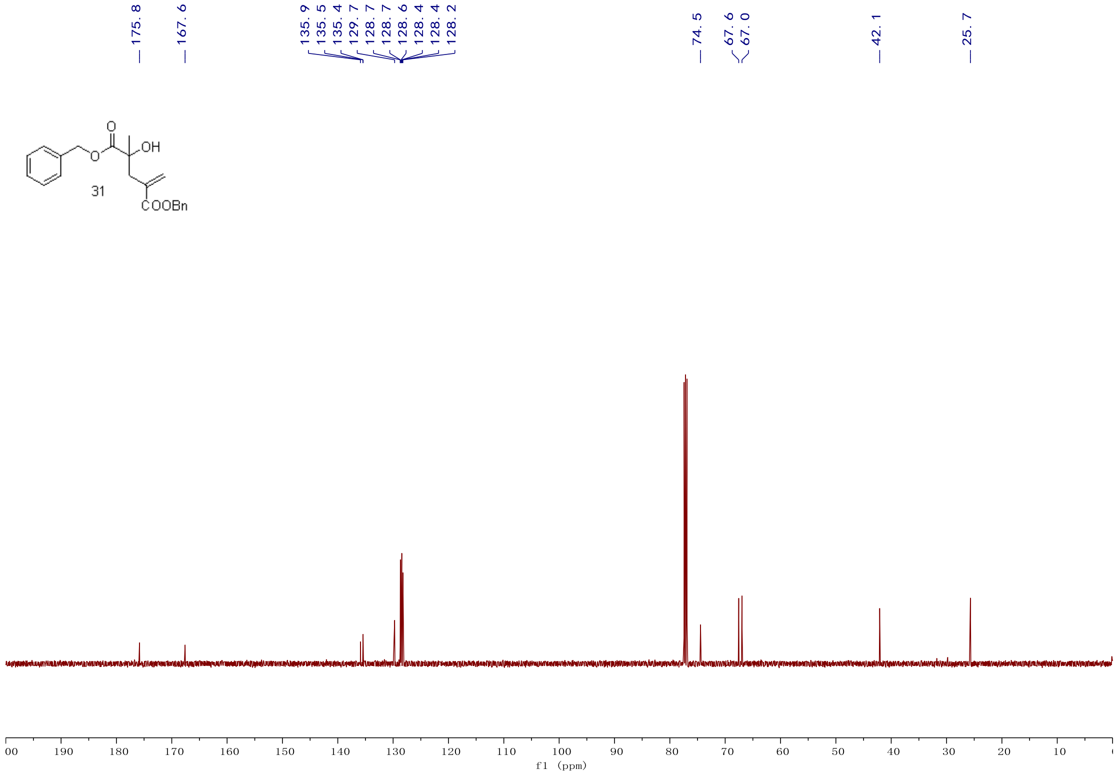

Figure S60. ^{13}C NMR (126 MHz, CDCl3) spectrum of compound 31, related to Scheme 2.

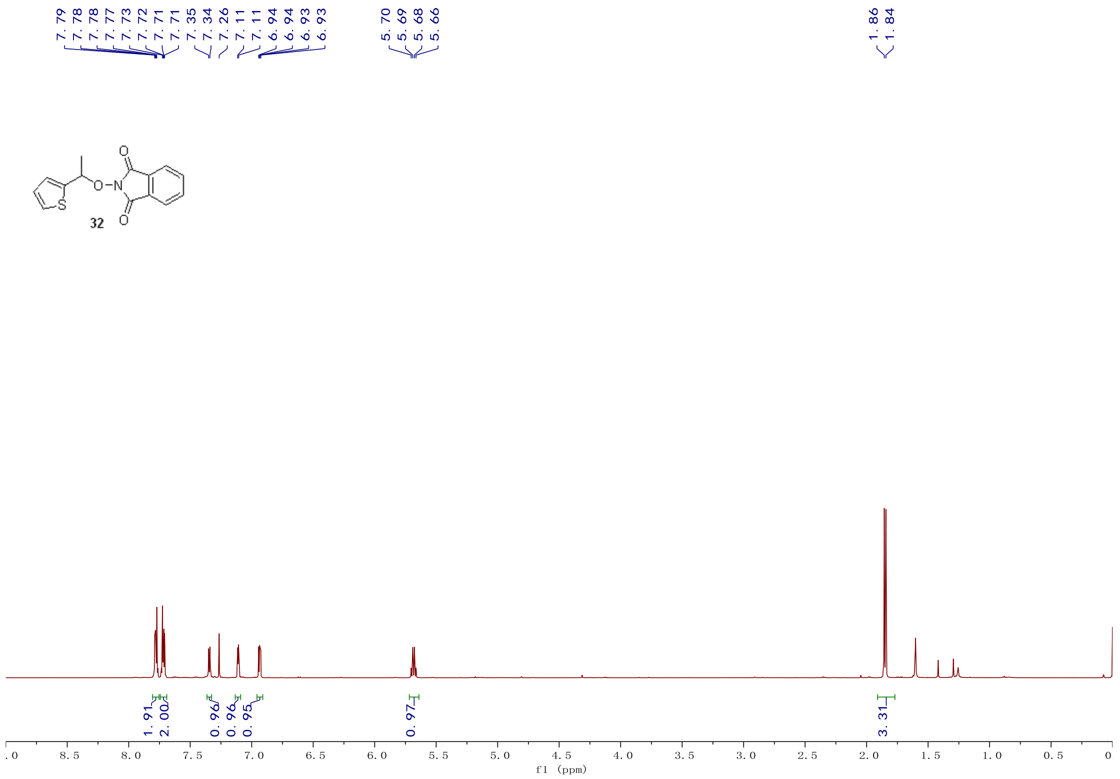

Figure S61. ^{1}H **NMR (500 MHz, CDCl3) spectrum of compound 32, related to Scheme 2.**

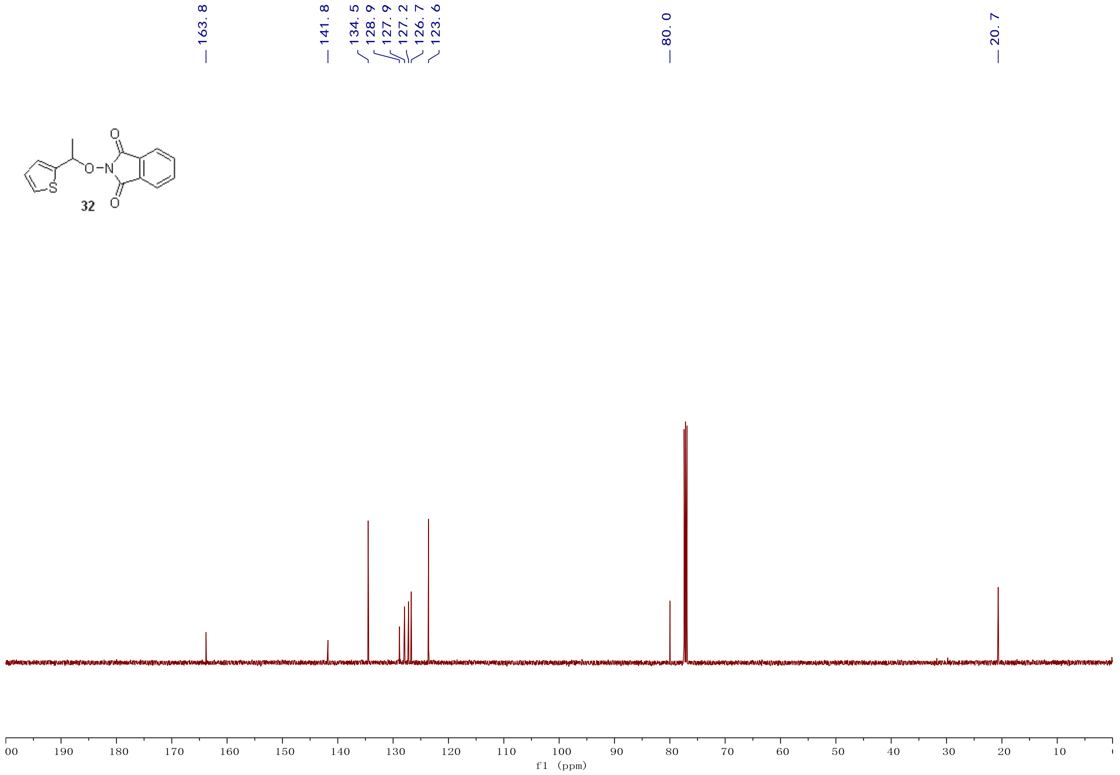

Figure S62. ^{13}C **NMR (126 MHz, CDCl3) spectrum of compound 32, related to Scheme 2.**

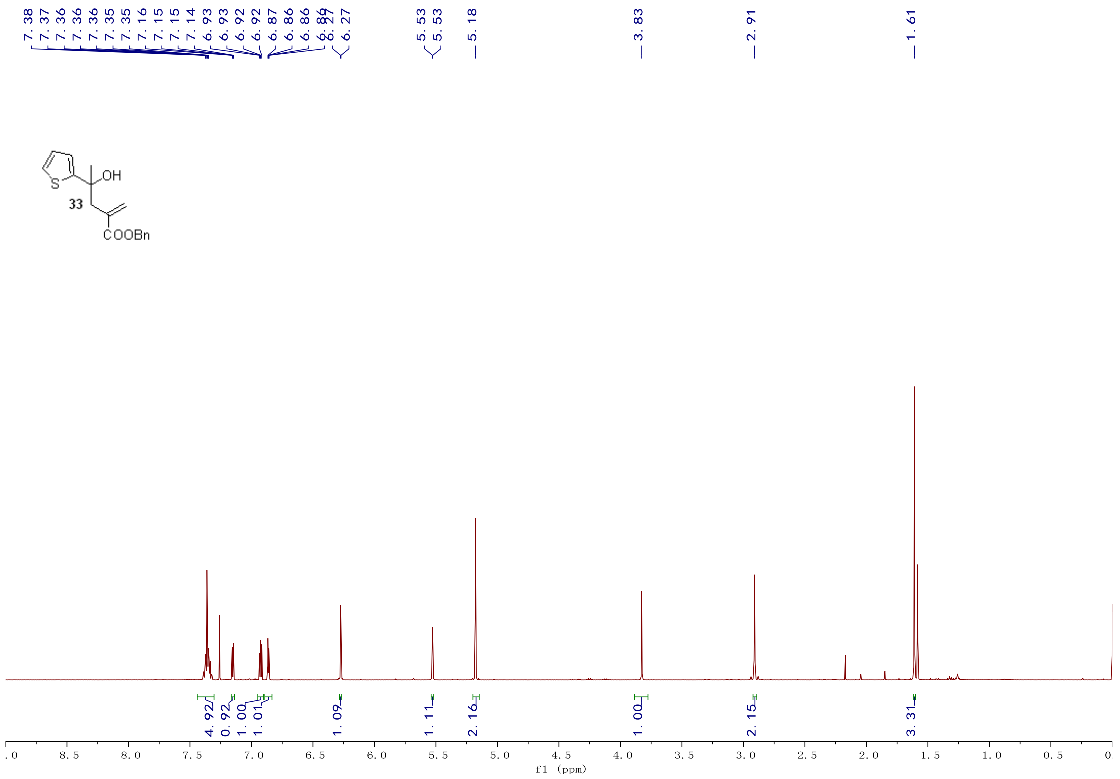

Figure S63. ^{1}H **NMR (500 MHz, CDCl3) spectrum of compound 33, related to Scheme 2.**

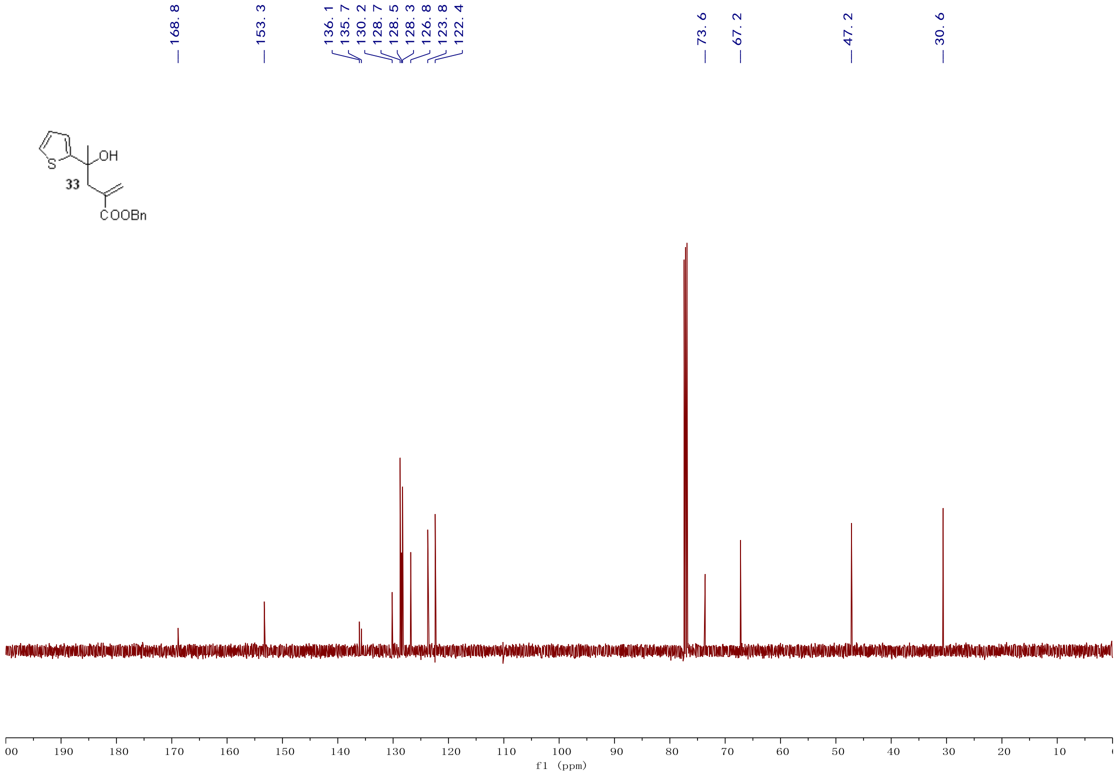

Figure S64. ^{13}C **NMR (126 MHz, CDCl3) spectrum of compound 33, related to Scheme 2.**

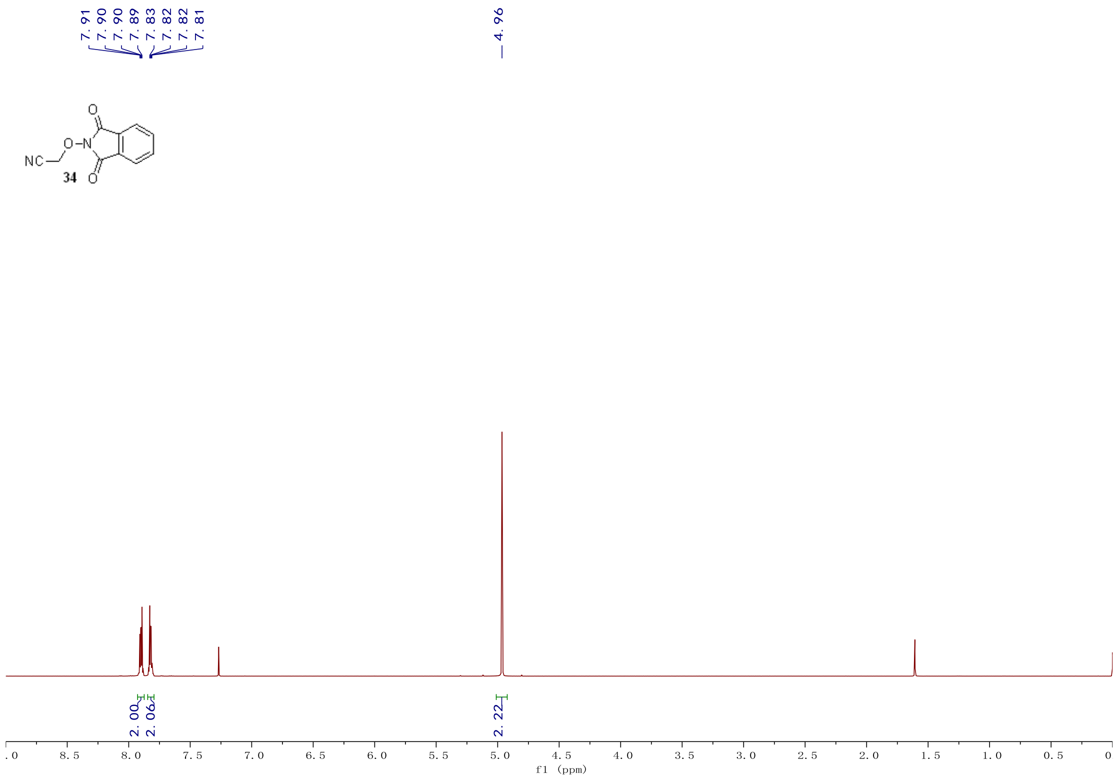

Figure S65. ^{1}H NMR (500 MHz, CDCl3) spectrum of compound 34, related to Scheme 2.

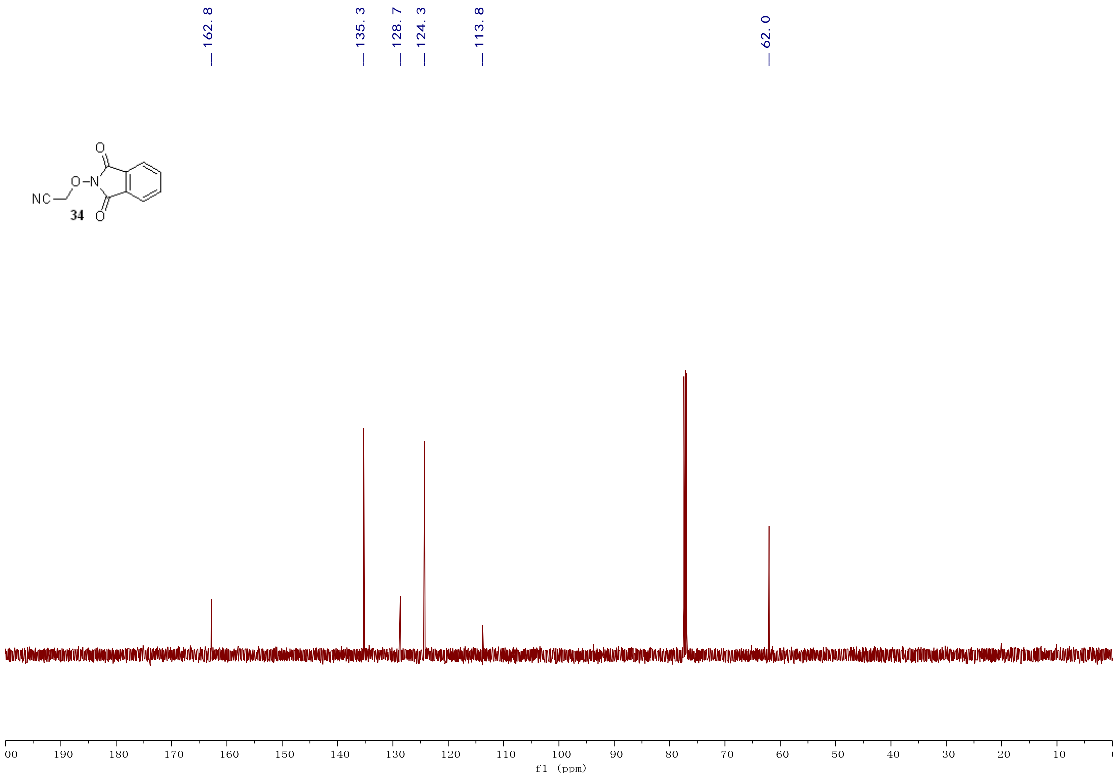

Figure S66. ^{13}C NMR (126 MHz, CDCl3) spectrum of compound 34, related to Scheme 2.

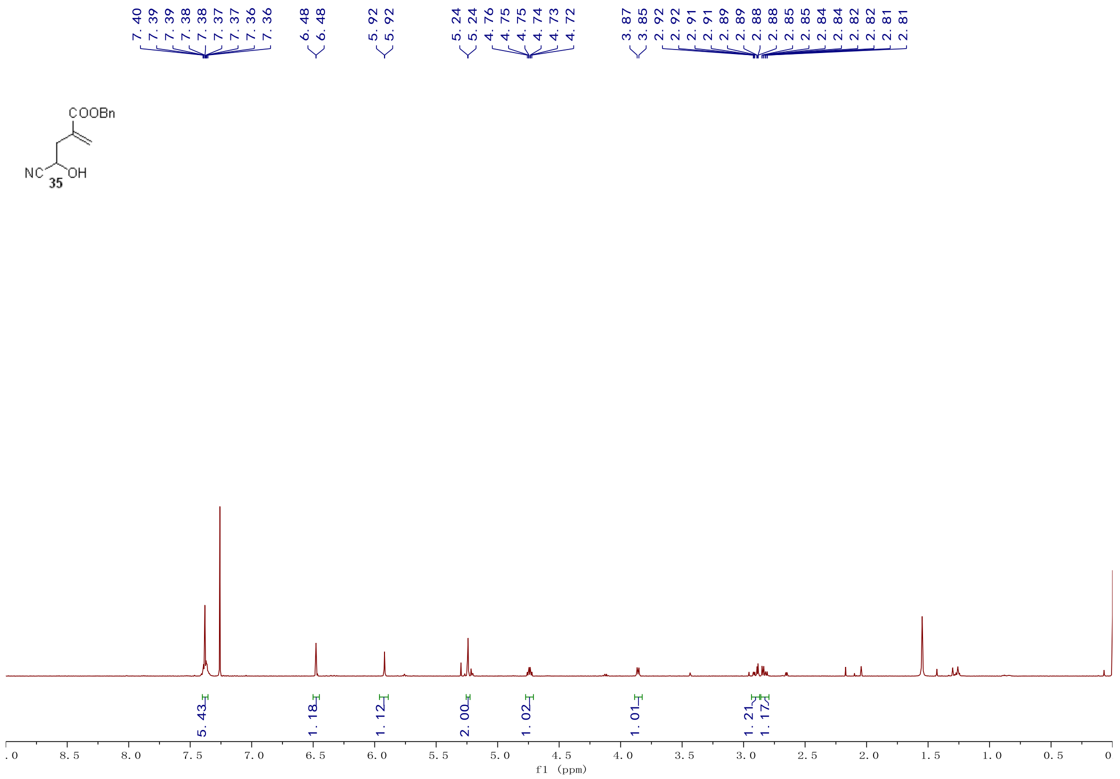

Figure S67. ^{1}H **NMR (500 MHz, CDCl3) spectrum of compound 35, related to Scheme 2.**

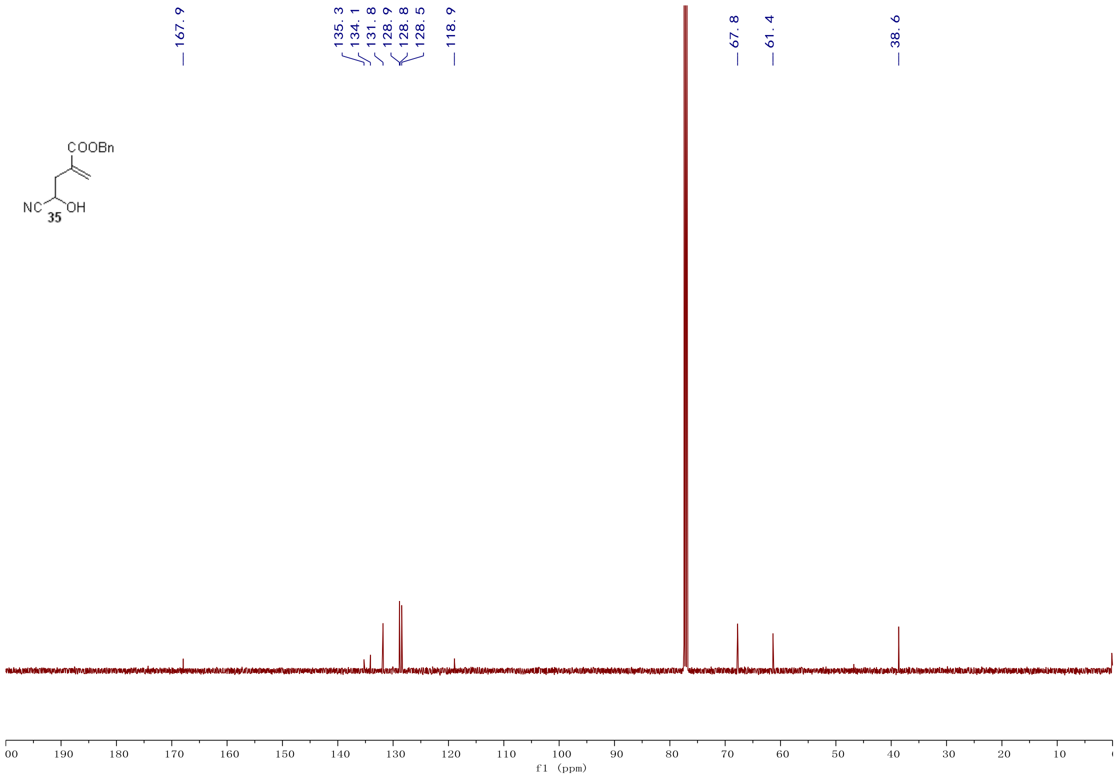

Figure S68. ^{13}C **NMR (126 MHz, CDCl3) spectrum of compound 36, related to Scheme 2.**

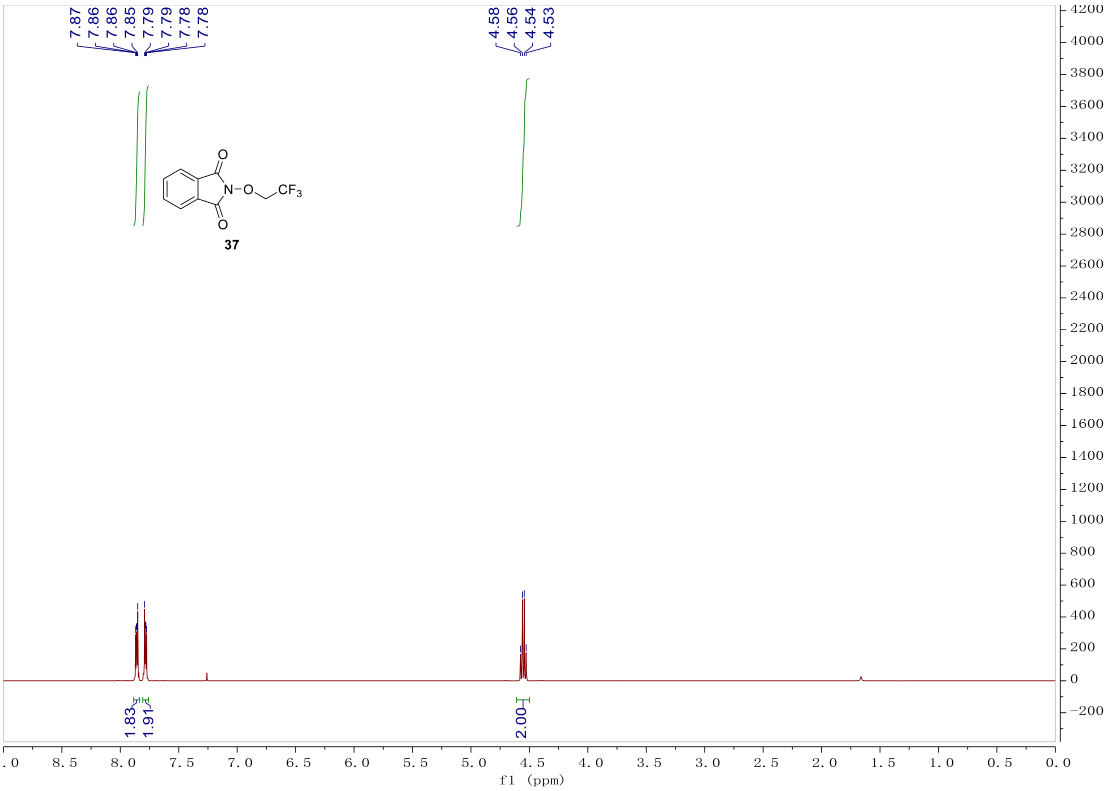

Figure S69. ^{1}H NMR (500 MHz, CDCl3) spectrum of compound 37, related to Scheme 2.

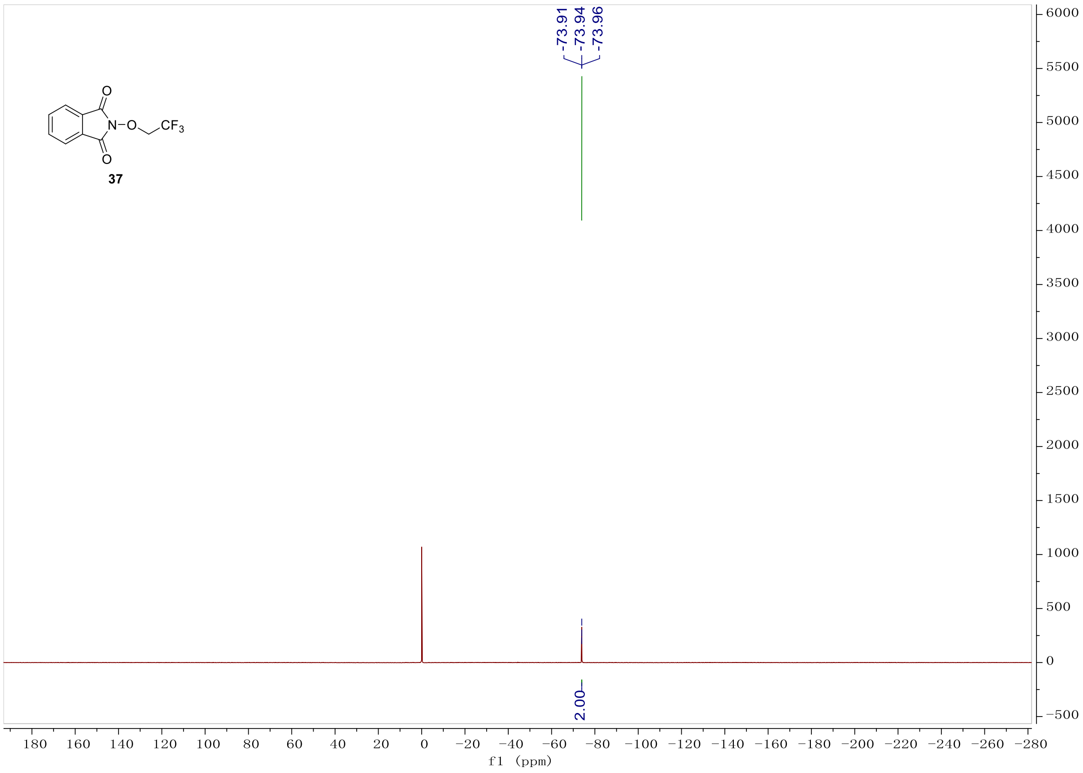

Figure S70. ^{13}C NMR (126 MHz, CDCl3) spectrum of compound 37, related to Scheme 2.

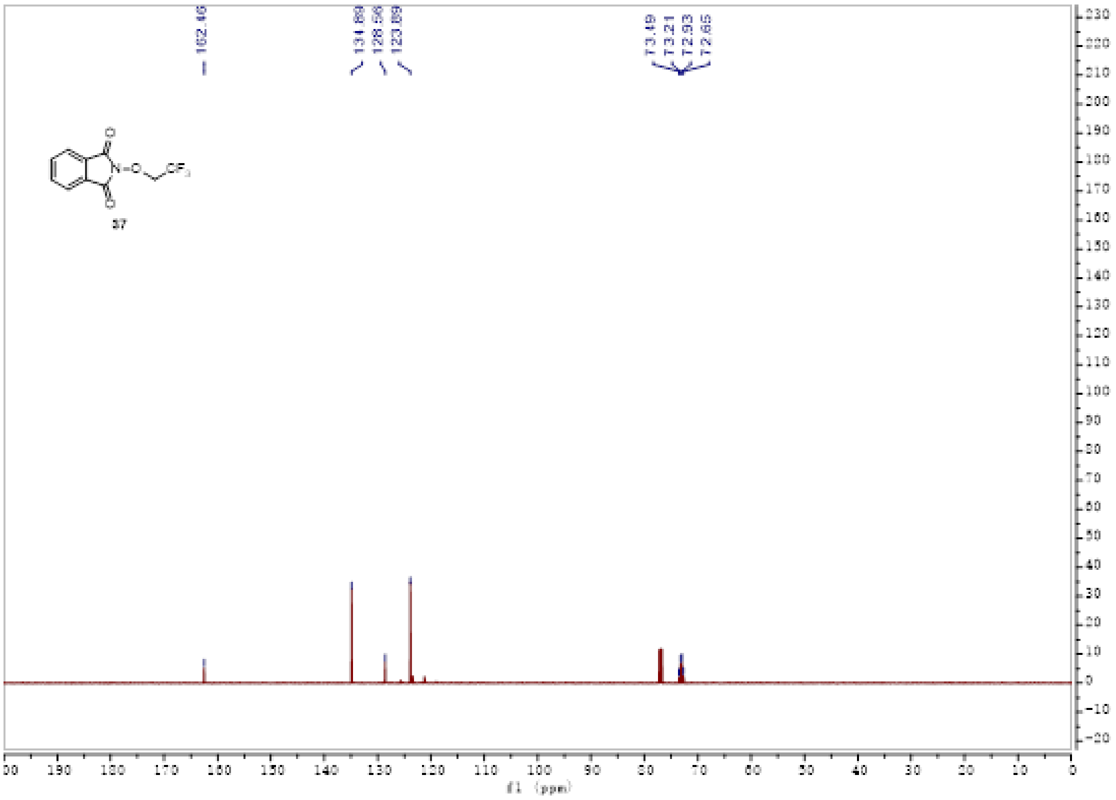

Figure S71. ^{19}F NMR (376 MHz, CDCl3) spectrum of compound 37, related to Scheme 2

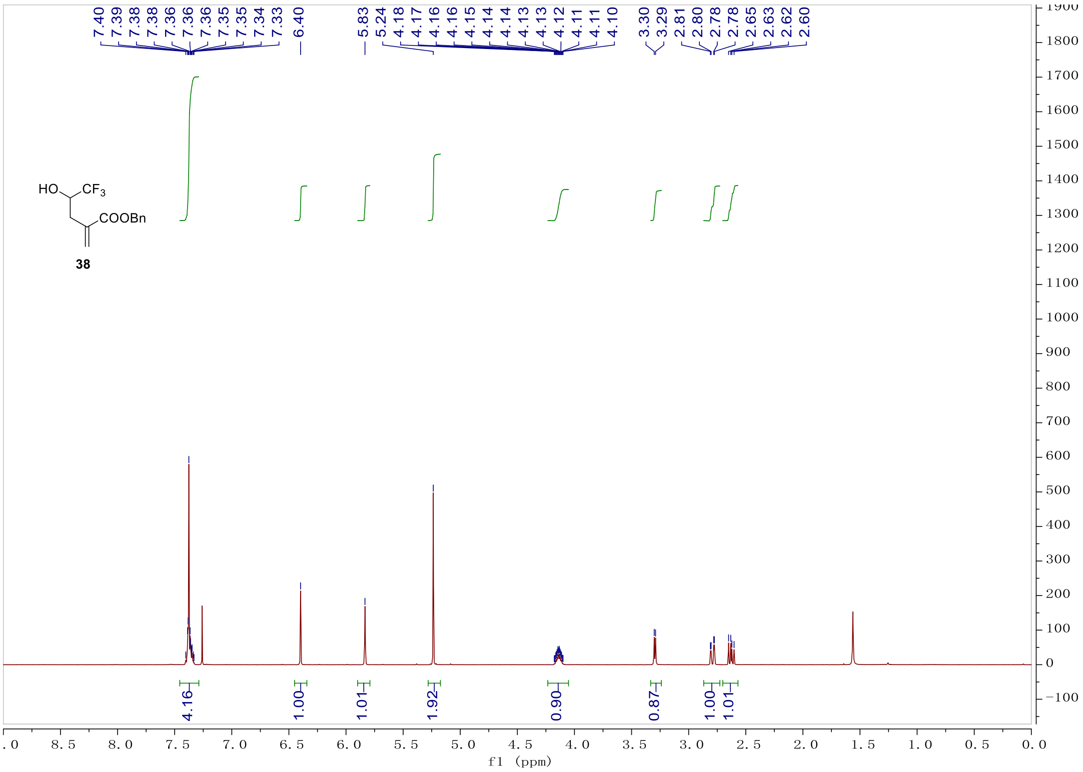

Figure S72. 1H NMR (500 MHz, CDCl3) spectrum of compound 38, related to Scheme 2.

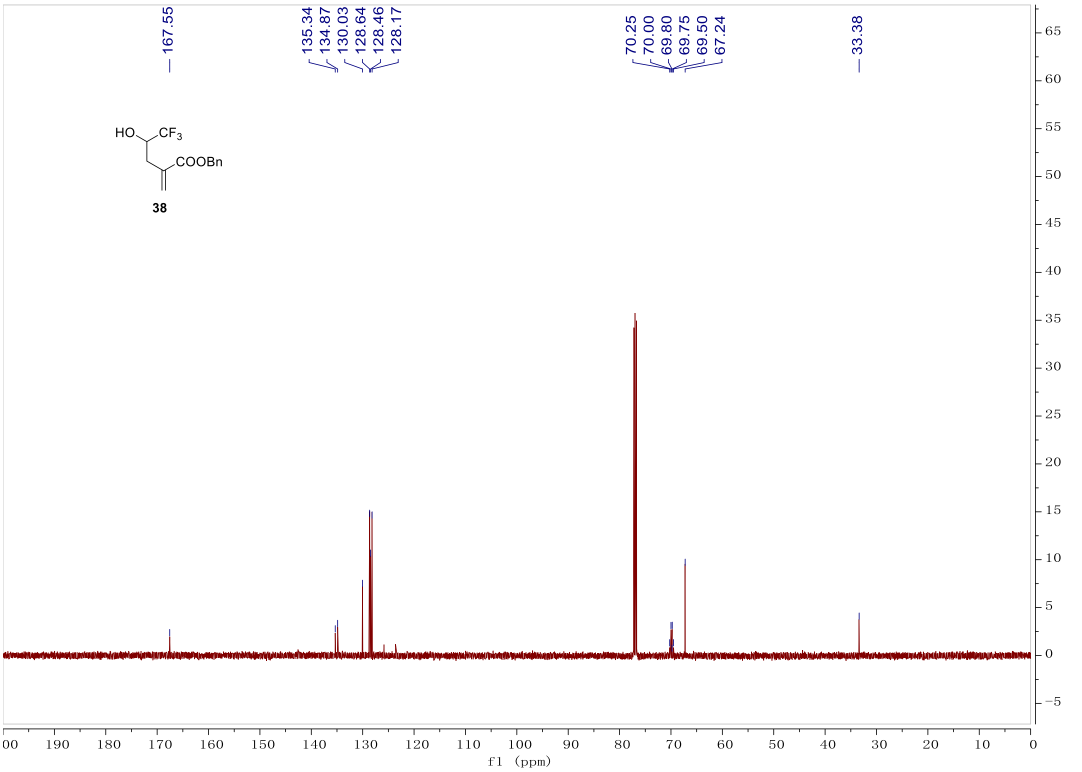

Figure S73. ^{13}C NMR (126 MHz, CDCl3) spectrum of compound 38, related to Scheme 2.

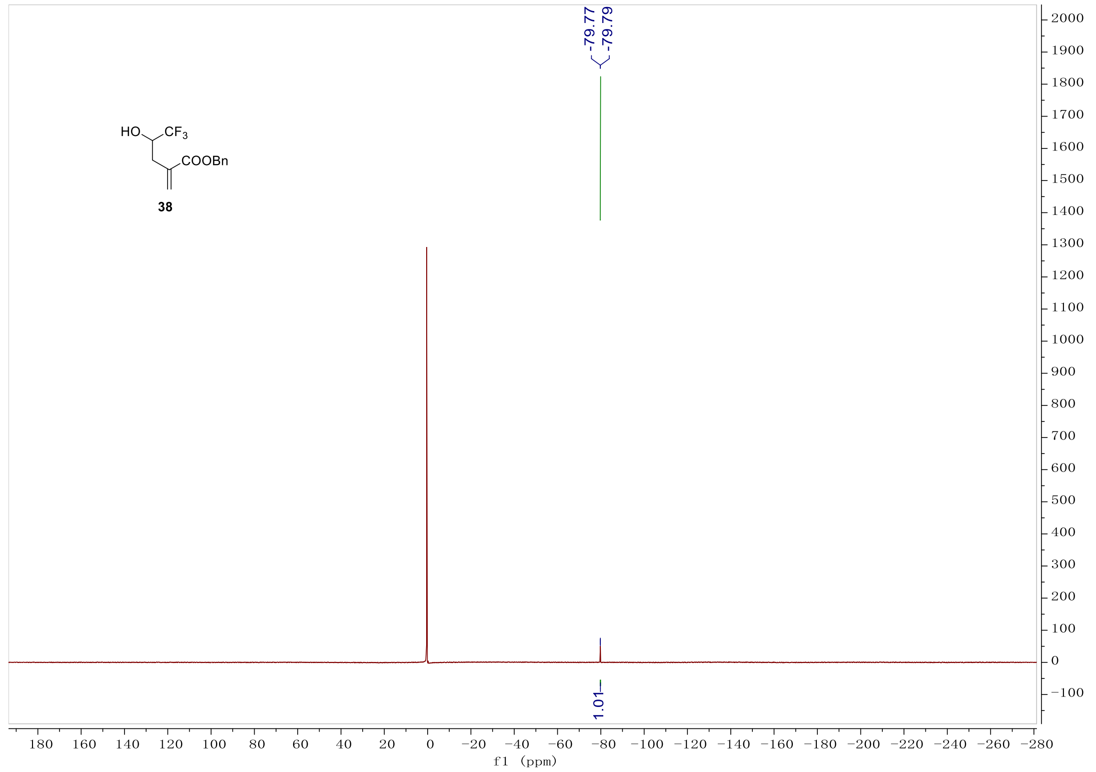

Figure S74. ^{19}F **NMR (376 MHz, CDCl3) spectrum of compound 38, related to Scheme 2**

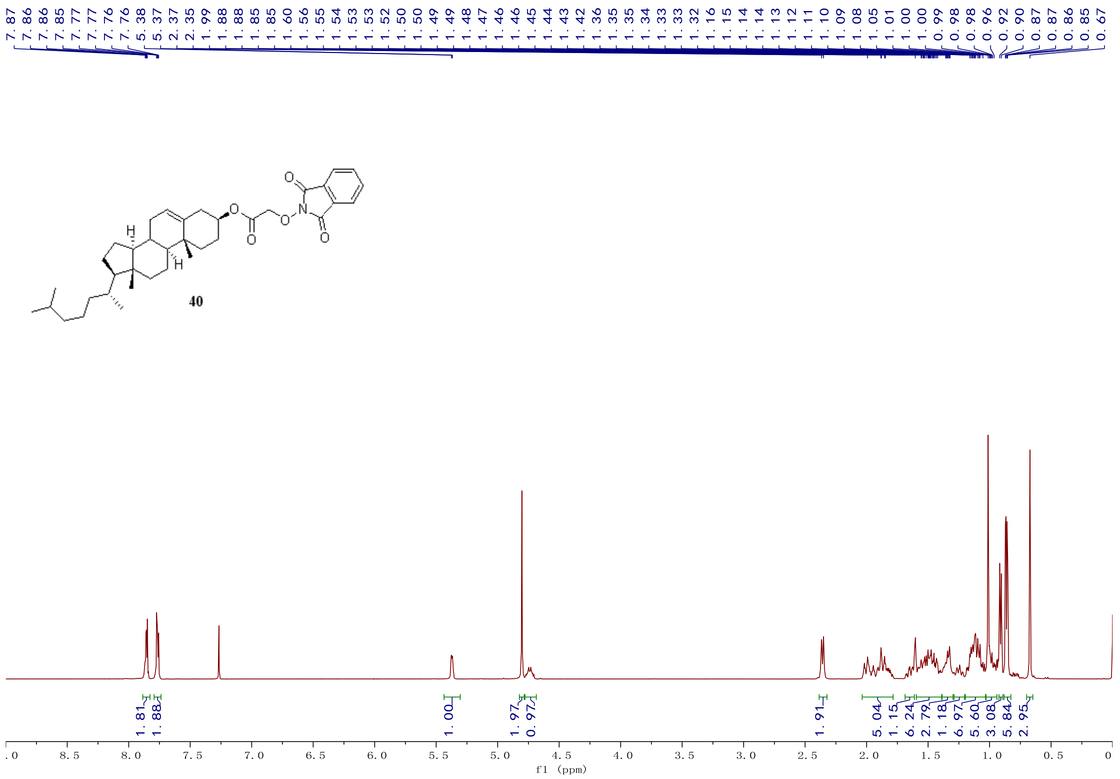

Figure S75. ^{1}H NMR (500 MHz, CDCl3) spectrum of compound 40, related to Scheme 2.

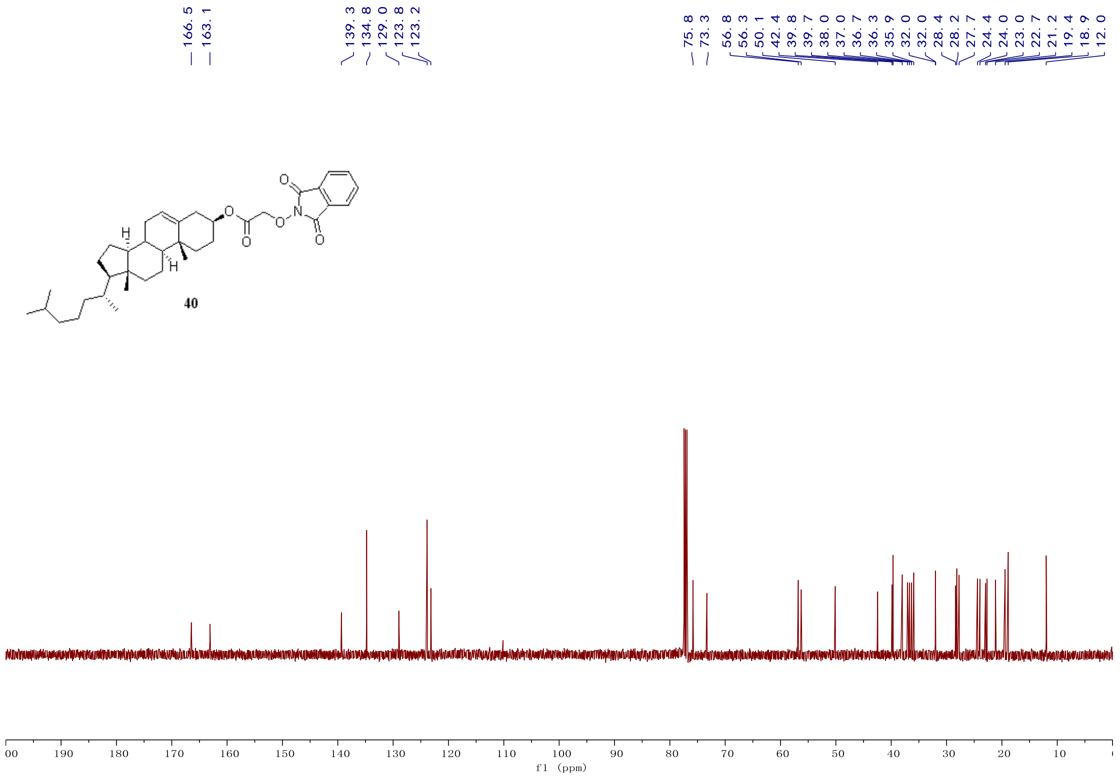

Figure S76. ^{13}C NMR (126 MHz, CDCl3) spectrum of compound 40, related to Scheme 2.

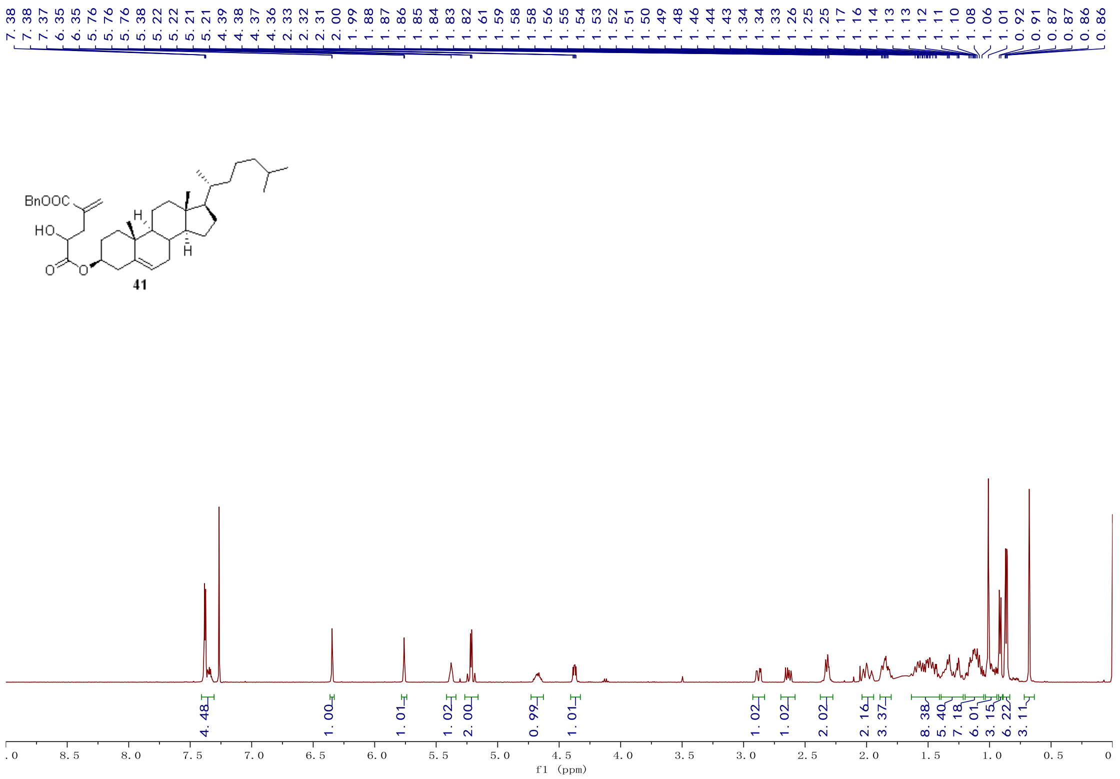

Figure S77. ^{1}H NMR (500 MHz, CDCl3) spectrum of compound 41, related to Scheme 2.

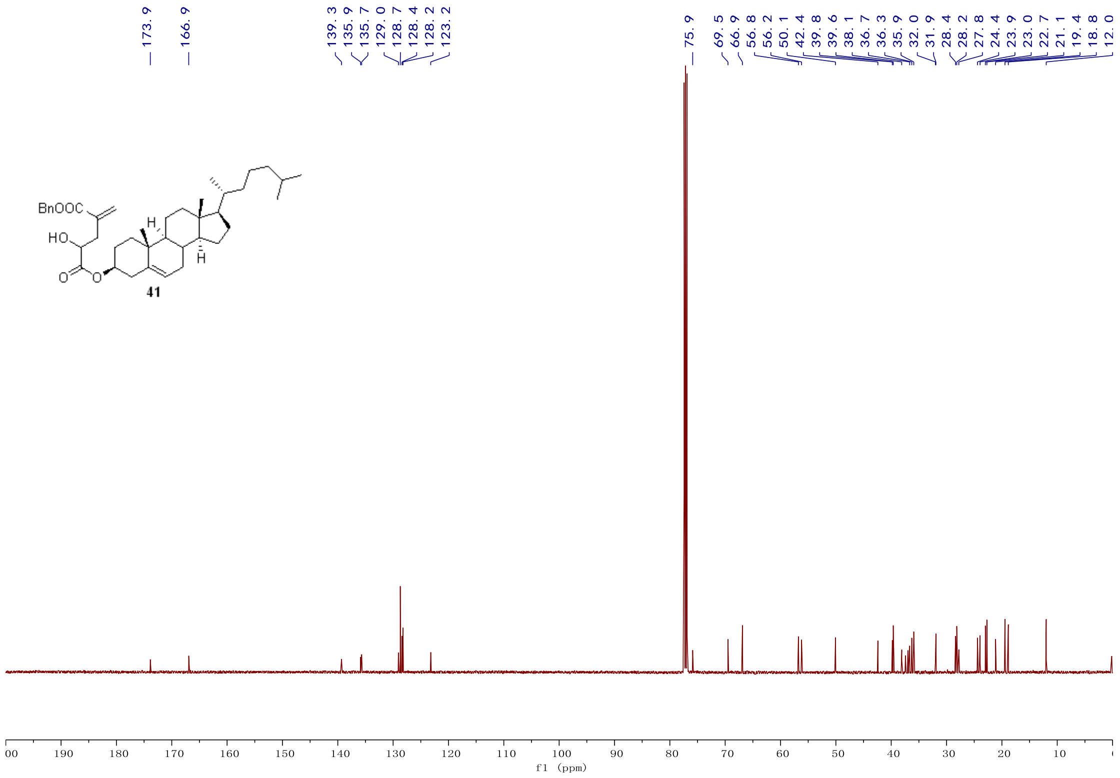

Figure S78. ^{13}C NMR (126 MHz, CDCl3) spectrum of compound 41, related to Scheme 2.

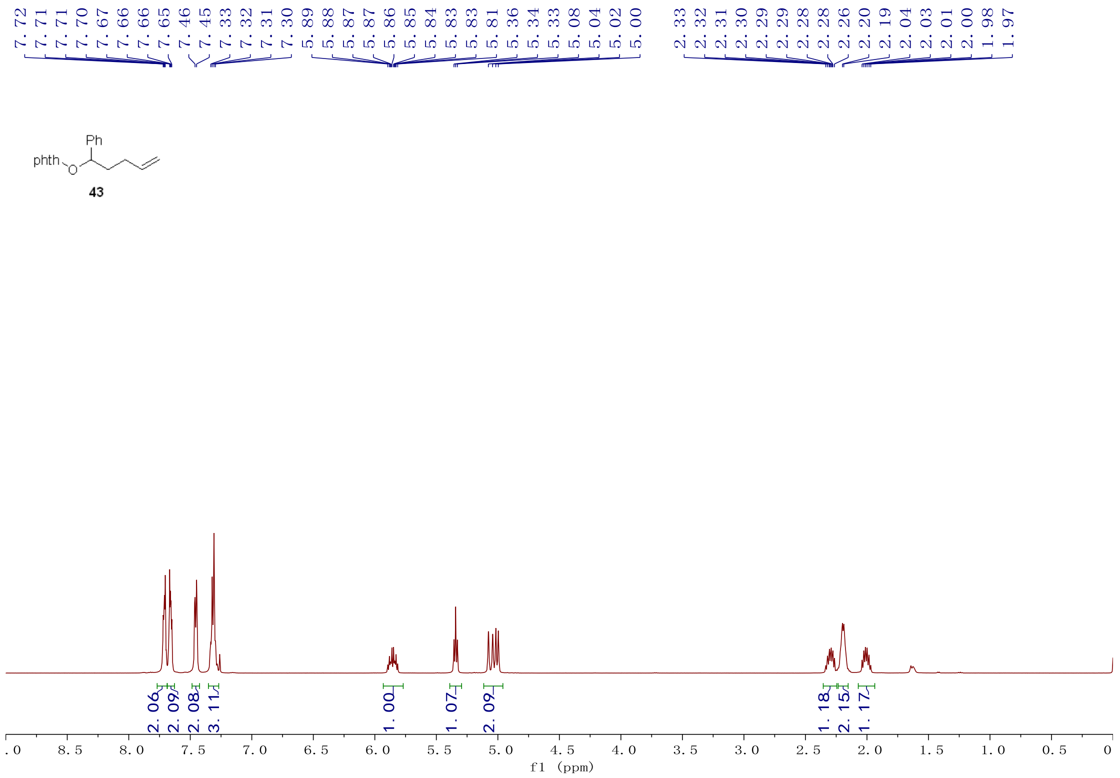

Figure S79. ^{1}H NMR (500 MHz, CDCl3) spectrum of compound 43, related to Scheme 3.

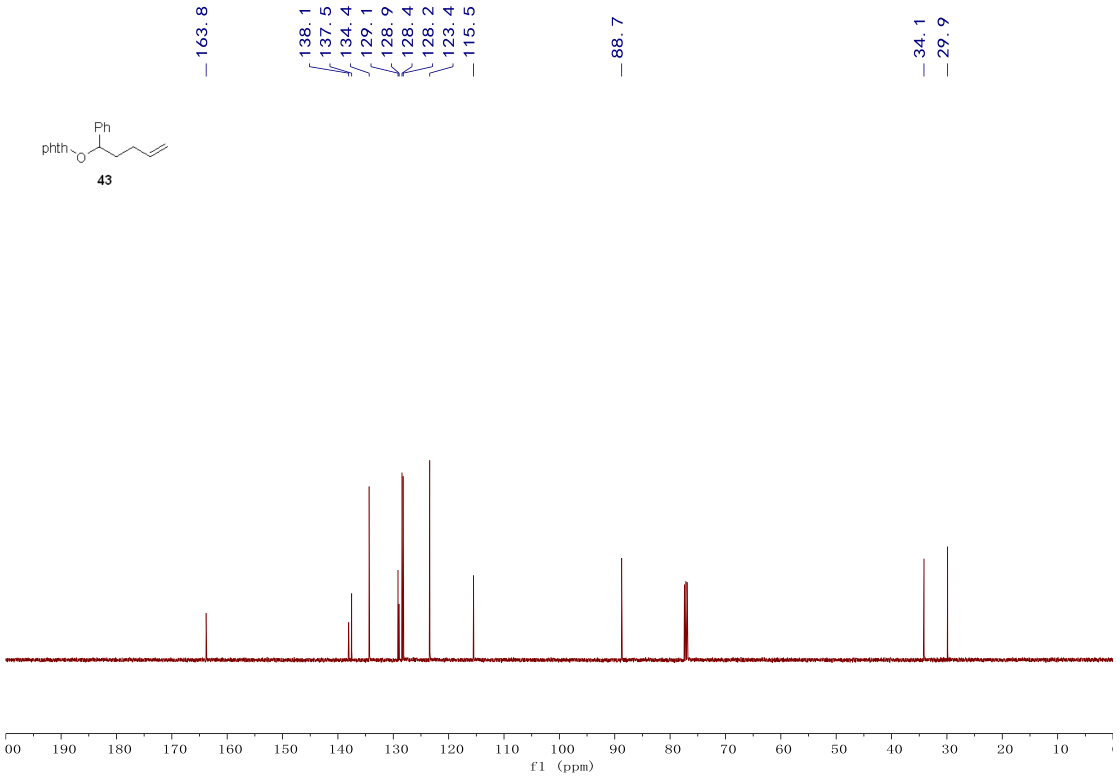

Figure S80. ^{13}C NMR (126 MHz, CDCl3) spectrum of compound 43, related to Scheme 2.

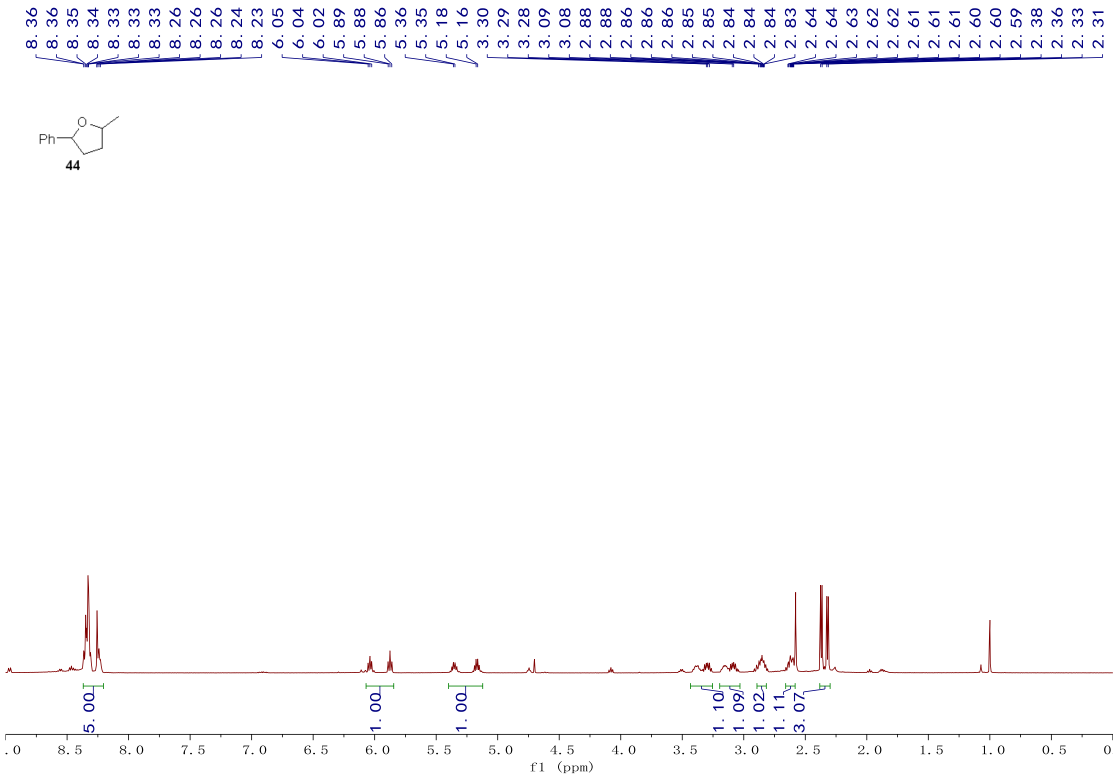

Figure S81. ^{1}**H NMR (500 MHz, CDCl3) spectrum of compound 44, related to Scheme 3.**

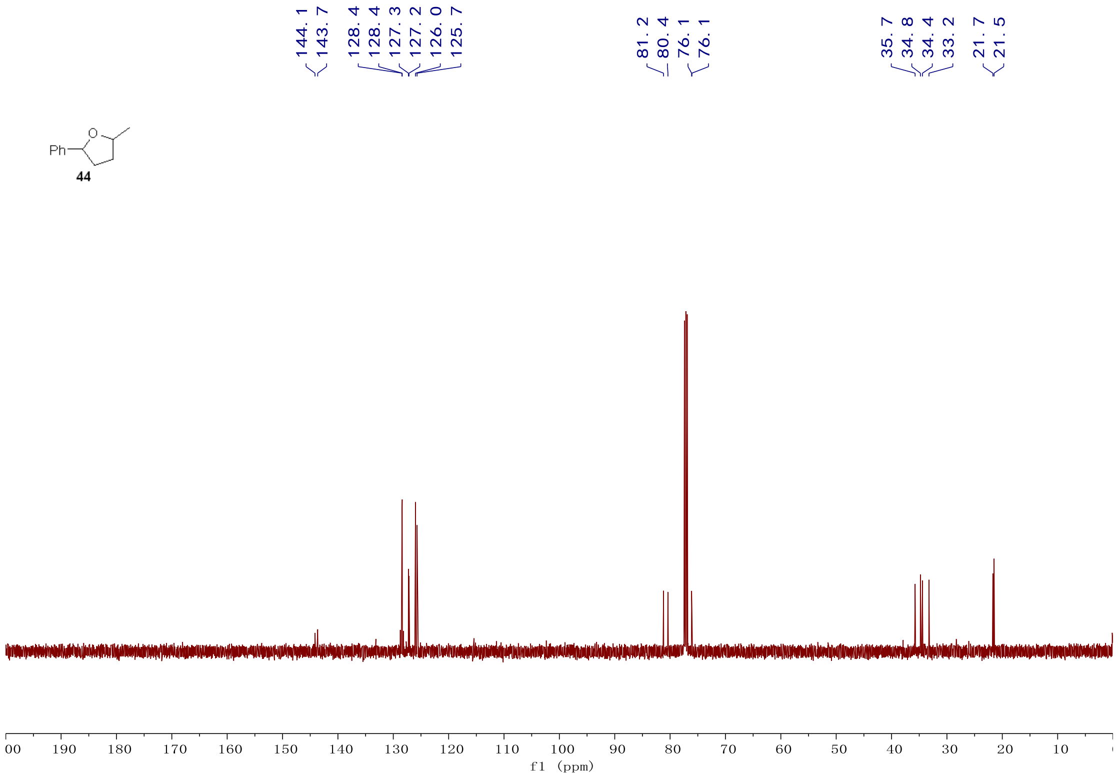

Figure S82. ^{13}**C NMR (126 MHz, CDCl3) spectrum of compound 44, related to Scheme 3.**

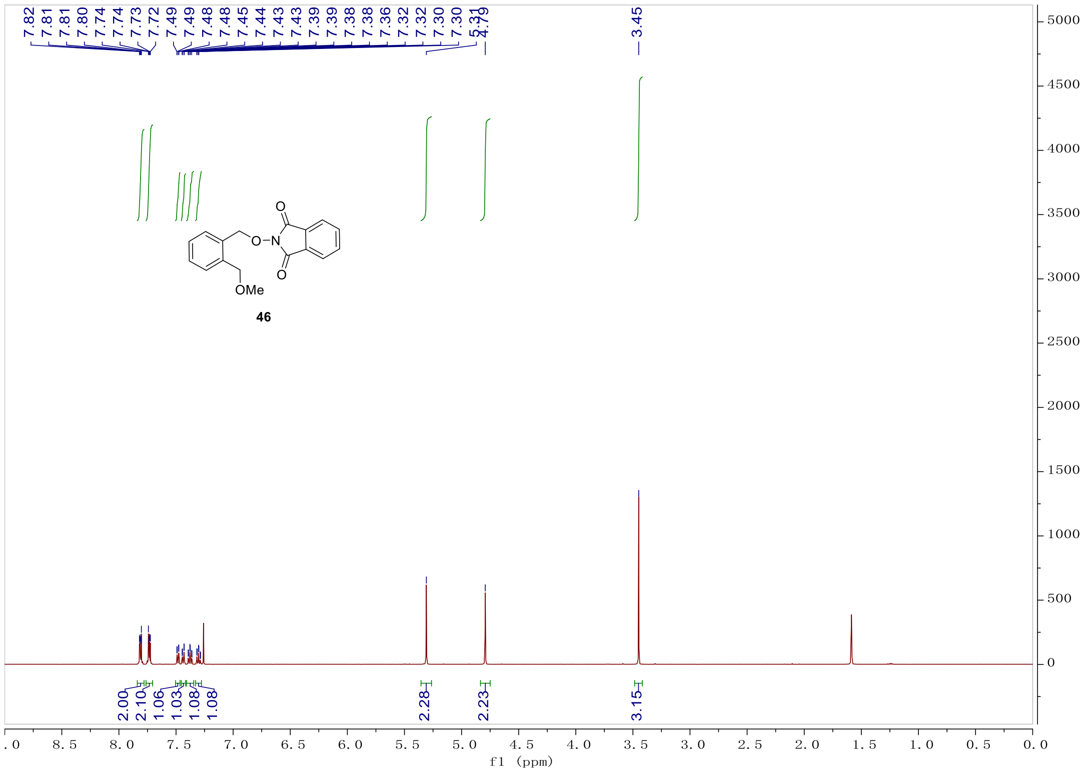

Figure S83. ^{1}H **NMR (500 MHz, CDCl3) spectrum of compound 46, related to Scheme 3.**

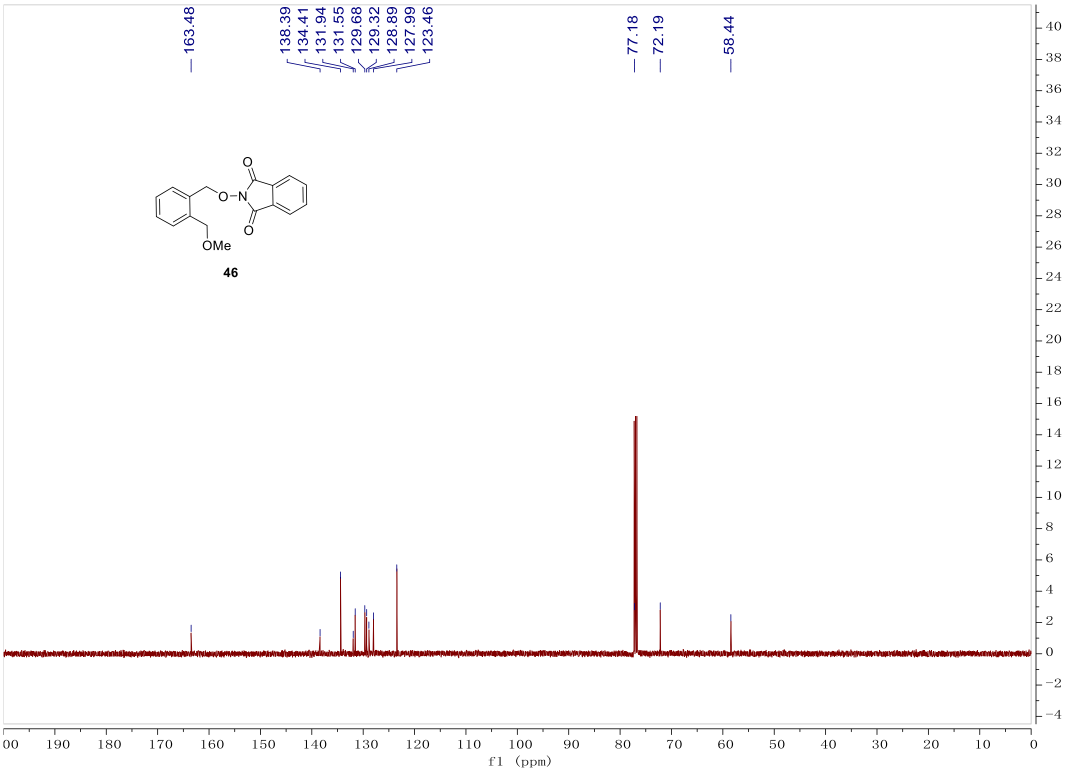

Figure S84. ^{13}C **NMR (126 MHz, CDCl3) spectrum of compound 46, related to Scheme 3.**

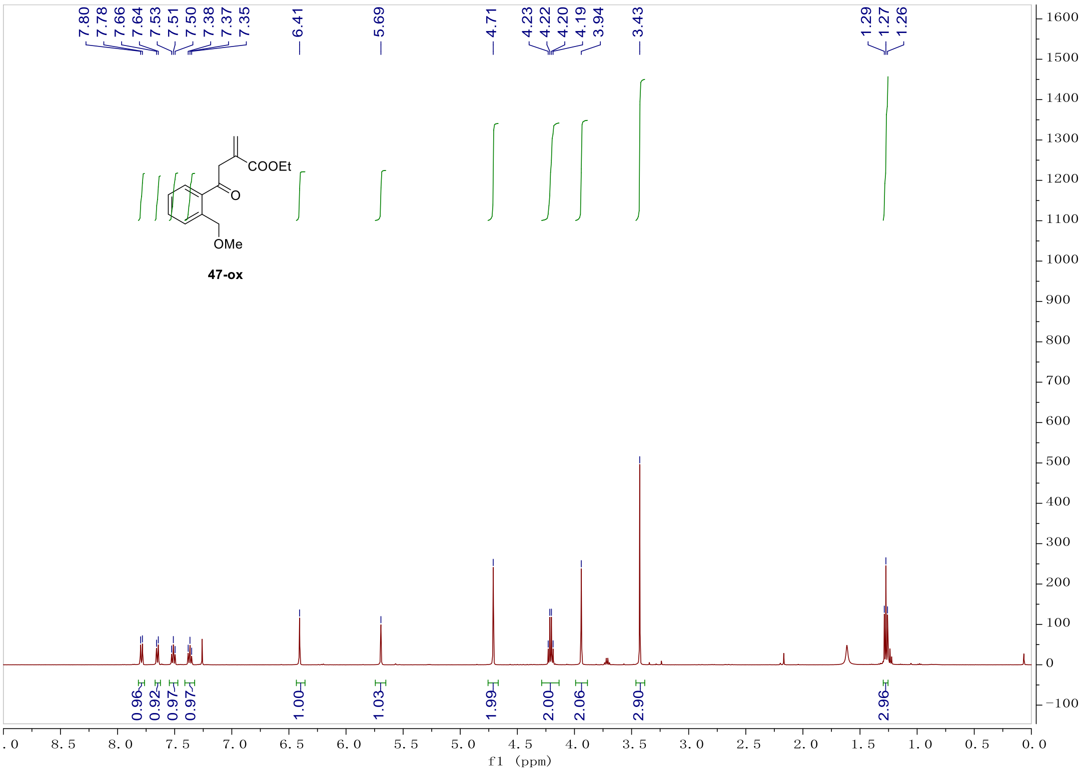

Figure S85. ^{1}H **NMR (500 MHz, CDCl3) spectrum of compound 47-ox, related to Scheme 3.**

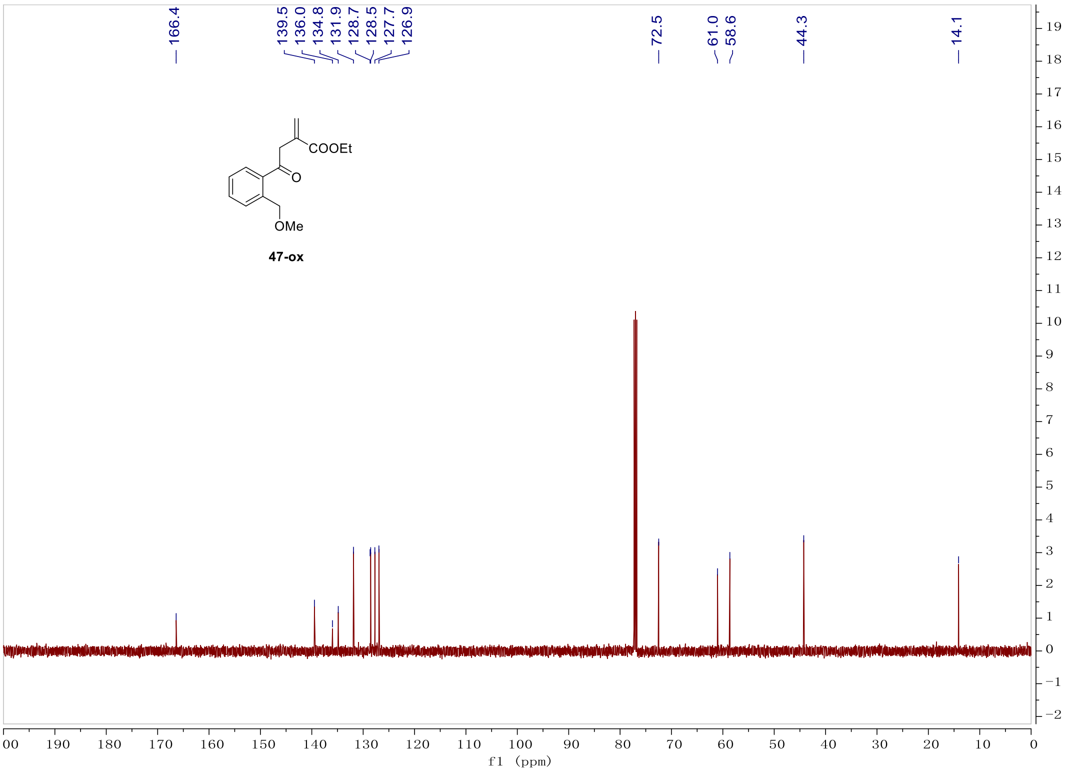

Figure S86. ^{13}C **NMR (126 MHz, CDCl3) spectrum of compound 47-ox, related to Scheme 3.**

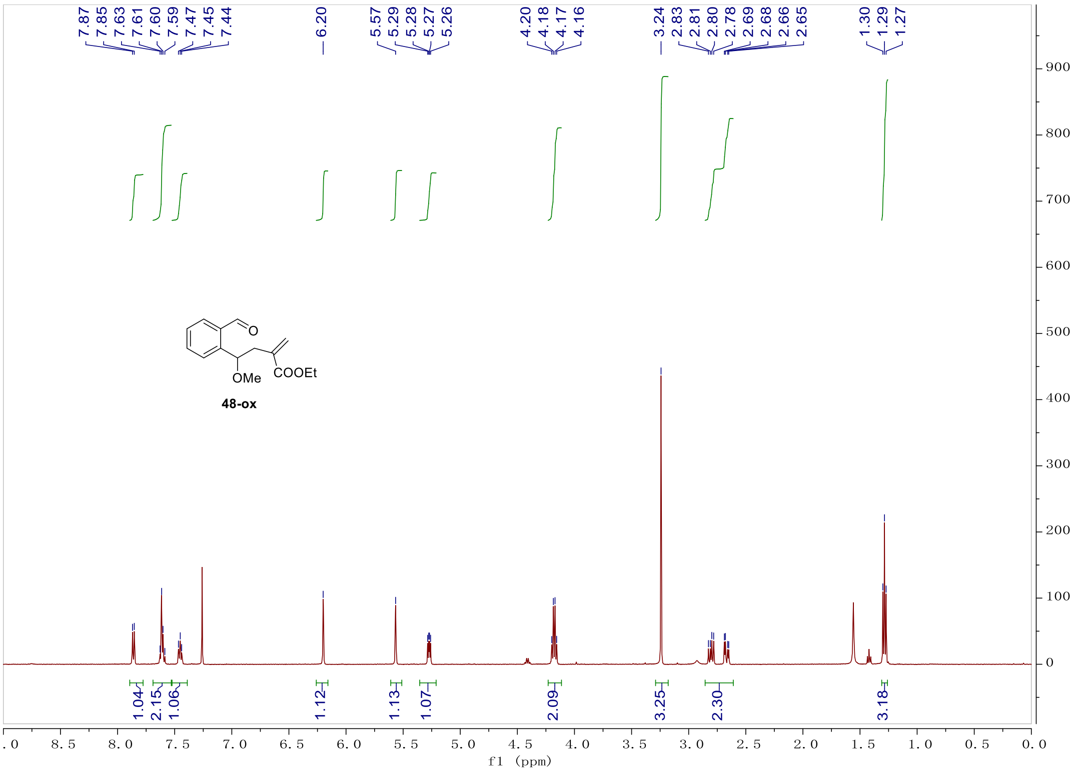

Figure S87. ^{1}**H NMR (500 MHz, CDCl3) spectrum of compound 48-ox, related to Scheme 3.**

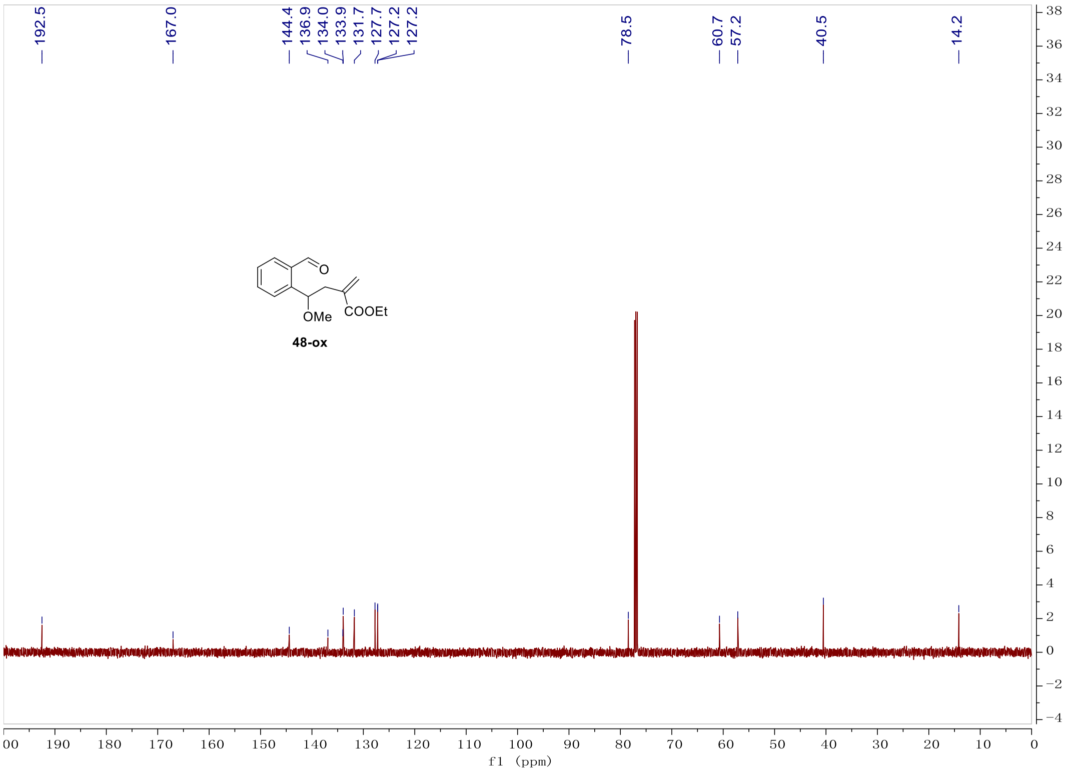

Figure S88. ^{13}**C NMR (126 MHz, CDCl3) spectrum of compound 48-ox, related to Scheme 3.**

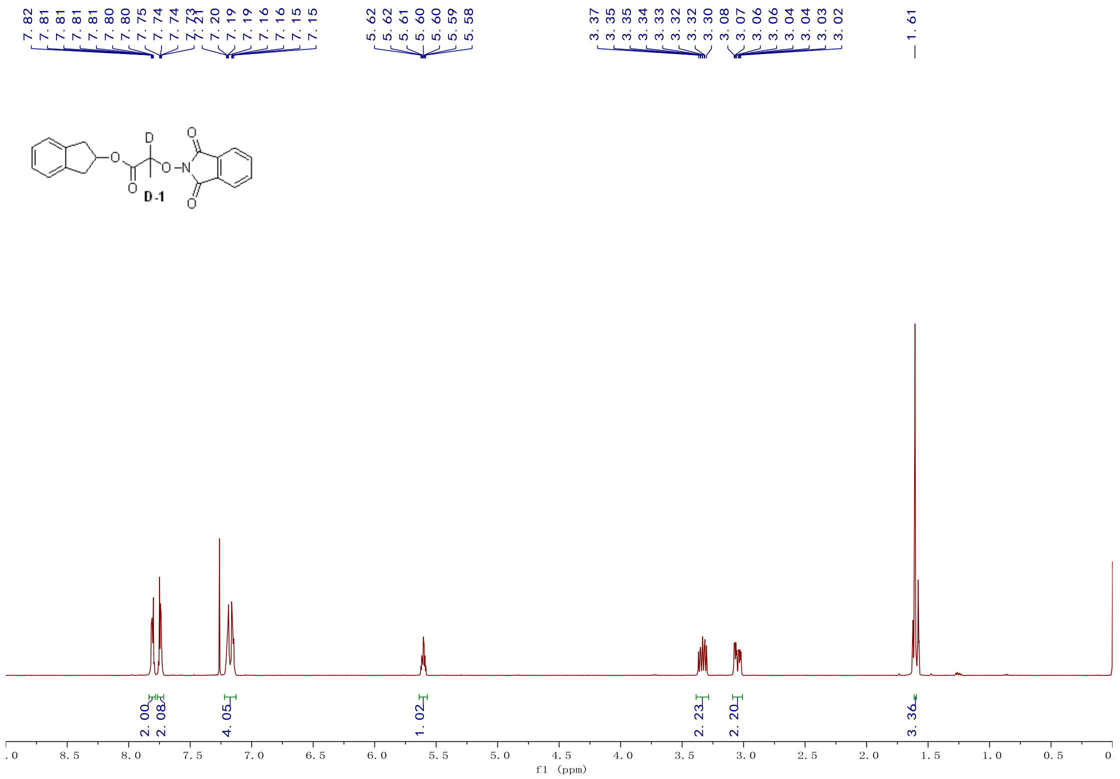

Figure S89. ^{1}H **NMR (500 MHz, CDCl3) spectrum of compound D-1, related to Scheme 3.**

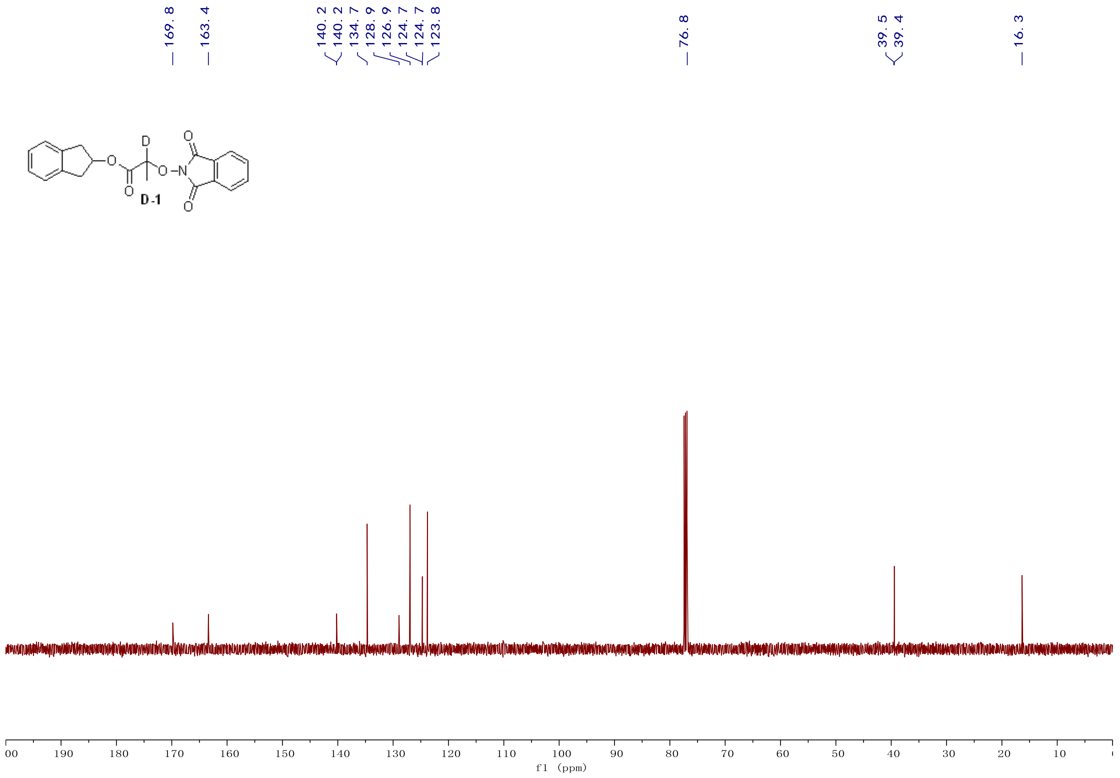

Figure S90. ^{13}C **NMR (126 MHz, CDCl3) spectrum of compound D-1, related to Scheme 3.**

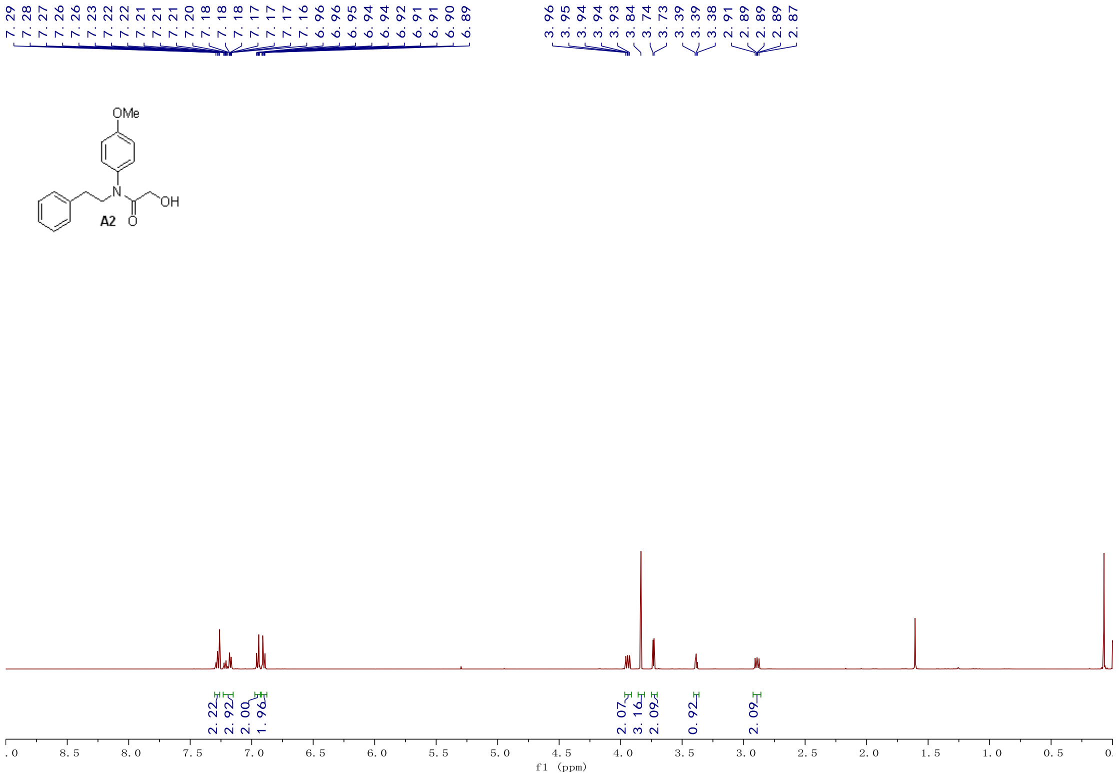

Figure S91. ^{1}H NMR (500 MHz, CDCl3) spectrum of compound A2, related to Scheme 2.

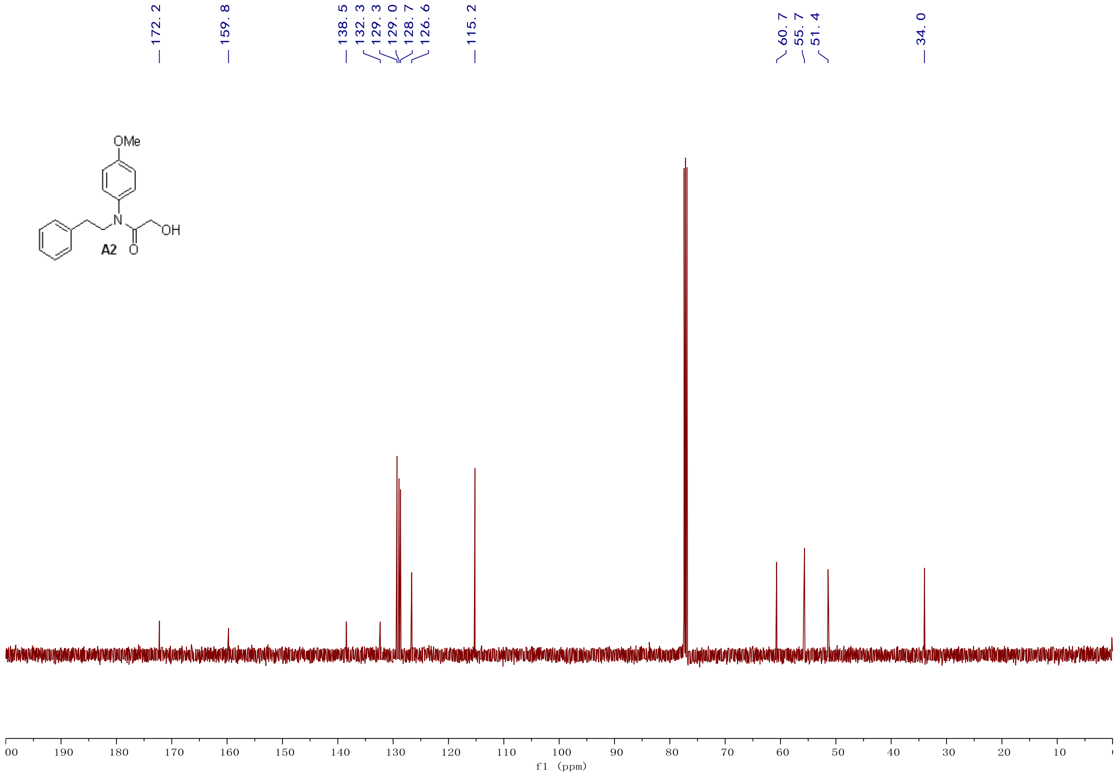

Figure S92. ^{13}C NMR (126 MHz, CDCl3) spectrum of compound A2, related to Scheme 2.

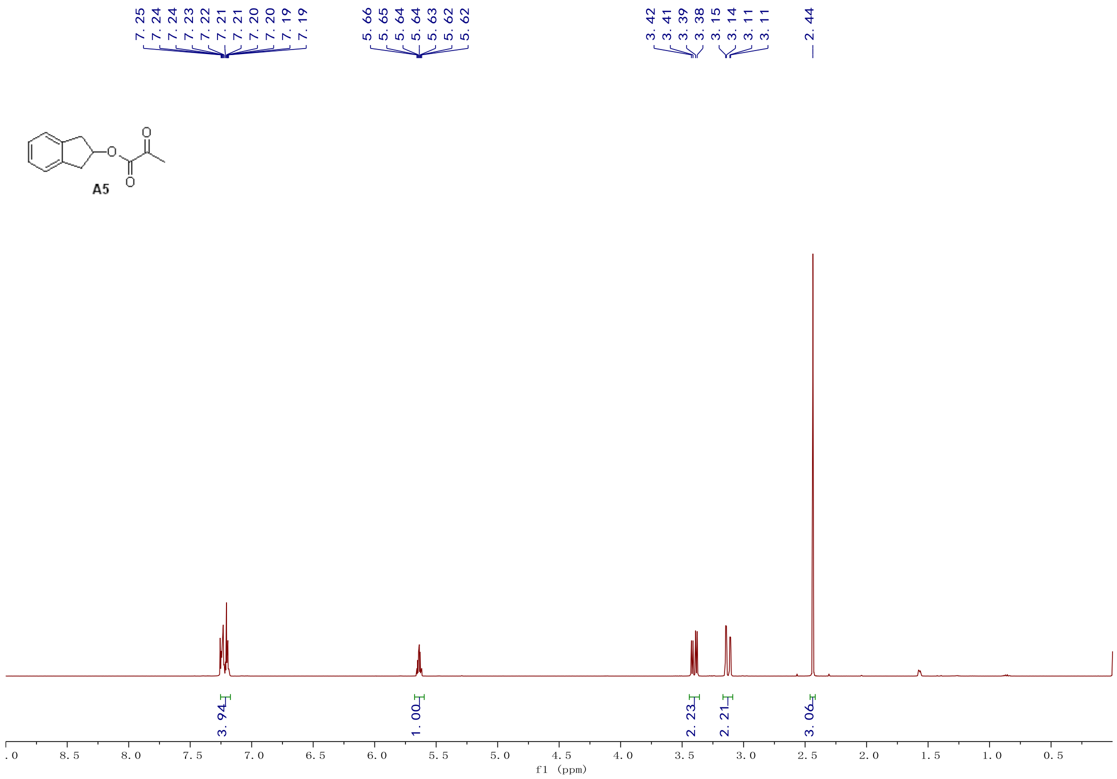

Figure S93. ^{1}H **NMR (500 MHz, CDCl3) spectrum of compound A5, related to Scheme 2.**

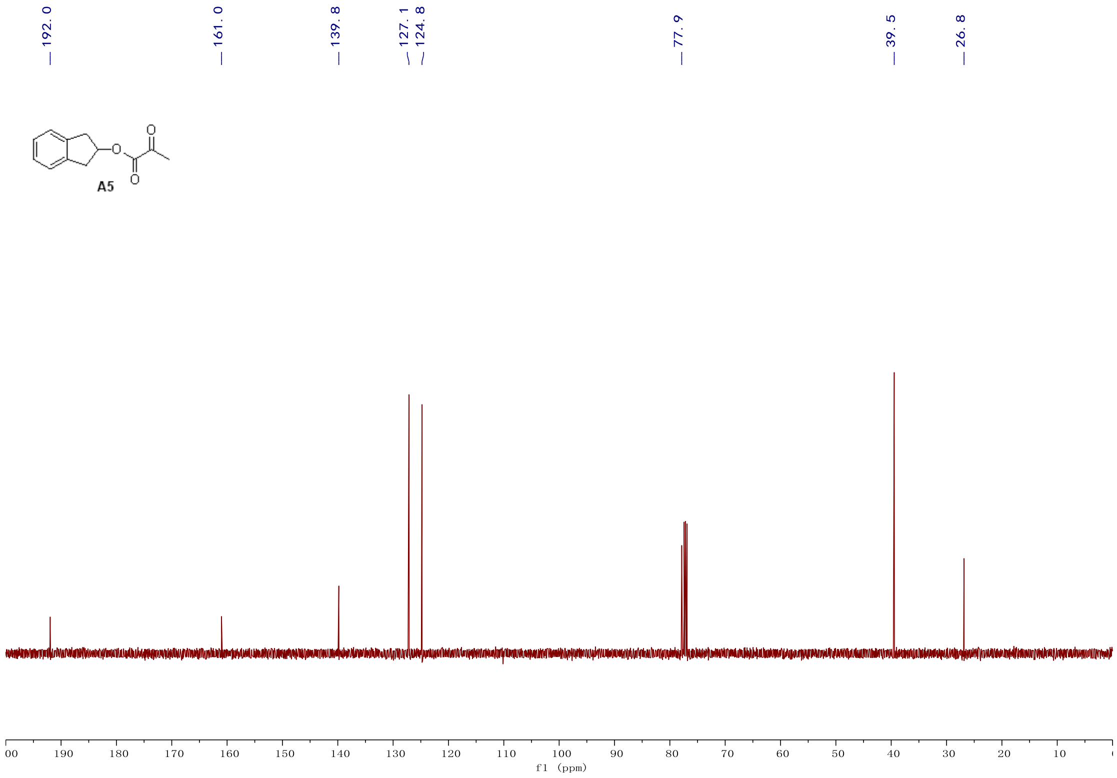

Figure S94. ^{13}C **NMR (126 MHz, CDCl3) spectrum of compound A5, related to Scheme 2.**

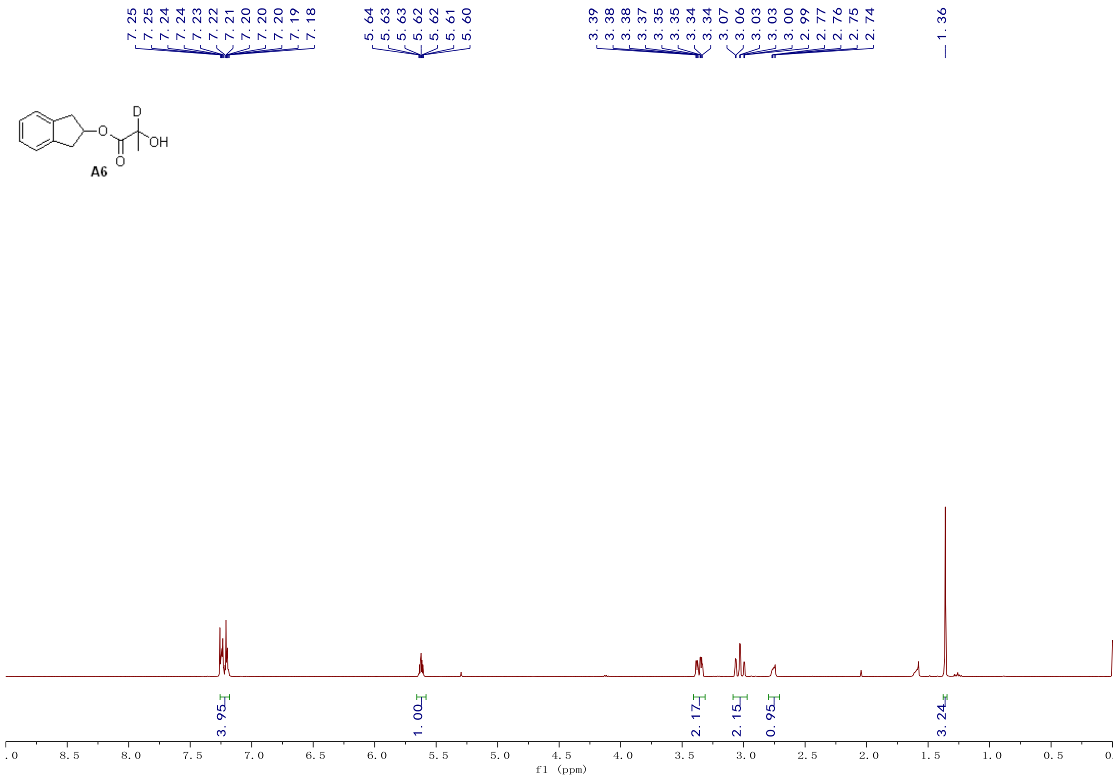

Figure S95. ^{1}H NMR (500 MHz, CDCl3) spectrum of compound A6, related to Scheme 3.

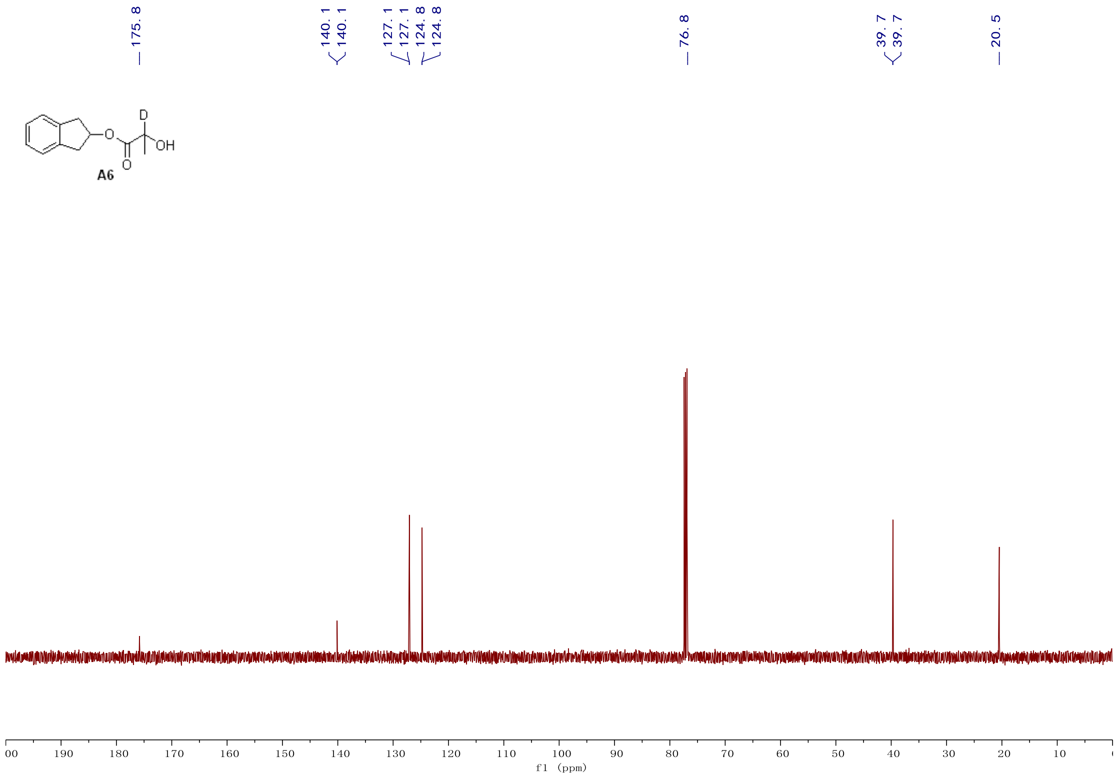

Figure S96. ^{13}C NMR (126 MHz, CDCl3) spectrum of compound A6, related to Scheme 3.

II. Mechanistic Investigations

The Cross-Over Experiment

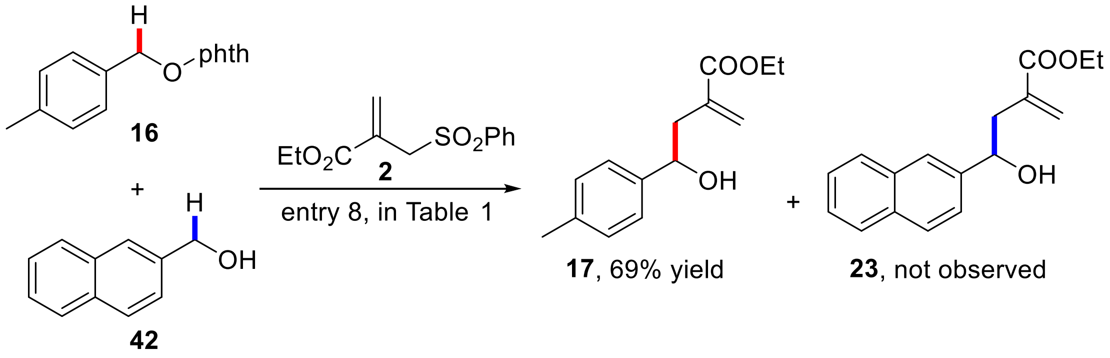

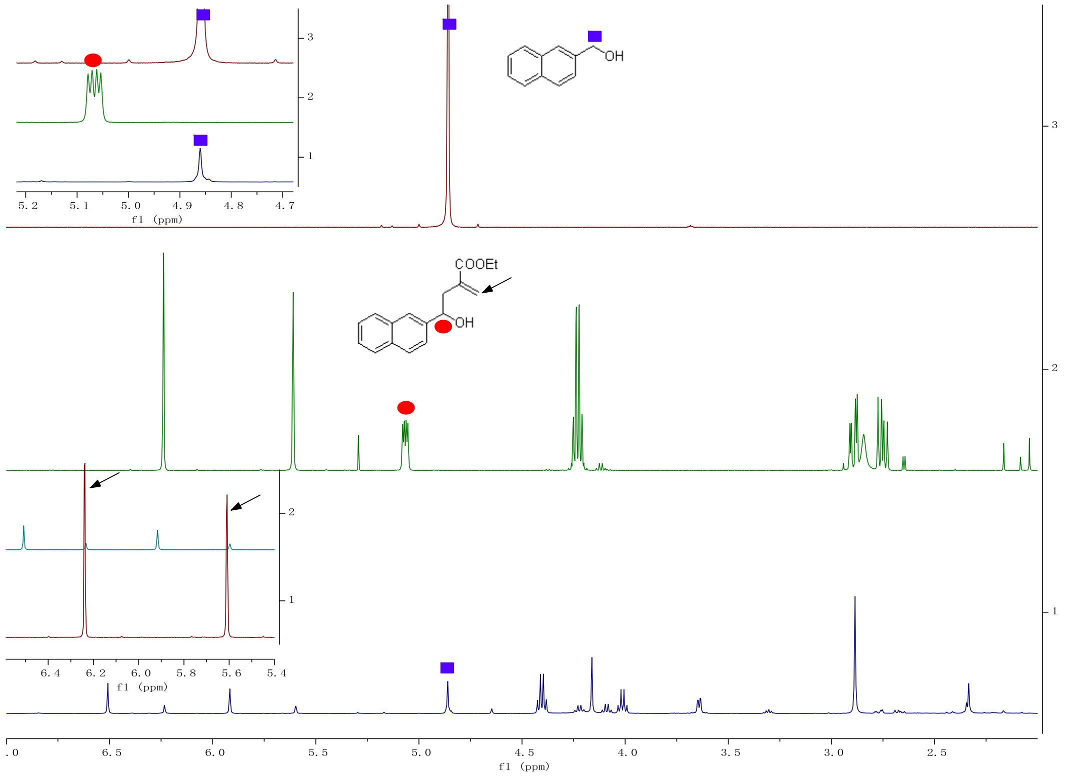

Scheme S1. The NMR spectra of the Cross-Over Experiment, Related to Scheme 3.

The allylation adduct **17** in 69% yield and no occurrence of **23** from crude NMR spectra.

KIE experiments

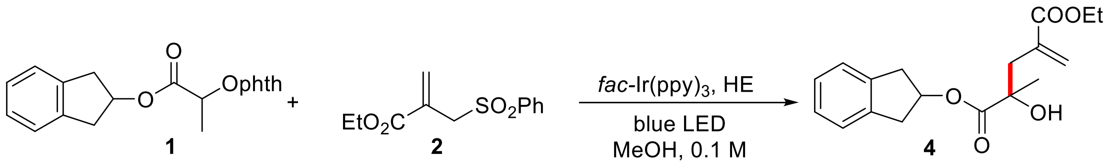

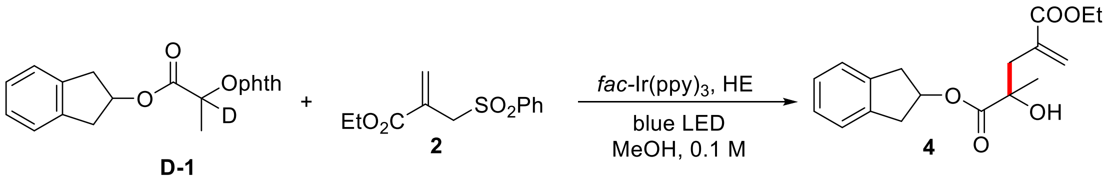

Reaction Time (min)	Yield of Sub **1** (%)	Yied of Sub **D-1** (%)
10	9	9
20	12	18
30	19	24
40	26	27

Table S1. The NMR Yield of the KIE Experiment, Related to Scheme 3.

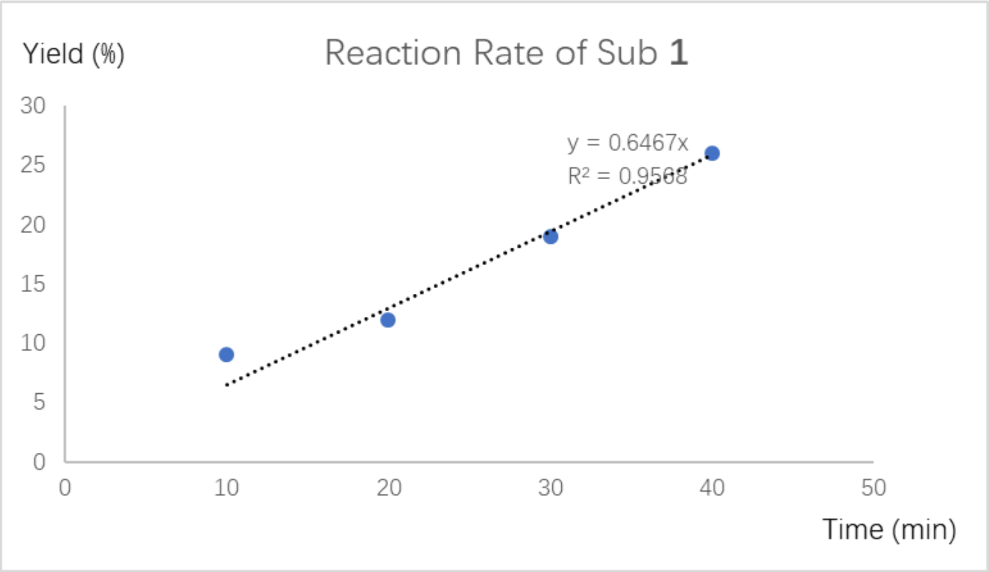

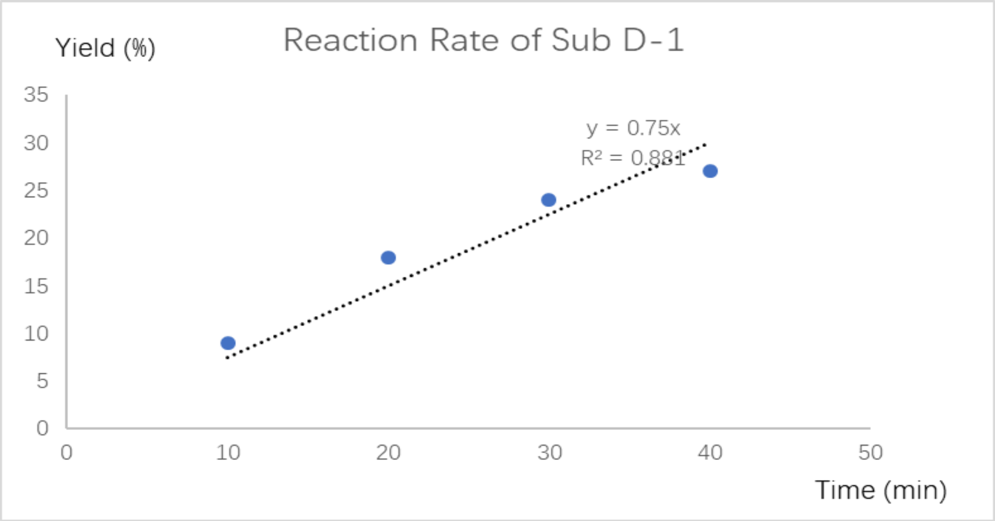

Scheme S2. Deuterium labeling experimtns, Related to Scheme 3.

The KIE value was calculated as K_H/K_D = 0.65/ 0.75 = 0.87, suggesting that the cleavage of the α-C(sp^3)-H bond was not the rate determining step.

1,2-HAT Competes with Other Alkoxyl Radical Reaction Pathways

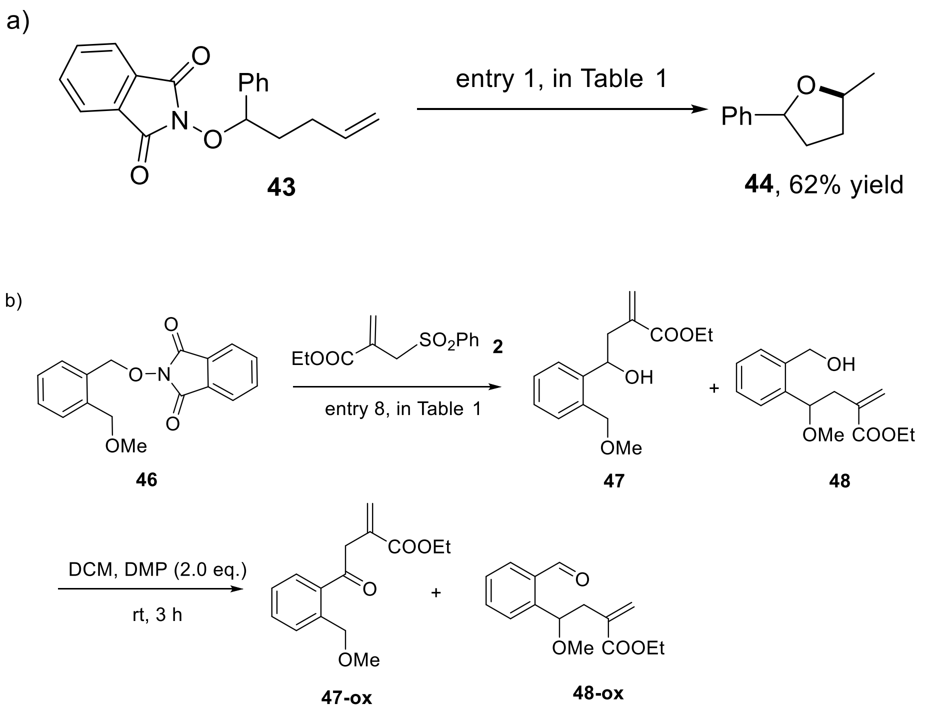

entry	conditions	conversion	NMR yield of 47	NMR yield of 48
**1**	Entry 8, in table 1	> 95 %	37%	14 %
**2**	Entry 1, in table 1	> 95 %	28 %	53 %

Scheme S3. 1,2-HAT Competes with 1,5-HAT, Related to Scheme 3.

Luminescence Quenching Experiments

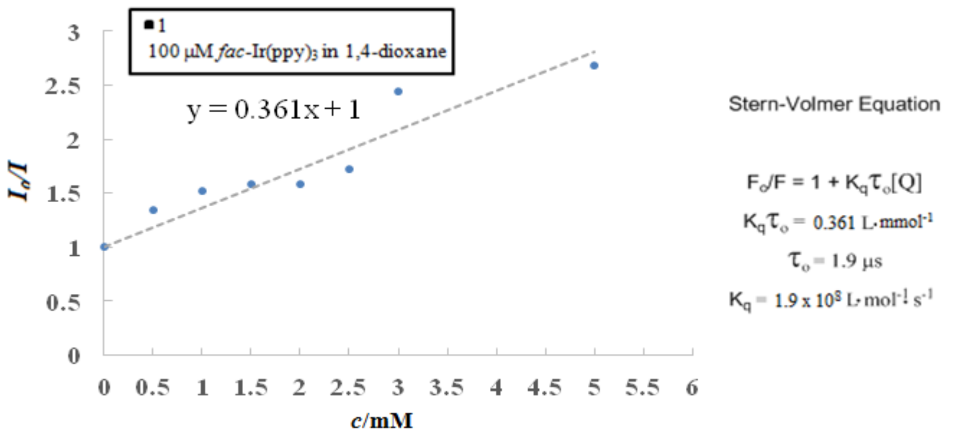

Scheme S4. ***fac*-Ir(ppy)$_3$ Emission Quenching by *N*-alkoxyphthalimide 1, Related to Scheme 6.**

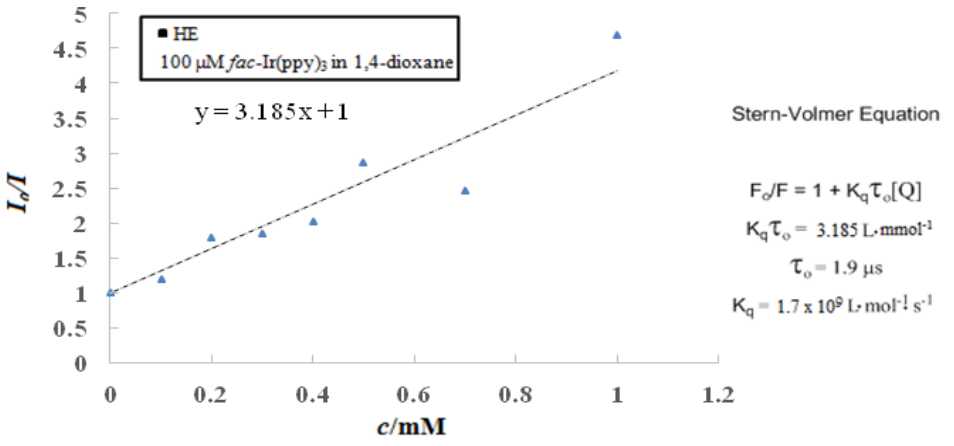

Scheme S5. ***fac*-Ir(ppy)$_3$ Emission Quenching by Hanztsch Ester (HE), Related to Scheme 6.**

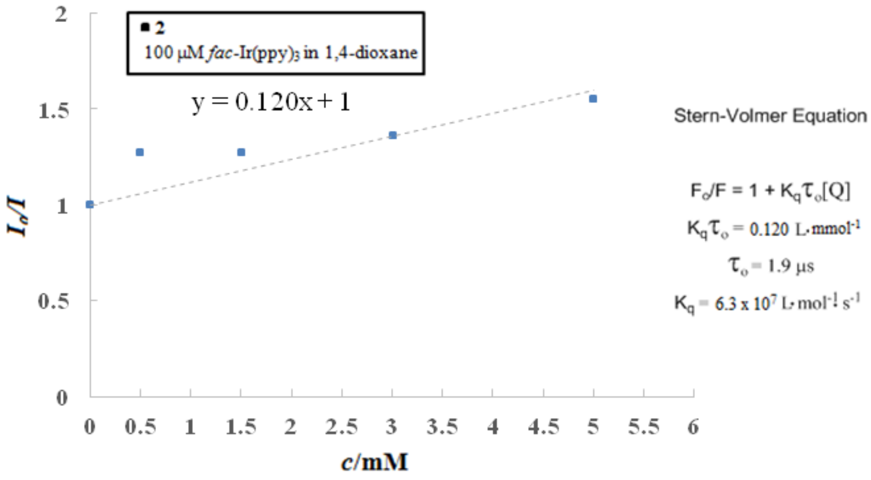

Stern-Volmer Equation

$F_o/F = 1 + K_q\tau_o[Q]$

$K_q\tau_o = 0.120\ L\cdot mmol^{-1}$

$\tau_o = 1.9\ \mu s$

$K_q = 6.3 \times 10^7\ L\cdot mol^{-1}\ s^{-1}$

Scheme S6. *fac*-Ir(ppy)3 Emission Quenching by Allyl Sulfone 2, Related to Scheme 6.

Cyclic Voltammetry Data

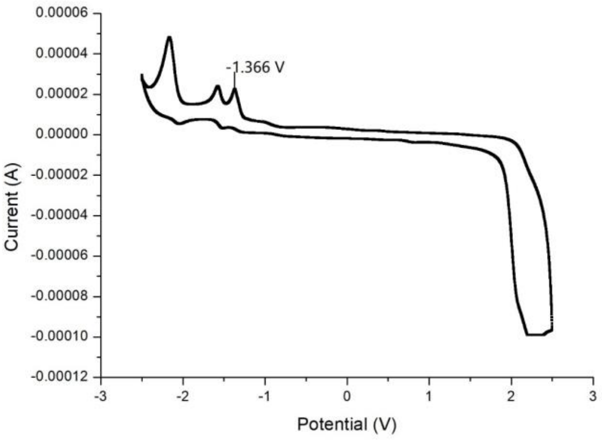

Scheme S7. Cyclic Voltammogram of *N*-alkoxyphthalimide 1, Related to Scheme 6.

$E_{1/2}^{red}$ (1) = -1.37 V vs. SCE in CH_3CN

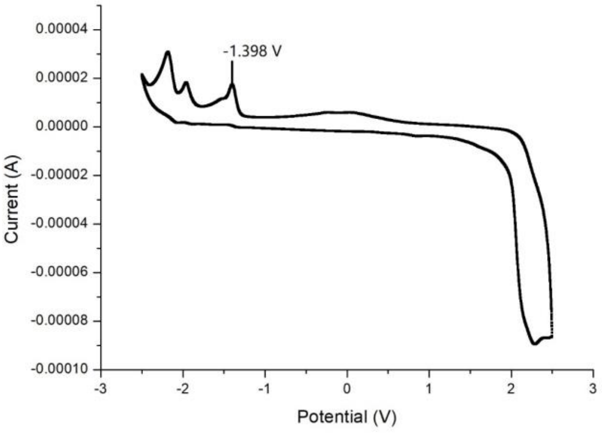

Scheme S8. Cyclic Voltammogram of *N*-alkoxyphthalimide 16, Related to Scheme 6.

$E_{1/2}^{red}$ (1) = -1.40 V vs. SCE in CH_3CN

The Quantum Yield Measurement

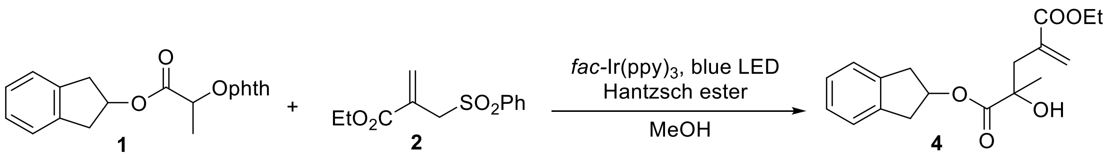

$$\Phi = \frac{N_{\mathrm{prod}}}{N_{abs\text{'}\,\mathrm{photon}}} = \frac{n_{\mathrm{x}}}{n_{p}}$$

Scheme S9. The Quantum Yield Measurement, Related to Scheme 6.
The quantum yield is calculated to be 1.96.

The On-Off-Light Experiments

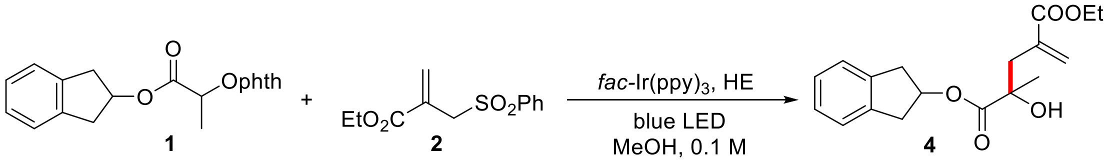

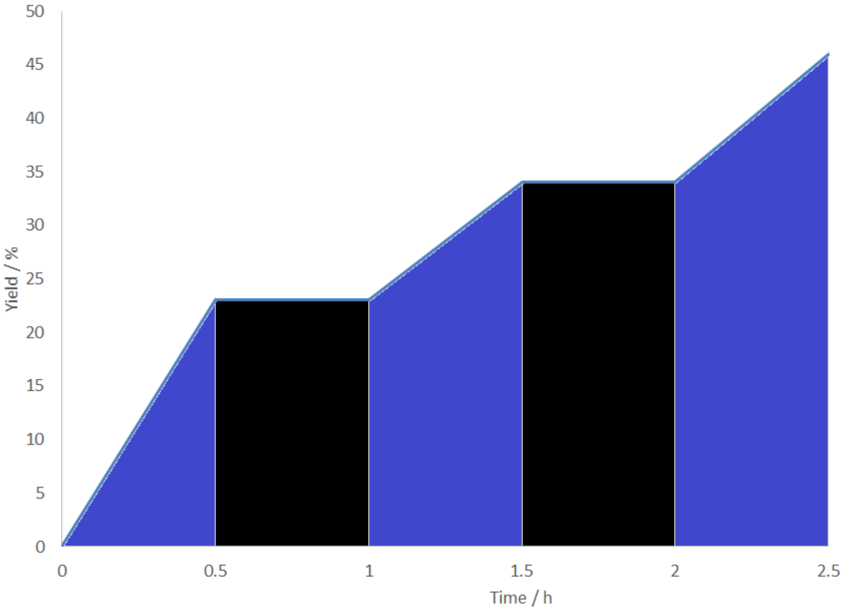

Scheme S10. The On-off-light Experiments, Related to Scheme 6.

The results suggest the radical chain may exist, however the chain length is short.

Detailed Reaction Optimizations

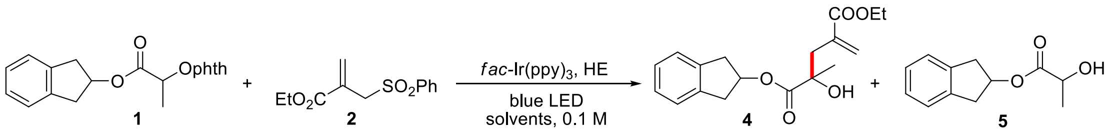

entry	conditions[a]	conversion	**4** yield (%)[b]	**5** yield (%)[b]
1	HE, 1,4-dioxane	>95%	41%	52%
2	entry 1, 0.05 M	>95%	36%	60%
3	entry 1, 0.2 M	>95%	49%	48%
4	entry 1, CH_3CN	>95%	37%	56%
5	entry 1, DCM	>95%	60%	33%
6	entry 1, EtOH	>95%	93%	7%
7	entry 1, MeOH	>95%	97%	<5%
8	entry 1, $CHCl_3$	>95%	66%	25%
9	entry 7, 0.05 M	>95%	89%	11%

[a]Reaction conditions: 1 (0.10 mmol, 1.0 equiv.), 2 (0.30 mmol, 3.0 equiv.), *fac*-Ir(ppy)$_3$ (0.001 mmol, 1%) and Hantzsch ester (0.15 mmol, 1.5 equiv.) in 1.0 mL solvent under nitrogen with 4 W blue LED irradiation at ambient temperature for 3 h, conversion was >95%, unless otherwise noted. [b]Conversion and yields were determined by ^{1}H NMR analysis and isolated yields were in parentheses. [c]Hantzsch ester (HE)

Table S2. Detailed Reaction Optimizations, Related to Table 1.

The Effect of Water Addition for the Reaction

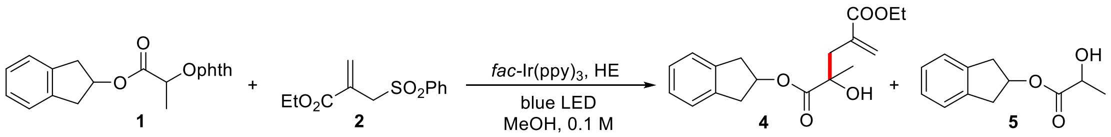

entry	conditions	conversion[b]	**4** (%)[b]	**5** (%)[b]
1	ultra dry MeOH	>95%	>95%	<5%
2	ultra dry MeOH : H_2O = 9:1	>95%	93%	<5%
3	ultra dry MeOH : H_2O = 1:1	>95%	89%	9%
4	ultra dry 1,4-dioxane	>95%	29%	67%
5	ultra dry 1,4-dioxane : H_2O = 9:1	>95%	66%	33%
6	ultra dry 1,4-dioxane : H_2O = 1:1	>95%	76%	11%

[a]Reaction conditions: **1** (0.10 mmol), **2** (0.30 mmol), HE (0.15 mmol) in 1.0 mL solvents under nitrogen with 4 W blue LED irradiation at ambient temperature. [b]Conversions and yields were determined by ^{1}H NMR analysis.

Table S3. The Effect of Water Addition, Related to Table 1.

EPR studies

The ketyl radical addition adduct **52** was found in MeOH. Instrumental parameters: ν = 9.37 GHz, modulation frequency = 100 kHz, modulation amplitude = 1.00 G, microwave power = 7.96 mW, conversion time = 58.59 ms, time constant = 0 ms, sweep time = 60 s. The hyperfine coupling constants determined after simulation correspond to an adduct DMPO-CH (a_N = 14.9 G and $a_{H\beta}$ = 21.1 G, g = 2.00538). The simulated EPR signals were obtained by Xepr software.

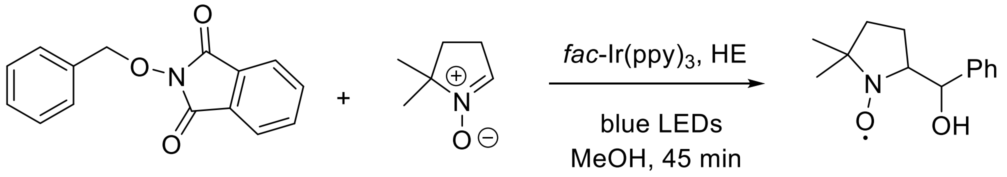

49 **50** **52**

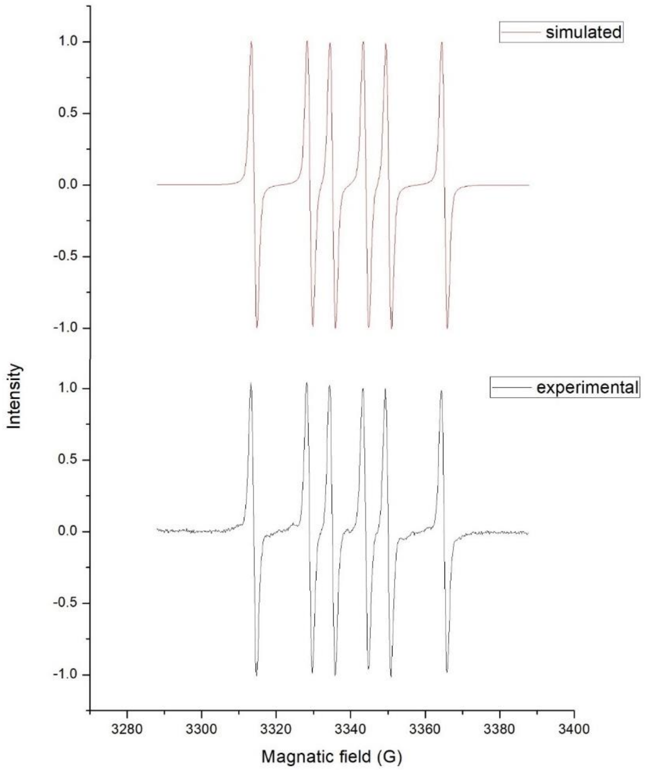

Scheme S11. EPR Spectrum of Spin Adducts in MeOH, Related to Scheme 4.

The alkoxyl radical and the ketyl radical addition adducts **51** and **52** were found in dioxane. Instrumental parameters: ν = 9.37 GHz, modulation frequency = 100 kHz, modulation amplitude = 1.00 G, microwave power = 0.50 mW, conversion time = 58.59 ms, time constant = 0 ms, sweep time = 60 s. The hyperfine coupling constants determined after simulation correspond to a mixture of adduct DMPO-OCH (a_N = 14.1 G and $a_{H\beta}$ = 9.6 G, g = 2.00585) and adduct DMPO-CH (a_N = 14.2 G, and $a_{H\beta}$ = 19.7 G, g = 2.00573). The asterisks peaks are assigned to the alkoxyl radical addition adduct **47**. The simulated EPR signals were obtained by Xepr software.

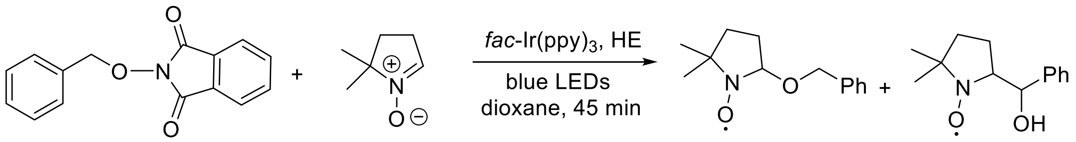

49 **50** **51** **52**

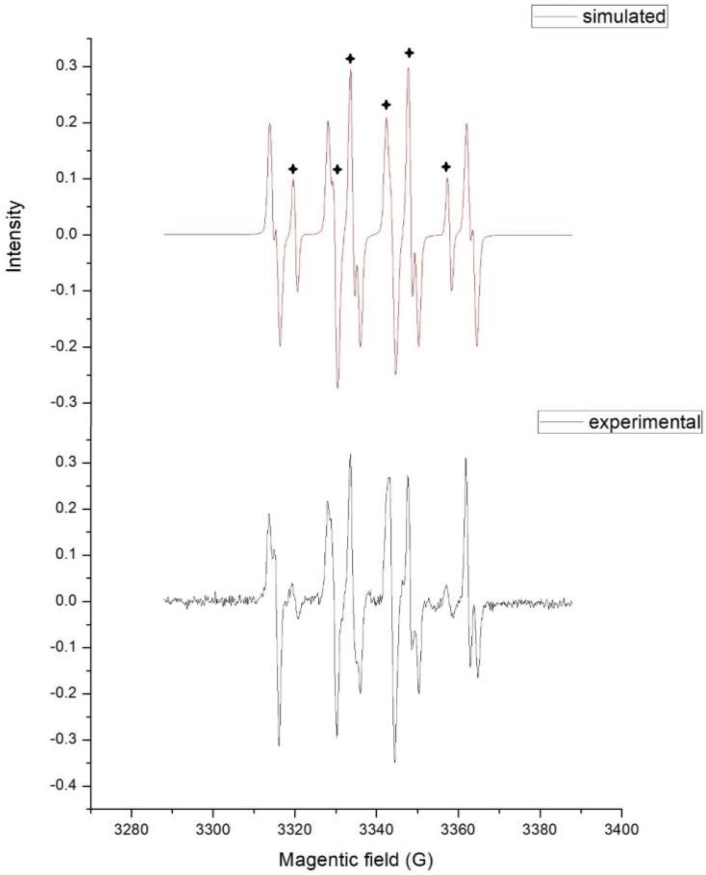

Scheme S12. EPR Spectrum of Spin Adducts in Dioxane, Related to Scheme 4.

The alkyl radical addition adduct **43-adduct** was found. Instrumental parameters: ν = 9.37 GHz, modulation frequency = 100 kHz, modulation amplitude = 1.00 G, microwave power = 7.96 mW, conversion time = 58.59 ms, time constant = 0 ms, sweep time = 60 s. The coupling constants of adduct DMPO-CH is a_N = 14.3 G and $a_{H\beta}$ = 20.1 G.

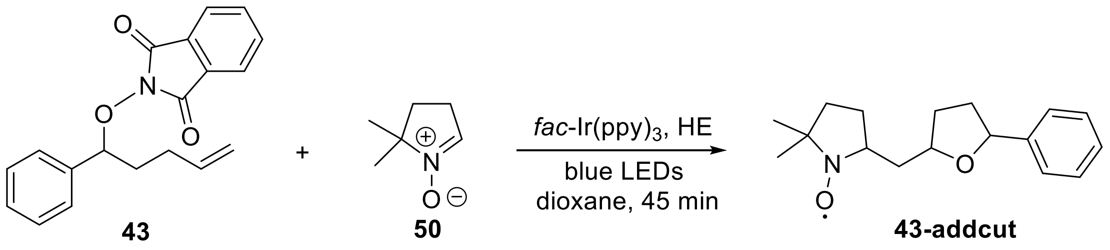

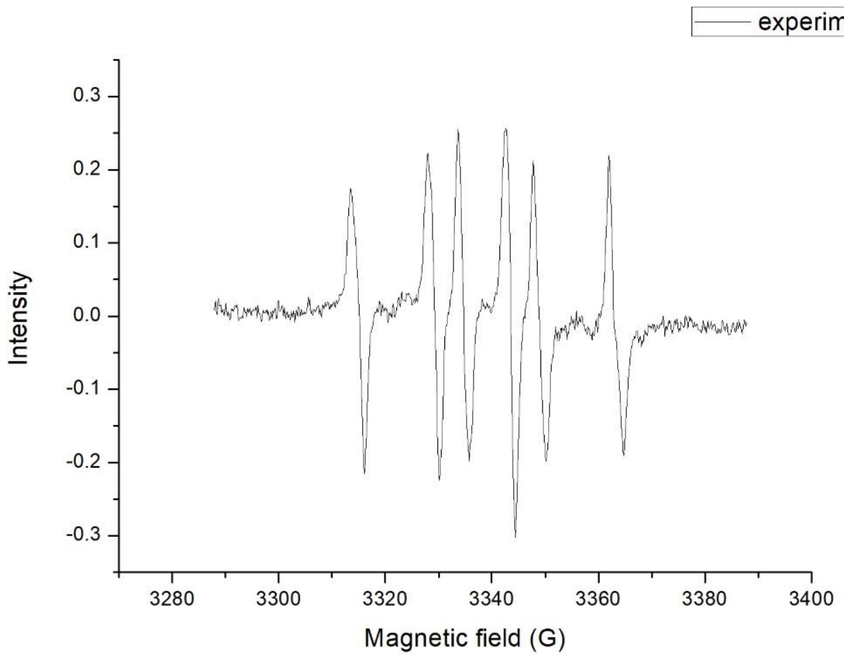

Scheme S13. EPR Spectrum of Spin Adduct in Dioxane, Related to Scheme 4.

DFT Calculation

Geometry	$E_{(elec-B3LYP)}$[1]	$H_{(corr-B3LYP)}$[2]	$G_{(corr-B3LYP)}$[3]	$E_{(solv-M11)}$[4]	IF[5]
**CP1**	-1203.234367	0.356076	0.279012	-1203.054513	-
**$HE^{\cdot+}$**	-862.231455	0.330610	0.258334	-862.095451	-
**CP2**	-1203.749639	0.369532	0.291021	-1203.493082	-
**$E^{\cdot}$**	-861.846357	0.316992	0.245785	-861.647058	-
**TS1**	-1203.725890	0.366709	0.287669	-1203.459777	-654.9
**CP3**	-690.707331	0.241721	0.184576	-690.528852	-
**CP4**	-513.047076	0.124823	0.083306	-512.948779	-
**TS2**	-690.660762	0.236711	0.179942	-690.499607	-2017.5
**CP5**	-690.748343	0.242768	0.186119	-690.583403	-
**TS3**	-1203.174837	0.352692	0.276452	-1202.988998	-338.5
**CP6**	-1203.206799	0.354285	0.277336	-1203.016213	-
**TS4**	-1203.198201	0.351380	0.275214	-1202.994127	-398.4
**CP7**	-690.167476	0.230970	0.174412	-690.007959	-
**CP8**	-513.070911	0.121791	0.079440	-513.072304	-
**MeOH**	-115.712204	0.055708	0.028753	-115.704694	-
**TS5**	-806.401221	0.294046	0.228819	-806.230046	-1206.9
**TS6**	-922.153016	0.352611	0.277625	-921.965704	-743.9
**TS7**	-1037.893693	0.410793	0.325078	-1037.684964	-752.6
**TS8**	-690.692319	0.240238	0.185178	-690.522421	-64.8
**CP9**	-690.700720	0.241164	0.185274	-690.530673	-
**TS9**	-690.690385	0.235426	0.179987	-690.521347	-1362.4
**CP10**	-690.718053	0.241179	0.183760	-690.557158	-

Table S4. B3LYP geometries for all the optimized compounds and transition states, Related to Scheme 5.

[1]The electronic energy calculated by B3LYP in gas phase.

[2]The thermal correction to enthalpy calculated by B3LYP in gas phase.

[3]The thermal correction to Gibbs free energy calculated by B3LYP in gas phase.

[4]The electronic energy calculated by M11 in methanol solvent.

[5]The B3LYP calculated imaginary frequencies for the transition states.

Table S5. B3LYP and M11 absolute calculation energies, enthalpies, and free energies, Related to Scheme 5.

The Gibbs free energy profiles for the MeOH/H_2O-assisted 1,2-HAT reaction are shown in Scheme S14. The direct hydrogen atom transfer to form the ketyl radical

CP5-1 was calculated to have 16.0 kcal/mol of activation barrier in **TS2-1** (red line). With one molecule of H_2O assisted (blue line), the activation barrier of the hydrogen atom transfer transition state **TS3-1** could be increased to be 16.2 kcal/mol. Significantly, one methanol and one H_2O molecules decrease the activation barrier to 6.9 kcal/mol in **TS4-1** (black line), while two methanol and one H_2O molecules can lower the activation barrier to 9.4 kcal/mol in **TS5-1** (purple line). The calculated results indicate that one methanol and one H_2O molecules decrease the activation barrier to merely 6.9 kcal/mol with multiple hydrogen bonds formation.

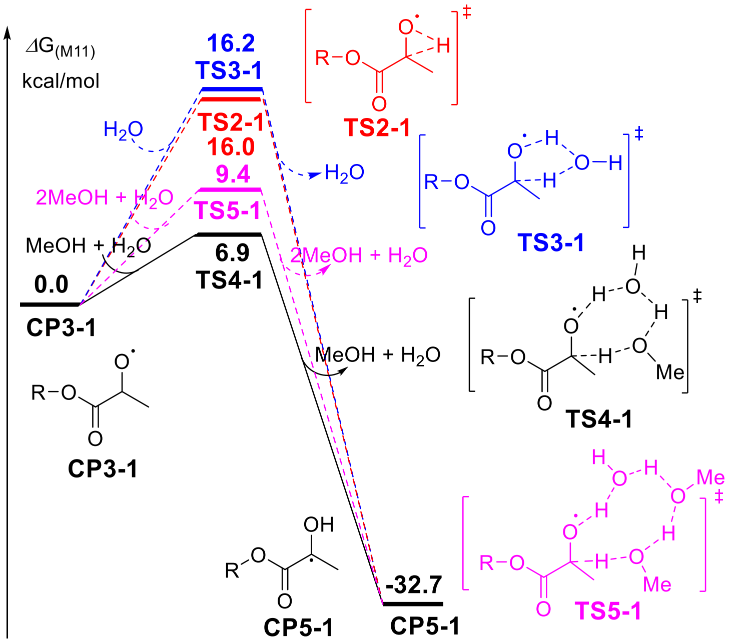

Scheme S14. **Gibbs Free Energy Profiles for the MeOH/H_2O-assisted 1,2-HAT reaction, Related to Scheme 5.**

Geometry	$E_{(elec-B3LYP)}$[1]	$H_{(corr-B3LYP)}$[2]	$G_{(corr-B3LYP)}$[3]	$E_{(solv-M11)}$[4]	IF[5]
**CP3-1**	-690.708645	0.241817	0.184734	-690.529933	-
**TS2-1**	-690.660762	0.236711	0.179942	-690.499607	-2017.5
**TS3-1**	-767.093055	0.262944	0.202253	-766.952305	-1363.3
**H_2O**	-76.407024	0.024920	0.002821	-76.433519	-
**TS4-1**	-882.849357	0.322367	0.252417	-882.693235	-746.6
**MeOH**	-115.712204	0.055708	0.028753	-115.704694	-
**TS5-1**	-998.589743	0.380337	0.299903	-998.412740	-748.4
**CP5-1**	-690.748343	0.242768	0.186119	-690.583403	-

Table S6 B3LYP and M11 absolute calculation energies, enthalpies, and free energies, Related to Scheme 5.

[1]The electronic energy calculated by B3LYP in gas phase.
[2]The thermal correction to enthalpy calculated by B3LYP in gas phase.
[3]The thermal correction to Gibbs free energy calculated by B3LYP in gas phase.
[4]The electronic energy calculated by M11 in methanol solvent.
[5]The B3LYP calculated imaginary frequencies for the transition states.

Table S7. B3LYP geometries for all the optimized compounds and transition states, Related to Scheme 5.

III. Transparant Methods:

General Procedures

Unless otherwise noted, all reactions of substrates preparation were conducted in flame-dried glassware under a nitrogen atmosphere using anhydrous solvent passed through an activated alumina column (Innovative Technology). Commercially available anhydrous MeOH was treated by 4 Å MS. Commercially available reagents were used without further purification. Hantzsch ester (HE) was recrystallized from ethanol and 1,4-dioxane was distilled over sodium. Thin layer chromatography (TLC) was performed using Jiangyou TLC silica gel plates HSG F_{254} and visualized using UV light, and potassium permanganate. Flash chromatography was performed on Lisure science EZ purification system using the Santai technologies silica gel cartridge. Preparative thin layer chromatography separations were carried out on 0.25 or 0.50 mm E. Merck silica gel plates (60F-254). Photochemical reactions were carried with 4.8 W blue LED (ZL-3036R) obtained from Beijing Jolly Lighting Engineering Co. Ltd. ^{1}H and ^{13}C NMR spectra were recorded in $CDCl_3$, unless otherwise noted, on a Bruker AV-400 MHz or an Agilent 500 MHz spectrometer. Chemical shifts in ^{1}H NMR spectra were reported in parts per million (ppm) on the δ scale from an internal standard of residual $CDCl_3$ (7.26 ppm). Data for ^{1}H NMR were reported as follows: chemical shift, multiplicity (s = singlet, d = doublet, t = triplet, q = quartet, m = multiplet), coupling constant in Herts (Hz) and integration. Data for ^{13}C NMR spectra were reported in terms of chemical shift in ppm from the central peak of $CDCl_3$ (77.16 ppm). IR spectra were recorded on a Thermo Scientific Nicolet 380 FT-IR spectrometer. MS experiments were performed on a Bruker maXis 4G instrument for HRMS-ESI, an Agilent 5973N instrument for EI-MS, and a Waters Micromass GCT Premier instrument for HRMS-EI. Cyclic Voltammetry was performed on a CH Instruments Electrochemical Workstation model CHI600E.

Synthesis of *N*-alkoxyphthalimide Precursors

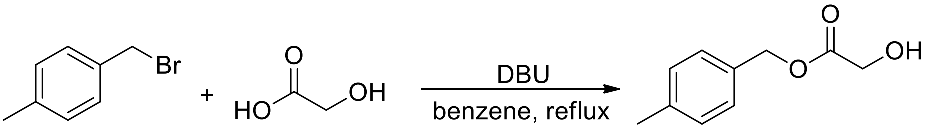

Scheme S15. Synthetic Procedure, Related to Scheme 2

To a solution of 4-methylbenzyl bromide (1.11 g, 6.0 mmol) and DBU (0.76 g, 5.0 mmol) in benzene (12 mL) was stirred for 15 min at room temperature. The reaction mixture was slowly added glycolic acid (0.38 g, 5.0 mmol) and refluxed for 6 hours. The resulting reaction mixture was extracted with 1 M aqueous HCl (2 x 20 mL). The organic layers were washed with brine, dried over anhydrous Na_2SO_4, and concentrated in vacuo. The crude product was purified by column chromatography on silica gel (20% hexanes/EtOAc) to give **A1** as a colorless oil.

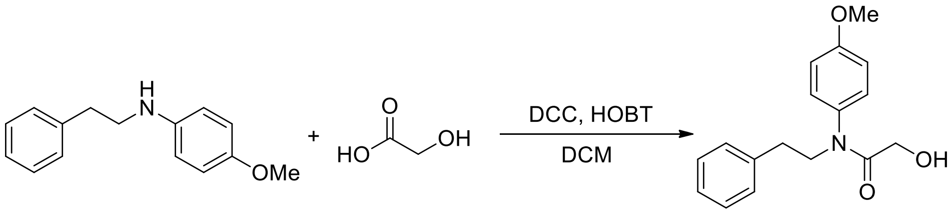

Scheme S16. Synthetic Procedure, Related to Scheme 2

To a solution of 4-methoxy-*N*-phenethylaniline (0.68 g, 3.0 mmol), glycolic acid (0.19 g, 2.5 mmol) and 1-hydroxybenzotriazole (0.68 g, 5.0 mmol) in DCM (10 mL) was added dicyclohexylcarbodiimide (0.78 g, 3.8 mmol) at 0 °C. The resulting suspension was filtered and the filtrate was concentrated in vacuo. The crude product was purified by column chromatography on silica gel (20% hexanes/EtOAc) to give **A2** as a colorless oil.

Synthesis of N-alkoxyphthalimide substrates

Method A

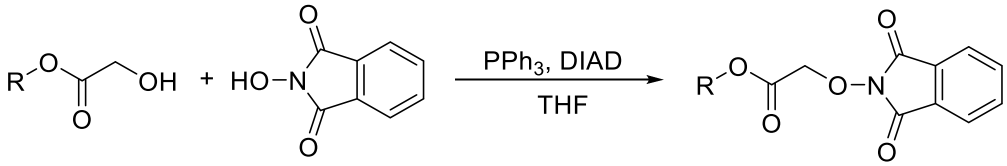

Scheme S17. Synthetic Procedure, Related to Scheme 2

To a solution of the alcohol (10.0 mmol), PPh_3 (3.15 g, 12.0 mmol), and N-hydroxyphthalimide (1.96 g, 12.0 mmol) in THF (30 mL) was added diisopropyl azodicarboxylate (2.4 mL, 12.0 mmol) over 10 min at room temperature. The resulting mixture was stirred for 3-24 h, taken up in EtOAc (20 mL), and washed with saturated $NaHCO_3$ (3 x 20 mL) and brine (2 x 30 mL). The organic layers were dried over anhydrous Na_2SO_4, concentrated in vacuo, and subjected to flash chromatography to afford the *N*-alkoxyphthalimides.

Method B

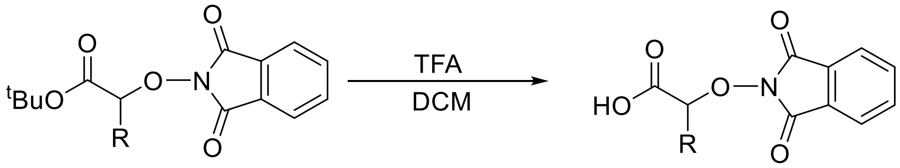

Scheme S18. Synthetic Procedure, Related to Scheme 2

To a solution of *N*-alkoxyphthalimides (33 mmol) in 110 mL DCM was slowly added TFA (37.0 mL, 495 mmol) at 0 °C. The reaction mixture was stirred at room temperature under N_2 for 2 hours. The reaction was then concentrated and azeotroped with DCM to afford *N*-alkoxyphthalimides and the crude product was directly subjected to the next reaction without further purification.

Method C

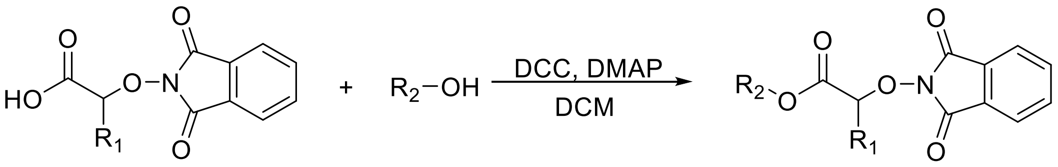

Scheme S19. Synthetic Procedure, Related to Scheme 2

To a solution of *N*-alkoxyphthalimides (1.5 mmol), alcohol (1.0 mmol) and DMAP (61.1 mg, 0.5 mmol) in 10 mL DCM was added N,N'-dicyclohexylcarbodiimide (0.31 g, 1.5 mmol) at 0 °C. The reaction mixture was stirred at 0 °C for 30 min and continued stirring at 25 °C overnight. The resulting suspension was filtered and the filtrate was concentrated in vacuo. Purification by column chromatography afforded the *N*-alkoxyphthalimides.

Method D

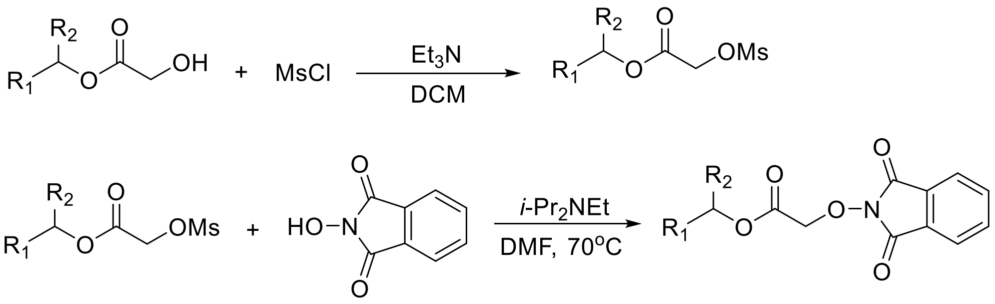

Scheme S20. Synthetic Procedure, Related to Scheme 2

To a solution of the alcohol (10 mmol) and Et_3N (2.2 mL, 16 mmol) in CH_2Cl_2 (30 mL) was added methanesulfonyl chloride (0.93 mL, 12 mmol) dropwise over 5 min at 0 °C. The reaction mixture was then allowed to warm to ambient temperature and stirred for 1 h. It was then washed with brine (3 x 30 mL). The organic layer was dried over Na_2SO_4, the solvent was removed by rotary evaporation to provide a yellow oil. The crude mesylate and directly mixed with N-hydroxyphthalimide (2.61 g, 16.0 mmol) and diisopropylethylamine (3.5 mL, 20 mmol) in DMF (20 mL). The resulting reaction mixture was stirred at 70 °C for 3 h and allowed to cool to room

temperature. The mixture was then taken up in Et_2O (50 mL), washed with sat. $NaHCO_3$ solution (3 x 25 mL), and brine (50 mL). It was then dried over Na_2SO_4, concentrated in vacuo, puried by column chromatography to afford the *N*-alkoxyphthalimides.

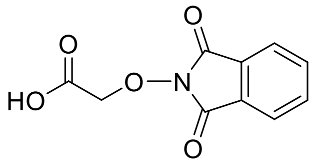

2-((1,3-dioxoisoindolin-2-yl)oxy)acetic acid (A3). Following the general method B, the reaction of *tert*-butyl 2-((1,3-dioxoisoindolin-2-yl)oxy)acetate (9.12 g, 33.0 mmol) afforded *N*-alkoxyphthalimides **A3** as a white solid (7.49 g, 100% yield) and the crude product was directly subjected to the next reaction without further purification.

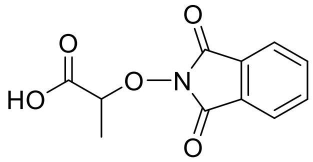

2-((1,3-dioxoisoindolin-2-yl)oxy)acetic acid (A4). Following the general method B, the reaction of *tert*-butyl 2-((1,3-dioxoisoindolin-2-yl)oxy)propanoate (6.89 g, 23.6 mmol) afforded *N*-alkoxyphthalimides **A4** as a white solid (5.54 g, 100% yield) and the crude product was directly subjected to the next reaction without further purification.

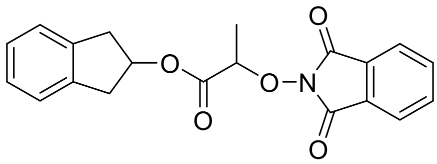

2,3-Dihydro-1*H*-inden-2-yl 2-((1,3-dioxoisoindolin-2-yl)oxy)propanoate (1). Following the general method C, the reaction of 2-indanol (1.34 g, 10.0mmol) afforded *N*-alkoxyphthalimides **1** as a white solid**.**

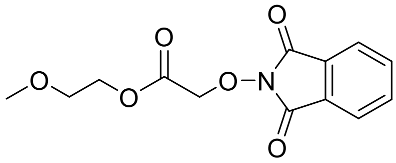

2-Methoxyethyl 2-((1,3-dioxoisoindolin-2-yl)oxy)acetate (6). Following the general method C, the reaction of 2-methoxyethanol (0.23 g, 3.0 mmol) afforded N-alkoxyphthalimides **6** as a white solid.

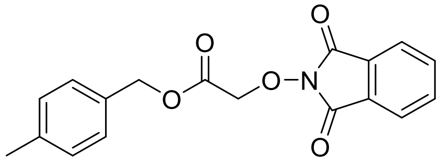

4-Methylbenzyl 2-((1,3-dioxoisoindolin-2-yl)oxy)acetate (8). Following the general method D, the reaction of **A1** (0.62 g, 3.5 mmol) afforded *N*-alkoxyphthalimides **8** as a white solid**.**

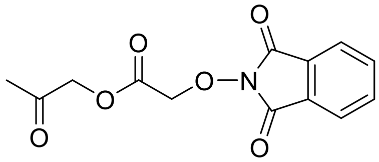

2-Oxopropyl 2-((1,3-dioxoisoindolin-2-yl)oxy)acetate (10). Following the general method C, the reaction of hydroxyacetone (0.15 g, 1.0 mmol) afforded *N*-alkoxyphthalimides **10** as a white solid.

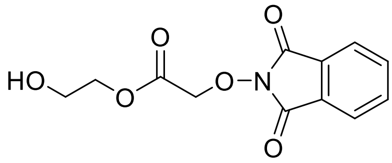

2-Hydroxyethyl 2-((1,3-dioxoisoindolin-2-yl)oxy)acetate (12). Following the general method C, the reaction of ethylene glycol (0.16 g, 2.5 mmol) and *N*-alkoxyphthalimides **12** (0.83 g, 3.8 mmol) afforded *N*-alkoxyphthalimides **12** as a white solid.

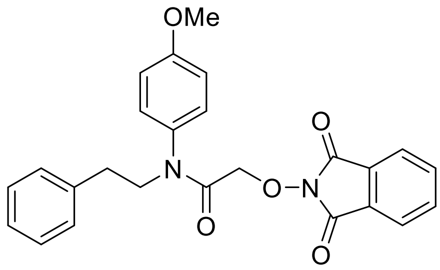

2-((1,3-Dioxoisoindolin-2-yl)oxy)-*N*-(4-methoxyphenyl)-N-phenethylacetamide (14). Following the general method A, the reaction of **A2** (0.43 g, 1.5 mmol) afforded *N*-alkoxyphthalimides **14** as a white solid**.**

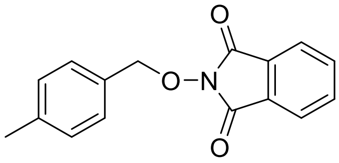

2-((4-Methylbenzyl)oxy)isoindoline-1,3-dione (16). Following the generalmethod A, the reaction of 4-methylbenzyl alcohol (1.22 g, 10.0 mmol) afforded *N*-alkoxyphthalimides **16** as a white solid**.**

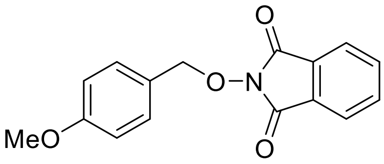

2-((4-Methoxybenzyl)oxy)isoindoline-1,3-dione (18). Following the general method A, the reaction of 4-methoxybenzyl alcohol (1.38 g, 10.0 mmol) afforded *N*-alkoxyphthalimides **18** as a white solid.

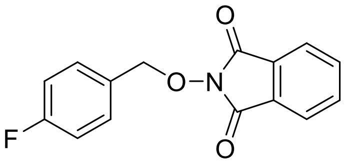

2-(4-fluorobenzyloxy)isoindoline-1,3-dione (20). Following the general method A, the reaction of 4-fluorobenzyl alcohol (1.26 g, 10.0 mmol) afforded *N*-alkoxyphthalimides **20** as a white solid.

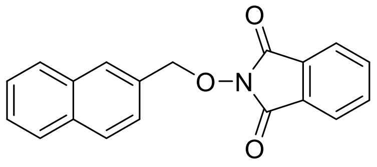

2-(Naphthalen-2-ylmethoxy)isoindoline-1,3-dione (22). Following the general method A, the reaction of 2-naphthalenemethanol (0.40 g, 2.6 mmol) afforded *N*-alkoxyphthalimides **22** as a white solid.

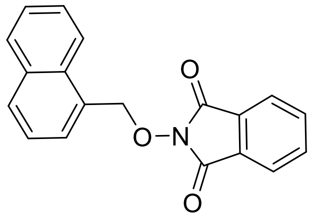

2-(Naphthalen-1-ylmethoxy)isoindoline-1,3-dione (24). Following the general method A, the reaction of 1-naphthalenemethanol (0.79 g, 5.0 mmol) afforded *N*-alkoxyphthalimides **24** as a white solid.

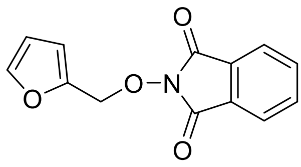

2-(Furan-2-ylmethoxy)isoindoline-1,3-dione (26). Following the general method A, the reaction of furfuryl alcohol (0.98 g, 10.0 mmol) afforded *N*-alkoxyphthalimides **26** as a white solid.

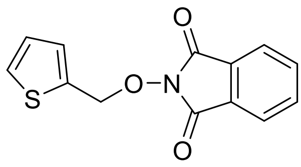

2-(Thiophen-2-ylmethoxy)isoindoline-1,3-dione (28). Following the general method A, the reaction of 2-thiophenemethanol (1.14 g, 10.0 mmol) afforded *N*-alkoxyphthalimides **28** as a white solid.

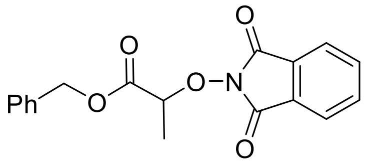

Benzyl 2-((1,3-dioxoisoindolin-2-yl)oxy)propanoate (30). Following the general method A, the reaction of benzyl 2-hydroxypropanoate (0.53 g, 3.0 mmol) afforded *N*-alkoxyphthalimides **30** as a white solid.

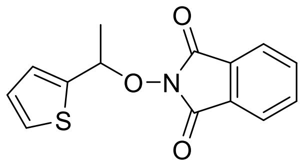

2-(1-(thiophen-2-yl)ethoxy)isoindoline-1,3-dione (32). Following the general method A, the reaction of 1-(thiophen-2-yl)ethan-1-ol (1.28 g, 10.0 mmol) afforded *N*-alkoxyphthalimides **32** as a white solid.

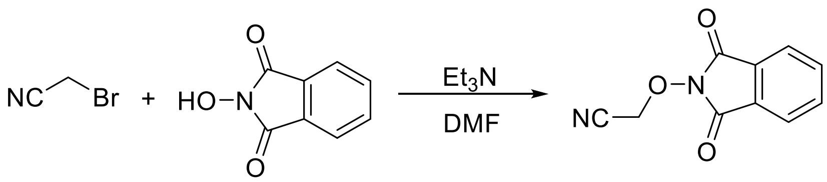

Scheme S21. Synthetic Procedure, Related to Scheme 2

To a solution of the N-hydroxyphthalimide (1.63 g, 10.0 mmol) in DMF (10 mL) was added Et_3N (3.1 mL, 22.0 mmol) and bromoacetonitrile (1.44 g, 12.0 mmol) at 0 ^oC. The reaction mixture was warmed to room temperature and stirred overnight. The mixture was then taken up in EtOAc (100 mL), washed with H_2O (3 x 30 mL), and brine (30 mL). It was then dried over Na_2SO_4, concentrated in vacuo, puried by column chromatography to afford **34** as a white solid.

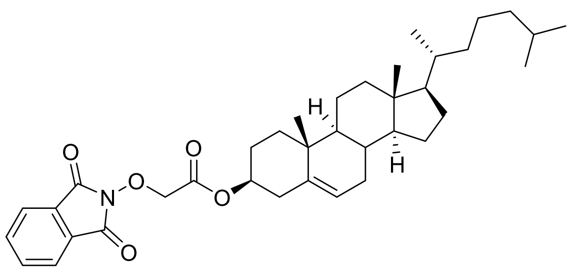

(3S,9S,10R,13R,14S,17R)-10,13-dimethyl-17-(6-methylheptan-2-yl)-2,3,4,7,8,9,10,11,12,13,14,15,16,17-tetradecahydro-1H-cyclopenta[a]phenanthren-3-yl 2-((1,3-dioxoisoindolin-2-yl)oxy)acetate (40). Following the general method C, the reaction of cholesterol (0.77 g, 2.0mmol) afforded *N*-alkoxyphthalimides **40** as a white solid**.**

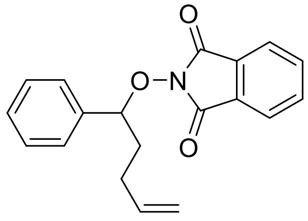

2-((1-Phenylpent-4-en-1-yl)oxy)isoindoline-1,3-dione (43). Following the general method A, the reaction of 1-phenylpent-4-en-1-ol (1.26 g, 8.0 mmol) afforded *N*-alkoxyphthalimides **43** as a white solid.

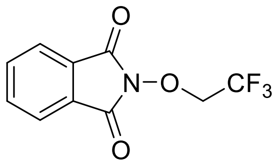

2-(2,2,2-trifluoroethoxy)isoindoline-1,3-dione(37). Following the general method D, the reaction of 2,2,2-trifluoroethan-1-ol (1.00 g, 10.0 mmol) afforded *N*-alkoxyphthalimides **37** as a white solid.

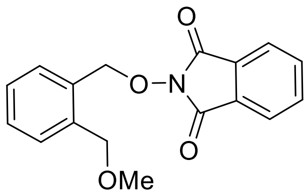

2-((2-(methoxymethyl)benzyl)oxy)isoindoline-1,3-dione. (46) Following the general method A, the reaction of (2-(methoxymethyl)phenyl)methanol (1.52 g, 10.0 mmol) afforded *N*-alkoxyphthalimides **46** as a white solid.

Synthesis of Allyl Sulfones

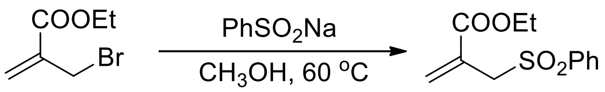

Scheme S22. Synthetic Procedure, Related to Scheme 2

Ethyl-2-((phenylsulfonyl)methyl)acrylate (2). To a solution of **B2** (1.99 g, 10.4 mmol) in dry methanol (25 mL) was added sodium phenylsulfinate (2.50 g, 15.2 mmol). After 2.5 h of reflux, the mixture was concentrated under reduced pressure, the obtained residue was dissolved in EtOAc and the mixture was washed with water, brine, dried with Na_2SO_4, filtered and the filtrate was evaporated and purified by chromatography (50% EtOAc/hexanes) to give **2** as a viscous oil.

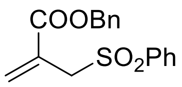

Benzyl-2-((phenylsulfonyl)methyl)acrylate (2-a). Following the above procedure, the reaction of benzylacrylate (3.24 g, 20.0 mmol) afforded **2-a** as a white solid.

Procedure for Allylation

standard procedure for allylation:

A solution of *N*-alkoxyphthalimides (0.1 mmol, 1.0 equiv.), allyl sulfone (0.3 mmol, 3.0 equiv.) and Hantzsch ester (38.0 or 76.0 mg, 0.15 or 0.3 mmol, 1.5 or 3.0 equiv.) was placed in a 5 mL clear-colored glass vial. After 1.0 mL MeOH (bubbled with nitrogen gas for 30 minutes to remove oxygen) was added, the vial was sealed and exposed to 4W blue LED at room temperature with stirring for appropriate hours. The reaction mixture was concentrated and purified directly by column chromatography to afford the allylation adduct.

*The heating effect from LED irradiation conditions above is minimal. With 6-12 hours irradiation, the increase of temperature is less than 5°C.

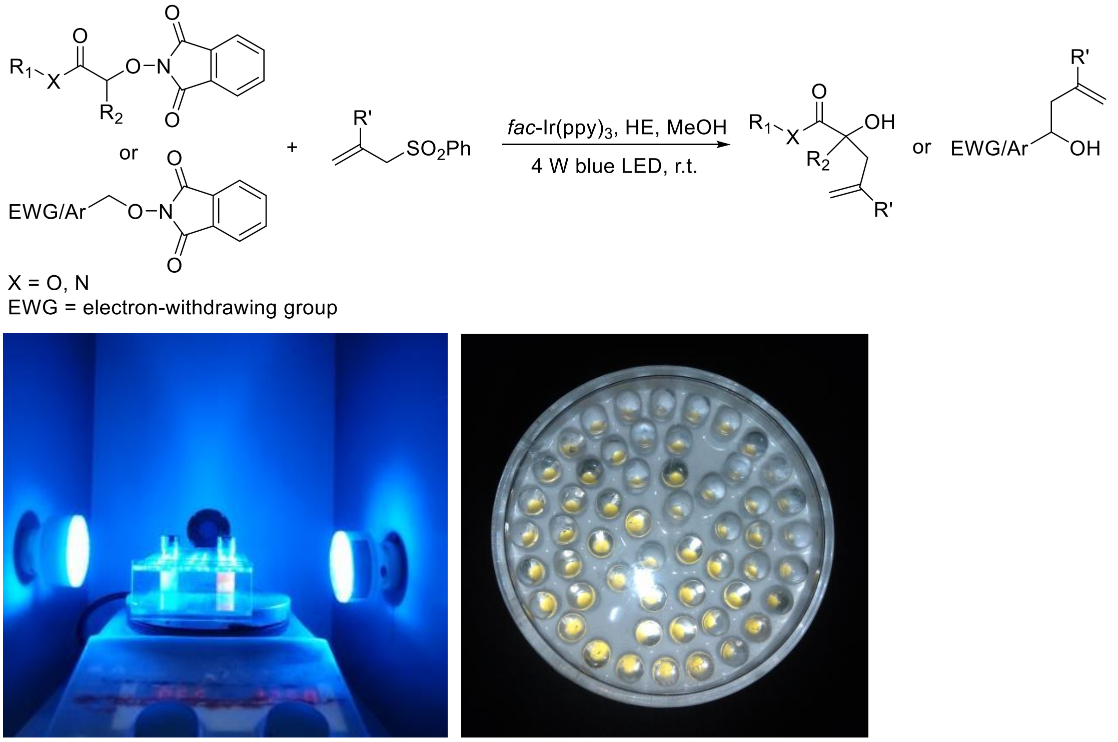

Scheme S23. Synthetic Procedure, Related to Scheme 2

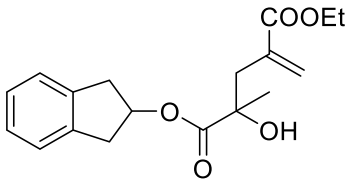

1-(2,3-Dihydro-1*H*-inden-2-yl) 5-ethyl 2-hydroxy-2-methyl-4-methylenepentane-dioate (4). Following the standard procedure, the reaction of *N*-alkoxyphthalimides **1** (35.1 mg, 0.1 mmol), Hantzsch ester (38.0 mg, 0.15 mmol) and allyl sulfone **2** (76.2 mg, 0.3 mmol) in 1 mL MeOH for 3 h afforded target product **4** as a colorless oil .

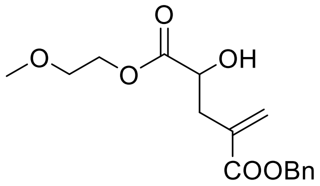

1-Benzyl 5-(2-methoxyethyl) 4-hydroxy-2-methylenepentanedioate (7). Following the standard procedure, the reaction of *N*-alkoxyphthalimides **6** (27.9 mg, 0.1 mmol), Hantzsch ester (38.0 mg, 0.15 mmol) and allyl sulfone **2-a** (94.8 mg, 0.3 mmol) in 1 mL MeOH for 3 h afforded target product **7** as a light yellow oil.

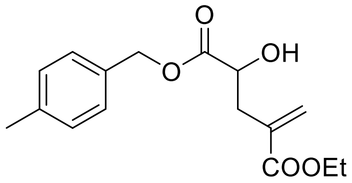

1-Ethyl 5-(4-methylbenzyl) 4-hydroxy-2-methylenepentanedioate (9). Following the standard procedure, the reaction of *N*-alkoxyphthalimides **8** (32.5 mg, 0.1 mmol), Hantzsch ester (38.0 mg, 0.15 mmol) and allyl sulfone **2** (76.2 mg, 0.3 mmol) in 1 mL MeOH for 3 h afforded target product **9** as a light yellow oil.

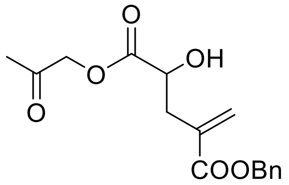

1-Benzyl 5-(2-oxopropyl) (*S*)-4-hydroxy-2-methylenepentanedioate (11).

Following the standard procedure, the reaction of *N*-alkoxyphthalimides **10** (27.7 mg, 0.1 mmol), Hantzsch ester (38.0 mg, 0.15 mmol) and allyl sulfone **2-a** (94.8 mg, 0.3 mmol) in 1 mL MeOH for 3 h afforded target product **11** as a light yellow oil.

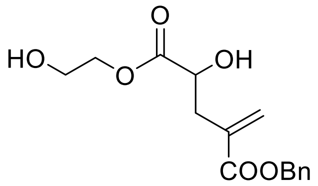

1-Benzyl 5-(2-hydroxyethyl) 4-hydroxy-2-methylenepentanedioate (13). Following the standard procedure, the reaction of *N*-alkoxyphthalimides **12** (26.5 mg, 0.1 mmol), Hantzsch ester (38.0 mg, 0.15 mmol) and allyl sulfone **2-a** (94.8 mg, 0.3 mmol) in 1 mL MeOH for 3 h afforded target product **13** as a light yellow oil.

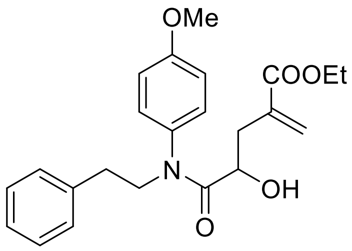

Ethyl 4-hydroxy-5-((4-methoxyphenyl)(phenethyl)amino)-2-methylene-5-oxopentanoate (15). Following the standard procedure, the reaction of *N*-alkoxyphthalimides **14** (43.0 mg, 0.1 mmol), Hantzsch ester (38.0 mg, 0.15 mmol) and allyl sulfone **2** (76.2 mg, 0.3 mmol) in 1 mL MeOH for 3 h afforded target product **15** as a light yellow oil.

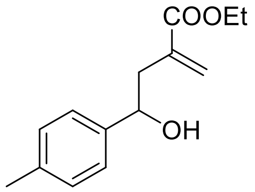

Ethyl 4-hydroxy-2-methylene-4-(p-tolyl)butanoate (17). Following the standard procedure, the reaction of *N*-alkoxyphthalimides **16** (26.7 mg, 0.1 mmol), Hantzsch

ester (38.0 mg, 0.15 mmol) and allyl sulfone **2** (76.2 mg, 0.3 mmol) in 1 mL MeOH for 6 h afforded target product **17** as a light yellow oil.

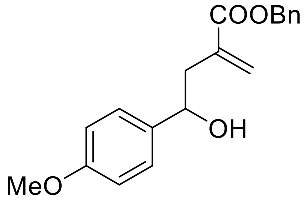

Benzyl 4-hydroxy-4-(4-methoxyphenyl)-2-methylenebutanoate (19). Following the standard procedure, the reaction of *N*-alkoxyphthalimides **18** (28.3 mg, 0.1 mmol), Hantzsch ester (38.0 mg, 0.15 mmol) and allyl sulfone **2-a** (94.8 mg, 0.3 mmol) in 1 mL MeOH for 6 h afforded target product **19** as a light yellow oil.

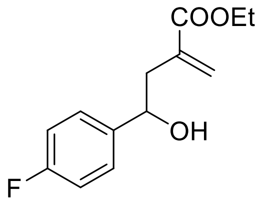

Ethyl 4-(4-fluorophenyl)-4-hydroxy-2-methylenebutanoate (21). Following the standard procedure, the reaction of *N*-alkoxyphthalimides **20** (27.1 mg, 0.1 mmol), Hantzsch ester (38.0 mg, 0.15 mmol) and allyl sulfone **2** (76.2 mg, 0.3 mmol) in 1 mL MeOH for 3 h afforded target product **21** as a light yellow oil.

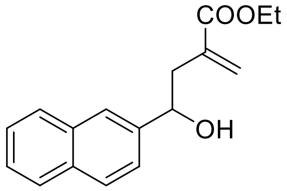

Ethyl 4-hydroxy-2-methylene-4-(naphthalen-2-yl)butanoate (23). Following the standard procedure, the reaction of *N*-alkoxyphthalimides **22** (30.3 mg, 0.1 mmol), Hantzsch ester (76.0 mg, 0.3 mmol) and allyl sulfone **2** (76.2 mg, 0.3 mmol) in 1 mL MeOH for 12 h afforded target product **23** as a light yellow oil.

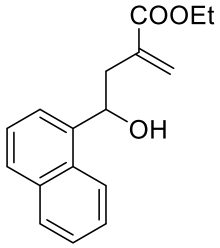

Ethyl 4-hydroxy-2-methylene-4-(naphthalen-1-yl)butanoate (25). Following the standard procedure, the reaction of *N*-alkoxyphthalimides **24** (30.3 mg, 0.1 mmol), Hantzsch ester (38.0 mg, 0.15 mmol) and allyl sulfone **2** (76.2 mg, 0.3 mmol) in 1 mL MeOH for 6 h afforded target product **25** as a light yellow oil.

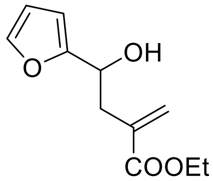

Ethyl 4-(furan-2-yl)-4-hydroxy-2-methylenebutanoate (27). Following the standard procedure, the reaction of *N*-alkoxyphthalimides **26** (24.3 mg, 0.1 mmol), Hantzsch ester (76.0 mg, 0.3 mmol) and allyl sulfone **2** (76.2 mg, 0.3 mmol) in 1 mL MeOH for 12 h afforded target product **27** as a light yellow oil.

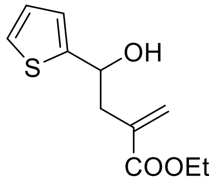

Ethyl 4-hydroxy-2-methylene-4-(thiophen-2-yl)butanoate (29). Following the standard procedure, the reaction of *N*-alkoxyphthalimides **28** (25.9 mg, 0.1 mmol), Hantzsch ester (38.0 mg, 0.15 mmol) and allyl sulfone **2** (76.2 mg, 0.3 mmol) in 1 mL MeOH for 6 h afforded target product **29** as a light yellow oil.

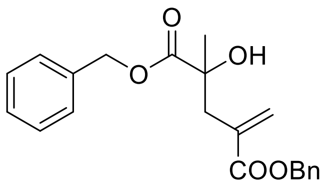

Dibenzyl 2-hydroxy-2-methyl-4-methylenepentanedioate (31). Following the standard procedure, the reaction of *N*-alkoxyphthalimides **30** (32.5 mg, 0.1 mmol), Hantzsch ester (38.0 mg, 0.15 mmol) and allyl sulfone **2-a** (94.8 mg, 0.3 mmol) in 1 mL MeOH for 3 h afforded target product **31** as a colourless oil.

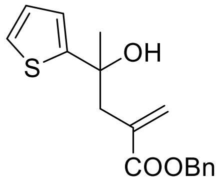

Benzyl 4-hydroxy-2-methylene-4-(thiophen-2-yl)pentanoate (33). Following the standard procedure, the reaction of *N*-alkoxyphthalimides **32** (27.3 mg, 0.1 mmol), Hantzsch ester (38.0 mg, 0.15 mmol) and allyl sulfone **2-a** (94.8 mg, 0.3 mmol) in 1 mL MeOH for 12 h afforded target product **33** as a colourless oil.

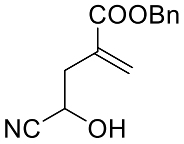

Benzyl 4-cyano-4-hydroxy-2-methylenebutanoate (35). Following the standard procedure, the reaction of *N*-alkoxyphthalimides **34** (20.2 mg, 0.1 mmol), Hantzsch ester (38.0 mg, 0.15 mmol) and allyl sulfone **2-a** (94.8 mg, 0.3 mmol) in 1 mL MeOH for 6 h afforded target product **35** as a light yellow oil.

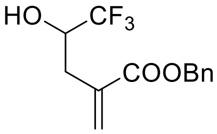

benzyl 5,5,5-trifluoro-4-hydroxy-2-methylenepentanoate (**38**). Following the standard procedure, the reaction of *N*-alkoxyphthalimides **37** (25.4 mg, 0.1 mmol), Hantzsch ester (38.0 mg, 0.15 mmol) and allyl sulfone **2-a** (94.8 mg, 0.3 mmol) in 1 mL MeOH for 6 h afforded target product **38** as a colourless oil.

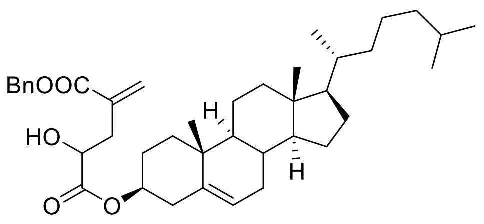

1-Benzyl 5-((3S,9S,10R,13R,14S,17R)-10,13-dimethyl-17-(6-methylheptan-2-yl)-2,3,4,7,8,9,10,11,12,13,14,15,16,17-tetradecahydro-1H-cyclopenta[a]phenanthren-3-yl) 4-hydroxy-2-methylenepentanedioate (41). Following the standard procedure, the reaction of *N*-alkoxyphthalimides **40** (58.9 mg, 0.1 mmol), Hantzsch ester (38.0 mg, 0.15 mmol) and allyl sulfone **2-a** (94.8 mg, 0.3 mmol) in 1 mL MeOH for 12 h afforded target product **41** as a colourless oil.

Procedure for the Cross-Over Experiment

Following the standard procedure for allylations, the reaction of **16** (26.7 mg, 0.1 mmol), **42** (15.8 mg, 0.1 mmol) and allyl sulfone **2** (76.2 mg, 0.3 mmol) for 6 h.

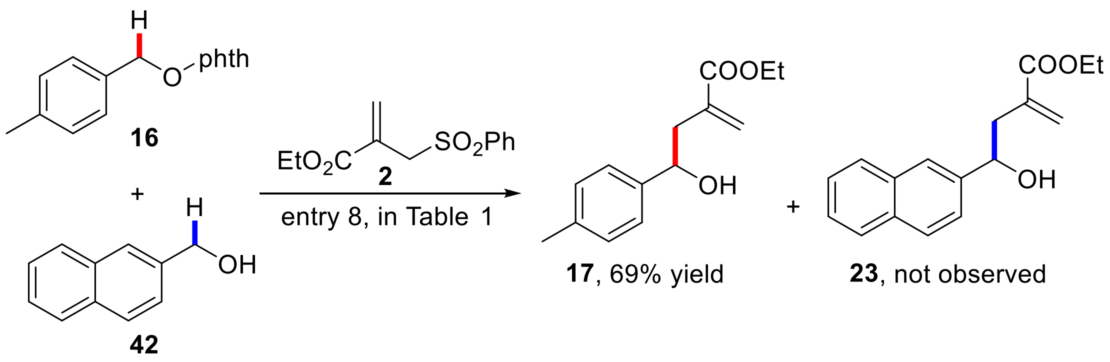

Scheme S24. Cross-Over Experiment, Related to Scheme 3

Procedure for the KIE experiments

A solution of *N*-alkoxyphthalimides **1/ D-1** (0.3 mmol, 1.0 equiv.), allyl sulfone **2** (0.9 mmol, 3.0 equiv.), Hantzsch ester (0.45 mmol, 1.5 equiv.) and 1,3,5-trimethoxybenzene (0.3 mmol, 1.0 equiv.) was placed in an 8 mL clear-colored glass vial. After 3.0 mL MeOH was added, the vial was sealed and exposed to 4W blue LED at room temperature with stirring. The reaction mixture was tested at different time points by ^{1}H-NMR.

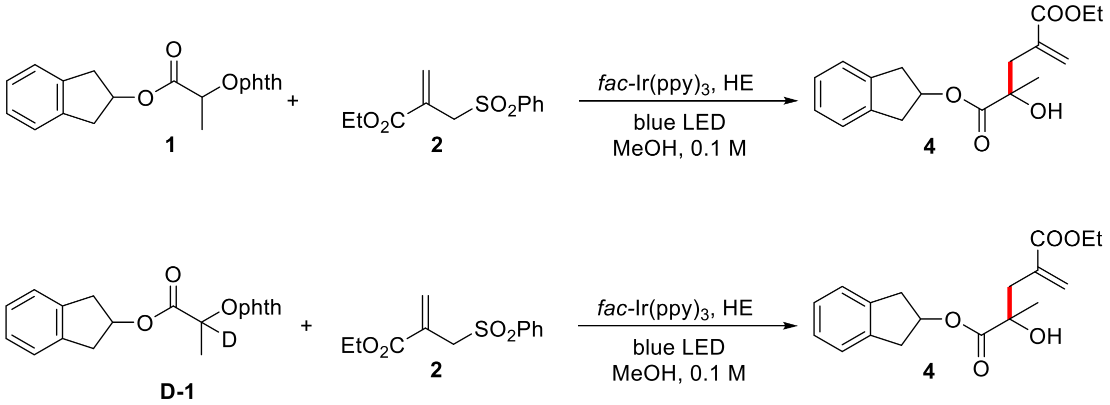

Scheme S25. KIE experiments, Related to Scheme 3.

Procedure for the 1,2-HAT Competes with Other Alkoxyl Radical Reaction Pathways

Following the standard procedure without the addition of allyl sulfone, the reaction of **42** (29.5 mg, 0.1 mmol) and Hantzsch ester (0.30 mmol, 3.0 equiv.) in 1 mL 1,4-dioxane for 12 h.

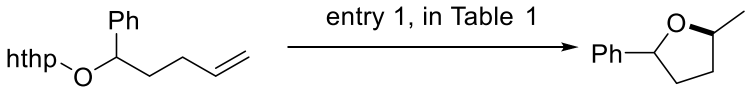

43 **44**, 62% yield

Scheme S26. 1,2-HAT Competition Experimrnts, Related to Scheme 3.

Following the standard procedure, the reaction of **46** (29.7 mg, 0.1 mmol) and Hantzsch ester (0.30 mmol, 3.0 equiv.) in 1 mL MeOH for 6 h afforded an unseperable mixture of **47** and **48** as a colorless oil after flash chromatography (90% hexanes : 10% EtOAc). Then the mixture of X and X was dissolved in 2 mL DCM and treated with Dess-Martin Periodinan (0.2 mmol, 2.0 equiv.) at room temperature for 3 hours. The saturated $Na_2S_2O_3$ aqueous solution was then added to quench the reaction, after which the mixture was washed with brine, dried over Na_2SO_4, and concentrated in vacuo. The crude product was puried by preparative thin layer chromatography (75% hexanes : 25% EtOAc).

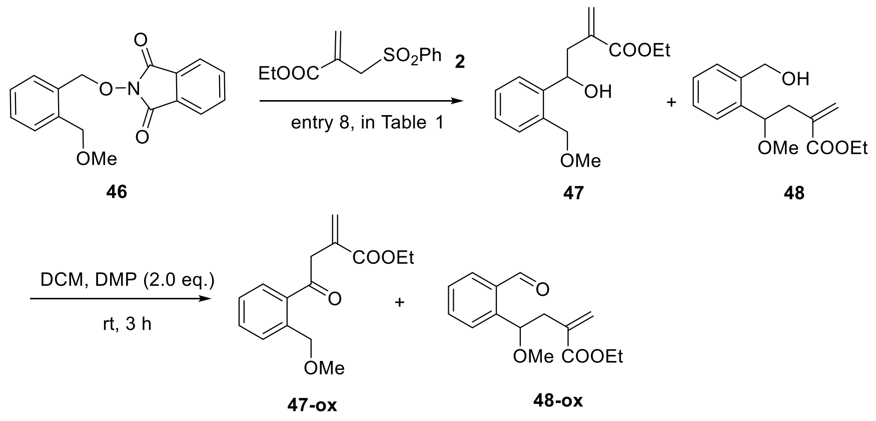

Scheme S27. 1,2-HAT Competition Experimrnts, Related to Scheme 3.

Procedure for the Luminescence Quenching Experiments

Emission intensities were recorded using Microplate Accessory 5JO-0139 spectrometer for all experiments. All *fac*-Ir(ppy)$_3$ solutions were excited at 320 nm and the emission intensity was collected at 518 nm. In a typical experiment, the 1,4-dioxane solution of *fac*-Ir(ppy)$_3$ (100 μM) was added the appropriate amount of quencher in a screw-top 1.0 cm quartz cuvette. After degassing with nitrogen for 10 min, the emission spectra of the samples were collected.

Procedure for the Cyclic Voltammetry

Cyclic Voltammetry was performed on a CH Instruments Electrochemical Workstation model CHI600E. A 0.001 M MeCN solution of the sample was prepared with 0.1 M Bu_4NPF_6 as the supporting electrolyte, using a glassy carbon working electrode, a Pt counter electrode, and a saturated calomel electrode reference electrode. Scan rate = 0.05 V/s, 2 sweep segments, a sample interval of 0.001 V.

Proceure for the Quantum Yield Measurement

n_x is the amount of photochemical or photophysical events during irradiation, n_p is the amount of photons aborbed by the reactant. n_x was calculated by NMR analysis, n_p was measured by Handy FZ-A Portable Radiometer/Photometer.

N-alkoxyphthalimides **1** (0.10 mmol, 1.0 equiv.), allyl sulfone **2**, *fac*-Ir(ppy)$_3$ (0.7 mg, 0.001 mmol, 0.01 equiv.) and Hantzsch eater (38.0 mg, 0.15 mmol, 1.5 equiv.) were placed in a 5 mL tube vial equipped with a magnetic stir bar. After 1.0 mL MeOH (treated by 4 Å MS) was added into the tube via a syringe, the reaction mixture was

exposed to blue LEDs at room temperature for 60 min and analyzed by ^{1}H-NMR

Produce for the On-Off-Light Experiments

Following the standard procedure, to a solution of **1** (105.2 mg, 0.3 mmol), **2** (232.2 mg, 0.9 mmol), *fac*-Ir(ppy)$_3$ (1.9 mg, 0.003 mmol), and HE (113.8 mg, 0.45 mmol) in MeOH (3 mL). The reaction mixture was stirred at 25 ^{o}C using 4W blue LED with on-off-light. 500 μL of the reaction mixture aliquot was collected at different points and concentrated in vacuo. The ^{1}H NMR analysis was calculated using 1,3,5-Trimethoxybenzene as the internal standard.

Proceduce for EPR studies

EPR experiments were performed on a Bruker E500 CW-EPR spectrometer at 298 K. A solution of *N*-alkoxyphthalimides **45** (0.1 mmol, 1.0 equiv.), Hantzsch ester (38.0 mg, 0.15 mmol, 1.5 equiv.) and DMPO **46** (5,5-dimethyl-pyrroline N-oxide) (13 μL 0.12 mmol, 1.2 equiv.) was placed in a 5 mL clear-colored glass vial. After 1.0 mL MeOH or dioxane (bubbled with nitrogen gas for 30 seconds to remove oxygen) was added, the vial was sealed and exposed to 4W blue LED at room temperature with stirring for 45 min. The reaction mixture was diluted 10 times and transferred to a sealed melting point tube in the glove box. The EPR signals were subsequently tested.

Computational methods for DFT caculations

All DFT calculations were performed with the GAUSSIAN 09 series of programs. Density functional B3-LYP (Becke, 1993; Lee et al., 1988) with a standard 6-31G(d) basis set was used for geometry optimizations. Harmonic frequency calculations were

performed at all stationary points to confirm them as local minima or transition structures and to derive the thermochemical corrections for the enthalpies and free energies. The DFT method M11 functional was used to calculate the single point energies in methanol and 1,4-dioxane. (Peverati and Truhlar, 2011) The solvent effects were considered by single point calculations on the gas-phase stationary points with a continuum solvation model SMD. (Cossi et al., 1996; Cances et al., 1997; Barone et al., 1998; Marenich et al., 2009; Liu et al., 2018) The larger basis set 6-311+G(d) was used in the solvation single point calculations. The energies given in this report are the M11 calculated Gibbs free energies in methanol and 1,4-dioxane solvent.

Complete reference for Gaussian 09

Frisch, M. J.; Trucks, G. W.; Schlegel, H. B.; Scuseria, G. E.; Robb, M. A.; Cheeseman, J. R.; Scalmani, G.; Barone, V.; Mennucci, B.; Petersson, G. A.; Nakatsuji, H.; Caricato, M.; Li, X.; Hratchian, H. P.; Izmaylov, A. F.; Bloino, J.; Zheng, G.; Sonnenberg, J. L.; Hada, M.; Ehara, M.; Toyota, K.; Fukuda, R.; Hasegawa, J.; Ishida, M.; Nakajima, T.; Honda, Y.; Kitao, O.; Nakai, H.; Vreven, T.; Montgomery, Jr., J. A.; Peralta, J. E.; Ogliaro, F.; Bearpark, M.; Heyd, J. J.; Brothers, E.; Kudin, K. N.; Staroverov, V. N.; Keith, T.; Kobayashi, R.; Normand, J.; Raghavachari, K.; Rendell, A.; Burant, J. C.; Iyengar, S. S.; Tomasi, J.; Cossi, M.; Rega, N.; Millam, J. M.; Klene, M.; Knox, J. E.; Cross, J. B.; Bakken, V.; Adamo, C.; Jaramillo, J.; Gomperts, R.; Stratmann, R. E.; Yazyev, O.; Austin, A. J.; Cammi, R.; Pomelli, C.; Ochterski, J. W.; Martin, R. L.; Morokuma, K.; Zakrzewski, V. G.; Voth, G. A.; Salvador, P.; Dannenberg, J. J.; Dapprich, S.; Daniels, A. D.; Farkas, O.; Foresman, J. B.; Ortiz, J. V.; Cioslowski, J.; and Fox, D. J. Gaussian 09, revision D.01; Gaussian, Inc.: Wallingford, CT, **2013**.

IV. Substrate And Product Characterizations

Characterizations of *N*-alkoxyphthalimide Precursors

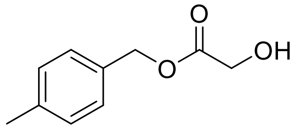

4-Methylbenzyl 2-hydroxyacetate (A1).

Colorless oil (0.62 g, 57% yield): TLC R_f = 0.43 (EtOAc/hexanes = 1/5); ^{1}H NMR (400 MHz, $CDCl_3$) δ 7.26 (d, *J* = 8.0 Hz, 2H), 7.18 (d, *J* = 8.0 Hz, 2H), 5.19 (s, 2H), 4.18 (d, *J* = 5.3 Hz, 2H), 2.36 (s, 3H); ^{13}C NMR (100 MHz, $CDCl_3$) δ 173.4, 138.8, 132.2, 129.5, 128.8, 67.4, 60.8, 21.4.

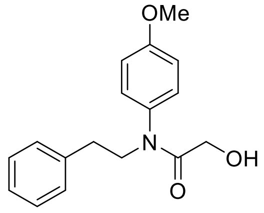

2-Hydroxy-*N*-(4-methoxyphenyl)-N-phenethylacetamide (A2).

Colorless oil (0.43 g, 62% yield): TLC R_f = 0.27 (EtOAc/hexanes = 1/2); ^{1}H NMR (500 MHz, $CDCl_3$) δ 7.29 – 7.26 (m, 2H), 7.23 – 7.16 (m, 3H), 6.96 – 6.94 (m, 2H), 6.92 – 6.89 (m, 2H), 3.96 – 3.93 (m, 2H), 3.84 (s, 3H), 3.73 (d, *J* = 4.5 Hz, 2H), 3.39 (t, *J* = 4.5 Hz, 1H, -OH), 2.91 – 2.87 (m, 2H); ^{13}C NMR (125 MHz, $CDCl_3$) δ 172.2, 159.8, 138.5, 132.3, 129.3, 129.0, 128.7, 126.6, 115.2, 60.7, 55.7, 51.4, 34.0; IR (KBr, thin film): 3436, 2933, 1655, 1512, 1386, 1250, 1030, 840, 743, 700 cm^{-1}; HRMS-ESI (m/z) [$M+Na^+$]: calcd. for $C_{17}H_{19}NNaO_3$ 308.1257, found 308.1258.

Characterizations of *N*-alkoxyphthalimide substrates

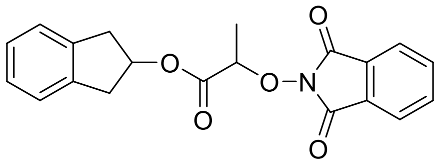

2,3-Dihydro-1*H*-inden-2-yl 2-((1,3-dioxoisoindolin-2-yl)oxy)propanoate (1).

White solid (2.36 g, 67% yield): TLC R_f = 0.46 (EtOAc/hexanes = 1/3); ^{1}H NMR (500 MHz, $CDCl_3$) δ 7.80 (dd, *J* = 5.5, 3.1 Hz, 2H), 7.74 (dd, *J* = 5.5, 3.1 Hz, 2H), 7.20 – 7.14 (m, 4H), 5.62 – 5.58 (m, 1H), 4.84 (q, *J* = 6.8 Hz, 1H), 3.37 – 3.30 (m, 2H), 3.08 – 3.02 (m, 2H), 1.61 (d, *J* = 6.8 Hz, 3H); ^{13}C NMR (125 MHz, $CDCl_3$) δ 169.8, 163.3, 140.2, 140.2, 134.7, 128.9, 126.9, 124.7, 124.7, 123.8, 81.3, 76.8, 39.5 , 39.4, 16.4; IR (KBr, thin film): 3073, 2943, 1792, 1737, 1467, 1375, 1188, 978, 747, 701 cm^{-1}; HRMS-ESI (m/z) [M+H$^+$]: calcd. for $C_{20}H_{18}NO_5$ 352.1179, found 352.1188.

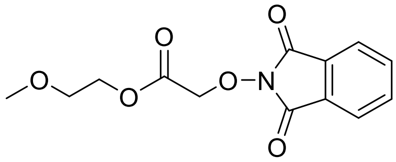

2-Methoxyethyl 2-((1,3-dioxoisoindolin-2-yl)oxy)acetate (6).

White solid (0.21 g, 27% yield): TLC R_f = 0.47 (EtOAc/hexanes = 1/4); ^{1}H NMR (500 MHz, $CDCl_3$) δ 7.85 (dd, *J* = 5.5, 3.1 Hz, 2H), 7.76 (dd, *J* = 5.5, 3.1 Hz, 2H), 4.87 (s, 2H), 4.36 – 4.34 (m, 2H), 3.63 – 3.61 (m, 2H), 3.35 (s, 3H); ^{13}C NMR (125 MHz, $CDCl_3$) δ 167.1, 163.1, 134.8, 129.0, 123.9, 73.1, 70.1, 64.6, 59.1; IR (KBr, thin film): 2987, 1793, 1733, 1276, 1260, 1187, 1130, 1054, 764, 702 cm^{-1}; HRMS-ESI (m/z) [M+Na$^+$]: calcd. for $C_{13}H_{13}NnaO_6$ 302.0635, found 302.0642.

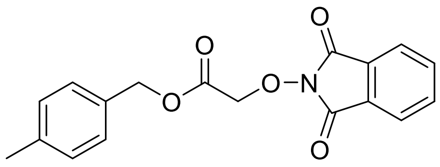

4-Methylbenzyl 2-((1,3-dioxoisoindolin-2-yl)oxy)acetate (8).

White solid (0.26 g, 24% yield over two steps): TLC R_f = 0.51 (EtOAc/hexanes = 1/4); ^{1}H NMR (500 MHz, $CDCl_3$) δ 7.84 (dd, *J* = 5.5, 3.1 Hz, 2H), 7.75 (dd, *J* = 5.5, 3.1 Hz, 2H), 7.25 (d, *J* = 7.8 Hz, 2H), 7.15 (d, *J* = 7.8 Hz, 2H), 5.19 (s, 2H), 4.84 (s, 2H), 2.34 (s, 3H); ^{13}C NMR (125 MHz, $CDCl_3$) δ 166.9, 163.1, 138.7, 134.8, 132.0, 129.4, 129.0, 128.9, 123.9, 73.2, 67.4, 21.4; IR (KBr, thin film): 3032, 2946, 1755, 1732, 1466, 1378, 1187, 1054, 878, 700 cm^{-1}; HRMS-ESI (m/z) [$M+Na^+$]: calcd. for $C_{18}H_{15}NNaO_5$ 348.0842, found 348.0844.

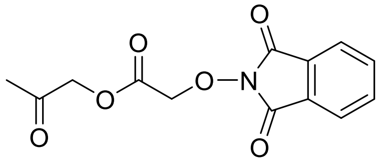

2-Oxopropyl 2-((1,3-dioxoisoindolin-2-yl)oxy)acetate (10).

White solid (0.19 g, 68% yield): TLC R_f = 0.38 (EtOAc/hexanes = 1/1); ^{1}H NMR (500 MHz, $CDCl_3$) δ 7.86 (dd, *J* = 5.5, 3.1 Hz, 2H), 7.77 (dd, *J* = 5.5, 3.1 Hz, 2H), 4.96 (s, 2H), 4.79 (s, 2H), 2.17 (s, 3H); ^{13}C NMR (125 MHz, $CDCl_3$) δ 200.2, 166.4, 163.1, 134.8, 128.9, 123.9, 72.9, 68.9, 26.1; IR (KBr, thin film): 3326, 2923, 1770, 1732, 1625, 1373, 1173, 1064, 877, 701 cm^{-1}; HRMS-ESI (m/z) [$M+Na^+$]: calcd. for $C_{13}H_{11}NNaO_6$ 300.0479, found 300.0484.

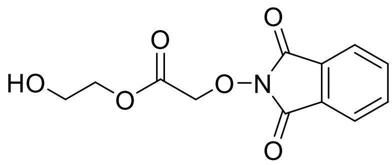

2-Hydroxyethyl 2-((1,3-dioxoisoindolin-2-yl)oxy)acetate (12).

White solid (0.24 g, 24% yield): TLC R_f = 0.44 (EtOAc/hexanes = 1/1); ^{1}H NMR (500 MHz, $CDCl_3$) δ 7.86 (dd, *J* = 5.5, 3.1 Hz, 2H), 7.78 (dd, *J* = 5.5, 3.1 Hz, 2H),

4.86 (s, 2H), 4.35 – 4.33 (m, 2H), 3.89 – 3.87 (m, 2H); ^{13}C NMR (125 MHz, $CDCl_3$) δ 167.5, 163.4, 135.0, 128.8, 124.0, 73.9, 67.6, 60.7 ; IR (KBr, thin film): 3505, 1732, 1375, 1276, 1187, 1082, 1050, 877, 750, 701 cm^{-1}; HRMS-ESI (m/z) [M+Na$^+$]: calcd. for $C_{12}H_{11}NO_6$ 288.0479, found 288.0478.

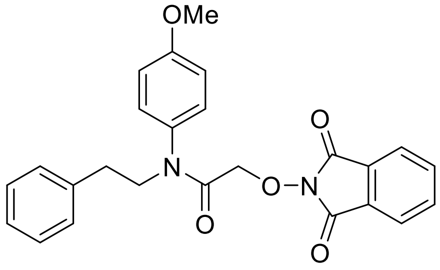

2-((1,3-Dioxoisoindolin-2-yl)oxy)-*N*-(4-methoxyphenyl)-N-phenethylacetamide (14).

White solid (0.46 g, 69% yield): TLC R_f = 0.24 (EtOAc/hexanes = 1/2); ^{1}H NMR (500 MHz, $CDCl_3$) δ 7.82 (dd, *J* = 5.5, 3.1 Hz, 2H), 7.73 (dd, *J* = 5.5, 3.1 Hz, 2H), 7.24 (t, *J* = 7.6 Hz, 2H), 7.19 – 7.12 (m, 5H), 6.91 (d, *J* = 8.8 Hz, 2H), 4.51 (s, 2H), 3.91 – 3.88 (m, 2H), 3.83 (s, 3H), 2.91 – 2.88 (m, 2H); ^{13}C NMR (125 MHz, $CDCl_3$) δ 165.8, 163.2, 159.6, 138.7, 134.6, 133.3, 129.5, 129.1, 129.0, 128.5, 126.4, 123.7, 115.2, 73.8, 55.6, 51.2, 33.7; IR (KBr, thin film): 3058, 2931, 1734, 1676, 1511, 1375, 1249, 1185, 877, 700 cm^{-1}; HRMS-ESI (m/z) [M+Na$^+$]: calcd. for $C_{25}H_{22}N_2NaO_5$ 453.1421, found 453.1423.

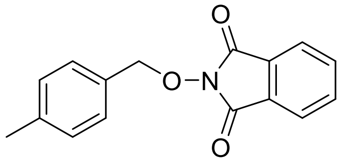

2-((4-Methylbenzyl)oxy)isoindoline-1,3-dione (16).

White solid (2.23 g, 83% yield): TLC R_f = 0.43 (EtOAc/hexanes = 1/4); ^{1}H NMR (500 MHz, $CDCl_3$) δ 7.81 (dd, *J* = 5.5, 3.1 Hz, 2H), 7.73 (dd, *J* = 5.5, 3.1 Hz, 2H), 7.42 (d, *J* = 8.0 Hz, 2H), 7.18 (d, *J* = 8.0 Hz, 2H), 5.17 (s, 2H), 2.35 (s, 3H); ^{13}C NMR (125 MHz, $CDCl_3$) δ 163.6, 139.4, 134.5, 130.8, 130.1, 129.4, 129.0, 123.6, 79.8, 21.5; IR (KBr, thin film): 3093, 1785, 1737, 1466, 1391, 1186, 1142, 975, 880, 699

cm^{-1}; HRMS-ESI (m/z) [M+Na$^+$]: calcd. for $C_{16}H_{13}NNaO_3$ 290.0788, found 290.0795.

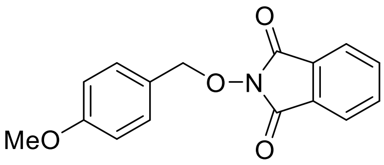

2-((4-Methoxybenzyl)oxy)isoindoline-1,3-dione (18).

White solid (2.30 g, 81% yield): TLC R_f = 0.27 (EtOAc/hexanes = 1/4); ^{1}H NMR (500 MHz, $CDCl_3$) δ 7.80 (dd, *J* = 5.5, 3.1 Hz, 2H), 7.72 (dd, *J* = 5.5, 3.1 Hz, 2H), 7.47 – 7.44 (m, 2H), 6.90 – 6.87 (m, 2H), 5.15 (s, 2H), 3.80 (s, 3H); ^{13}C NMR (125 MHz, $CDCl_3$) δ 163.7, 160.6, 134.5, 131.8, 129.0, 126.0, 123.6, 114.1, 79.6, 55.4; IR (KBr, thin film): 2963, 1786, 1736, 1612, 1515, 1392, 1253, 1143, 879, 698 cm^{-1}; HRMS-ESI (m/z) [M+Na$^+$]: calcd. for $C_{16}H_{13}NNaO_4$ 306.0737, found 306.0744.

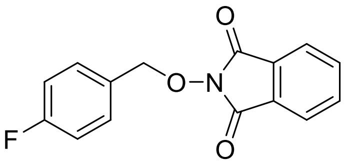

2-(4-fluorobenzyloxy)isoindoline-1,3-dione (20).

White solid (0.85 g, 31% yield): TLC R_f = 0.57 (EtOAc/hexanes = 1/5); ^{1}H NMR (400 MHz, $CDCl_3$) δ 7.82 (dd, *J* = 5.5, 3.1 Hz, 2H), 7.74 (dd, *J* = 5.5, 3.1 Hz, 2H), 7.52 (dd, *J* = 8.6, 5.4 Hz, 2H), 7.06 (t, *J* = 8.6 Hz, 2H), 5.18 (s, 2H); ^{13}C NMR (125 MHz, $CDCl_3$) δ 164.5, 163.6, 162.5, 134.6, 132.1, 132.0, 129.8, 129.8, 128.9, 123.7, 115.8, 115.6, 79.1; ^{19}F NMR (376 MHz, $CDCl_3$) δ -111.9 (m); IR (KBr, thin film): 3647, 3098, 1726, 1466, 1389, 1187, 1127, 975, 878, 696 cm^{-1}; HRMS-ESI (m/z) [M+NH_4^+]: calcd. for $C_{15}H_{14}FN_2O_3$ 289.0983, found 289.0987.

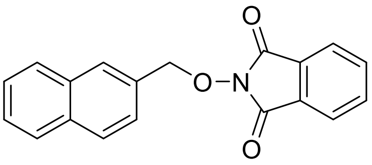

2-(Naphthalen-2-ylmethoxy)isoindoline-1,3-dione (22).

White solid (1.33 g, 88% yield): TLC R_f = 0.42 (EtOAc/hexanes = 1/4); ^{1}H NMR (500 MHz, $CDCl_3$) δ 7.93 (s, 1H), 7.88 (d, *J* = 8.4 Hz, 1H), 7.84 (dd, *J* = 6.5, 3.0 Hz, 2H), 7.79 (dd, *J* = 5.5, 3.1 Hz, 2H), 7.74 – 7.70 (m, 3H), 7.52 – 7.46 (m, 2H), 5.38 (s, 2H); ^{13}C NMR (125 MHz, $CDCl_3$) δ 163.7, 134.6, 133.8, 133.1, 131.4, 129.5, 129.0, 128.6, 128.3, 127.9, 127.2, 126.8, 126.4, 123.6, 80.1; IR (KBr, thin film): 3093, 1739, 1466, 1383, 1275, 1261, 1139, 972, 750, 699 cm^{-1}; HRMS-ESI (m/z) [M+Na$^+$]: calcd. for $C_{19}H_{13}NNaO_3$ 326.0788, found 326.0793.

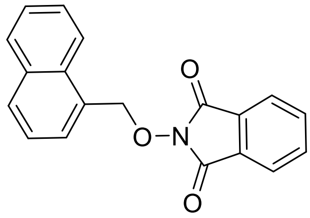

2-(Naphthalen-1-ylmethoxy)isoindoline-1,3-dione (24).

White solid (0.83 g, 54% yield): TLC R_f = 0.36 (EtOAc/hexanes = 1/4); ^{1}H NMR (500 MHz, $CDCl_3$) δ 8.63 (dd, *J* = 8.5, 1.1 Hz, 1H), 7.90 – 7.87 (m, 2H), 7.82 (dd, *J* = 5.5, 3.1 Hz, 2H), 7.72 (dd, *J* = 5.5, 3.1 Hz, 2H), 7.67 (ddd, *J* = 8.4, 6.8, 1.3 Hz, 1H), 7.61 (dd, *J* = 7.0, 1.1 Hz, 1H), 7.54 (ddd, *J* = 8.0, 6.8, 1.1 Hz, 1H), 7.43 (dd, *J* = 8.4, 6.8 Hz, 1H), 5.64 (s, 2H); ^{13}C NMR (125 MHz, $CDCl_3$) δ 163.7, 134.6, 133.8, 132.6, 130.7, 129.7, 129.6, 129.1, 128.6, 127.1, 126.3, 125.2, 124.6, 123.6, 78.2; IR (KBr, thin film): 3043, 2896, 1786, 1729, 1383, 1186, 1131, 971, 877, 699 cm^{-1}; HRMS-ESI (m/z) [M+Na$^+$]: calcd. for $C_{19}H_{13}NNaO_3$ 326.0788, found 326.0795.

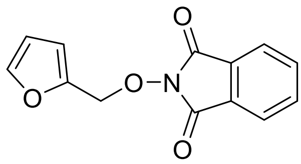

2-(Furan-2-ylmethoxy)isoindoline-1,3-dione (26).

White solid (0.70 g, 29% yield): TLC R_f = 0.50 (EtOAc/hexanes = 1/4); ^{1}H NMR (500 MHz, $CDCl_3$) δ 7.81 (dd, *J* = 5.5, 3.1 Hz, 2H), 7.74 (dd, *J* = 5.5, 3.1 Hz, 2H),

7.48 – 7.47 (m, 1H), 6.49 (dd, *J* = 3.3, 0.8 Hz, 1H), 6.35 (dd, *J* = 3.3, 1.8 Hz, 1H), 5.16 (s, 2H); ^{13}C NMR (125 MHz, $CDCl_3$) δ 163.4, 148.1, 144.6, 134.6, 128.9, 123.6, 113.3, 110.9, 70.4; IR (KBr, thin film): 3123, 1786, 1742, 1380, 1185, 1137, 976, 925, 759, 700 cm^{-1}; HRMS-ESI (m/z) [M+H$^+$]: calcd. for $C_{13}H_{10}NO_4$ 244.0604, found 244.0607.

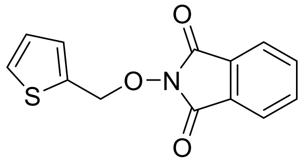

2-(Thiophen-2-ylmethoxy)isoindoline-1,3-dione (28).

White solid (0.61 g, 23% yield): TLC R_f = 0.49 (EtOAc/hexanes = 1/4); ^{1}H NMR (500 MHz, $CDCl_3$) δ 7.81 (dd, *J* = 5.5, 3.1 Hz, 2H), 7.74 (dd, *J* = 5.5, 3.1 Hz, 2H), 7.40 (dd, *J* = 5.1, 1.2 Hz, 1H), 7.20 – 7.19 (m, 1H), 6.99 (dd, *J* = 5.1, 3.5 Hz, 1H), 5.38 (s, 2H); ^{13}C NMR (125 MHz, $CDCl_3$) δ 163.6, 135.5, 134.6, 130.5, 129.0, 128.7, 127.2, 123.7, 73.1; IR (KBr, thin film): 3102, 3040, 1719, 1465, 1386, 1185, 1132, 1084, 875, 699 cm^{-1}; HRMS-ESI (m/z) [M+Na$^+$]: calcd. for $C_{13}H_9NNaO_3S$ 282.0195, found 282.0202.

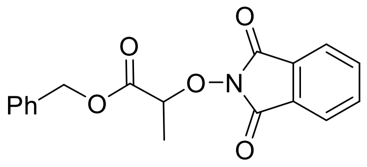

Benzyl 2-((1,3-dioxoisoindolin-2-yl)oxy)propanoate (30).

White solid (0.57 g, 38 % yield): TLC R_f = 0.35 (EtOAc/hexanes = 1/4); ^{1}H NMR (500 MHz, $CDCl_3$) δ 7.82 (dd, *J* = 5.5, 3.1 Hz, 2H), 7.74 (dd, *J* = 5.5, 3.1 Hz, 2H), 7.37 – 7.27 (m, 5H), 5.26 – 5.14 (m, 2H), 4.92 (q, *J* = 6.8 Hz, 1H), 1.66 (d, *J* = 6.8 Hz, 3H); ^{13}C NMR (125 MHz, $CDCl_3$) δ 169.7, 163.4, 135.2, 134.7, 129.0, 128.7, 128.6, 128.6, 123.8, 81.4, 67.5, 16.4; IR (KBr, thin film): 3446, 1734, 1620, 1384, 1187, 1080, 977, 877, 698, 664 cm^{-1}; HRMS-ESI (m/z) [M+H$^+$]: calcd. for $C_{18}H_{16}NO_5$ 326.1023, found 326.1021.

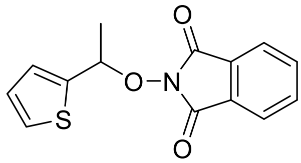

2-(1-(thiophen-2-yl)ethoxy)isoindoline-1,3-dione (32).

White solid (2.10 g, 77 % yield): TLC R_f = 0.52 (EtOAc/hexanes = 1/4): ^{1}H NMR (500 MHz, $CDCl_3$) δ 7.78 (dd, *J* = 5.5, 3.1 Hz, 2H), 7.72 (dd, *J* = 5.5, 3.1 Hz, 2H), 7.35 (d, *J* = 5.0 Hz, 1H), 7.11 (d, *J* = 4.4 Hz, 1H), 6.93 (dd, *J* = 5.0, 3.6 Hz, 1H), 5.68 (q, *J* = 6.5 Hz, 1H), 1.85 (d, *J* = 6.5 Hz, 3H); ^{13}C NMR (125 MHz, $CDCl_3$) δ 163.8, 141.8, 134.5, 128.9, 127.9, 127.2, 126.7, 123.6, 80.0, 20.7; IR (KBr, thin film): 1789, 1831, 1466, 1373, 1186, 1128, 1081, 971, 878, 699 cm^{-1}; HRMS-ESI (m/z) [M+Na$^+$]: calcd. for $C_{14}H_{11}NNaO_3S$ 296.0352, found 296.0357.

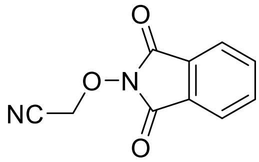

2-((1,3-Dioxoisoindolin-2-yl)oxy)acetonitrile (34).

White solid (1.38 g, 68% yield): TLC R_f = 0.29 (EtOAc/hexanes = 1/2); ^{1}H NMR (500 MHz, $CDCl_3$) δ 7.90 (dd, *J* = 5.5, 3.1 Hz, 2H), 7.82 (dd, *J* = 5.5, 3.1 Hz, 2H), 4.96 (s, 2H); ^{13}C NMR (125 MHz, $CDCl_3$) δ 162.8, 135.3, 128.7, 124.3, 113.8, 62.0; IR (KBr, thin film): 2993, 1793, 1735, 1362, 1276, 1187, 1021, 876, 750, 701 cm^{-1}; HRMS-ESI (m/z) [M+Na$^+$]: calcd. for $C_{10}H_6N_2NaO_3$ 225.0271, found 225.0278.

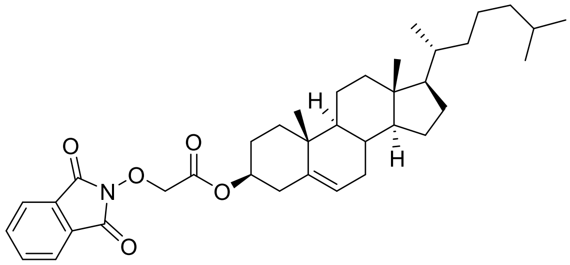

(3S,9S,10R,13R,14S,17R)-10,13-dimethyl-17-(6-methylheptan-2-yl)-2,3,4,7,8,9,10,

11,12,13,14,15,16,17-tetradecahydro-1H-cyclopenta[a]phenanthren-3-yl 2-((1,3-dioxoisoindolin-2-yl)oxy)acetate (40).

White solid (0.73 g, 62 % yield): TLC R_f = 0.48 (EtOAc/hexanes = 1/4): ^{1}H NMR (500 MHz, $CDCl_3$) δ 7.86 (dd, *J* = 5.4, 3.1 Hz, 2H), 7.76 (dd, *J* = 5.4, 3.1 Hz, 2H), 5.37 (d, *J* = 4.2 Hz, 1H), 4.80 (s, 2H), 4.74 (dtd, *J* = 12.5, 8.0, 4.3 Hz, 1H), 2.36 (d, *J* = 7.8 Hz, 2H), 2.04 – 1.78 (m, 5H), 1.65 (ddd, *J* = 14.0, 8.7, 2.9 Hz, 1H), 1.49 (dtdd, *J* = 37.3, 16.2, 12.4, 10.3 Hz, 6H), 1.39 – 1.30 (m, 3H), 1.24 (ddd, *J* = 13.2, 9.8, 3.1 Hz, 1H), 1.20 – 1.03 (m, 7H), 1.03 – 0.94 (m, 6H), 0.91 (d, *J* = 6.5 Hz, 3H), 0.86 (dd, *J* = 6.6, 2.3 Hz, 6H), 0.67 (s, 3H); ^{13}C NMR (125 MHz, $CDCl_3$) δ 166.5, 163.1, 139.3, 134.8, 129.0, 123.8, 123.2, 75.8, 73.3, 56.8, 56.3, 50.1, 42.4, 39.8, 39.7, 38.0, 37.0, 36.7, 36.3, 35.9, 32.0, 32.0, 28.4, 28.2, 27.7, 24.4, 24.0, 23.0, 22.7, 21.2, 19.4, 18.9, 12.0. IR (KBr, thin film): 3445, 2944, 1731, 1466, 1383, 1188, 1135, 1052, 877, 696 cm^{-1}; HRMS-ESI (m/z) [M+Na$^+$]: calcd. for $C_{37}H_{51}NNaO_5$ 612.3659, found 612.3665.

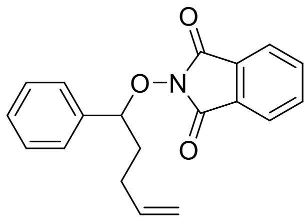

2-((1-Phenylpent-4-en-1-yl)oxy)isoindoline-1,3-dione (43).

White solid (1.53 g, 62% yield): TLC R_f = 0.51 (EtOAc/hexanes = 1/4); ^{1}H NMR (500 MHz, $CDCl_3$) δ 7.71 (dd, *J* = 5.3, 3.2 Hz, 2H), 7.66 (dd, *J* = 5.3, 3.2 Hz, 2H), 7.46 (d, *J* = 6.4 Hz, 2H), 7.31 (q, *J* = 5.9 Hz, 3H), 5.85 (ddt, *J* = 16.8, 10.2, 6.5 Hz, 1H), 5.34 (t, *J* = 6.9 Hz, 1H), 5.03 (dd, *J* = 26.7, 13.7 Hz, 2H), 2.29 (ddd, *J* = 13.1, 8.6, 6.7 Hz, 1H), 2.23 – 2.15 (m, 2H), 2.00 (dq, *J* = 14.8, 6.8 Hz, 1H); ^{13}C NMR (125 MHz, $CDCl_3$) δ 163.8, 138.1, 137.5, 134.4, 129.1, 128.9, 128.4, 128.2, 123.4, 115.5, 88.7, 34.1, 29.9.

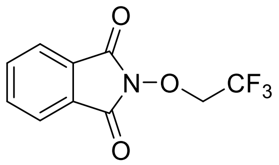

2-(2,2,2-trifluoroethoxy)isoindoline-1,3-dione(37).

White solid (1.64 g, 67% yield): TLC R_f = 0.53 (EtOAc/hexanes = 1/4);^{1}H NMR (500 MHz, $CDCl_3$) δ 7.86 (dd, *J* = 5.5, 3.1 Hz, 2H), 7.78 (dd, *J* = 5.4, 3.1 Hz, 2H), 4.55 (q, *J* = 8.0 Hz, 2H). ^{13}C NMR (126 MHz, $cdcl_3$) δ 162.5, 134.9, 128.6, 123.9, 73.5, 73.2, 72.9, 72.7. ^{19}F NMR (376 MHz, $CDCl_3$) δ -73.94 (t, *J* = 8.0 Hz).

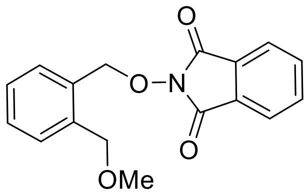

2-((2-(methoxymethyl)benzyl)oxy)isoindoline-1,3-dione. (46)

White solid (1.40 g, 47% yield): TLC R_f = 0.43 (EtOAc/hexanes = 1/4); ^{1}H NMR (500 MHz, $CDCl_3$) δ 7.81 (dd, *J* = 5.5, 3.0 Hz, 2H), 7.73 (dd, *J* = 5.5, 3.1 Hz, 2H), 7.50 – 7.46 (m, 1H), 7.45 – 7.42 (m, 1H), 7.38 (td, *J* = 7.5, 1.3 Hz, 1H), 7.30 (td, *J* = 7.5, 1.4 Hz, 1H), 5.31 (s, 2H), 4.79 (s, 2H), 3.45 (s, 3H). ^{13}C NMR (126 MHz, $CDCl_3$) δ 163.5 138.4 134.4, 131.9, 131.6 129.7 129.3, 128.9, 127.9, 123.6, 77.2 72.2, 58.4; IR (KBr, thin film): 1731, 1464, 1386, 1184, 1136, 1087, 928, 877, 753 cm^{-1}; HRMS-ESI (m/z) [$M+NH_4^+$]: calcd. for $C_{17}H_{16}N_2O_4$ 315.1339, found 315.1339.

Characterization of Allyl Sulfones

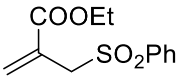

Ethyl-2-((phenylsulfonyl)methyl)acrylate (2).

Viscous oil (1.94 g, 74% yield): TLC R_f = 0.50 (EtOAc/hexanes = 1/4); ^{1}H NMR (400 MHz, $CDCl_3$) δ 7.86 (d, *J* = 7.6 Hz, 2H), 7.64 (t, *J* = 7.6 Hz, 1H), 7.54 (t, *J* = 7.6 Hz, 2H), 6.51 (s, 1H), 5.92(s, 1H), 4.17(s, 2H), 4.01(q, *J* = 7.2 Hz, 2H), 1.17(t, *J* = 7.2 Hz, 3H); ^{13}C NMR (100 MHz, $CDCl_3$) δ 164.7, 138.4, 133.9, 133.3, 129.1, 129.0, 128.8, 61.5, 57.5, 14.0.

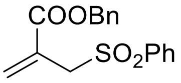

Benzyl-2-((phenylsulfonyl)methyl)acrylate (2-a).

White solid (1.3 g, 20% yield over three steps): TLC R_f = 0.49 (EtOAc/hexanes = 1/4); ^{1}H NMR (400 MHz, $CDCl_3$) δ 7.82 (d, *J* = 7.2 Hz, 2H), 7.59 (t, *J* = 6.9 Hz, 1H), 7.46 (t, *J* = 7.9 Hz, 2H), 7.42 – 7.15 (m, 5H), 6.54 (s, 1H), 5.94 (s, 1H), 4.99 (s, 2H), 4.16 (s, 2H); ^{13}C NMR (125 MHz, $CDCl_3$) δ 164.7, 138.4, 135.4, 134.0, 133.9, 129.1, 129.0, 128.8, 128.7, 128.5, 128.3, 67.3, 57.6; IR (KBr, thin film) 2938, 1721, 1447, 1309, 1246, 1177, 1145, 1084, 967, 750, 689 cm^{-1}; HRMS-ESI (m/z) $[M+H^+]$: calcd. for $C_{17}H_{17}O_4S$ 317.0841, found 317.0842.

Characterization of Allylation Product

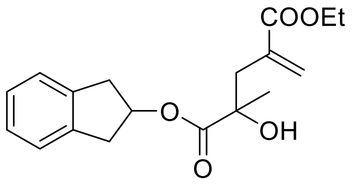

1-(2,3-Dihydro-1*H*-inden-2-yl) 5-ethyl 2-hydroxy-2-methyl-4-methylenepentane-dioate (4).

Colorless oil (29.5 mg, 93% yield) after flash chromatography (95% hexanes : 5% EtOAc): TLC R_f = 0.48 (EtOAc/hexanes = 1/4); ^{1}H NMR (500 MHz, $CDCl_3$) δ 7.24 – 7.18 (m, 4H), 6.22 (d, *J* = 1.5 Hz, 1H), 5.60 (d, *J* = 1.3 Hz, 1H), 5.60 – 5.50 (m, 1H), 4.17 (qd, *J* = 7.1, 5.6 Hz, 2H), 3.64 (s, 1H, -OH), 3.37 – 3.31 (m, 2H), 3.08 – 2.98 (m, 2H), 2.80 (dd, *J* = 14.0, 1.0 Hz, 1H), 2.64 (dd, *J* = 14.0, 1.0 Hz, 1H), 1.40 (s, 3H), 1.28 (t, *J* = 7.1 Hz, 3H); ^{13}C NMR (125 MHz, $CDCl_3$) δ 175.9, 167.9, 140.2, 140.2, 136.0, 128.9, 127.0, 127.0, 124.7, 74.3, 61.2, 41.8, 39.7, 39.5, 25.9, 14.2; IR (KBr, thin film): 3502, 2981, 1720, 1628, 1483, 1370, 1177, 1024, 965, 743 cm^{-1}; HRMS-ESI (m/z) [$M+Na^+$]: calcd. for $C_{18}H_{22}NaO_5$ 341.1359, found 341.1360.

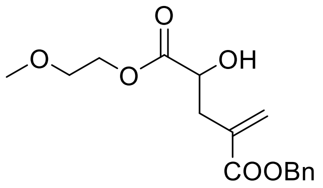

1-Benzyl 5-(2-methoxyethyl) 4-hydroxy-2-methylenepentanedioate (7).

Light yellow oil (27.5 mg, 89% yield) after flash chromatography (95% DCM : 5% EtOAc): TLC R_f = 0.14 (EtOAc/DCM = 1/10); ^{1}H NMR (500 MHz, $CDCl_3$) δ 7.38 – 7.31 (m, 5H), 6.35 (d, *J* = 1.2 Hz, 1H), 5.77 (d, *J* = 1.2 Hz, 1H), 5.24 – 5.18 (m, 2H), 4.47 – 4.43 (m, 1H), 4.34 – 4.25 (m, 2H), 3.59 (t, *J* = 4.7 Hz, 2H), 3.36 (s, 3H), 3.03 (d, *J* = 6.5 Hz, 1H, -OH), 2.91 – 2.87 (m, 1H), 2.72 – 2.67 (m, 1H); ^{13}C NMR (125 MHz, $CDCl_3$) δ 174.3, 167.0, 135.9, 135.6, 129.1, 128.7, 128.4, 128.2, 70.3, 69.6,

66.9, 64.6, 59.1, 37.2; IR (KBr, thin film): 3492, 2930, 1720, 1630, 1456, 1272, 1140, 1039, 742, 699 cm^{-1}; HRMS-ESI (m/z) [M+Na$^+$]: calcd. for $C_{16}H_{20}NaO_6$ 331.1152, found 331.1158.

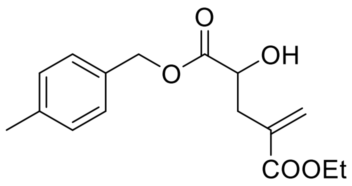

1-Ethyl 5-(4-methylbenzyl) 4-hydroxy-2-methylenepentanedioate (9).

Light yellow oil (26.8 mg, 92% yield) after preparative thin layer chromatography separation (75% hexanes : 12.5% acetone : 12.5% EtOAc): TLC R_f = 0.49 (DCM /acetone/hexanes = 1/1/6); ^{1}H NMR (500 MHz, $CDCl_3$) δ 7.25 (d, *J* = 7.8 Hz, 2H), 7.17 (d, *J* = 7.8 Hz, 2H), 6.24 (d, *J* = 1.3 Hz, 1H), 5.64 (d, *J* = 1.2 Hz, 1H), 5.19 – 5.12 (m, 2H), 4.44 – 4.40 (m, 1H), 4.20 (qd, *J* = 7.1, 2.4 Hz, 2H), 3.09 (d, *J* = 6.6 Hz, 1H, -OH), 2.87 – 2.83 (m, 1H), 2.67 – 2.62 (m, 1H), 2.36 (s, 3H), 1.29 (t, *J* = 7.1 Hz, 3H); ^{13}C NMR (125 MHz, $CDCl_3$) δ 174.2, 167.2, 138.6, 135.8, 132.3, 129.4, 128.7, 128.5, 69.8, 67.4, 61.2, 37.3, 21.3, 14.3; IR (KBr, thin film): 3487, 2981, 1716, 1632, 1447, 1206, 1148, 1098, 1031, 808 cm^{-1}; HRMS-ESI (m/z) [M+Na$^+$]: calcd. for $C_{16}H_{20}NaO_5$ 315.1203, found 315.1205.

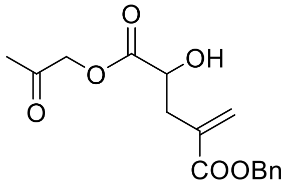

1-Benzyl 5-(2-oxopropyl) (*S*)-4-hydroxy-2-methylenepentanedioate (11).

Light yellow oil (26.8 mg, 88% yield) after flash chromatography (95% DCM : 5% EtOAc): TLC R_f = 0.15 (EtOAc/DCM = 1/10); ^{1}H NMR (500 MHz, $CDCl_3$) δ 7.39 – 7.32 (m, 5H), 6.37 (d, *J* = 1.2 Hz, 1H), 5.82 (d, *J* = 1.1 Hz, 1H), 5.24 – 5.18 (m, 2H), 4.69 (d, *J* = 1.3 Hz, 2H), 4.56 – 4.52 (m, 1H), 3.06 (d, *J* = 6.4 Hz, 1H, -OH), 3.00 – 2.96 (m, 1H), 2.77 – 2.72 (m, 1H), 2.16 (s, 3H); ^{13}C NMR (125 MHz, $CDCl_3$) δ 200.5, 173.5, 167.1, 135.9, 135.3, 129.6, 128.7, 128.4, 128.3, 69.7, 68.9, 67.0, 37.4, 26.2; IR

(KBr, thin film): 3473, 2929, 1719, 1633, 1423, 1274, 1171, 960, 742, 699 cm^{-1}; HRMS-ESI (m/z) [M+Na$^+$]: calcd. for $C_{16}H_{18}NaO_6$ 329.0996, found 329.0998.

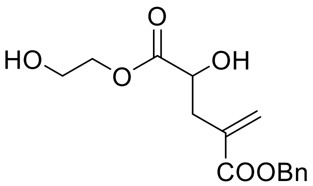

1-Benzyl 5-(2-hydroxyethyl) 4-hydroxy-2-methylenepentanedioate (13).

Light yellow oil (27.9 mg, 95% yield) after flash chromatography (50% hexanes : 50% EtOAc): TLC R_f = 0.32 (EtOAc/hexanes = 2/1); ^{1}H NMR (500 MHz, $CDCl_3$) δ 7.38 – 7.33 (m, 5H), 6.36 (d, *J* = 1.3 Hz, 1H), 5.80 (d, *J* = 1.1 Hz, 1H), 5.21 (s, 2H), 4.44 (td, *J* = 6.5, 5.5 Hz, 1H), 4.35 – 4.30 (m, 1H), 4.21 – 4.17 (m, 1H), 3.79 (q, *J* = 4.9 Hz, 2H), 3.02 (d, *J* = 6.7 Hz, 1H, -OH), 2.87 – 2.79 (m, 2H), 2.32 (d, *J* = 5.9 Hz, 1H, -OH); ^{13}C NMR (125 MHz, $CDCl_3$) δ 174.5, 167.3, 135.7, 135.1, 129.8, 128.8, 128.5, 128.3, 69.5, 67.6, 67.2, 60.9, 37.1; IR (KBr, thin film): 3400, 2954, 1719, 1632, 1455, 1210, 1145, 1083, 748, 699 cm^{-1}; HRMS-ESI (m/z) [M+Na$^+$]: calcd. for $C_{15}H_{18}NaO_6$ 317.0996, found 317.0996.

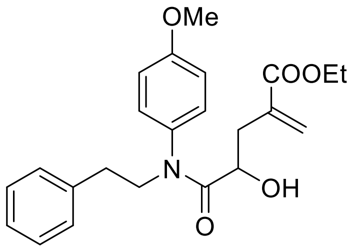

Ethyl 4-hydroxy-5-((4-methoxyphenyl)(phenethyl)amino)-2-methylene-5-oxopentanoate (15).

Light yellow oil (20.7 mg, 52% yield) after flash chromatography (70% hexanes : 30% EtOAc): TLC R_f = 0.29 (EtOAc/hexanes = 1/2); ^{1}H NMR (500 MHz, $CDCl_3$) δ 7.28 – 7.25 (m, 2H), 7.21 – 7.17 (m, 3H), 7.11 (d, *J* = 8.6 Hz, 2H), 6.92 (d, *J* = 8.6 Hz, 2H), 6.20 (d, *J* = 1.5 Hz, 1H), 5.54 (d, *J* = 1.5 Hz, 1H), 4.25 – 4.21 (m, 1H), 4.11 – 4.04 (m, 2H), 4.02 – 3.98 (m, 1H), 3.84 (s, 3H), 3.78 – 3.73 (m, 1H), 3.31 (d, *J* = 9.3

Hz, 1H, -OH), 2.93 – 2.81 (m, 2H), 2.46 (dd, *J* = 13.9, 7.0 Hz, 1H), 2.36 (dd, *J* = 13.9, 4.4 Hz, 1H), 1.21 (t, *J* = 7.1 Hz, 3H); ^{13}C NMR (125 MHz, $CDCl_3$) δ 173.8, 166.8, 159.5, 138.7, 135.5, 133.6, 129.7, 129.0, 128.6, 128.4, 126.5, 115.0, 67.1, 60.8, 55.6, 52.0, 37.1, 33.8, 14.3; IR (KBr, thin film): 3417, 2934, 1714, 1650, 1512, 1455, 1249, 1145, 1029, 701 cm^{-1}; HRMS-ESI (m/z) [M+Na$^+$]: calcd. for $C_{23}H_{27}NNaO_5$ 420.1781, found 420.1786.

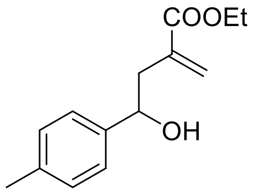

Ethyl 4-hydroxy-2-methylene-4-(p-tolyl)butanoate (17).

Light yellow oil (15.5 mg, 66% yield) after flash chromatography (90% hexanes : 10% acetone): TLC R_f = 0.36 (EtOAc/hexanes = 1/3); ^{1}H NMR (500 MHz, $CDCl_3$) δ 7.25 (d, *J* = 7.8 Hz, 2H), 7.15 (d, *J* = 7.8 Hz, 2H), 6.24 (d, *J* = 1.5 Hz, 1H), 5.60 (d, *J* = 1.3 Hz, 1H), 4.86 (dt, *J* = 8.1, 3.7 Hz, 1H), 4.23 (qd, *J* = 7.1, 1.0 Hz, 2H), 2.80 – 2.76 (m, 1H), 2.69 – 2.65 (m, 1H), 2.54 (d, *J* = 3.5 Hz, 1H, -OH), 2.34 (s, 3H), 1.32 (t, *J* = 7.1 Hz, 3H); ^{13}C NMR (125 MHz, $CDCl_3$) δ 167.8, 141.2, 137.4, 137.3, 129.2, 128.2, 125.8, 73.2, 61.2, 42.6, 21.3, 14.3; IR (KBr, thin film): 3486, 2981, 1713, 1631, 1370, 1305, 1192, 1144, 1028, 816 cm^{-1}; HRMS-ESI (m/z) [M+Na$^+$]: calcd. for $C_{14}H_{18}NaO_3$ 257.1148, found 257.1151.

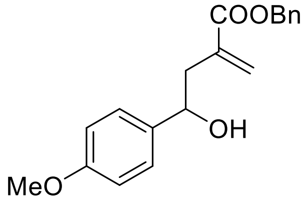

Benzyl 4-hydroxy-4-(4-methoxyphenyl)-2-methylenebutanoate (19).

Light yellow oil (22.1 mg, 71% yield) after preparative thin layer chromatography separation (75% hexanes : 12.5% acetone : 12.5% DCM): TLC R_f = 0.30 (DCM/acetone/hexanes = 1/1/6); ^{1}H NMR (500 MHz, $CDCl_3$) δ 7.38 – 7.33 (m, 5H), 7.27 – 7.24 (m, 2H), 6.87 – 6.84 (m, 2H), 6.28 (d, *J* = 1.4 Hz, 1H), 5.62 (d, *J* = 1.2 Hz,

1H), 5.21 (d, *J* = 3.2 Hz, 2H), 4.84 (dt, *J* = 8.0, 3.8 Hz, 1H), 3.79 (s, 3H), 2.79 – 2.75 (m, 1H), 2.72 – 2.67 (m, 1H), 2.43 (d, *J* = 3.3 Hz, 1H, -OH); ^{13}C NMR (125 MHz, $CDCl_3$) δ 167.5, 159.2, 137.1, 136.2, 136.0, 128.7, 128.4, 128.3, 127.1, 113.9, 72.9, 66.9, 55.4, 42.6; IR (KBr, thin film): 3485, 2955, 1715, 1513, 1456, 1177, 1034, 832, 739, 698 cm^{-1}; HRMS-ESI (m/z) [M+Na$^+$]: calcd. for $C_{19}H_{20}NaO_4$ 335.1254, found 335.1258.

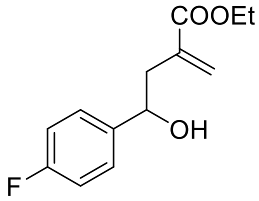

Ethyl 4-(4-fluorophenyl)-4-hydroxy-2-methylenebutanoate (21).

Light yellow oil (12.3 mg, 52% yield) after preparative thin layer chromatography separation (80% hexanes : 20% EtOAc): TLC R_f = 0.52 (EtOAc/hexanes = 1/5); ^{1}H NMR (500 MHz, $CDCl_3$) δ 7.36 – 7.30 (m, 2H), 7.06 – 6.99 (m, 2H), 6.23 (d, *J* = 1.5 Hz, 1H), 5.58 (q, *J* = 1.2 Hz, 1H), 4.88 (dd, *J* = 8.3, 4.0 Hz, 1H), 4.23 (qd, *J* = 7.1, 0.9 Hz, 2H), 2.81 (s, 1H), 2.76 (ddd, *J* = 13.9, 4.2, 1.1 Hz, 1H), 2.64 (ddd, *J* = 14.1, 8.4, 0.9 Hz, 1H), 1.32 (t, *J* = 7.2 Hz, 3H); ^{13}C NMR (125 MHz, $CDCl_3$) δ 167.9, 163.2, 161.3, 139.8, 139.8, 137.1, 128.5, 127.5, 127.5, 115.4, 115.2, 72.7, 61.3, 42.8, 14.3; ^{19}F NMR (376 MHz, $CDCl_3$) δ -115.4 (m); IR (KBr, thin film): 3081, 2955, 1730, 1574, 1451, 1189, 1065, 952, 750, 661 cm^{-1}; HRMS-ESI (m/z) [M-H_2O+H$^+$]: calcd. for $C_{13}H_{13}FO_2$ 221.0972, found 221.0976.

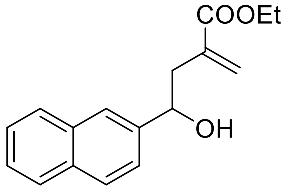

Ethyl 4-hydroxy-2-methylene-4-(naphthalen-2-yl)butanoate (23).

Light yellow oil (19.8 mg, 73% yield) after preparative thin layer chromatography separation (80% hexanes : 20% EtOAc): TLC R_f = 0.36 (EtOAc/hexanes = 1/4); ^{1}H

NMR (500 MHz, $CDCl_3$) δ 7.84 – 7.81 (m, 4H), 7.50 – 7.44 (m, 3H), 6.24 (d, *J* = 1.4 Hz, 1H), 5.61 (d, *J* = 1.2 Hz, 1H), 5.07 (dd, *J* = 8.4, 4.0 Hz, 1H), 4.23 (qd, *J* = 7.2, 1.1 Hz, 2H), 2.91 – 2.87 (m, 1H), 2.84 (s, 1H, -OH), 2.78 – 2.73 (m, 1H), 1.31 (t, *J* = 7.2 Hz, 3H); ^{13}C NMR (125 MHz, $CDCl_3$) δ 168.0, 141.5, 137.3, 133.4, 133.1, 128.4, 128.3, 128.1, 127.8, 126.2, 125.9, 124.5, 124.1, 73.5, 61.3, 42.7, 14.3; IR (KBr, thin film): 3464, 2981, 1710, 1630, 1321, 1142, 1028, 858, 819, 747 cm^{-1}; HRMS-ESI (m/z) $[M+Na^+]$: calcd. for $C_{17}H_{18}NaO_3$ 293.1148, found 293.1155.

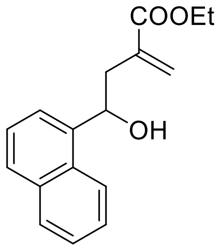

Ethyl 4-hydroxy-2-methylene-4-(naphthalen-1-yl)butanoate (25).

Light yellow oil (18.3 mg, 68% yield) after preparative thin layer chromatography separation (80% hexanes : 20% EtOAc): TLC R_f = 0.39 (EtOAc/hexanes = 1/4); ^1H NMR (500 MHz, $CDCl_3$) δ 8.22 (d, *J* = 8.5 Hz, 1H), 7.88 (d, *J* = 8.0 Hz, 1H), 7.78 (d, *J* = 8.2 Hz, 1H), 7.73 (d, *J* = 7.2 Hz, 1H), 7.56 – 7.53 (m, 1H), 7.50 – 7.47 (m, 2H), 6.31 (d, *J* = 1.4 Hz, 1H), 5.70 – 5.68 (m, 2H), 4.28 (qd, *J* = 7.1, 4.5 Hz, 2H), 3.09 – 3.06 (m, 1H), 2.77 (s, 1H, -OH), 2.68 – 2.64 (m, 1H), 1.36 (t, *J* = 7.1 Hz, 3H); ^{13}C NMR (125 MHz, $CDCl_3$) δ 167.9, 139.9, 137.5, 133.8, 130.3, 129.0, 128.6, 128.0, 126.2, 125.6, 125.6, 123.2, 122.7, 69.7, 61.3, 42.3, 14.3; IR (KBr, thin film): 3472, 2981, 1708, 1630, 1512, 1325, 1144, 1028, 802, 779 cm^{-1}; HRMS-ESI (m/z) $[M+Na^+]$: calcd. for $C_{17}H_{18}NaO_3$ 293.1148, found 293.1152.

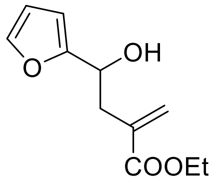

Ethyl 4-(furan-2-yl)-4-hydroxy-2-methylenebutanoate (27).

Light yellow oil (15.7 mg, 75% yield) after preparative thin layer chromatography

separation (75% hexanes : 12.5% acetone : 12.5% EtOAc): TLC R_f = 0.30 (EtOAc /acetone/hexanes = 1/1/6); ^{1}H NMR (500 MHz, $CDCl_3$) δ 7.37 (dd, *J* = 1.8, 0.8 Hz, 1H), 6.32 (dd, *J* = 3.3, 1.8 Hz, 1H), 6.26 – 6.25 (m, 2H), 5.64 (d, *J* = 1.2 Hz, 1H), 4.91 (dt, *J* = 8.2, 4.9 Hz, 1H), 4.23 (q, *J* = 7.1 Hz, 2H), 2.92 – 2.88 (m, 1H), 2.86 – 2.81 (m, 1H), 2.65 (d, *J* = 5.0 Hz, 1H, -OH), 1.31 (t, *J* = 7.1 Hz, 4H); ^{13}C NMR (125 MHz, $CDCl_3$) δ 167.7, 156.1, 142.1, 136.8, 128.4, 110.3, 106.3, 67.2, 61.3, 39.0, 14.3; IR (KBr, thin film): 3448, 2983, 1713, 1630, 1370, 1275, 1190, 1012, 949, 748 cm^{-1}; HRMS-EI (m/z) [M^+]: calcd. for $C_{11}H_{14}O_4$ 210.0892, found 210.0891.

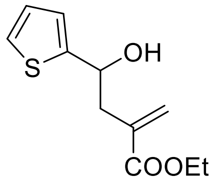

Ethyl 4-hydroxy-2-methylene-4-(thiophen-2-yl)butanoate (29).

Light yellow oil (16.0 mg, 71% yield) after preparative thin layer chromatography separation (75% hexanes : 25% acetone): TLC R_f = 0.50 (acetone/hexanes = 1/3); ^{1}H NMR (500 MHz, $CDCl_3$) δ 7.23 (dd, *J* = 4.6, 1.7 Hz, 1H), 6.97 – 6.95 (m, 2H), 6.26 (d, *J* = 1.4 Hz, 1H), 5.65 (d, *J* = 1.2 Hz, 1H), 5.15 (dd, *J* = 8.3, 4.5 Hz, 1H), 4.23 (q, *J* = 7.1 Hz, 2H), 2.91 – 2.84 (m, 2H), 2.82 – 2.78 (m, 1H), 1.32 (t, *J* = 7.1 Hz, 3H); ^{13}C NMR (125 MHz, $CDCl_3$) δ 167.7, 148.1, 136.8, 128.7, 126.8, 124.5, 123.7, 69.5, 61.3, 42.8, 14.3; IR (KBr, thin film): 3469, 2982, 2931, 1712, 1630, 1443, 1306, 1200, 1028, 700 cm^{-1}; HRMS-ESI (m/z) [$M+Na^+$]: calcd. for $C_{11}H_{14}NaO_3S$ 249.0556, found 249.0555.

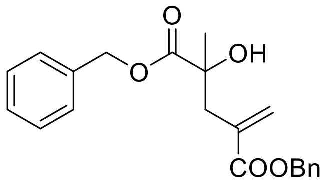

Dibenzyl 2-hydroxy-2-methyl-4-methylenepentanedioate (31).

Colourless oil (17.9 mg, 51 % yield) after preparative thin layer chromatography separation (75% hexanes : 12.5% acetone : 12.5% EtOAc): TLC R_f = 0.68

(EtOAc/acetone/hexanes = 1/1/6); ^{1}H NMR (500 MHz, $CDCl_3$) δ 7.38 – 7.30 (m, 8H), 6.26 (d, *J* = 1.3 Hz, 1H), 5.65 – 5.60 (m, 1H), 5.19 – 5.04 (m, 4H), 3.58 (s, 1H), 2.88 (d, *J* = 13.9 Hz, 1H), 2.70 (d, *J* = 13.9 Hz, 1H), 1.46 (s, 3H); ^{13}C NMR (125 MHz, $CDCl_3$) δ 175.8, 167.6, 135.9, 135.5, 135.4, 129.7, 128.7, 128.7, 128.6, 128.4, 128.4, 128.2, 74.5, 67.6, 67.0, 42.1, 25.7; IR (KBr, thin film): 3445, 2935, 1721, 1627, 1455, 1267, 1214, 1160, 962, 697 cm^{-1}; HRMS-ESI (m/z) [M+H$^+$]: calcd. for $C_{21}H_{23}O_5$ 355.1540, found 355.1538.

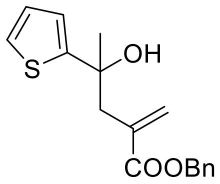

Benzyl 4-hydroxy-2-methylene-4-(thiophen-2-yl)pentanoate (33).

Colourless oil (14.4 mg, 53 % yield) after preparative thin layer chromatography separation (75% hexanes : 12.5% acetone : 12.5% EtOAc): TLC R_f = 0.74 (EtOAc/acetone/hexanes = 1/1/6); ^{1}H NMR (500 MHz, $CDCl_3$) δ 7.44 – 7.30 (m, 5H), 7.15 (dd, *J* = 5.1, 1.2 Hz, 1H), 6.92 (dd, *J* = 5.1, 3.5 Hz, 1H), 6.86 (dd, *J* = 3.5, 1.2 Hz, 1H), 6.27 (d, *J* = 1.3 Hz, 1H), 5.53 (d, *J* = 1.1 Hz, 1H), 5.18 (s, 2H), 3.83 (s, 1H), 2.91 (s, 2H), 1.61 (s, 3H); ^{13}C NMR (125 MHz, $CDCl_3$) δ 168.8, 153.3, 136.1, 135.7, 130.2, 128.7, 128.5, 128.3, 126.8, 123.8, 122.4, 73.6, 67.2, 47.2, 30.6; IR (KBr, thin film): 3445, 3066, 2975, 1714, 1625, 1455, 1383, 1164, 750, 697 cm^{-1}; HRMS-ESI (m/z) [M+Na$^+$]: calcd. for $C_{17}H_{18}NaO_3S$ 325.0869, found 325.0874.

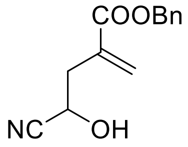

Benzyl 4-cyano-4-hydroxy-2-methylenebutanoate (35).

Light yellow oil (13.7 mg, 59% yield) after preparative thin layer chromatography separation (75% hexanes : 12.5% acetone : 12.5% DCM): TLC R_f = 0.27 (DCM /acetone/hexanes = 1/1/6); ^{1}H NMR (500 MHz, $CDCl_3$) δ 7.40 – 7.36 (m, 5H), 6.48 (d, *J* = 0.8 Hz, 1H), 5.92 (d, *J* = 1.0 Hz, 1H), 5.24 (d, *J* = 1.7 Hz, 2H), 4.74 (td, *J* = 6.8,

4.9 Hz, 1H), 3.86 (d, *J* = 6.9 Hz, 1H, -OH), 2.92 – 2.88 (m, 1H), 2.85 – 2.81 (m, 1H); ^{13}C NMR (125 MHz, $CDCl_3$) δ 167.9. 135.3, 134.1, 131.8, 128.9, 128.8, 128.5, 118.9, 67.8, 61.4, 38.6; IR (KBr, thin film): 3456, 2924, 1713, 1456, 1304, 1148, 1074, 913, 743, 699 cm^{-1}; HRMS-ESI (m/z) [$M+Na^+$]: calcd. for $C_{13}H_{13}NNaO_3$ 254.0788, found 254.0784.

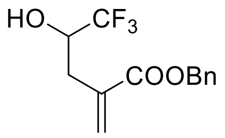

Benzyl 5,5,5-trifluoro-4-hydroxy-2-methylenepentanoate (38).

Colourless oil (10.9 mg, 39 % yield) after preparative thin layer chromatography separation (75% hexanes : 12.5% acetone : 12.5% EtOAc): TLC R_f = 0.63 (EtOAc /hexanes = 1/4); ^1H NMR (500 MHz, $CDCl_3$) δ 7.46 – 7.29 (m, 4H), 6.40 (s, 1H), 5.83 (s, 1H), 5.24 (s, 2H), 4.14 (th, *J* = 9.5, 3.3 Hz, 1H), 3.30 (d, *J* = 5.9 Hz, 1H), 2.79 (dd, *J* = 14.3, 2.8 Hz, 1H), 2.63 (dd, *J* = 14.4, 9.6 Hz, 1H). ^{13}C NMR (126 MHz, $CDCl_3$) δ 167.55, 135.34, 134.87, 130.03, 128.64, 128.46, 128.17, 70.25, 70.00, 69.80, 69.75, 69.50, 67.24, 33.38. ^{19}F NMR (376 MHz, Chloroform-*d*) δ -79.78 (d, *J* = 6.5 Hz); IR (KBr, thin film): 3446, 1714, 1697, 1455, 1415, 1318, 1213, 1171, 1127, 696 cm^{-1}; HRMS-ESI (m/z) [$M+NH_4^+$]: calcd. for $C_{13}H_{17}F_3NO_3$ 292.1155, found 292.1155.

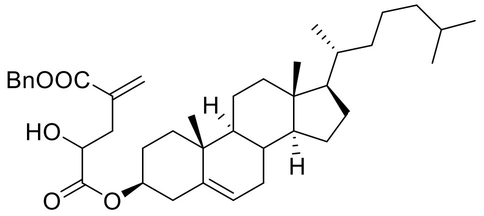

1-Benzyl 5-((3S,9S,10R,13R,14S,17R)-10,13-dimethyl-17-(6-methylheptan-2-yl)-2,3,4,7,8,9,10,11,12,13,14,15,16,17-tetradecahydro-1H-cyclopenta[a]phenanthren-3-yl) 4-hydroxy-2-methylenepentanedioate (41).

Colourless oil (38.4 mg, 62 % yield) after flash chromatography separation (EtOAc/hexanes = 1/10): TLC R_f = 0.71 (EtOAc /acetone/hexanes = 1/1/6); ^1H NMR (500 MHz, $CDCl_3$) δ 7.41 – 7.31 (m, 4H), 6.35 (d, *J* = 1.3 Hz, 1H), 5.76 (t, *J* = 1.3 Hz,

1H), 5.42 – 5.34 (m, 1H), 5.27 – 5.16 (m, 2H), 4.68 (dq, *J* = 10.7, 6.0, 5.3 Hz, 1H), 4.38 (dd, *J* = 8.2, 4.3 Hz, 1H), 2.88 (dd, *J* = 14.4, 4.3 Hz, 1H), 2.64 (dd, *J* = 14.3, 8.2 Hz, 1H), 2.32 (t, *J* = 6.7 Hz, 2H), 2.04 – 1.94 (m, 2H), 1.85 (tdd, *J* = 13.0, 6.7, 3.4 Hz, 3H), 1.64 – 1.41 (m, 8H), 1.39 – 1.21 (m, 5H), 1.20 – 1.05 (m, 7H), 1.01 (s, 6H), 0.91 (d, *J* = 6.5 Hz, 3H), 0.86 (dd, *J* = 6.6, 2.4 Hz, 6H), 0.68 (s, 3H); ^{13}C NMR (125 MHz, $CDCl_3$) δ 173.9, 166.9, 139.3, 135.9, 135.7, 129.0, 128.7, 128.4, 128.2, 123.2, 75.9, 69.5, 66.9, 56.8, 56.2, 50.1, 42.4, 39.8, 39.6, 38.1, 37.4, 37.0, 36.7, 36.3, 35.9, 32.0, 31.9, 28.4, 28.2, 27.8, 24.4, 23.9, 23.0, 22.7, 21.1, 19.4, 18.8, 12.0; IR (KBr, thin film): 3446, 2947, 2867, 1723, 1466, 1383, 1210, 1101, 749, 696 cm^{-1}; HRMS-ESI (m/z) [M+Na$^+$]: calcd. for $C_{40}H_{58}NaO_5$ 641.4176, found 641.4180.

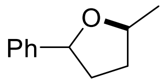

2-Methyl-5-phenyltetrahydrofuran (44).

Colorless oil (10.1 mg, 62 % yield) after flash chromatography (90% hexanes : 10% EtOAc): TLC R_f = 0.87 (EtOAc/hexanes = 1/9); ^{1}H NMR (500 MHz, $CDCl_3$) δ 8.36 – 8.23 (m, 5H), 6.05 – 5.86 (m, 1H), 5.39 – 5.14 (m, 1H), 3.39 – 3.28 (m, 1H), 3.17 – 3.07 (m, 1H), 2.90 – 2.82 (m, 1H), 2.64 – 2.59 (m, 1H), 2.34 (dd, *J* = 25.1, 6.1 Hz, 3H); ^{13}C NMR (125 MHz, $CDCl_3$) δ 144.1, 143.7, 128.4, 128.4, 127.3, 127.2, 126.0, 125.7, 81.2, 80.4, 76.1, 76.1, 35.7, 34.8, 34.4, 33.2, 21.7, 21.5.

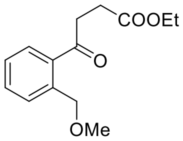

Ethyl 4-(2-(methoxymethyl)phenyl)-2-methylene-4-oxobutanoate (47-ox).

Colorless oil (7.9 mg, 30 % yield): TLC R_f = 0.65 (EtOAc/hexane = 1/4); ^{1}H NMR (500 MHz, $CDCl_3$) δ 7.79 (d, *J* = 7.7 Hz, 1H), 7.65 (d, *J* = 7.8 Hz, 1H), 7.51 (t, *J* = 7.6 Hz, 1H), 7.37 (t, *J* = 7.6 Hz, 1H), 6.41 (s, 1H), 5.69 (s, 1H), 4.71 (s, 2H), 4.21 (q, *J* = 7.1 Hz, 2H), 3.94 (s, 2H), 3.43 (s, 3H), 1.27 (t, *J* = 7.1 Hz, 3H). ^{13}C NMR (126 MHz, $CDCl_3$) δ 200.26, 166.39, 139.49, 135.97, 134.85, 131.88, 128.65, 128.54, 127.73,

126.94, 72.48, 61.03, 58.63, 44.26, 14.12. IR (KBr, thin film) 2982, 2930, 1716, 1685, 1312, 1198, 1146, 1100, 1026, 1003, 950, 760 cm^{-1}; HRMS-ESI (m/z) $[M+H^+]$: calcd. for $C_{15}H_{18}O_4$ 263.1278, found 263.1278.

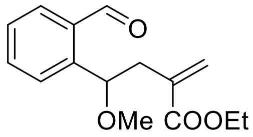

Ethyl 4-(2-formylphenyl)-4-methoxy-2-methylenebutanoate (48-ox)

Colorless oil (2.6 mg, 10 % yield): TLC Rf = 0.68 (EtOAc/hexane = 1/4); ^1H NMR (500 MHz, Chloroform-*d*) δ 10.36 (s, 1H), 7.86 (d, *J* = 7.6 Hz, 1H), 7.61 (d, *J* = 5.9 Hz, 2H), 7.45 (t, *J* = 7.0 Hz, 1H), 6.20 (s, 1H), 5.57 (s, 1H), 5.27 (dd, *J* = 8.1, 4.8 Hz, 1H), 4.18 (q, *J* = 7.1 Hz, 2H), 3.24 (s, 3H), 2.86 – 2.61 (m, 2H), 1.29 (t, *J* = 7.1 Hz, 3H). ^{13}C NMR (126 MHz, $cdcl_3$) δ 192.52, 167.03, 144.41, 136.89, 133.98, 133.91, 131.74, 127.74, 127.22, 127.21, 78.45, 60.72, 57.17, 40.51, 14.17. IR (KBr, thin film) 2982 2933 1713 1693 1599 1310 1188 1144 1098 1027 763 cm^{-1}; HRMS-ESI (m/z) $[M+H^+]$: calcd. for $C_{15}H_{18}O_4$ 263.1278, found 263.1279.

V. References

Barone, V.; Cossi, M.; Tomasi, J. (1998) Geometry optimization of molecular structures in solution by the polarizable continuum model. J. Comput. Chem. *19*, 404.

Becke, A. D. (1993) Density-functional thermochemistry. III. The role of exact exchange. J. Chem. Phys. *98*, 5648.

Cances, E.; Mennucci, B.; Tomasi, J. (1997) A new integral equation formalism for the polarizable continuum model: Theoretical background and applications to isotropic and anisotropic dielectrics. J. Chem. Phys. *107*, 3032.

Cossi, M.; Barone, V.; Cammi, R.; Tomasi, J. (1996) Ab initio study of solvated molecules: a new implementation of the polarizable continuum model. Chem. Phys. Lett. *255*, 327.

Lee, C.; Yang, W.; Parr, R. G. (1988) Development of the Colle-Salvetti correlation-energy formula into a functional of the electron density. Phys. Rev. B: Condens. Matter Mater. Phys. *37*, 785.

Liu, S.; Qi, X.; Qu, L.-B.; Bai, R. B.; Lan, Y. (2018) C–H bond cleavage occurring on a Rh(V) intermediate: a theoretical study of Rh-catalyzed arene azidation. Catal. Sci. Technol. *8*, 1645.

Marenich, A. V.; Cramer, C. J.; Truhlar, D. G. (2009) Universal Solvation Model Based on Solute Electron Density and on a Continuum Model of the Solvent Defined by the Bulk Dielectric Constant and Atomic Surface Tensions. J. Phys. Chem. B *113*, 6378.

Peverati, R.; Truhlar, D. G. (2011) Improving the accuracy of hybrid meta-GGA density functionals by range separation. J. Phys. Chem. Lett. *2*, 2810.
